# Synthesis of 1,3‐Bis‐(boryl)alkanes through Boronic Ester Induced Consecutive Double 1,2‐Migration

**DOI:** 10.1002/anie.202007541

**Published:** 2020-08-07

**Authors:** Cai You, Armido Studer

**Affiliations:** ^1^ Organisch-Chemisches Institut Westfälische Wilhelms-Universität Corrensstrasse 40 48149 Münster Germany

**Keywords:** 1,2-migration, 1,3-bis-(boryl)alkanes, reaction mechanisms, synthetic methods, vinylboron ate complexes

## Abstract

A general and efficient approach for the preparation of 1,3‐bis‐(boryl)alkanes is introduced. It is shown that readily generated vinylboron ate complexes react with commercially available ICH_2_Bpin to valuable 1,3‐bis‐(boryl)alkanes. The introduced transformation, which is experimentally easy to conduct, shows broad substrate scope and high functional‐group tolerance. Mechanistic studies reveal that the reaction does not proceed via radical intermediates. Instead, an unprecedented boronic ester induced sequential bis‐1,2‐migration cascade is suggested.

## Graphical abstract

**Two is better than one**: Commercially available ICH_2_Bpin reacts with readily prepared vinylboron ate complexes under practical conditions to valuable 1,3‐bis‐(boryl)alkanes. The cascade proceeds through two subsequent 1,2‐metallate rearrangements of boronate complexes, with the second boronate being formed in the initial 1,2‐migration step.

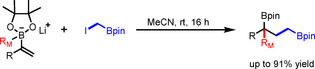

Organoboron compounds are versatile intermediates in synthesis^1^ that also play an important role in materials science and medicinal chemistry.^2^ Bis‐(boryl)alkanes, an interesting subclass, have attracted increasing attention as synthetic precursors in organic synthesis enabling multiple C−C and C–heteroatom bond construction.^3^, 4a, 4b Although many methods for accessing 1,1‐ and 1,2‐bis‐(boryl)alkanes have been reported,^4^ general methods for the synthesis of 1,3‐bis‐(boryl)alkanes are rare.3b, 5 Therefore, an efficient and general procedure for the preparation of 1,3‐bis‐(boryl)alkanes is demanded.

1,2‐migrations of boron ate complexes have been shown to be highly reliable for C−C bond construction while retaining the valuable boron moiety in the product.^6^, ^7^ In 1967, Zweifel and co‐workers first reported 1,2‐alkyl/aryl migrations of vinylboron ate complexes induced by electrophilic halogenation (Scheme 1 a).^8^ In 2016, Morken and co‐workers disclosed the electrophilic palladation‐induced 1,2‐alkyl/aryl migration of vinylboron ate complexes.^9^ More recently, we,^10^ Aggarwal,^11^ and Renaud^12^ developed radical polar crossover reactions, in which 1,2‐alkyl/aryl migrations of vinylboron ate complexes are induced by alkyl radical additions. This radical approach was further extended by Shi and co‐workers to the radical‐induced 1,2‐boron migration.^13^

**Scheme 1:**
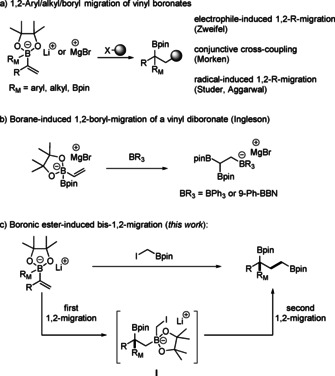
1,2‐Group migrations of vinyl boron ate complexes.

In 2018, Ingleson and co‐workers demonstrated that soft boron‐based Lewis acids (BPh_3_ and 9‐Ph‐BBN) induce 1,2‐boron migration of a vinyl diboron ate complex to enable the one‐pot synthesis of 1,1,2‐triborylated alkanes (Scheme 1 b).^14^ Inspired by this reaction, we envisioned that commercially available ICH_2_Bpin would react with vinyl boron ate complexes to form 1,3‐bis‐(boryl)alkanes (Scheme 1 c, pin=pinacolato). We considered that ICH_2_Bpin acting as a soft electrophile would induce a Zweifel‐type 1,2‐R_M_‐migration of a vinyl boron ate complex to form the 1,2‐diborylated alkane intermediate **I** that might engage in a subsequent Matteson rearrangement6b to afford a 1,3‐bis‐(boryl)alkane. This strategy comprising two sequential 1,2‐alkyl/aryl migration steps would offer a general and efficient approach for the synthesis of 1,3‐bis‐(boryl)alkanes, and first results are reported in this communication.

We began our investigations by exploring the reaction between the vinyl boron ate complex **2 a** and ICH_2_Bpin. To this end, **2 a** was generated in situ by addition of *n*‐butyllithium to the boronic ester **1 a** in diethyl ether at 0 °C. The solvent was removed and crude **2 a** was redissolved in acetonitrile. An excess (2 equivalents) of ICH_2_Bpin was added and the mixture was stirred at room temperature for 16 hours. To our delight, the desired 1,3‐bis‐(boryl)alkane **3 a** was obtained in high yield (85 %, Table 1, entry 1). Solvent screening revealed acetonitrile to be superior to all other solvents tested (entries 1–4). Upon replacing ICH_2_Bpin by BrCH_2_Bpin or ClCH_2_Bpin, the yield of **3 a** significantly dropped (entries 5 and 6). However, in the presence of 1.0 equiv of NaI, the reaction with ClCH_2_Bpin delivered **3 a** in 80 % yield (entry 7). Increasing the amount of ICH_2_Bpin led to a further improvement and the best result was obtained when 3 equivalents were used (86 % yield of isolated product; entries 8 and 9).

**Table 1:** Reaction optimization.^[a]^ 
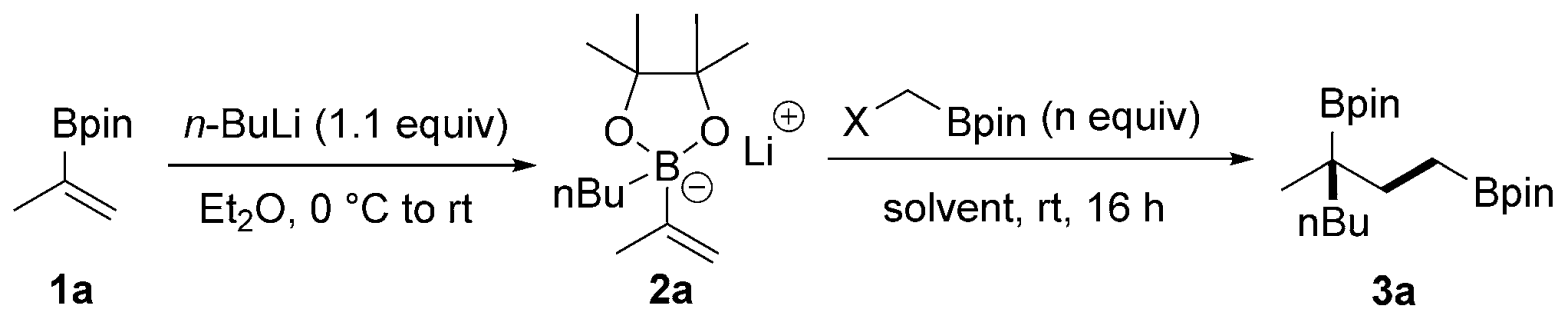

Entry	Solvent	X	*n*	Yield [%]^[b]^
1	MeCN	I	2	85
2	DMSO	I	2	43
3	THF	I	2	26
4	DMF	I	2	44
5	MeCN	Br	2	64
6	MeCN	Cl	2	5
7^[c]^	MeCN	Cl	2	80
8	MeCN	I	3	92 (86)
9	MeCN	I	4	90

[a] Reaction conditions: **1 a** (0.20 mmol), *n*‐BuLi (0.22 mmol), in Et_2_O (2 mL), 0 °C to rt, 1 h, under Ar. After boronate complex formation, solvent exchange to the selected solvent (2 mL) was performed. [b] GC yield using *n*‐C_14_H_30_ as an internal standard; yield of isolated product is given in parentheses. [c] 0.20 mmol NaI was added.

To investigate the substrate scope, various vinyl boron ate complexes were tested (Scheme 2). B‐ate complexes **2 a**–**2 e** generated by treating the boronic ester **1 a** with *n*‐butyllithium, *n*‐hexyllithium, isobutyllithium, isopropyllithium, or *tert*‐butyllithium underwent this transformation smoothly to afford **3 a**–**3 e** in 73–86 % yield, which demonstrates that the sequence tolerates different levels of steric hindrance with respect to the migrating alkyl group. By using PhLi for boronate formation, the tertiary benzylic boronic ester **3 f** was obtained (73 %). Other aryllithiums bearing various functional groups at the *para* position of the aryl moiety, such as methyl (**3 g**), *tert*‐butyl (**3 h**), trifluoromethyl (**3 i**), methoxyl (**3 j**), trimethylsilyl (**3 k**), and halides (**3 l** and **3 m**) are all compatible with this transformation. Aryl groups bearing *meta* or *ortho* substituents are tolerated, as documented by the successful preparation of **3 n**–**3 p**. We also tested a substrate containing a C=C bond at the phenyl ring, and **3 q** was obtained in 72 % yield with the less nucleophilic styrenic double bond unreacted. Substrates containing extended aromatic systems also engage in this cascade (see **3 r** and **3 s**). Isopropenylmagnesium bromide could be employed for boronate generation to afford **3 t**, albeit in a slightly lower yield (46 %). We also tested whether ICH_2_Bpin can be replaced by secondary borylated alkyl iodides. However, reaction of **2 a** with ICHCH_3_Bpin under optimized conditions afforded only traces of the targeted compound.

**Scheme 2:**
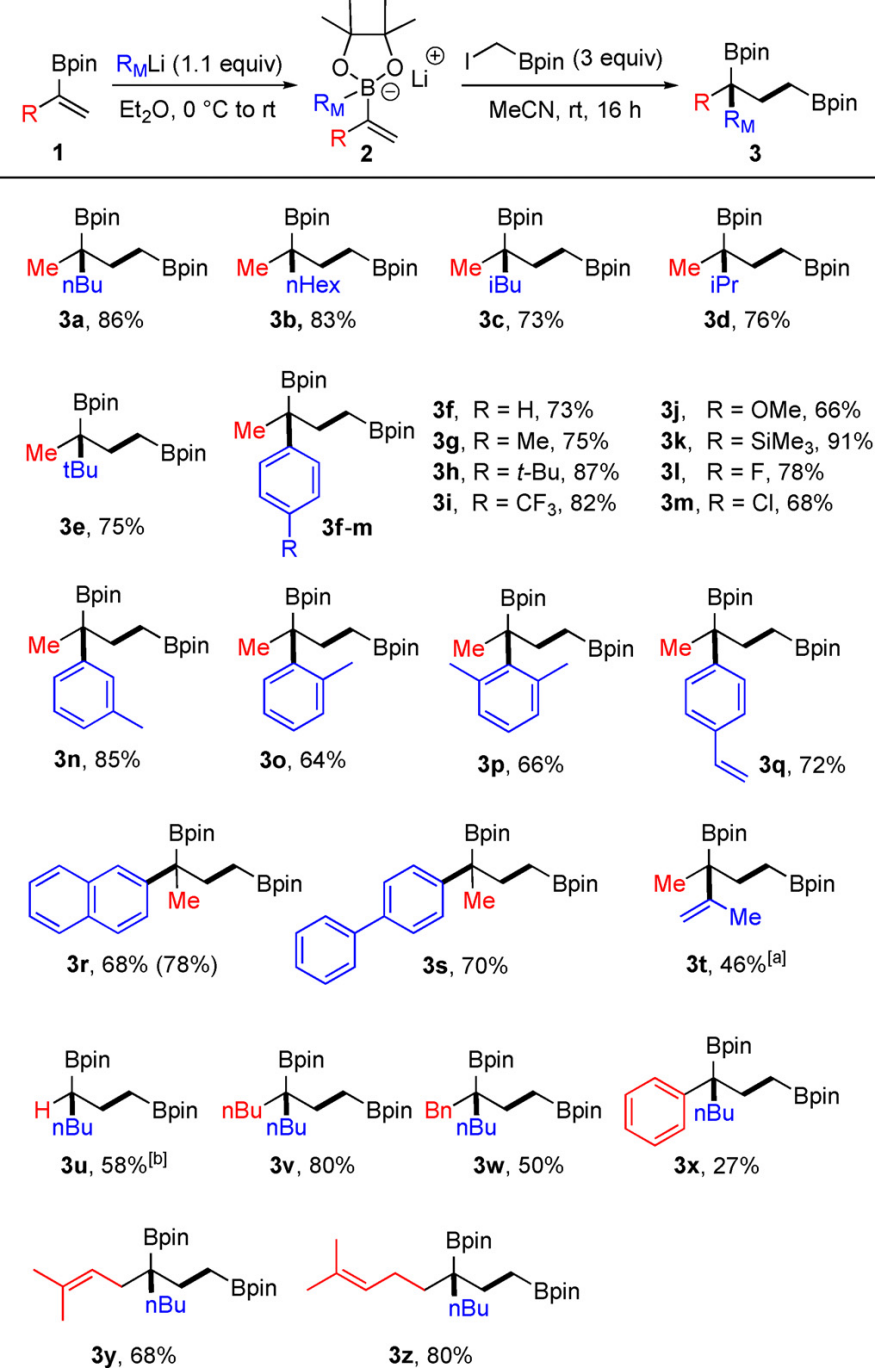
Substrate scope. Reaction conditions: **1** (0.20 mmol, 1.0 equiv), R_M_Li (0.22 mmol, 1.1 equiv), in Et_2_O (2 mL), 0 °C to rt, 1 h, under Ar; then ICH_2_Bpin (0.60 mmol, 3 equiv), rt, 16 h, in MeCN (2 mL). Yields given correspond to isolated products. Yield in parentheses for 1.2 mmol scale experiment. [a] α‐Substituted alkenyl Grignard reagent isopropenylmagnesium bromide was used. [b] Vinylboronic acid pinacol ester (0.30 mmol), *n*‐BuLi (0.33 mmol, 1.1 equiv), in Et_2_O (2 mL), 0 °C to rt, 1 h, under Ar; then 1 mol % Ir(ppy)_3_, ICH_2_Bpin (0.60 mmol, 2 equiv), rt, 16 h, in MeCN (1 mL), blue LEDs. ppy=2‐phenylpyridine.

Studies were continued by varying the R substituent at the vinyl boronic ester **1** with the *n*‐butyllithium group as the migrating substituent R_M_. When the unsubstituted vinyl boronate **2 u** was employed (R=H), trace amounts of the targeted **3 u** were formed, likely due to the lowered nucleophilicity of **2 u** compared to the α‐methyl congeners. However, upon using Ir photocatalysis, smooth reaction occurred and **3 u** was obtained in 58 % yield, likely through a different mechanism (see discussion below).

In contrast, vinylboron ate complexes bearing an activating R group at the α‐position of the double bond engaged in the cascade without the necessity of using an Ir photocatalyst. Hence, various α‐substituents R, such as *n*‐butyl (**3 v**), benzyl (**3 w**), prenyl (**3 y**), and homoprenyl (**3 z**) serve as activating groups and the corresponding products were obtained in 50–80 % yields. However, the α‐styrenyl boronate **2 x** gave a significantly lower yield of **3 x** (27 %). This is in our eyes not a serious limitation, since an α‐phenyl group in the product boronic ester can be installed through phenyl‐group migration (see **3 f**). Notably, upon running the reaction on a larger scale (1.2 mmol), an increase in yield was obtained (**3 r**, 78 %).

We also tested whether β‐substituents at the vinylic double bond of the boron ate complexes are tolerated (Scheme 3). The sterically highly hindered trimethyl derivative **2 aa** engaged in the cascade with nBuLi as reaction partner, although a drop in the yield was noted (**3 aa**, 34 %). Better yields were achieved with α,β‐disubstituted alkenylboron ate complexes. However, reactions were not stereospecific and the *cis* complex **2 ab** derived from **1 i** reacted with a 1.7:1 diastereoselectivity to bisboronic ester **3 ab**. The isomeric ate complex *trans*‐**2 ab′** derived from **1 j** provided **3 ab** with 1.3:1 diastereoselectivity. The relative configuration was not assigned but the same major isomer was formed in both transformations. 1‐Cyclopentenylboronic ester reacted with a 2.2:1 selectivity to **3 ac**.

**Scheme 3:**
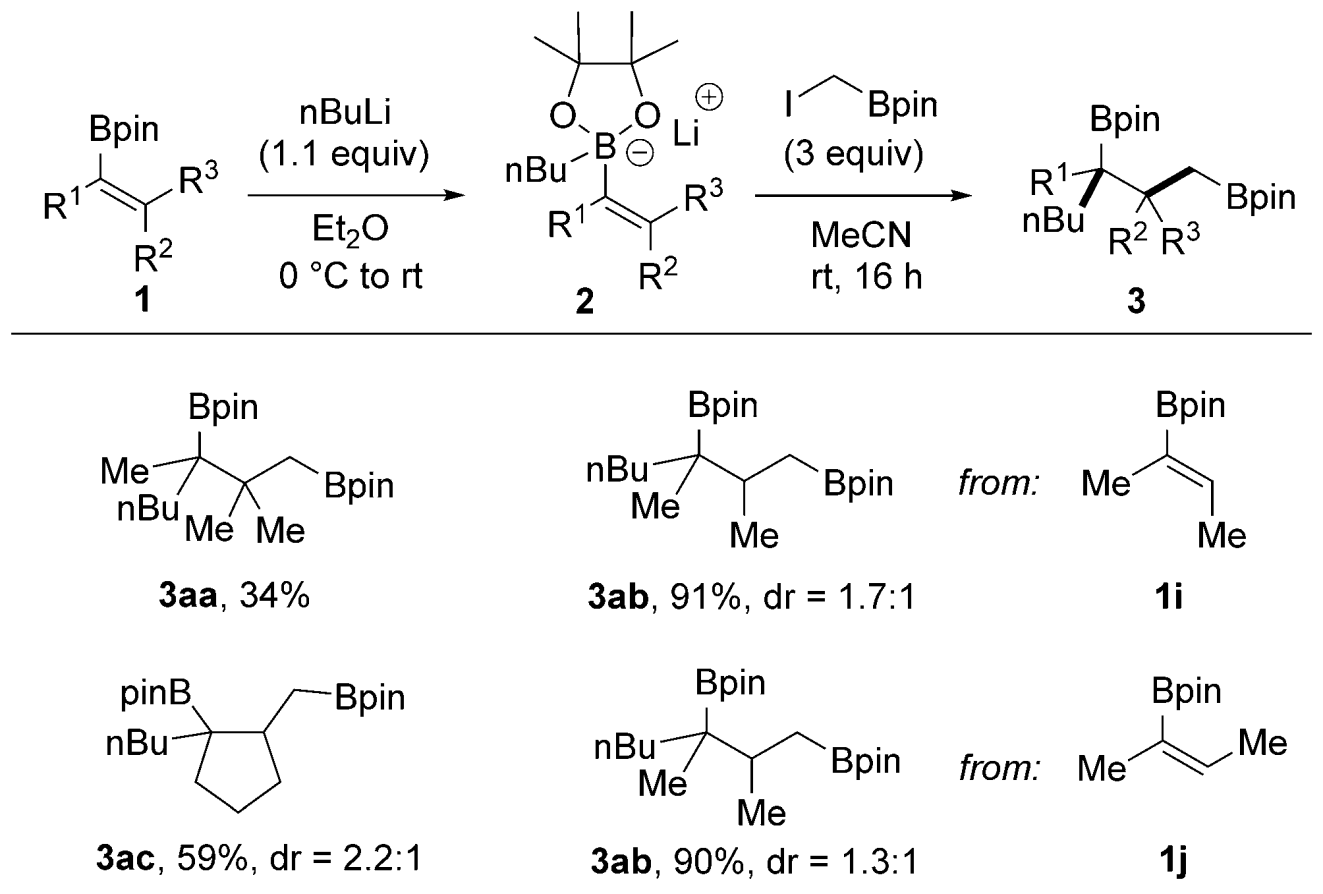
Reaction with β‐substituted boron ate complexes. Reaction conditions: **1** (0.20 mmol, 1.0 equiv), nBuLi (0.22 mmol, 1.1 equiv), in Et_2_O (2 mL), 0 °C to rt, 1 h, under Ar; then ICH_2_Bpin (0.60 mmol, 3 equiv), rt, 16 h, in MeCN (2 mL).

We next tested whether our strategy enables the preparation of triborylated alkanes. Reaction of B_2_pin_2_ with the propenyl Grignard gave bisboronate complex **2 ad** that was reacted with ICH_2_Bpin to give the targeted **3 ad**, albeit in a low yield (Scheme 4). Regarding the mechanism, we first explored the possibility of a radical‐based process^15^ by performing radical‐probe experiments^16^ (Scheme 5). Typical scavengers such as 2,2‐6,6‐tetramethyl piperidine‐N‐oxyl (TEMPO) or 3,5‐di‐*tert*‐4‐butylhydroxytoluene (BHT) did not suppress the reaction and radical trapping products could not be identified. Furthermore, the reaction of the radical probes **2 ae** and **2 af** gave the bisboronic esters **3 ae** and **3 af** in high yields. Ring‐opening products (in case of **2 ae**) or any products derived from a 5‐*exo*‐cyclization (in case of **2 af**) were not identified. These results indicate that the cascade does not occur through a radical process. Considering these findings and Ingleson's work,^14^ we suggest the following mechanism (Scheme 5 c). ICH_2_Bpin acts as soft Lewis acid, which triggers the first 1,2‐migration of the boron ate complex **2**. Since this initial 1,2‐migration does not proceed stereoselectively, the reaction likely proceeds via intermediate **A**, where the R_M_ group can migrate in a non‐concerted process to both sites of the carbenium ion. This is in agreement with the findings of Aggarwal and co‐workers, who showed that Zweifel‐type processes occur stereospecifically if induced by electrophiles that can form closed three‐membered‐ring intermediates with alkenes (onium ions).^17^ The R_M_‐1,2‐migration leads to the intermediate ate complex **B** that further reacts in a Matteson 1,2‐migration6b to give the 1,3‐bis‐(boryl)alkane **3**. However, for the less‐nucleophilic boronic ester **1 u**, where an Ir photocatalyst and light were required, the cascade likely proceeds via radical intermediates^18^ in analogy to the previously suggested radical/polar cross over additions to boronate complexes.^10^, ^11^, ^12^

**Scheme 4:**
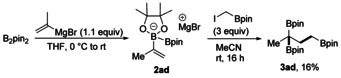
Synthesis 1,1,3‐triborylated alkanes through boronic ester induced consecutive double 1,2‐migration. Reaction conditions: B_2_pin_2_ (0.20 mmol, 1.0 equiv), isopropenylmagnesium bromide (0.22 mmol, 1.1 equiv), in THF (2 mL), 0 °C to rt, 1 h, under Ar; then ICH_2_Bpin (0.60 mmol, 3 equiv), rt, 16 h, in MeCN (2 mL).

**Scheme 5:**
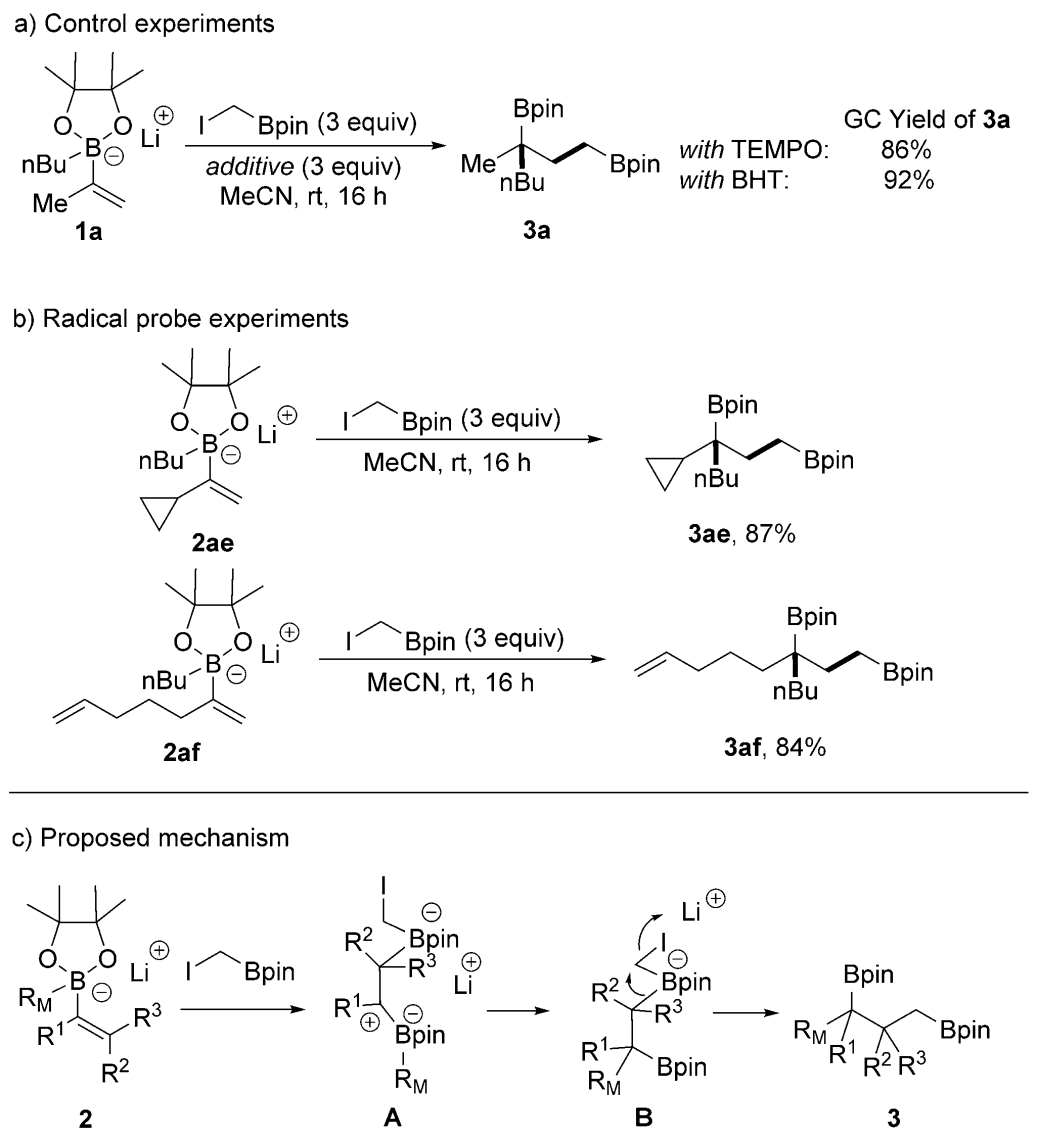
Mechanistic studies and suggested mechanism.

To demonstrate the synthetic utility, three follow‐up transformations were conducted on the product bisboronic esters (Scheme 6). Under Matteson conditions, 1,3‐bis‐(boryl)alkane **3 r** was successfully converted in a double homologation sequence into the 1,5‐bis‐(boryl)alkane **4** (63 %). Treatment of **3 r** with a NaOH‐H_2_O_2_ mixture provided the 1,3‐diol **5** in 90 % yield. Selective protodeboronation^19^ of **3 r** was achieved with *n*‐butylammonium fluoride trihydrate and the boronic ester **6** was obtained in 54 % yield.

**Scheme 6:**
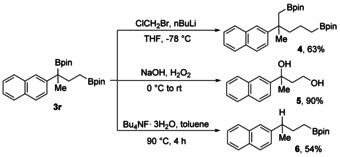
Synthetic transformations.

In summary, we have developed a process for the preparation of 1,3‐bis‐(boryl)alkanes. Commercially available ICH_2_Bpin reacts with readily prepared vinyl boron ate complexes to afford the corresponding valuable 1,3‐bisboronic esters. The cascade is operationally easy to conduct and features broad substrate scope and high functional‐group tolerance. Mechanistic investigations revealed that that the process does not proceed via radical intermediates. An ionic unprecedented boronic ester ICH_2_Bpin‐induced bis‐1,2‐migration mechanism is suggested. The value of the method was documented by successful follow‐up reactions.

## Conflict of interest

The authors declare no conflict of interest.

## Supporting information

As a service to our authors and readers, this journal provides supporting information supplied by the authors. Such materials are peer reviewed and may be re‐organized for online delivery, but are not copy‐edited or typeset. Technical support issues arising from supporting information (other than missing files) should be addressed to the authors.

Supplementary
