# Supplementary material for: Synthesis of 1,3‐Bis‐(boryl)alkanes through Boronic Ester Induced Consecutive Double 1,2‐Migration
Source: Angew Chem Int Ed Engl. 2020 Aug 7;59(39):17245–9. doi: 10.1002/anie.202007541 (PMC7540398; doi:10.1002/anie.202007541)
Supplement: Supplementary file 1 — Supplementary [file ANIE-59-17245-s001.pdf]

Angewandte Chemie
Eine Zeitschrift der Gesellschaft Deutscher Chemiker

Supporting Information

Synthesis of 1,3-Bis-(boryl)alkanes through Boronic Ester Induced Consecutive Double 1,2-Migration

*Cai You and Armido Studer**

anie_202007541_sm_miscellaneous_information.pdf

Contents

1 General information……………………………………………………………………….S2

2 Preparation and data of substrates…………………………………………………………S3

3 General procedure and characterization data for the products……………………………S5

4 Mechanistic studies………………………………………………………………………S19

5 Synthetic transformations…………………………………………………………………S21

6 References…………………………………………………………………………………S24

7 NMR spectra………………………………………………………………………………..S25

1. General Information

All reactions involving air- or moisture-sensitive reagents or intermediates were carried out in flame-dried glassware under an argon atmosphere using standard Schlenk techniques. Solvents used in reactions were either freshly distilled or obtained in extra-dry grade from commercial sources. Diethyl ether (Et_2O) was refluxed over K and freshly distilled from K-Na-alloy (4:1) afterwards. Tetrahydrofuran (THF) was refluxed over Na and distilled from K afterwards. All commercially available reagents were purchased from TCI, Sigma-Aldrich, Alfa Aesar, Acros or ABCR in the highest purity grade and used directly without further purification. Thin layer chromatography (TLC) was performed on Merck silica gel 60 F-254 plates and visualized by fluorescence quenching under UV light or staining with the standard solution of $KMnO_4$. Column chromatography was performed on Merck or Fluka silica gel 60 (40-63 μm). ^{1}H NMR, ^{13}C NMR, ^{11}B NMR and ^{19}F NMR spectra were recorded on Bruker DPX 300 spectrometer (300 MHz) or Bruker AV 400 (400 MHz). Chemical shifts (δ in ppm) were referenced on the residual peak of $CDCl_3$ (^{1}H NMR: δ = 7.26; ^{13}C NMR: δ = 77.0) or on an external standard ($CFCl_3$: ^{19}F NMR: δ = 0.0). Coupling constants were reported as Hertz (Hz), signal shapes and splitting patterns were indicated as follows: s, singlet; brs, broad singlet; d, doublet; t, triplet; q, quartet; m, multiplet. Melting points (MP) were determined with a Stuart SMP10 and are uncorrected. Infrared spectra (IR) were measured on a Digilab 3100 FT-IR Excalibur Series spectrometer and the position of the absorption bands is given in wave numbers ν (cm^{-1}). The method used for GCMS was: start at 50 °C and 1 ml/min, 1.85 psi, increase to 300 °C at 10 °C/min, hold for 15 min. Gas Chromatography (GC) was performed on an Hewlett Packard HP 6890 series GC system using an Agilent HP-1 column (30 m x 0.32 mm x 0.25 μm film thickness). The method used for GC was: start at 50 °C and 1.5 ml/min, 3.81 psi, increase to 300 °C at 10 °C/min, hold for 15 min. Mass spectra were recorded on a Finnigan MAT 4200S, a Bruker Daltonics Micro Tof, a Waters-Micromass Quatro LCZ (ESI); peaks are given in m/z (% of basis peak).

2. Preparation and Data of Substrates

1a, **1b**, **1e**, ICH_2Bpin, $BrCH_2$Bpin and $ClCH_2$Bpin were purchased from commercial source and used without further purification. **1c**,[1] **1d**,[1] **1g**,[2] **1h**,[3] **1i**,[4] **1j**,[5], **1k**[6] and **1m**[7] were prepared following literature procedures.

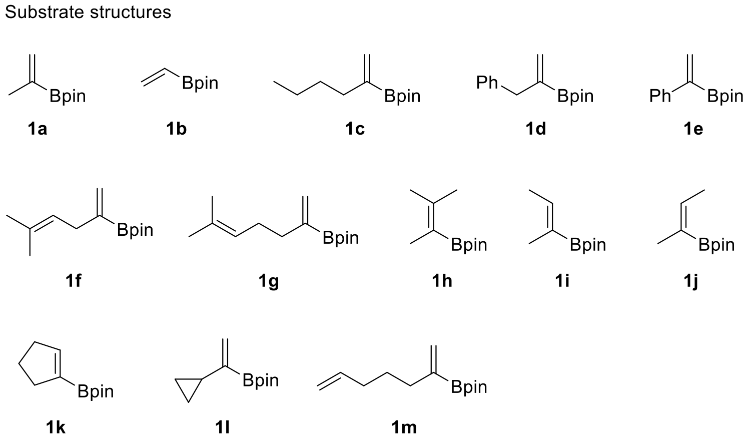

Preparation of vinyl boronic ester **1f**:

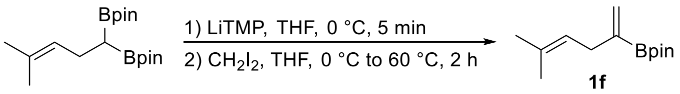

The vinyl boronic ester **1f** was prepared according to the literature procedure.[7] A 100-mL oven-dried flask with a stir bar was placed under nitrogen. The flask was charged with 2,2,6,6-tetramethylpiperidine (9 mmol, 1.2 equiv.) and 15 mL of THF. The flask was cooled to 0 °C. n-BuLi (9 mmol, 1.6 M in hexanes, 1.2 equiv.) was added via syringe. The reaction mixture was stirred for 15 minutes at 0 °C. Then, a solution of 2,2'-(4-methylpent-3-ene-1,1-diyl)bis(4,4,5,5-tetramethyl-1,3,2-dioxaborolane) (prepared according to the literature procedure[8] with bis(4,4,5,5-tetramethyl-1,3,2-dioxaborolan-2-yl)methane and 5-bromopent-1-ene) (7.5 mmol, 1.0 equiv) in THF (15 mL) was added. The reaction mixture was allowed to stir for 5 minutes at 0 °C. Next, a solution of diiodomethane (15 mmol, 2.0 equiv) in THF (7 mL) was added dropwise at 0 °C. The reaction vial was allowed to warm to 60 °C and stir for additional 2 hours. Upon completion, the reaction mixture was concentrated under reduced pressure. The crude mixture was purified by silica gel chromatography (pentane/Et_2O = 30:1) to afford **1f** (1.1 g, 66% yield) as a colorless oil.

4,4,5,5-Tetramethyl-2-(5-methylhexa-1,4-dien-2-yl)-1,3,2-dioxaborolane (1f):

^{1}H NMR (300 MHz, $CDCl_3$) δ 5.83 – 5.70 (m, 1H), 5.59 (s, 1H), 5.20 – 5.13 (m, 1H), 2.83 (d, J = 7.3 Hz, 2H), 1.71 (d, J = 0.9 Hz, 3H), 1.61 (s, 3H), 1.26 (s, 12H). ^{13}C NMR (75 MHz, $CDCl_3$) δ 132.3, 128.3, 122.4, 83.3, 33.5, 25.8, 24.8, 17.7 ppm. ^{11}B NMR (96 MHz, $CDCl_3$) δ 30.2 ppm. **HRMS** (ESI): Exact mass calculated for $C_{13}H_{23}BNaO_2^+$ ([M+Na]$^+$): 245.1683, mass found: 245.1683. **FTIR** (neat): υ (cm^{-1}) 2981, 2930, 1428, 1361, 1306, 1134, 971, 933, 865.

Preparation of vinyl boronic ester **1l**:

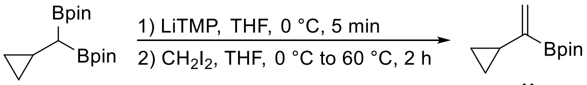

The vinyl boronic ester **1l** was prepared according to the literature procedure.[7] A 100-mL oven-dried flask with a stir bar was placed under nitrogen. The flask was charged with 2,2,6,6-tetramethylpiperidine (7.6 mmol, 1.2 equiv.) and 12 mL of THF. The flask was cooled to 0 °C. n-BuLi (7.6 mmol, 1.6 M in hexanes, 1.2 equiv.) was added via syringe. The reaction mixture was stirred for 15 minutes at 0 °C. Then, a solution of 2,2'-(cyclopropylmethylene)bis(4,4,5,5-tetramethyl-1,3,2-dioxaborolane) (prepared according to the literature procedure[9] with bis(4,4,5,5-tetramethyl-1,3,2-dioxaborolan-2-yl)methane and 5-bromopent-1-ene) (6.3 mmol, 1.0 equiv) in THF (10 mL) was added. The reaction mixture was allowed to stir for 5 minutes at 0 °C. Next, a solution of diiodomethane (12.6 mmol, 2.0 equiv) in THF (7 mL) was added dropwise at 0 °C. The reaction vial was allowed to warm to 60 °C and stir for additional 2 hours. Upon completion, the reaction mixture was concentrated under reduced pressure. The crude mixture was purified by silica gel chromatography (pentane/Et_2O = 30:1) to afford **1l** (0.31 g, 25% yield) as a colorless oil.

2-(1-Cyclopropylvinyl)-4,4,5,5-tetramethyl-1,3,2-dioxaborolane (1l):

^{1}H NMR (300 MHz, $CDCl_3$) δ 5.64 (d, J = 3.0 Hz, 1H), 5.49 (s, 1H), 1.55 – 1.47 (m, 1H), 1.26 (s, 12H), 0.70 – 0.64 (m, 2H), 0.61 – 0.54 (m, 2H). ^{13}C NMR (75 MHz, $CDCl_3$) δ 125.2, 83.2, 24.8, 15.4, 7.5 ppm. ^{11}B NMR (96 MHz, $CDCl_3$) δ 30.0 ppm. **FTIR** (neat): υ (cm^{-1}) 2986, 1426, 1405, 1389, 1329, 1305, 1208, 1134, 969, 847. The spectroscopic data are in accordance to those reported in the literature[10].

3. General Procedure and Characterization Data for the Products

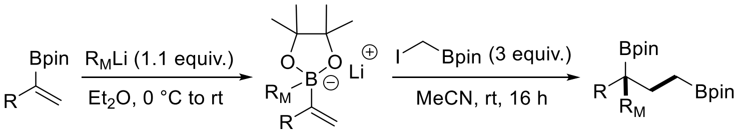

General Procedure A: Vinyl boronic ester (0.20 mmol, 1.0 equiv.) was dissolved in diethyl ether (2.0 mL) and the alkyl/aryllithium solution (0.22 mmol, 1.1 equiv.) was added dropwise over 5 minutes at 0 °C. The solution was then stirred for 0.5 h at 0 °C, warmed to room temperature and stirred for a further 0.5 h. Subsequently, the solvent was carefully removed *in vacuo* and the resulting residue was taken up in acetonitrile (2.0 mL). After the addition of ICH_2Bpin (0.60 mmol, 3.0 equiv.), the tube was sealed and the mixture was stirred at room temperature for 16 h. The reaction mixture was filtered through a pad of silica and rinsed with 30 mL Et_2O. Flash column chromatography eluting with pentane and Et_2O (pentane/Et_2O = 10:1) afforded the desired product. *Caution: In order to get a good yield, the chromatography should be finished within 10 min.*

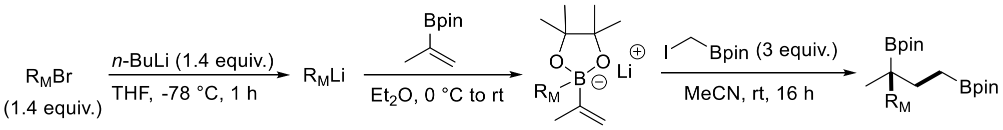

General Procedure B: To a solution of arylbromide (for **3m**, 1-chloro-4-iodobenzene was used) (0.28 mmol, 1.4 equiv.) in THF (1.5 mL) at -78 °C was added a solution of *n*-butyllithium (1.6 M, 0.26 mmol, 1.3 equiv.) over a period of 5 minutes. The solution was then stirred for 1 h at -78 °C, at which point a solution of isopropenylboronic acid pinacol ester **1a** (0.20 mmol, 1.0 equiv.) in THF (0.50 mL) was added dropwise. The solution was then stirred for 30 min at -78 °C, warmed to r.t. and stirred for a further 30 min. Subsequently, the solvent was carefully removed *in vacuo* and the resulting residue was taken up in acetonitrile (2.0 mL). After the addition of ICH_2Bpin (0.60 mmol, 3.0 equiv.), the tube was sealed and the mixture was stirred at room temperature for 16 h. The reaction mixture was filtered through a pad of silica and rinsed with 30 mL Et_2O. Flash column chromatography eluting with pentane and Et_2O (pentane/Et_2O = 10:1) afforded the desired product. *Caution: In order to get a good yield, the chromatography should be finished within 10 min.*

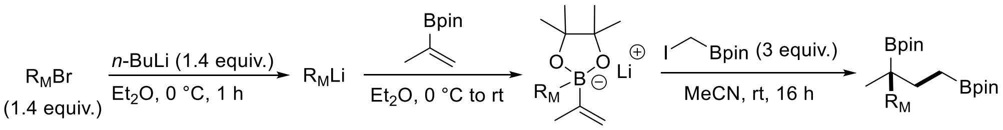

General Procedure C: To a solution of arylbromide (0.28 mmol, 1.4 equiv.) in Et_2O (1.5 mL) at 0 °C was added a solution of *n*-butyllithium (1.6 M, 0.26 mmol, 1.3 equiv.) over a period of 5 minutes. The mixture was stirred at that temperature for 1 h. After warming up to room temperature the aryllithium solution was added dropwise over 5 minutes to vinyl boronic ester (0.20 mmol, 1.0 equiv.) in diethyl ether (1.0 mL) at 0 °C. The solution was then stirred for 0.5 h at 0 °C, warmed to room temperature and stirred for a further 0.5 h. Subsequently, the solvent was carefully removed *in vacuo* and the resulting residue was taken up in acetonitrile (2.0 mL). After the addition of ICH_2Bpin (0.60 mmol, 3.0 equiv.), the tube was sealed and the mixture was stirred at room temperature for 16 h. The reaction mixture was filtered through a pad of silica and rinsed with 30 mL Et_2O. Flash column chromatography eluting with pentane and Et_2O (pentane/Et_2O = 10:1) afforded the desired product. *Caution: In order to get a good yield, the chromatography should be finished within 10 min.*

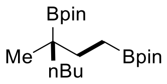

2,2'-(3-Methylheptane-1,3-diyl)bis(4,4,5,5-tetramethyl-1,3,2-dioxaborolane) (3a):

According to the General Procedure A, **3a** (63.0 mg, 86%) was prepared as a colorless sticky oil. ^{1}H NMR (300 MHz, $CDCl_3$) δ 1.60 – 1.47 (m, 1H), 1.45 – 1.05 (m, 31H), 0.96 – 0.80 (m, 6H), 0.78 – 0.61 (m, 2H). ^{13}C NMR (75 MHz, $CDCl_3$) δ 82.8, 82.7, 38.7, 32.7, 28.2, 24.9, 24.8, 23.7, 20.8, 14.1 ppm, *carbons attached to borons not observed.* ^{11}B NMR (96 MHz, $CDCl_3$) δ 35.1 ppm. **HRMS** (ESI): Exact mass calculated for $C_{20}H_{40}B_2NaO_4^+$ ([M+Na]$^+$): 389.3005, mass found: 389.3006. **FTIR** (neat): υ (cm^{-1}) 2980, 2929, 2859, 1469, 1371, 1308, 1272, 1215, 1145, 968, 856.

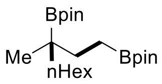

2,2'-(3-Methylnonane-1,3-diyl)bis(4,4,5,5-tetramethyl-1,3,2-dioxaborolane) (3b):

According to the General Procedure A, **3b** (65.2 mg, 83%) was prepared as a colorless sticky oil. ^{1}H NMR (300 MHz, $CDCl_3$) δ 1.57 – 1.47 (m, 1H), 1.37 – 1.10 (m, 35H), 0.92 – 0.83 (m, 6H), 0.77

– 0.62 (m, 2H). ^{13}C NMR (75 MHz, $CDCl_3$) δ 82.8, 82.7, 39.1, 32.7, 31.8, 30.3, 25.9, 24.9, 24.8, 24.8, 22.6, 20.8, 14.1 ppm, *carbons attached to borons not observed.* ^{11}B NMR (96 MHz, $CDCl_3$) δ 35.0 ppm. **HRMS** (ESI): Exact mass calculated for $C_{22}H_{44}B_2NaO_4^+$ ($[M+Na]^+$): 417.3318, mass found: 417.3316. **FTIR** (neat): υ (cm^{-1}) 2978, 2929, 2859, 1468, 1371, 1305, 1272, 1145, 968, 855.

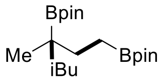

2,2'-(3,5-Dimethylhexane-1,3-diyl)bis(4,4,5,5-tetramethyl-1,3,2-dioxaborolane) (3c):

According to the General Procedure A, **3c** (53.7 mg, 73%) was prepared as a colorless sticky oil. ^{1}H NMR (300 MHz, $CDCl_3$) δ 1.69 – 1.44 (m, 2H), 1.39 – 1.20 (m, 26H), 1.13 – 1.06 (m, 1H), 0.90 – 0.83 (m, 9H), 0.80 – 0.61 (m, 2H). ^{13}C NMR (75 MHz, $CDCl_3$) δ 82.8, 82.7, 47.9, 33.5, 25.7, 25.0, 24.9, 24.8, 24.8, 24.5, 24.0, 20.9 ppm, *carbons attached to borons not observed.* ^{11}B NMR (96 MHz, $CDCl_3$) δ 35.2 ppm. **HRMS** (ESI): Exact mass calculated for $C_{20}H_{40}B_2NaO_4^+$ ($[M+Na]^+$): 389.3005, mass found: 389.3005. **FTIR** (neat): υ (cm^{-1}) 2979, 2954, 2870, 1469, 1371, 1307, 1145, 968, 849.

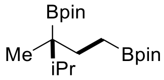

2,2'-(3,4-Dimethylpentane-1,3-diyl)bis(4,4,5,5-tetramethyl-1,3,2-dioxaborolane) (3d):

According to the General Procedure A, **3d** (53.4 mg, 76%) was prepared as a yellow solid, m.p. = 48-50 °C. ^{1}H NMR (300 MHz, $CDCl_3$) δ 1.70 – 1.61 (m, 1H), 1.59 – 1.49 (m, 1H), 1.37 – 1.18 (m, 25H), 0.92 – 0.86 (m, 3H), 0.82 – 0.78 (m, 6H), 0.77 – 0.54 (m, 2H). ^{13}C NMR (75 MHz, $CDCl_3$) δ 82.8, 82.7, 33.4, 30.8, 25.0, 25.0, 24.8, 24.8, 20.0, 17.1, 16.4 ppm, *carbons attached to borons not observed.* ^{11}B NMR (96 MHz, $CDCl_3$) δ 34.7 ppm. **HRMS** (ESI): Exact mass calculated for $C_{19}H_{38}B_2NaO_4^+$ ($[M+Na]^+$): 375.2848, mass found: 375.2855. **FTIR** (neat): υ (cm^{-1}) 2978, 2961, 2927, 1467, 1371, 1304, 1273, 1145, 969.

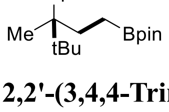

2,2'-(3,4,4-Trimethylpentane-1,3-diyl)bis(4,4,5,5-tetramethyl-1,3,2-dioxaborolane) (3e):

According to the General Procedure A, **3e** (55.0 mg, 76%) was prepared as a white solid, m.p. = 100-102 °C. ^{1}H NMR (300 MHz, $CDCl_3$) δ 1.79 (td, J = 12.4, 5.1 Hz, 1H), 1.46 – 1.06 (m, 25H), 0.89 (s, 9H), 0.86 (s, 3H), 0.78 – 0.51 (m, 2H). ^{13}C NMR (75 MHz, $CDCl_3$) δ 82.8, 82.7, 34.7, 27.2,

27.0, 25.1, 25.0, 24.8, 24.8, 16.4 ppm, *carbons attached to borons not observed.* ^{11}B NMR (96 MHz, $CDCl_3$) δ 34.3 ppm. **HRMS** (ESI): Exact mass calculated for $C_{20}H_{40}B_2NaO_4^+$ ($[M+Na]^+$): 389.3005, mass found: 389.3007. **FTIR** (neat): υ (cm^{-1}) 2978, 2951, 1468, 1371, 1363, 1348, 1317, 1299, 1216, 1166, 1145, 1087, 968, 886, 858, 847.

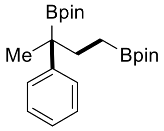

2,2'-(3-Phenylbutane-1,3-diyl)bis(4,4,5,5-tetramethyl-1,3,2-dioxaborolane) (3f):

According to the General Procedure A, **3f** (56.4 mg, 73%) was prepared as a white solid, m.p. = 87-89 °C. ^{1}H NMR (300 MHz, $CDCl_3$) δ 7.34 – 7.29 (m, 2H), 7.29 – 7.20 (m, 2H), 7.17 – 7.05 (m, 1H), 1.96 – 1.74 (m, 2H), 1.32 (s, 3H), 1.26 – 1.14 (m, 24H), 0.78 – 0.56 (m, 2H). ^{13}C NMR (75 MHz, $CDCl_3$) δ 147.1, 127.9, 127.1, 124.9, 83.2, 82.8, 33.3, 24.8, 24.6, 24.6, 20.8 ppm, *carbons attached to borons not observed.* ^{11}B NMR (96 MHz, $CDCl_3$) δ 34.6 ppm. **HRMS** (ESI): Exact mass calculated for $C_{22}H_{36}B_2NaO_4^+$ ($[M+Na]^+$): 409.2692, mass found: 409.2693. **FTIR** (neat): υ (cm^{-1}) 2978, 2930, 1468, 1371, 1310, 1272, 1144, 968, 849, 701.

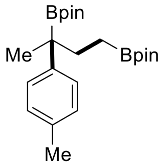

2,2'-(3-(*p*-Tolyl)butane-1,3-diyl)bis(4,4,5,5-tetramethyl-1,3,2-dioxaborolane) (3g):

According to the General Procedure B, **3g** (60.2 mg, 75%) was prepared as a white solid, m.p. = 58-60 °C. ^{1}H NMR (300 MHz, $CDCl_3$) δ 7.23 – 7.16 (m, 2H), 7.08 – 7.04 (m, 2H), 2.28 (s, 3H), 1.93 – 1.70 (m, 2H), 1.30 (s, 3H), 1.24 – 1.17 (m, 24H), 0.76 – 0.56 (m, 2H). ^{13}C NMR (75 MHz, $CDCl_3$) δ 144.0, 134.1, 128.6, 126.9, 83.1, 82.7, 33.4, 29.7, 24.8, 24.6, 24.6, 20.9, 20.9 ppm, *carbons attached to borons not observed.* ^{11}B NMR (96 MHz, $CDCl_3$) δ 34.7 ppm. **HRMS** (ESI): Exact mass calculated for $C_{23}H_{38}B_2NaO_4^+$ ($[M+Na]^+$): 423.2848, mass found: 423.2847. **FTIR** (neat): υ (cm^{-1}) 2978, 2929, 1467, 1371, 1308, 1270, 1144, 968, 849, 816.

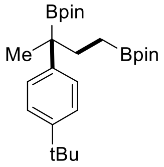

2,2'-(3-(4-(*tert*-Butyl)phenyl)butane-1,3-diyl)bis(4,4,5,5-tetramethyl-1,3,2-dioxaborolane) (3h):

According to the General Procedure B, **3h** (77.0 mg, 87%) was prepared as a white solid, m.p. = 87-89 °C. ^{1}H NMR (300 MHz, $CDCl_3$) δ 7.38 – 7.21 (m, 4H), 1.96 – 1.73 (m, 2H), 1.34 (s, 3H), 1.32 (s, 9H), 1.26 – 1.21 (m, 24H), 0.82 – 0.60 (m, 2H). ^{13}C NMR (75 MHz, $CDCl_3$) δ 147.3, 143.9, 126.6, 124.8, 83.1, 82.7, 34.2, 33.6, 31.4, 24.8, 24.7, 24.6, 21.0 ppm, *carbons attached to borons not observed.* ^{11}B NMR (96 MHz, $CDCl_3$) δ 34.3 ppm. **HRMS** (ESI): Exact mass calculated for $C_{26}H_{44}B_2NaO_4^+$ ($[M+Na]^+$): 465.3318, mass found: 465.3319. **FTIR** (neat): υ (cm^{-1}) 2977, 2934, 2869, 1468, 1371, 1309, 1271, 1145, 967, 848.

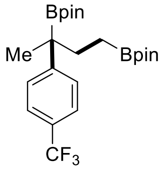

2,2'-(3-(4-(Trifluoromethyl)phenyl)butane-1,3-diyl)bis(4,4,5,5-tetramethyl-1,3,2-dioxaborolane) (3i):

According to the General Procedure B, **3i** (74.6 mg, 82%) was prepared as a yellow solid, m.p. = 80-82 °C. ^{1}H NMR (300 MHz, $CDCl_3$) δ 7.50 (d, J = 8.4 Hz, 2H), 7.41 (d, J = 8.4 Hz, 2H), 1.98 – 1.75 (m, 2H), 1.33 (s, 3H), 1.28 – 1.16 (m, 24H), 0.76 – 0.49 (m, 2H). ^{13}C NMR (75 MHz, $CDCl_3$) δ 151.4, 127.42, 127.2 (d, J = 32.3 Hz), 124.8 (q, J = 3.7 Hz), 124.6 (q, J = 271.6 Hz), 83.5 82.9, 33.2, 24.8, 24.6, 24.6, 20.6 ppm, *carbons attached to borons not observed.* ^{19}F NMR (282 MHz, $CDCl_3$) δ -62.2 ppm. ^{11}B NMR (96 MHz, $CDCl_3$) δ 34.1 ppm. **HRMS** (ESI): Exact mass calculated for $C_{23}H_{35}B_2F_3NaO_4^+$ ($[M+Na]^+$): 477.2566, mass found: 477.2566. **FTIR** (neat): υ (cm^{-1}) 2979, 2929, 1372, 1327, 1165, 1144, 1122, 1074, 1016, 967, 847.

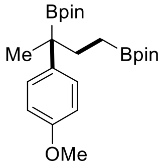

2,2'-(3-(4-Methoxyphenyl)butane-1,3-diyl)bis(4,4,5,5-tetramethyl-1,3,2-dioxaborolane) (3j): According to the General Procedure B, **3j** (54.9 mg, 66%) was prepared as a white solid, m.p. = 66-68 °C. ^{1}H NMR (300 MHz, $CDCl_3$) δ 7.26 – 7.19 (m, 2H), 6.84 – 6.77 (m, 2H), 3.77 (s, 3H), 1.91 – 1.70 (m, 2H), 1.29 (s, 3H), 1.24 – 1.17 (m, 24H), 0.75 – 0.55 (m, 2H). ^{13}C NMR (75 MHz, $CDCl_3$) δ 157.0, 139.1, 128.0, 113.4, 83.1, 82.8, 55.1, 33.4, 24.8, 24.6, 24.6, 21.0 ppm, *carbons attached to borons not observed.* ^{11}B NMR (96 MHz, $CDCl_3$) δ 34.9 ppm. **HRMS** (ESI): Exact mass calculated for $C_{23}H_{38}B_2NaO_5^+$ ($[M+Na]^+$): 439.2798, mass found: 439.2806. **FTIR** (neat): υ (cm^{-1}) 2980, 2937, 2932, 1512, 1372, 1307, 1248, 1145, 968, 847.

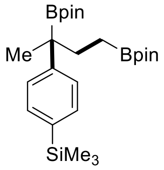

(4-(2,4-Bis(4,4,5,5-tetramethyl-1,3,2-dioxaborolan-2-yl)butan-2-yl)phenyl) trimethylsilane (3k):

According to the General Procedure B **3k** (83.7 mg, 91%) was prepared as a white solid, m.p. = 87-89 °C. ^{1}H NMR (300 MHz, $CDCl_3$) δ 7.44 – 7.38 (m, 2H), 7.33 – 7.27 (m, 2H), 1.95 – 1.73 (m, 2H), 1.32 (s, 3H), 1.23 – 1.19 (m, 24H), 0.78 – 0.57 (m, 2H), 0.24 (s, 9H). ^{13}C NMR (75 MHz, $CDCl_3$) δ 147.8, 136.0, 133.0, 126.5, 83.2, 82.8, 33.5, 24.8, 24.7, 24.6, 20.9, -1.0 ppm, *carbons attached to borons not observed.* ^{11}B NMR (96 MHz, $CDCl_3$) δ 34.8 ppm. **HRMS** (ESI): Exact mass calculated for $C_{25}H_{44}B_2NaO_4Si^+$ ($[M+Na]^+$): 481.3087, mass found: 481.3089. **FTIR** (neat): υ (cm^{-1}) 2978, 2957, 1465, 1371, 1311, 1249, 1144, 968, 848, 819.

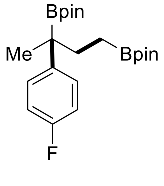

2,2'-(3-(4-Fluorophenyl)butane-1,3-diyl)bis(4,4,5,5-tetramethyl-1,3,2-dioxaborolane) (3l):

According to the General Procedure B, **3l** (63.2 mg, 78%) was prepared as a white solid, m.p. = 69-71 °C. ^{1}H NMR (300 MHz, $CDCl_3$) δ 7.34 – 7.24 (m, 2H), 7.03 – 6.90 (m, 2H), 1.94 – 1.75 (m, 2H), 1.33 (s, 3H), 1.26 – 1.20 (m, 24H), 0.79 – 0.54 (m, 2H). ^{13}C NMR (75 MHz, $CDCl_3$) δ 160.7 (d, *J* = 242.6 Hz), 142.6 (d, *J* = 3.1 Hz), 128.4 (d, *J* = 7.6 Hz), 114.5 (d, *J* = 20.7 Hz), 83.3, 82.8, 33.4, 24.8, 24.6, 24.6, 20.9 ppm, *carbons attached to borons not observed.* ^{19}F NMR (282 MHz, $CDCl_3$) δ -119.4 ppm. ^{11}B NMR (96 MHz, $CDCl_3$) δ 34.6 ppm. **HRMS** (ESI): Exact mass calculated for $C_{22}H_{35}B_2FNaO_4^+$ ([M+Na]$^+$): 427.2598, mass found: 427.2598. **FTIR** (neat): υ (cm^{-1}) 2979, 2934, 1508, 1467, 1372, 1316, 1273, 1223, 1164, 1144, 968, 848, 833.

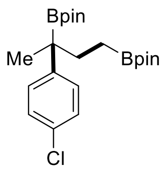

2,2'-(3-(4-Chlorophenyl)butane-1,3-diyl)bis(4,4,5,5-tetramethyl-1,3,2-dioxaborolane) (3m): According to the General Procedure B, **3m** (57.6 mg, 68%) was prepared as a white solid, m.p. = 83-85 °C. ^{1}H NMR (300 MHz, $CDCl_3$) δ 7.26 – 7.17 (m, 4H), 1.91 – 1.72 (m, 2H), 1.29 (s, 3H), 1.23 – 1.17 (m, 24H), 0.74 – 0.52 (m, 2H). ^{13}C NMR (75 MHz, $CDCl_3$) δ 145.6, 130.6, 128.6, 128.0, 83.3, 82.8, 33.2, 24.8, 24.6, 24.6, 20.6 ppm, *carbons attached to borons not observed.* ^{11}B NMR (96 MHz, $CDCl_3$) δ 34.2 ppm. **HRMS** (ESI): Exact mass calculated for $C_{22}H_{35}B_2ClNaO_4^+$ ([M+Na]$^+$): 443.2302, mass found: 443.2304. **FTIR** (neat): υ (cm^{-1}) 2977, 2923, 1492, 1464, 1372, 1315, 1269, 1212, 1145, 968.

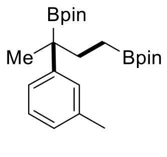

2,2'-(3-(*m*-Tolyl)butane-1,3-diyl)bis(4,4,5,5-tetramethyl-1,3,2-dioxaborolane) (3n):

According to the General Procedure B, **3n** (68.3 mg, 85%) was prepared as a white solid, m.p. = 65-67 °C. ^{1}H NMR (300 MHz, $CDCl_3$) δ 7.19 – 7.07 (m, 3H), 6.97 – 6.88 (m, 1H), 2.31 (s, 3H), 1.95 – 1.71 (m, 2H), 1.31 (s, 3H), 1.26 – 1.18 (m, 24H), 0.78 – 0.58 (m, 2H). ^{13}C NMR (75 MHz, $CDCl_3$) δ 147.1, 137.1, 127.8, 127.7, 125.7, 124.2, 83.1, 82.8, 33.3, 24.8, 24.6, 24.6, 21.6, 20.8 ppm, *carbons attached to borons not observed.* ^{11}B NMR (96 MHz, $CDCl_3$) δ 34.0 ppm. **HRMS** (ESI):

Exact mass calculated for $C_{23}H_{38}B_2NaO_4^+$ ([M+Na]$^+$): 423.2848, mass found: 423.2847. **FTIR** (neat): υ (cm^{-1}) 2978, 2931, 1467, 1371, 1349, 1308, 1272, 1144, 968, 856.

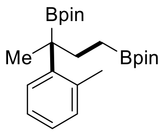

2,2'-(3-(*o*-Tolyl)butane-1,3-diyl)bis(4,4,5,5-tetramethyl-1,3,2-dioxaborolane) (3o):

According to the General Procedure B, **3o** (51.1 mg, 64%) was prepared as a white solid, m.p. = 106-108 °C. ^{1}H NMR (300 MHz, $CDCl_3$) δ 7.22 – 7.18 (m, 1H), 7.16 – 6.99 (m, 3H), 2.33 (s, 3H), 2.00 – 1.75 (m, 2H), 1.32 (s, 3H), 1.26 – 1.16 (m, 24H), 0.70 (ddd, *J* = 17.1, 12.4, 4.8 Hz, 1H), 0.31 (ddd, *J* = 15.9, 12.4, 5.2 Hz, 1H). ^{13}C NMR (75 MHz, $CDCl_3$) δ 144.3, 136.0, 130.7, 126.7, 125.5, 125.1, 83.1, 82.7, 30.2, 24.9, 24.8, 24.8, 24.8, 21.4, 21.2 ppm, *carbons attached to borons not observed*. ^{11}B NMR (96 MHz, $CDCl_3$) δ 34.3 ppm. **HRMS** (ESI): Exact mass calculated for $C_{23}H_{38}B_2NaO_4^+$ ([M+Na]$^+$): 423.2848, mass found: 423.2847. **FTIR** (neat): υ (cm^{-1}) 2979, 2931, 1469, 1371, 1346, 1310, 1271, 1145, 966, 848.

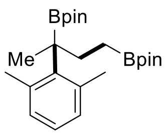

2,2'-(3-(2,6-Dimethylphenyl)butane-1,3-diyl)bis(4,4,5,5-tetramethyl-1,3,2-dioxaborolane) (3p):

According to the General Procedure B, **3p** (54.8 mg, 66%) was prepared as a white solid, m.p. = 106-108 °C. ^{1}H NMR (300 MHz, $CDCl_3$) δ 7.01 – 6.88 (m, 3H), 2.44 (s, 6H), 2.00 – 1.83 (m, 2H), 1.55 (s, 3H), 1.28 (s, 12H), 1.23 (s, 12H), 1.02 – 0.88 (m, 1H), 0.76 – 0.57 (m, 1H). ^{13}C NMR (75 MHz, $CDCl_3$) δ 142.1, 137.6, 130.1, 125.0, 82.9, 82.7, 31.7, 25.1, 24.9, 24.8, 23.9, 23.1 ppm. ^{11}B NMR (96 MHz, $CDCl_3$) δ 34.7 ppm. **HRMS** (ESI): Exact mass calculated for $C_{24}H_{40}B_2NaO_4^+$ ([M+Na]$^+$): 437.3005, mass found: 437.3005. **FTIR** (neat): υ (cm^{-1}) 2979, 2930, 1469, 1370, 1298, 1140, 966, 846, 766.

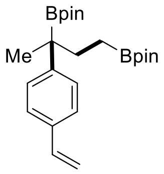

2,2'-(3-(4-Vinylphenyl)butane-1,3-diyl)bis(4,4,5,5-tetramethyl-1,3,2-dioxaborolane) (3q): According to the General Procedure B, **3q** (59.6 mg, 72%) was prepared as a white solid, m.p. = 80-82 °C. ^{1}H NMR (300 MHz, $CDCl_3$) δ 7.35 – 7.24 (m, 4H), 6.68 (dd, J = 17.6, 10.9 Hz, 1H), 5.68 (dd, J = 17.6, 1.0 Hz, 1H), 5.15 (dd, J = 10.9, 1.0 Hz, 1H), 1.95 – 1.74 (m, 2H), 1.31 (s, 3H), 1.25 – 1.18 (m, 24H), 0.76 – 0.56 (m, 2H). ^{13}C NMR (75 MHz, $CDCl_3$) δ 147.0, 136.9, 134.3, 127.2, 125.8, 112.4, 83.2, 82.8, 33.2, 24.8, 24.6, 24.6, 20.7 ppm, *carbons attached to borons not observed.* ^{11}B NMR (96 MHz, $CDCl_3$) δ 34.5 ppm. **HRMS** (ESI): Exact mass calculated for $C_{24}H_{38}B_2NaO_4^+$ ([M+Na]$^+$): 435.2848, mass found: 435.2849. **FTIR** (neat): υ (cm^{-1}) 2978, 2932, 1510, 1461, 1370, 1348, 1308, 1271, 1214, 1142, 967, 846.

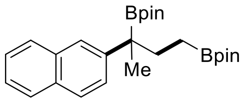

2,2'-(3-(Naphthalen-2-yl)butane-1,3-diyl)bis(4,4,5,5-tetramethyl-1,3,2-dioxaborolane) (3r): According to the General Procedure C, **3r** (59.1 mg, 68%) was prepared as a white solid, m.p. = 109-111 °C. ^{1}H NMR (300 MHz, $CDCl_3$) δ 7.85 – 7.68 (m, 4H), 7.51 (dd, J = 8.6, 1.9 Hz, 1H), 7.47 – 7.32 (m, 2H), 2.08 – 1.87 (m, 2H), 1.44 (s, 3H), 1.28 – 1.18 (m, 24H), 0.82 – 0.57 (m, 2H). ^{13}C NMR (75 MHz, $CDCl_3$) δ 144.7, 133.7, 131.6, 127.8, 127.2, 127.1, 126.7, 125.4, 124.8, 83.3, 82.8, 33.0, 24.8, 24.6, 24.6, 20.6 ppm, *carbons attached to borons not observed.* ^{11}B NMR (96 MHz, $CDCl_3$) δ 34.8 ppm. **HRMS** (ESI): Exact mass calculated for $C_{26}H_{38}B_2NaO_4^+$ ([M+Na]$^+$): 459.2848, mass found: 459.2850. **FTIR** (neat): υ (cm^{-1}) 2978, 2936, 1469, 1371, 1312, 1271, 1144, 967, 859, 749.

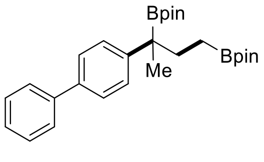

2,2'-(3-([1,1'-Biphenyl]-4-yl)butane-1,3-diyl)bis(4,4,5,5-tetramethyl-1,3,2-dioxaborolane) (3s): According to the General Procedure C, **3r** (64.5 mg, 70%) was prepared as a white solid, m.p. =

117-119 °C. ^{1}H NMR (300 MHz, $CDCl_3$) δ 7.62 – 7.56 (m, 2H), 7.54 – 7.47 (m, 2H), 7.45 – 7.36 (m, 4H), 7.35 – 7.27 (m, 1H), 2.00 – 1.78 (m, 2H), 1.37 (s, 3H), 1.27 – 1.20 (m, 24H), 0.82 – 0.61 (m, 2H). ^{13}C NMR (75 MHz, $CDCl_3$) δ 146.3, 141.3, 137.6, 128.6, 127.5, 126.9, 126.7, 126.6, 83.3, 82.8, 33.3, 24.8, 24.6, 24.6, 20.8 ppm, *carbons attached to borons not observed.* ^{11}B NMR (96 MHz, $CDCl_3$) δ 35.1 ppm. **HRMS** (ESI): Exact mass calculated for $C_{28}H_{40}B_2NaO_4^+$ ([M+Na]$^+$): 485.3005, mass found: 485.3007. **FTIR** (neat): υ (cm^{-1}) 2977, 2933, 1488, 1469, 1371, 1311, 1271, 1144, 968, 847, 767, 737, 700.

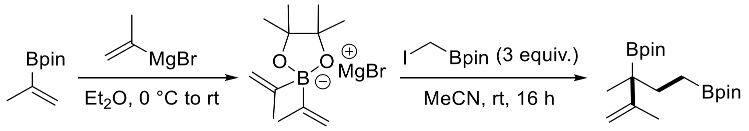

3t

2,2'-(3,4-Dimethylpent-4-ene-1,3-diyl)bis(4,4,5,5-tetramethyl-1,3,2-dioxaborolane) (3t): Isopropenylboronic acid pinacol ester **1a** (0.20 mmol, 1.0 equiv.) was dissolved in diethyl ether (2.0 mL) and the Grignard reagent isopropenylmagnesium bromide solution (0.22 mmol, 0.5 M in THF, 1.1 equiv.) was added dropwise over 5 minutes at 0 °C. The solution was then stirred for 0.5 h at 0 °C, warmed to room temperature and stirred for a further 0.5 h. Subsequently, the solvent was carefully removed in vacuo and the resulting residue was taken up in acetonitrile (2.0 mL). After the addition of ICH_2Bpin (0.60 mmol, 3.0 equiv.), the tube was sealed and the mixture was stirred at room temperature for 16 h. The reaction mixture was filtered through a pad of silica and rinsed with 30 mL Et_2O. Flash column chromatography eluting with pentane and Et_2O (pentane/Et_2O = 10:1) afforded the desired product. *Caution: In order to get a good yield, the chromatography should be finished within 10 min.*

3t (32.1 mg, 46%) was prepared as a white solid, m.p. = 44-46 °C. ^{1}H NMR (300 MHz, $CDCl_3$) δ 4.80 (s, 1H), 4.67 (s, 1H), 1.72 (s, 3H), 1.68 – 1.60 (m, 2H), 1.24 – 1.20 (m, 24H), 1.04 (s, 3H), 0.72 – 0.53 (m, 2H). ^{13}C NMR (75 MHz, $CDCl_3$) δ 149.6, 109.7, 83.1, 82.8, 29.1, 24.8, 24.7, 24.6, 21.7, 19.3 ppm, *carbons attached to borons not observed.* ^{11}B NMR (96 MHz, $CDCl_3$) δ 34.1 ppm. **HRMS** (ESI): Exact mass calculated for $C_{19}H_{36}B_2NaO_4^+$ ([M+Na]$^+$): 373.2692, mass found: 373.2693. **FTIR** (neat): υ (cm^{-1}) 2978, 2928, 1457, 1371, 1345, 1308, 1272, 1144, 1098, 968, 880, 848.

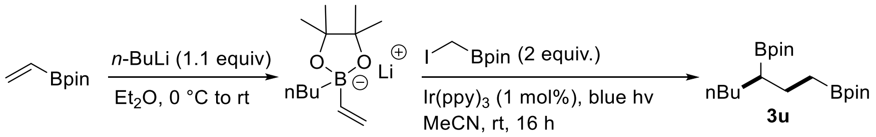

2,2'-(Heptane-1,3-diyl)bis(4,4,5,5-tetramethyl-1,3,2-dioxaborolane) (3u):

Vinyl boronic ester **1b** (0.30 mmol, 1.0 equiv.) was dissolved in diethyl ether (2.0 mL) and a solution of *n*-butyllithium (1.6 M, 0.33 mmol, 1.1 equiv.) was added dropwise over 5 minutes at 0 °C. The solution was then stirred for 0.5 h at 0 °C, warmed to room temperature and stirred for a further 0.5 h. Subsequently, the solvent was carefully removed in vacuo. After addition of $Ir(ppy)_3$ (2.0 mg, 1 mol%) and acetonitrile (1 mL), the mixture was stirred for 1 min until all solid was dissolved. Then ICH_2Bpin (0.60 mmol, 2.0 equiv.) was added to the reaction mixture and the reaction mixture was irradiated with a 30 W blue LED (465 nm) and stirred at room temperature for 16 h. The reaction mixture was filtered through a pad of silica and rinsed with 30 mL Et_2O. Flash column chromatography eluting with pentane and Et_2O (pentane/Et_2O = 10:1) afforded the desired product.

Caution: In order to get a good yield, the chromatography should be finished within 10 min

3u (61.0 mg, 58%) was prepared as a colorless sticky oil. ^{1}H NMR (300 MHz, $CDCl_3$) δ 1.52 – 1.41 (m, 2H), 1.38 – 1.30 (m, 2H), 1.26 – 1.19 (m, 28H), 0.97 – 0.88 (m, 1H), 0.87 – 0.82 (m, 3H), 0.80 – 0.65 (m, 2H). ^{13}C NMR (75 MHz, $CDCl_3$) δ 82.7, 82.7, 31.4, 30.8, 25.4, 24.8, 24.8, 23.0, 14.0 ppm, *carbons attached to borons not observed.* ^{11}B NMR (96 MHz, $CDCl_3$) δ 34.2 ppm. **HRMS** (ESI): Exact mass calculated for $C_{19}H_{38}B_2NaO_4^+$ ([M+Na]$^+$): 375.2848, mass found: 375.2849. **FTIR** (neat): υ (cm^{-1}) 2979, 2924, 2859, 1457, 1371, 1314, 1272, 1216, 1145, 969, 867.

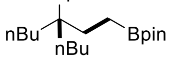

2,2'-(3-Butylheptane-1,3-diyl)bis(4,4,5,5-tetramethyl-1,3,2-dioxaborolane) (3v):

According to the General Procedure B, **3v** (65.0 mg, 80%) was prepared as a colorless sticky oil. ^{1}H NMR (300 MHz, $CDCl_3$) δ 1.48 – 1.41 (m, 2H), 1.34 – 1.26 (m, 8H), 1.24 – 1.08 (m, 28H), 0.87 (t, J = 7.1 Hz, 6H), 0.66 – 0.58 (m, 2H). ^{13}C NMR (75 MHz, $CDCl_3$) δ 82.7, 33.6, 27.5, 27.0, 24.9, 24.8, 23.7, 14.2 ppm, *carbons attached to borons not observed.* ^{11}B NMR (96 MHz, $CDCl_3$) δ 35.2 ppm. **HRMS** (ESI): Exact mass calculated for $C_{23}H_{46}B_2NaO_4^+$ ([M+Na]$^+$): 431.3474, mass found: 431.3474. **FTIR** (neat): υ (cm^{-1}) 2979, 2957, 2928, 2859, 1460, 1371, 1306, 1215, 1144, 968, 855.

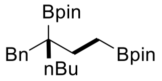

2,2'-(3-Benzylheptane-1,3-diyl)bis(4,4,5,5-tetramethyl-1,3,2-dioxaborolane) (3w):

According to the General Procedure B, **3w** (44.1 mg, 50%) was prepared as a yellow sticky oil. ^{1}H NMR (300 MHz, $CDCl_3$) δ 7.22 – 7.09 (m, 5H), 2.68 (s, 2H), 1.51 – 1.42 (m, 2H), 1.30 – 1.18 (m, 30H), 0.89 (t, *J* = 6.2 Hz, 3H), 0.81 – 0.75 (m, 2H). ^{13}C NMR (75 MHz, $CDCl_3$) δ 140.3, 130.3, 127.6, 125.4, 83.0, 82.8, 39.5, 33.6, 29.7, 27.6, 27.1, 25.1, 25.1, 24.8, 24.8, 23.6, 14.2 ppm, *carbons attached to borons not observed.* ^{11}B NMR (96 MHz, $CDCl_3$) δ 34.3 ppm. **HRMS** (ESI): Exact mass calculated for $C_{26}H_{44}B_2NaO_4^+$ ([M+Na]$^+$): 465.3318, mass found: 465.3320. **FTIR** (neat): υ (cm^{-1}) 2978, 2957, 2927, 2857, 1457, 1371, 1311, 1271, 1144, 967, 849, 701.

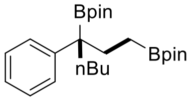

2,2'-(3-Phenylheptane-1,3-diyl)bis(4,4,5,5-tetramethyl-1,3,2-dioxaborolane) (3x):

According to the General Procedure B, **3x** (23.0 mg, 27%) was prepared as a white solid, m.p. = 88-90 °C. ^{1}H NMR (300 MHz, $CDCl_3$) δ 7.31 – 7.21 (m, 4H), 7.13 – 7.06 (m, 1H), 1.93 – 1.86 (m, 2H), 1.83 – 1.74 (m, 2H), 1.29 – 1.26 (m, 2H), 1.24 – 1.19 (m, 24H), 1.15 – 1.07 (m, 2H), 0.85 (t, *J* = 7.3 Hz, 3H), 0.60 – 0.50 (m, 2H). ^{13}C NMR (75 MHz, $CDCl_3$) δ 145.8, 127.8, 124.8, 83.1, 82.7, 33.8, 28.8, 27.5, 24.8, 24.8, 24.7, 24.7, 23.5, 14.1 ppm, *carbons attached to borons not observed.* ^{11}B NMR (96 MHz, $CDCl_3$) δ 34.3 ppm. **HRMS** (ESI): Exact mass calculated for $C_{25}H_{42}B_2NaO_4^+$ ([M+Na]$^+$): 451.3161, mass found: 451.3161. **FTIR** (neat): υ (cm^{-1}) 2978, 2932, 2860, 1467, 1371, 1314, 1272, 1214, 1144, 968, 849, 701.

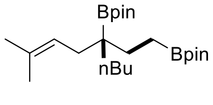

2,2'-(3-Butyl-6-methylhept-5-ene-1,3-diyl)bis(4,4,5,5-tetramethyl-1,3,2-dioxaborolane) (3y):

According to the General Procedure B, **3y** (57.1 mg, 68%) was prepared as a colorless sticky oil. ^{1}H NMR (300 MHz, $CDCl_3$) δ 5.17 – 5.06 (m, 1H), 2.01 (d, *J* = 7.3 Hz, 2H), 1.65 (s, 3H), 1.59 (s, 3H), 1.50 – 1.42 (m, 2H), 1.33 – 1.18 (m, 30H), 0.86 (t, *J* = 7.1 Hz, 3H), 0.70 – 0.60 (m, 2H). ^{13}C NMR (75 MHz, $CDCl_3$) δ 131.4, 122.2, 82.8, 82.7, 33.6, 32.4, 27.7, 27.1, 26.0, 24.9, 24.8, 23.7,

18.0, 14.2 ppm, *carbons attached to borons not observed.* ^{11}B NMR (96 MHz, $CDCl_3$) δ 35.6 ppm. **HRMS** (ESI): Exact mass calculated for $C_{24}H_{46}B_2NaO_4^+$ ($[M+Na]^+$): 443.3474, mass found: 443.3476. **FTIR** (neat): υ (cm^{-1}) 2977, 2925, 2860, 1457, 1371, 1309, 1272, 1215, 1146, 967, 857.

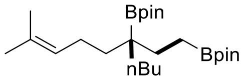

2,2'-(3-Butyl-7-methyloct-6-ene-1,3-diyl)bis(4,4,5,5-tetramethyl-1,3,2-dioxaborolane) (3z): According to the General Procedure B, **3z** (69.3 mg, 80%) was prepared as a colorless sticky oil. ^{1}H NMR (300 MHz, $CDCl_3$) δ 5.10 (t, J = 7.1 Hz, 1H), 1.90 – 1.79 (m, 2H), 1.65 (s, 3H), 1.58 (s, 3H), 1.51 – 1.42 (m, 2H), 1.35 – 1.12 (m, 32H), 0.86 (t, J = 7.1 Hz, 3H), 0.71 – 0.57 (m, 2H). ^{13}C NMR (75 MHz, $CDCl_3$) δ 130.4, 125.6, 82.7, 82.7, 33.9, 33.6, 27.5, 27.0, 25.7, 24.9, 24.8, 23.6, 23.6, 17.5, 14.2 ppm, *carbons attached to borons not observed.* ^{11}B NMR (96 MHz, $CDCl_3$) δ 34.7 ppm. **HRMS** (ESI): Exact mass calculated for $C_{25}H_{48}B_2NaO_4^+$ ($[M+Na]^+$): 457.3631, mass found: 457.3633. **FTIR** (neat): υ (cm^{-1}) 2978, 2927, 2857, 1457, 1371, 1308, 1215, 1145, 967, 856.

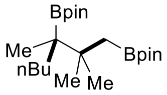

2,2'-(2,2,3-Trimethylheptane-1,3-diyl)bis(4,4,5,5-tetramethyl-1,3,2-dioxaborolane) (3aa): According to the General Procedure A, **3aa** (27.0 mg, 34%) was prepared as a colorless sticky oil. ^{1}H NMR (300 MHz, $CDCl_3$) δ 1.65 – 1.54 (m, 2H), 1.24 – 1.22 (m, 24H), 1.04 – 0.98 (m, 9H), 0.90 – 0.83 (m, 9H). ^{13}C NMR (75 MHz, $CDCl_3$) δ 82.8, 82.6, 36.6, 33.4, 29.6, 26.4, 25.9, 25.1, 25.0, 24.9, 24.8, 24.0, 16.9, 14.2 ppm, *carbons attached to borons not observed.* ^{11}B NMR (96 MHz, $CDCl_3$) δ 35.2 ppm. **HRMS** (ESI): Exact mass calculated for $C_{22}H_{44}B_2NaO_4^+$ ($[M+Na]^+$): 417.3318, mass found: 417.3317. **FTIR** (neat): υ (cm^{-1}) 2978, 2933, 2875, 1467, 1371, 1347, 1321, 1298, 1214, 1142, 1111, 969, 854.

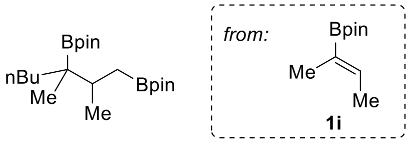

2,2'-(2,3-Dimethylheptane-1,3-diyl)bis(4,4,5,5-tetramethyl-1,3,2-dioxaborolane) (3ab): According to the General Procedure A, **3ab** (69.2 mg, 91%, dr = 1.7:1) was prepared as a colorless

sticky oil. ^{1}H NMR (300 MHz, $CDCl_3$) δ 1.91 – 1.72 (m, 1H), 1.54 – 1.41 (m, 1H), 1.40 – 1.06 (m, 29H), 1.06 – 0.75 (m, 10H), 0.71 – 0.40 (m, 1H). ^{13}C NMR (75 MHz, $CDCl_3$) δ 82.7, 82.7, 82.6, 37.6, 36.8, 35.4, 35.2, 28.6, 28.5, 25.0, 25.0, 24.9, 24.9, 24.9, 24.7, 24.7, 23.8, 23.8, 19.6, 17.2, 16.8, 16.3, 14.1 ppm, *carbons attached to borons not observed.* ^{11}B NMR (96 MHz, $CDCl_3$) δ 35.0 ppm. **HRMS** (ESI): Exact mass calculated for $C_{21}H_{42}B_2NaO_4^+$ ([M+Na]$^+$): 403.3161, mass found: 403.3167. **FTIR** (neat): υ (cm^{-1}) 2981, 2933, 2874, 1468, 1370, 1300, 1213, 1140, 967, 848.

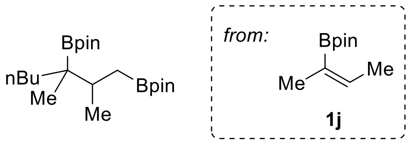

2,2'-(2,3-Dimethylheptane-1,3-diyl)bis(4,4,5,5-tetramethyl-1,3,2-dioxaborolane) (3ab): According to the General Procedure A, **3ab** (68.7 mg, 90%, dr = 1.3:1) was prepared as a colorless sticky oil. ^{1}H NMR (300 MHz, $CDCl_3$) δ 1.93 – 1.69 (m, 1H), 1.48 – 1.41 (m, 1H), 1.40 – 1.07 (m, 29H), 1.05 – 0.74 (m, 10H), 0.69 – 0.41 (m, 1H). ^{13}C NMR (75 MHz, $CDCl_3$) δ 82.7, 82.7, 82.7, 82.6, 37.5, 36.7, 35.4, 35.2, 28.6, 28.4, 25.0, 24.9, 24.9, 24.9, 24.9, 24.7, 24.6, 23.8, 23.8, 19.6, 17.2, 16.8, 16.3, 14.1 ppm, *carbons attached to borons not observed.* ^{11}B NMR (96 MHz, $CDCl_3$) δ 34.9 ppm. **FTIR** (neat): υ (cm^{-1}) 2979, 2932, 2871, 1464, 1370, 1300, 1213, 1141, 967, 848.

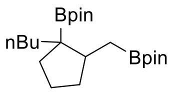

2-(1-Butyl-2-((4,4,5,5-tetramethyl-1,3,2-dioxaborolan-2-yl)methyl)cyclopentyl)-4,4,5,5-tetramethyl-1,3,2-dioxaborolane (3ac): According to the General Procedure A, **3ac** (46.1 mg, 59%, dr = 2.2:1)) was prepared as a white solid, m.p. = 36-38 °C. ^{1}H NMR (300 MHz, $CDCl_3$) 1H NMR (300 MHz, CDCl3) δ 2.13 – 1.77 (m, 2H), 1.76 – 1.63 (m, 2H), 1.61 – 1.50 (m, 2H), 1.48 – 1.38 (m, 1H), 1.23 – 1.19 (m, 24H), 1.16 – 1.08 (m, 2H), 0.97 – 0.90 (m, 1H), 0.86 (t, J = 7.0 Hz, 3H), 0.68 – 0.57 (m, 1H). ^{13}C NMR (75 MHz, $CDCl_3$) δ13C NMR (75 MHz, CDCl3) δ 82.7, 82.6, 82.6, 46.7, 41.4, 38.3, 34.4, 33.9, 32.4, 32.2, 30.6, 30.00, 29.8, 25.2, 24.9, 24.8, 24.8, 24.7, 24.6, 23.8, 22.8, 22.6, 14.1 ppm, *carbons attached to borons not observed.* ^{11}B NMR (96 MHz, $CDCl_3$) δ 34.9 ppm. **HRMS** (ESI): Exact mass calculated for $C_{22}H_{42}B_2NaO_4^+$ ([M+Na]$^+$): 415.3161, mass found: 415.3160. **FTIR** (neat): υ (cm^{-1}) 2978, 2953, 2928, 2863, 1371, 1314, 1296, 1215, 1143, 969, 849.

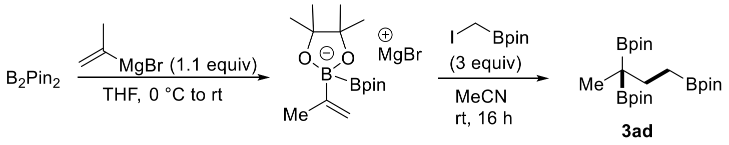

2,2',2''-(Butane-1,3,3-triyl)tris(4,4,5,5-tetramethyl-1,3,2-dioxaborolane) (3ad): Bis(pinacolato)diboron (B_2Pin_2) (0.20 mmol, 1.0 equiv.) was dissolved in THF (2.0 mL) and the Grignard reagent isopropenylmagnesium bromide solution (0.22 mmol, 0.5 M in THF, 1.1 equiv.) was added dropwise over 5 minutes at 0 °C. The solution was then stirred for 0.5 h at 0 °C, warmed to room temperature and stirred for a further 0.5 h. Subsequently, the solvent was carefully removed in vacuo and the resulting residue was taken up in acetonitrile (2.0 mL). After the addition of ICH_2Bpin (0.60 mmol, 3.0 equiv.), the tube was sealed and the mixture was stirred at room temperature for 16 h. The reaction mixture was filtered through a pad of silica and rinsed with 30 mL Et_2O. Flash column chromatography eluting with pentane and Et_2O (pentane/Et_2O = 10:1 to 5:1) afforded the desired product.

3ad (14.2 mg, 16%) was prepared as a colorless sticky oil. ^1H NMR (400 MHz, $CDCl_3$) δ 1.61 – 1.57 (m, 2H), 1.16 (s, 12H), 1.14 (s, 24H), 0.97 (s, 3H), 0.71 – 0.67 (m, 2H). ^{13}C NMR (100 MHz, $CDCl_3$) δ 82.8, 82.7, 27.7, 24.8, 24.7, 24.7, 15.4 ppm, *carbons attached to borons not observed.* ^{11}B NMR (128 MHz, $CDCl_3$) δ 33.7 ppm. **HRMS** (ESI): Exact mass calculated for $C_{22}H_{43}B_3NaO_6^+$ ($[M+Na]^+$): 459.3231, mass found: 459.3238. **FTIR** (neat): υ (cm^{-1}) 2978, 2930, 1371, 1343, 1299, 1266, 1215, 1141, 1081, 968, 848.

4. Mechanistic Studies

4.1 Control Experiments

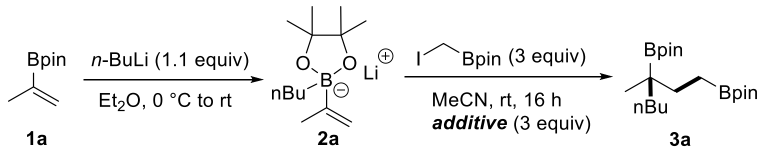

According to the General Procedure A, isopropenylboronic acid pinacol ester **1a** (0.20 mmol, 1.0 equiv.) was dissolved in diethyl ether (2.0 mL) and *n*-butyllithium (1.6 M, 0.22 mmol, 1.1 equiv.) was added dropwise over 5 minutes at 0 °C. The solution was then stirred for 0.5 h at 0 °C, warmed

to room temperature and stirred for a further 0.5 h. Subsequently, the solvent was carefully removed *in vacuo* and the resulting residue was taken up in acetonitrile (2.0 mL). After the addition of 2,2-6,6-tetramethyl piperidine-*N*-oxyl (TEMPO, 0.60 mmol, 3 equiv.) or 3,5-di-tert-4-butylhydroxytoluene (BHT, 0.60 mmol, 3 equiv.), ICH_2Bpin (0.60 mmol, 3.0 equiv.) was added, and the tube was sealed and the mixture was stirred at room temperature for 16 h. The yield of **3a** was determined by GC with n-$C_{14}H_{30}$ as an internal standard. 86% yield was obtained in the presence of TEMPO, and 92% yield was obtained in the presence of BHT. Radical trapping products were not identified.

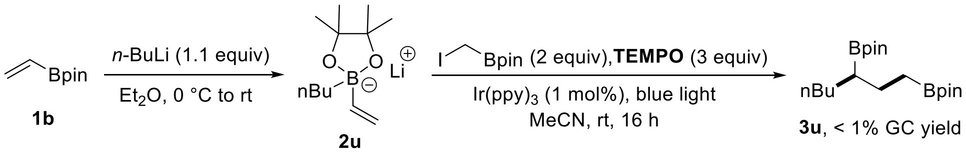

Vinyl boronic ester **1b** (0.30 mmol, 1.0 equiv.) was dissolved in diethyl ether (2.0 mL) and a solution of *n*-butyllithium (1.6 M, 0.33 mmol, 1.1 equiv.) was added dropwise over 5 minutes at 0 °C. The solution was then stirred for 0.5 h at 0 °C, warmed to room temperature and stirred for a further 0.5 h. Subsequently, the solvent was carefully removed *in vacuo*. After addition of $Ir(ppy)_3$ (2.0 mg, 1 mol%) and acetonitrile (1 mL), the mixture was stirred for 1 min until all solid was dissolved. Then 2,2,6,6-tetramethyl piperidine-*N*-oxyl (TEMPO, 0.60 mmol, 2 equiv.) and ICH_2Bpin (0.60 mmol, 2.0 equiv.) were added to the reaction mixture and the reaction mixture was irradiated with a 30 W blue LED (465 nm) and stirred at room temperature for 16 h. The yield of **3a** was determined by GC with n-$C_{14}H_{30}$ as an internal standard. The reaction was suppressed upon addition of TEMPO, and only less than 1% yield of **3a** was obtained.

4.2 Radical Probe Experiments

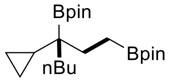

2,2'-(3-Cyclopropylheptane-1,3-diyl)bis(4,4,5,5-tetramethyl-1,3,2-dioxaborolane) (3ae): According to the General Procedure A, **3ae** (68.2 mg, 87%) was obtained as a colorless sticky oil.

^{1}H NMR (300 MHz, $CDCl_3$) δ 1.61 – 1.42 (m, 2H), 1.29 – 1.14 (m, 30H), 0.91 – 0.78 (m, 5H), 0.54 – 0.45 (m, 1H), 0.35 – 0.22 (m, 4H). ^{13}C NMR (75 MHz, $CDCl_3$) δ 82.7, 82.6, 35.8, 30.1, 27.7, 24.9, 24.8, 23.8, 17.8, 14.2, 2.2, 1.9 ppm, *carbons attached to borons not observed.* ^{11}B NMR (96 MHz, $CDCl_3$) δ 37.9 ppm. **HRMS** (ESI): Exact mass calculated for $C_{22}H_{42}B_2NaO_4^+$ ($[M+Na]^+$): 415.3161, mass found: 415.3160. **FTIR** (neat): υ (cm^{-1}) 2979, 2932, 2859, 1467, 1371, 1305, 1272, 1215, 1144, 968, 865, 849.

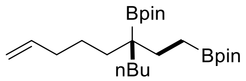

2,2'-(3-Butyloct-7-ene-1,3-diyl)bis(4,4,5,5-tetramethyl-1,3,2-dioxaborolane) (3af):

According to the General Procedure A, **3af** (70.8 mg, 84%) was obtained as a colorless sticky oil. ^{1}H NMR (300 MHz, $CDCl_3$) δ 5.90 – 5.76 (m, 1H), 5.08 – 4.87 (m, 2H), 2.02 (q, J = 6.6 Hz, 2H), 1.52 – 1.44 (m, 2H), 1.35 – 1.14 (m, 34H), 0.89 (t, J = 7.1 Hz, 3H), 0.71 – 0.59 (m, 2H). ^{13}C NMR (75 MHz, $CDCl_3$) δ 139.4, 113.9, 82.7, 82.7, 34.7, 33.6, 33.5, 27.5, 27.0, 24.9, 24.8, 24.2, 23.6, 14.1 ppm, *carbons attached to borons not observed.* ^{11}B NMR (96 MHz, $CDCl_3$) δ 35.0 ppm. **HRMS** (ESI): Exact mass calculated for $C_{24}H_{46}B_2NaO_4^+$ ($[M+Na]^+$): 443.3474, mass found: 443.3476. **FTIR** (neat): υ (cm^{-1}) 2979, 2931, 2861, 1460, 1371, 1309, 1272, 1214, 1145, 968, 849.

5. Synthetic Transformations

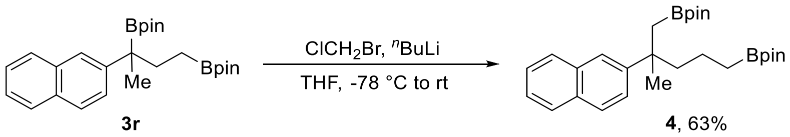

The title compound was prepared according to a literature procedure[11].

A solution of 1,3-bis(boronic ester) **3r** (87.2 mg, 0.2 mmol, 1.0 equiv) and bromochloromethane (155 mg, 78 μL, 1.2 mmol, 6.0 equiv.) was dissolved in anhydrous Et_2O (0.2 M) under an atmosphere of nitrogen. The reaction mixture was cooled to -78 °C. ^{n}BuLi (1.6 M in hexanes, 5 equiv.) was added dropwise to the reaction mixture at -78 °C. The reaction mixture was stirred for 20 min at -78 °C. The reaction mixture was removed from the cooling bath and stirred at room temperature for 1 h. Afterwards, the reaction mixture was diluted with water and extracted with Et_2O (3 × 10 mL). The combined organic phases were washed with brine and dried over $MgSO_4$.

The solvent was removed in vacuo and the crude material purified by flash chromatography (pentane/Et_2O = 15:1) to afford double homologation product **4** (58.6 mg, 63%) as a white solid, m.p. = 90-92 °C.

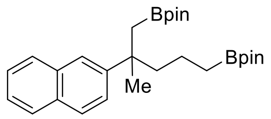

2,2'-(2-Methyl-2-(naphthalen-2-yl)pentane-1,5-diyl)bis(4,4,5,5-tetramethyl-1,3,2-dioxaborolane) (4):

^{1}H NMR (300 MHz, $CDCl_3$) δ 7.82 – 7.69 (m, 4H), 7.54 (dd, J = 8.6, 1.9 Hz, 1H), 7.45 – 7.35 (m, 2H), 1.91 – 1.68 (m, 2H), 1.54 (s, 3H), 1.45 – 1.38 (m, 2H), 1.27 – 1.19 (m, 16H), 1.04 – 1.01 (m, 10H), 0.68 (t, J = 7.7 Hz, 2H). ^{13}C NMR (75 MHz, $CDCl_3$) δ 147.3, 133.3, 131.6, 127.9, 127.2, 125.4, 125.3, 124.8, 124.2, 82.7, 82.6, 48.3, 39.6, 26.7, 24.8, 24.8, 24.7, 24.6, 19.1 ppm, *carbons attached to borons not observed.* ^{11}B NMR (96 MHz, $CDCl_3$) δ 34.1 ppm. **HRMS** (ESI): Exact mass calculated for $C_{28}H_{42}B_2NaO_4^+$ ($[M+Na]^+$): 487.3161, mass found: 487.3162. **FTIR** (neat): υ (cm^{-1}) 2977, 2930, 1468, 1379, 1371, 1356, 1321, 1273, 1145, 968, 849, 815, 747.

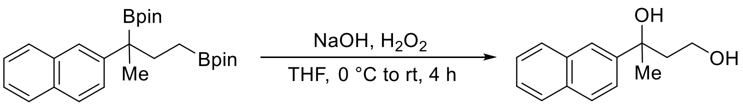

3r **5**, 90%

The title compound was prepared according to a literature procedure[12].

To a solution of **3r** (87.2 mg, 0.2 mmol) in tetrahydrofuran (3 mL) at 0 °C was added aqueous sodium hydroxide solution (3 mL, 9 mmol, 3 M). Aqueous hydrogen peroxide solution (1.5 mL, 30 % w/w) was added dropwise. The mixture was stirred at room temperature for 4 hours. Upon the completion of the reaction as determined by TLC, the mixture was cooled to 0 °C and saturated aqueous sodium thiosulfate solution (6 mL) was added dropwise. The aqueous layer was extracted with ethyl acetate (3 x 15 mL). The combined organic layers were dried over magnesium sulfate, filtered and concentrated. The product was purified by flash column chromatography on silica gel with n-pentane/ethyl acetate (5:1 to 2:1) as eluent to give the corresponding product **5** (38.9 mg, 90%) as a colorless oil.

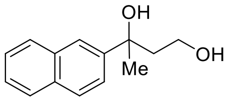

3-(Naphthalen-2-yl)butane-1,3-diol (5):

^{1}H NMR (300 MHz, $CDCl_3$) δ 7.98 (d, J = 1.2 Hz, 1H), 7.88 – 7.79 (m, 3H), 7.52 – 7.43 (m, 3H), 3.80 – 3.74 (m, 1H), 3.61 – 3.52 (m, 1H), 3.06 (br s, 2H), 2.25 – 2.05 (m, 2H), 1.65 (s, 3H). ^{13}C NMR (75 MHz, $CDCl_3$) δ 144.9, 133.2, 132.2, 128.1, 127.9, 127.4, 126.1, 125.7, 123.4, 123.3, 76.0, 60.4, 43.8, 31.0 ppm. **HRMS** (ESI): Exact mass calculated for $C_{14}H_{16}NaO_2^+$ ([M+Na]$^+$): 239.1043, mass found: 239.1041. **FTIR** (neat): υ (cm^{-1}) 3345, 3053, 2970, 2930, 1432, 1375, 1275, 1128, 1099, 1049, 859, 819, 748.

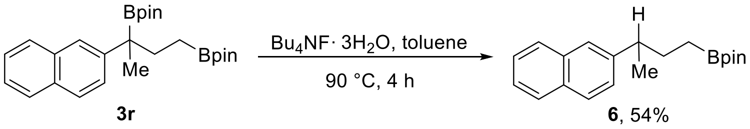

The title compound was prepared according to a literature procedure[13].

1,3-Bis(boronic ester) **3r** (87.2 mg, 0.2 mmol) and tetra-*n*-butylammonium fluoride trihydrate (189 mg, 0.6 mmol) were stirred in toluene at 90 °C for 4 h. Afterwards the mixture was filtered through a short silica column, concentrated in vacuo and the residue subjected to flash chromatography to afford primary boronic ester **6** (33.7 mg, 54%) as a white solid.

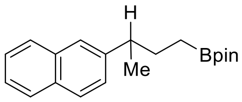

4,4,5,5-Tetramethyl-2-(3-(naphthalen-2-yl)butyl)-1,3,2-dioxaborolane (6):

^{1}H NMR (300 MHz, $CDCl_3$) δ 7.81 – 7.75 (m, 3H), 7.61 (s, 1H), 7.48 – 7.32 (m, 3H), 2.81 (h, J = 7.0 Hz, 1H), 1.85 – 1.71 (m, 2H), 1.34 (d, J = 7.0 Hz, 3H), 1.22 (s, 12H), 0.81 – 0.65 (m, 2H). ^{13}C NMR (75 MHz, $CDCl_3$) δ 145.0, 133.6, 132.2, 127.7, 127.5, 127.5, 126.0, 125.6, 125.3, 124.9, 82.9, 42.3, 32.6, 24.8, 21.6 ppm. ^{11}B NMR (96 MHz, $CDCl_3$) δ 34.7 ppm. **FTIR** (neat): υ (cm^{-1}) 2977, 2923, 1371, 1316, 1145, 967, 849, 817, 746. The spectroscopic data are in accordance to those reported in the literature[14].

6. References

[1] A. Ganić, A. Pfaltz, *Chem. Eur. J.* **2012**, *18*, 6724.

[2] J. R. Vyvyan, J. A. Dell, T. J. Ligon, K. K. Motanic, H. S. Wall, *Synthesis* **2010**, *21*, 3637.

[3] M. Silvi, C. Sandford, V. K. Aggarwal, *J. Am. Chem. Soc.* **2017**, *139*, 5736.

[4] J. L.-Y. Chen, H. K. Scott, M. J. Hesse, C. L. Willis, V. K. Aggarwal, *J. Am. Chem. Soc.* **2013**, *135*, 5316.

[5] R. J. Armstrong, C. Sandford, C. García-Ruiz, V. K. Aggarwal, *Chem. Commun.* **2017**, *53*, 4922.

[6] V. Rauniyar, H. Zhai, D. G. Hall, *Synth. Commun.* **2008**, *38*, 3984.

[7] J. A. Myhill, L. Zhang, G. J. Lovinger, J. P. Morken, *Angew. Chem. Int. Ed.* **2018**, *57*, 12799; *Angew. Chem.* **2018**, *130*, 12981.

[8] a) T. Miura, J. Nakahashi, M. Murakami, *Angew. Chem. Int. Ed.* **2017**, *56*, 6989; *Angew. Chem.* **2017**, *129*, 7093; b) Z.-Q. Zhang, C.-T. Yang, L.-J. Liang, B. Xiao, X. Lu, J.-H. Liu, Y.-Y. Sun, T. B. Marder, Y. Fu, *Org. Lett.* **2014**, *16*, 6342.

[9] H. Li, X. Shangguan, Z. Zhang, S. Huang, Y. Zhang, J. Wang, *Org. Lett.* **2014**, *16*, 448.

[10] Z.-J. Yao, S. Hong, W. Zhang, M. Liu, W. Deng, *Tetrahedron Lett.* **2016**, *57*, 910.

[11] D. J. Blair, D. Tanini, J. M. Bateman, H. K. Scott, E. L. Myers, V. K. Aggarwal. *Chem. Sci.* **2017**, *8*, 2898.

[12] C. Gerleve, M. Kischkewitz, A. Studer, *Angew. Chem. Int. Ed.* **2018**, *57*, 2441; *Angew. Chem.* **2018**, *130*, 2466.

[13] S. Nave, R. P. Sonawane, T. G. Elford, V. K. Aggarwal, *J. Am. Chem. Soc.* **2010**, *132*, 17096.

[14] D. Wang, X.-S. Xue, K. N. Houk, Z. Shi, *Angew. Chem. Int. Ed.* **2018**, *57*, 16861; *Angew. Chem.* **2018**, *130*, 17103.

7. NMR spectra

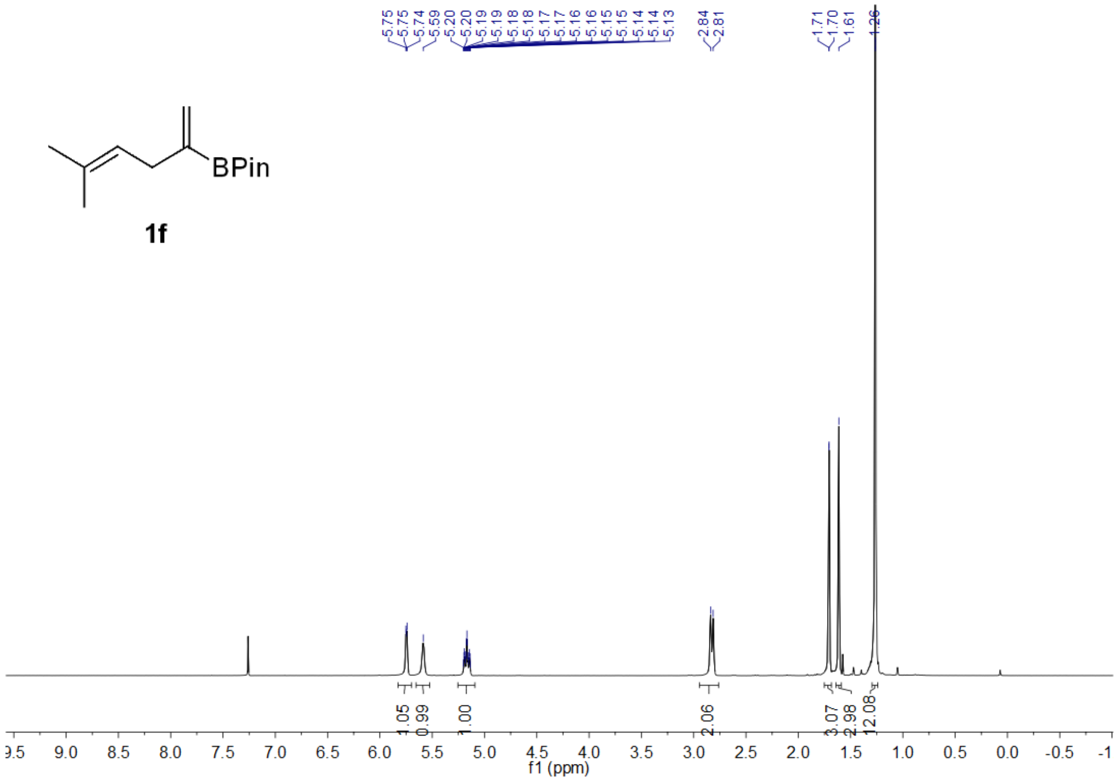

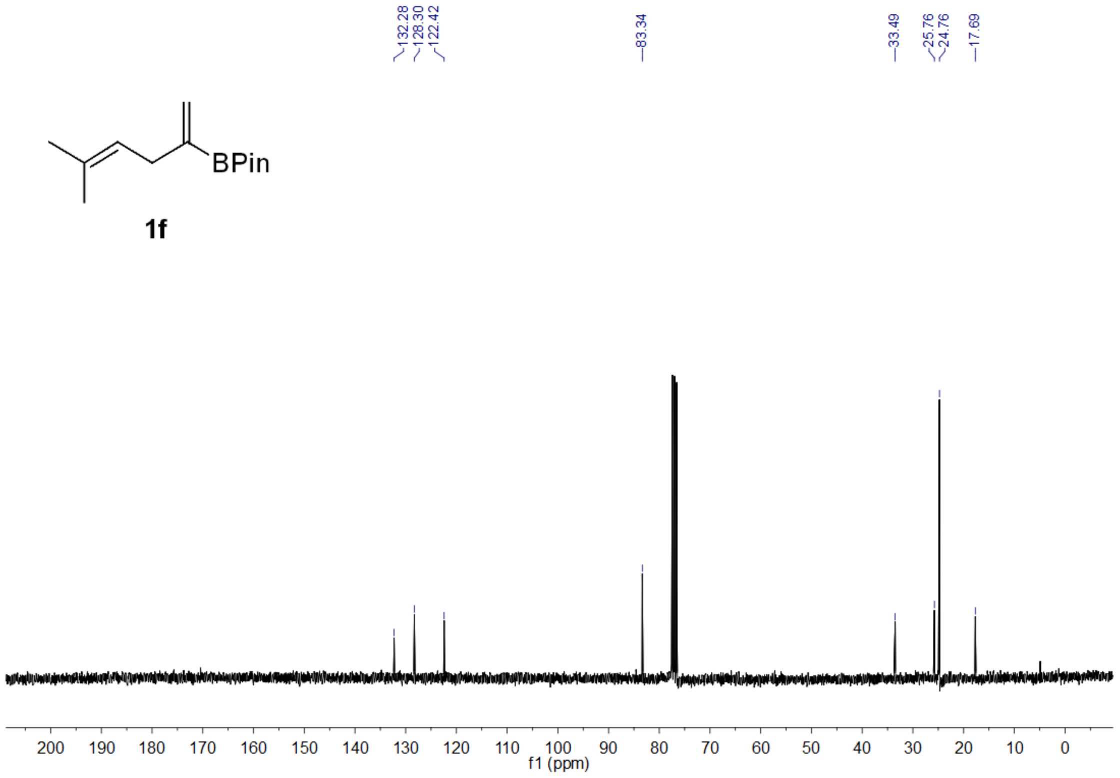

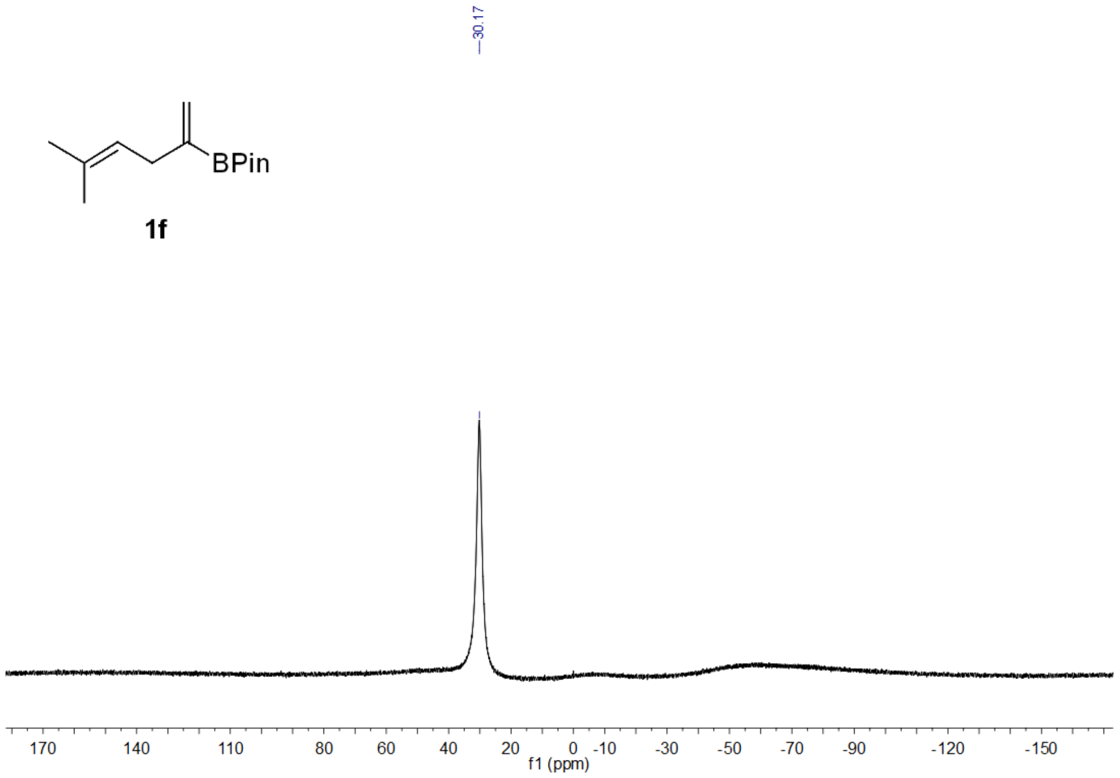

30.17
BPin
1f
170
140
110
80
60
40
20
0
-10
-30
-50
-70
-90
-120
-150
f1 (ppm)

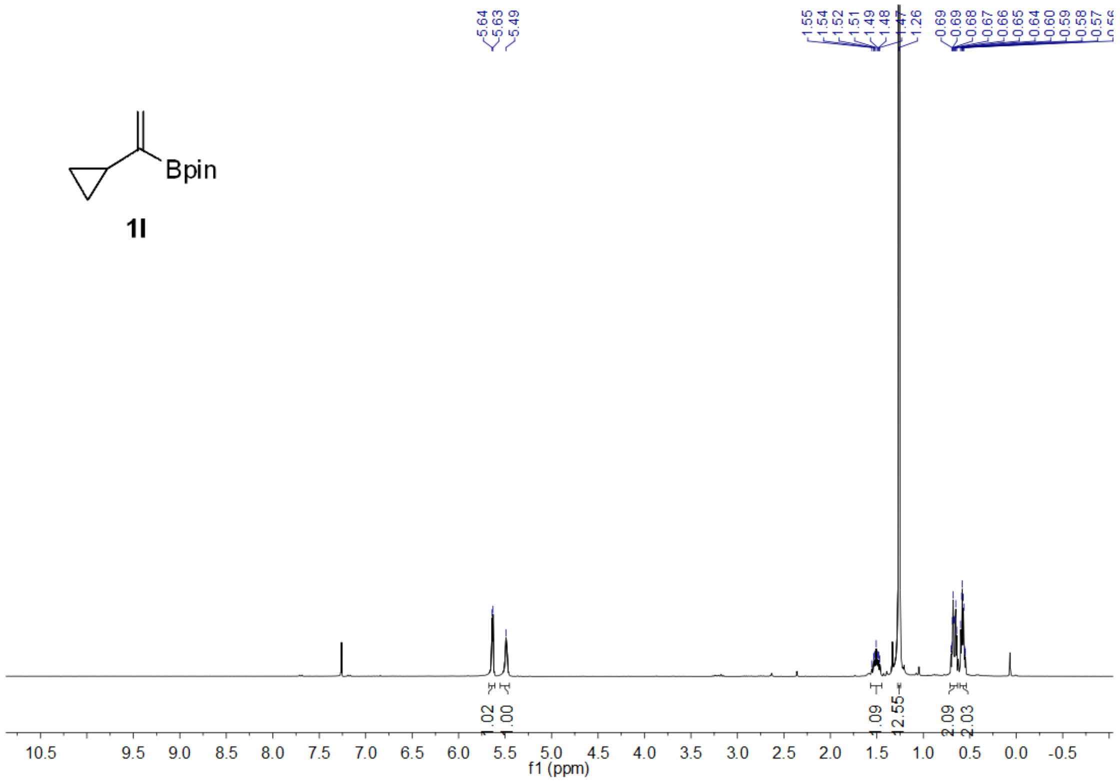

5.64
5.63
5.49
1.55
1.54
1.52
1.51
1.49
1.48
1.47
1.26
0.69
0.69
0.68
0.67
0.66
0.65
0.64
0.60
0.59
0.58
0.57
Bpin
1l
1.02
1.00
1.09
12.55
2.09
2.03
10.5
9.5
9.0
8.5
8.0
7.5
7.0
6.5
6.0
5.5
5.0
4.5
4.0
3.5
3.0
2.5
2.0
1.5
1.0
0.5
0.0
-0.5
f1 (ppm)

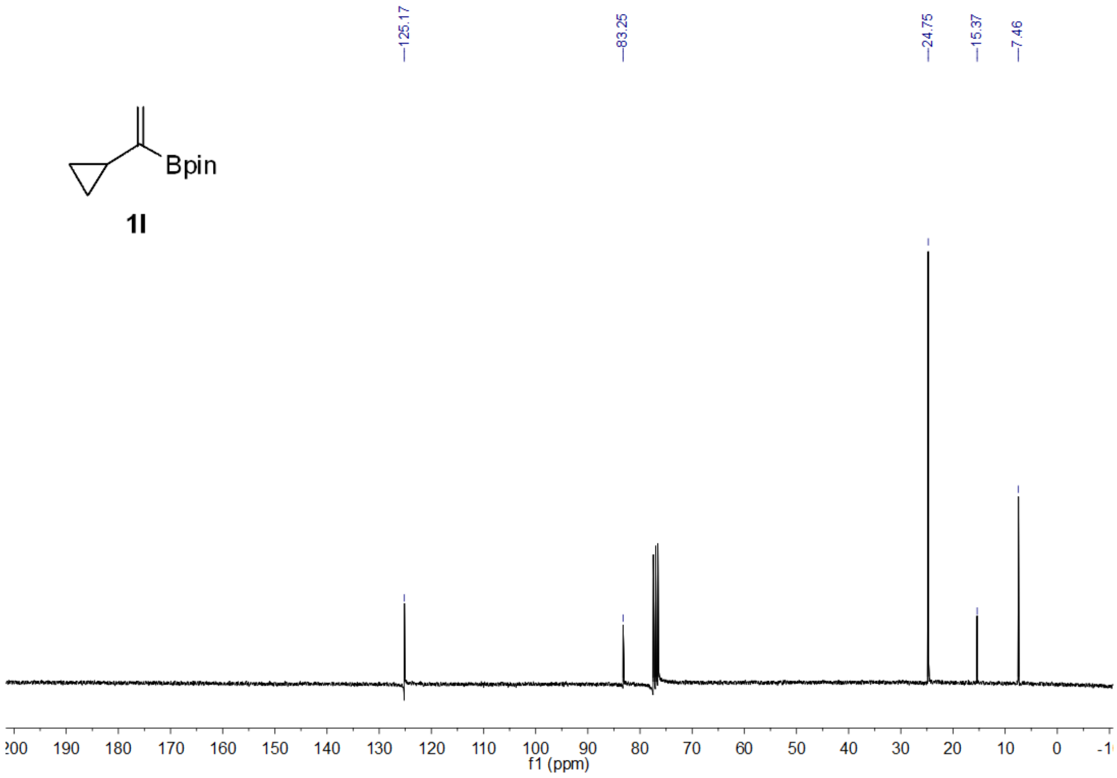
125.17
83.25
24.75
15.37
7.46
Bpin
1l
f1 (ppm)

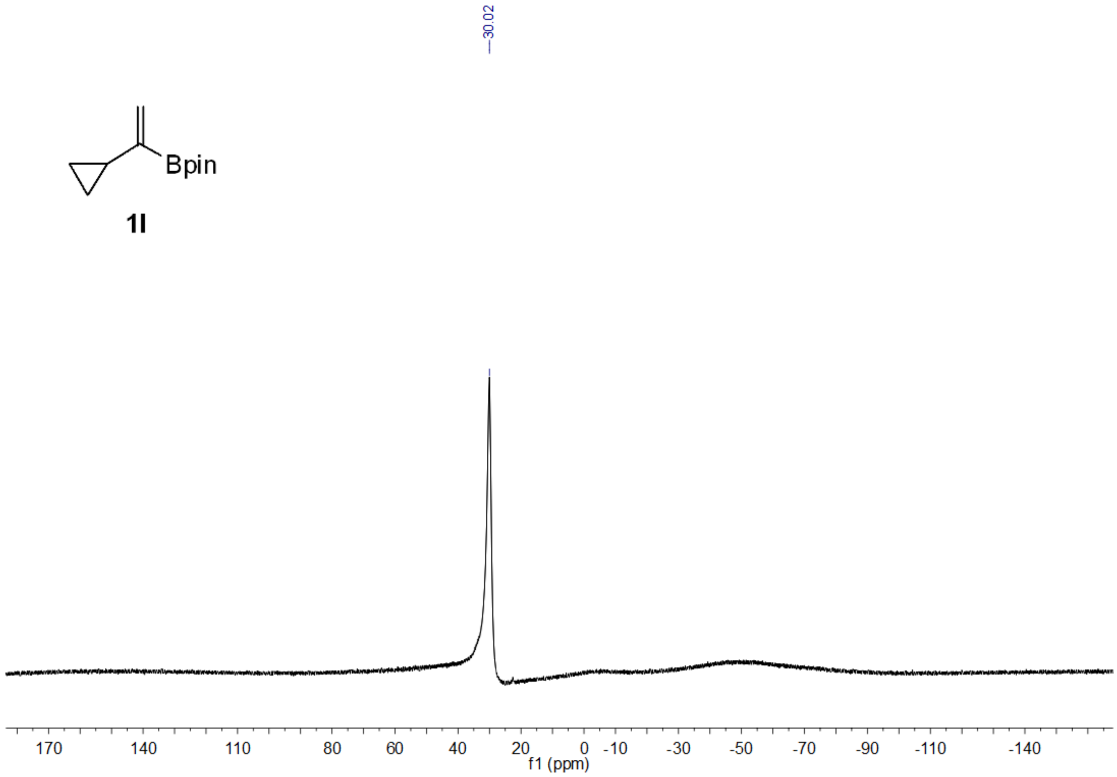
30.02
Bpin
1l
f1 (ppm)

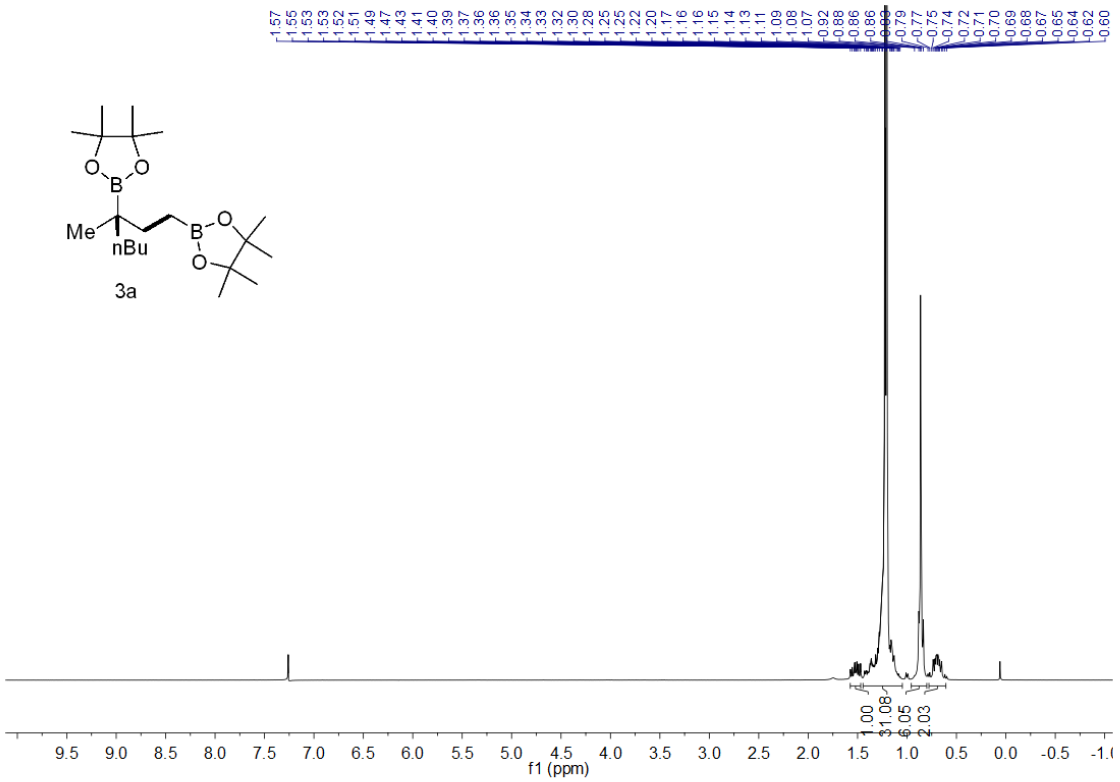
1.57 1.55 1.53 1.52 1.51 1.49 1.47 1.43 1.41 1.40 1.39 1.37 1.36 1.36 1.35 1.34 1.33 1.32 1.30 1.28 1.25 1.25 1.22 1.20 1.17 1.16 1.16 1.15 1.14 1.13 1.11 1.09 1.08 1.07 0.92 0.88 0.86 0.86 0.79 0.77 0.75 0.74 0.72 0.71 0.70 0.69 0.68 0.67 0.65 0.64 0.62 0.60
Me
nBu
3a
1.00
31.08
6.05
2.03
f1 (ppm)

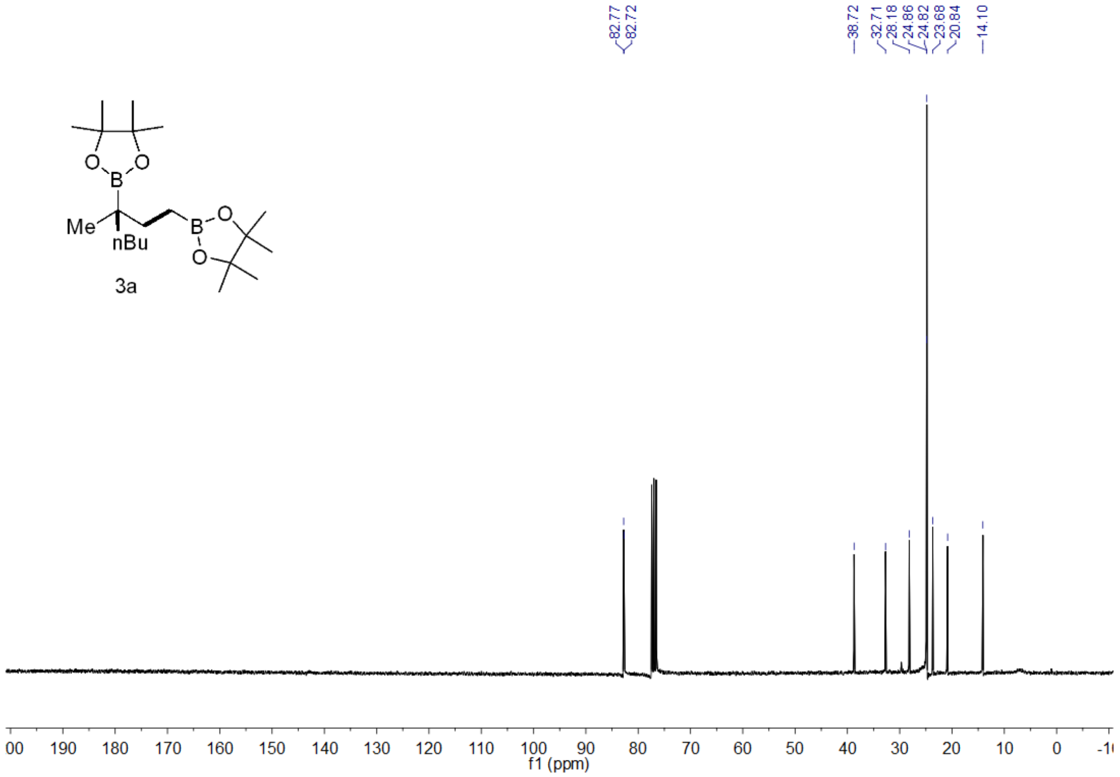
82.77
82.72
38.72
32.71
28.18
24.86
24.82
23.68
20.84
14.10
Me
nBu
3a
f1 (ppm)

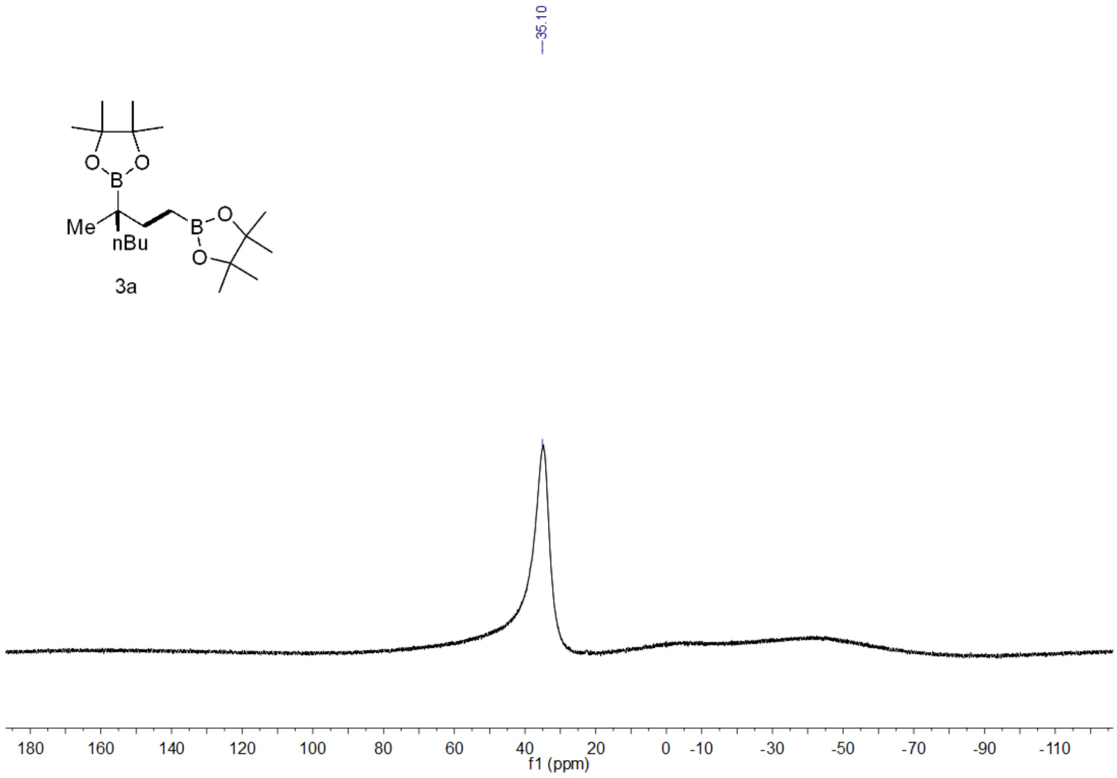
35.10
Me
nBu
O
B
3a
180 160 140 120 100 80 60 40 20 0 -10 -30 -50 -70 -90 -110
f1 (ppm)

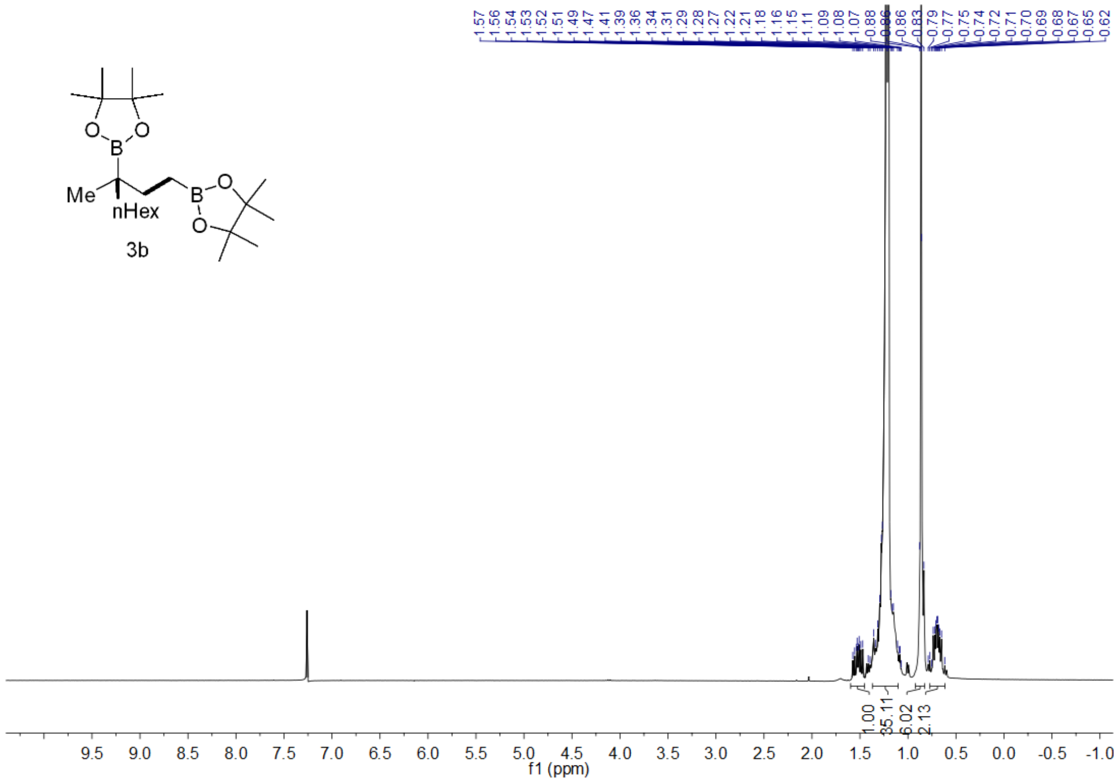
1.57 1.56 1.54 1.53 1.52 1.51 1.49 1.47 1.41 1.39 1.36 1.34 1.31 1.29 1.28 1.27 1.22 1.21 1.18 1.16 1.15 1.11 1.09 1.08 1.07 0.88 0.86 0.86 0.83 0.79 0.77 0.75 0.74 0.72 0.71 0.70 0.69 0.68 0.67 0.65 0.62
Me
nHex
O
B
3b
1.00
35.11
6.02
2.13
9.5 9.0 8.5 8.0 7.5 7.0 6.5 6.0 5.5 5.0 4.5 4.0 3.5 3.0 2.5 2.0 1.5 1.0 0.5 0.0 -0.5 -1.0
f1 (ppm)

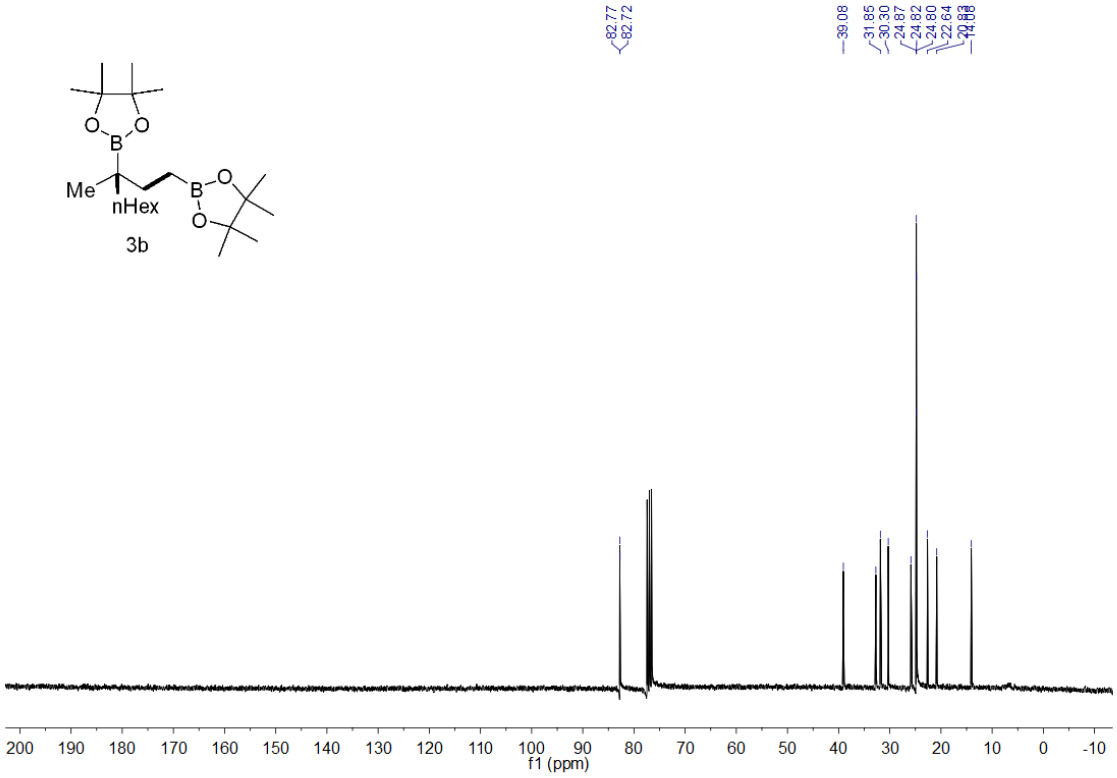
82.77
82.72
39.08
31.85
30.30
24.87
24.82
24.80
22.64
Me
nHex
3b
200 190 180 170 160 150 140 130 120 110 100 90 80 70 60 50 40 30 20 10 0 -10
f1 (ppm)

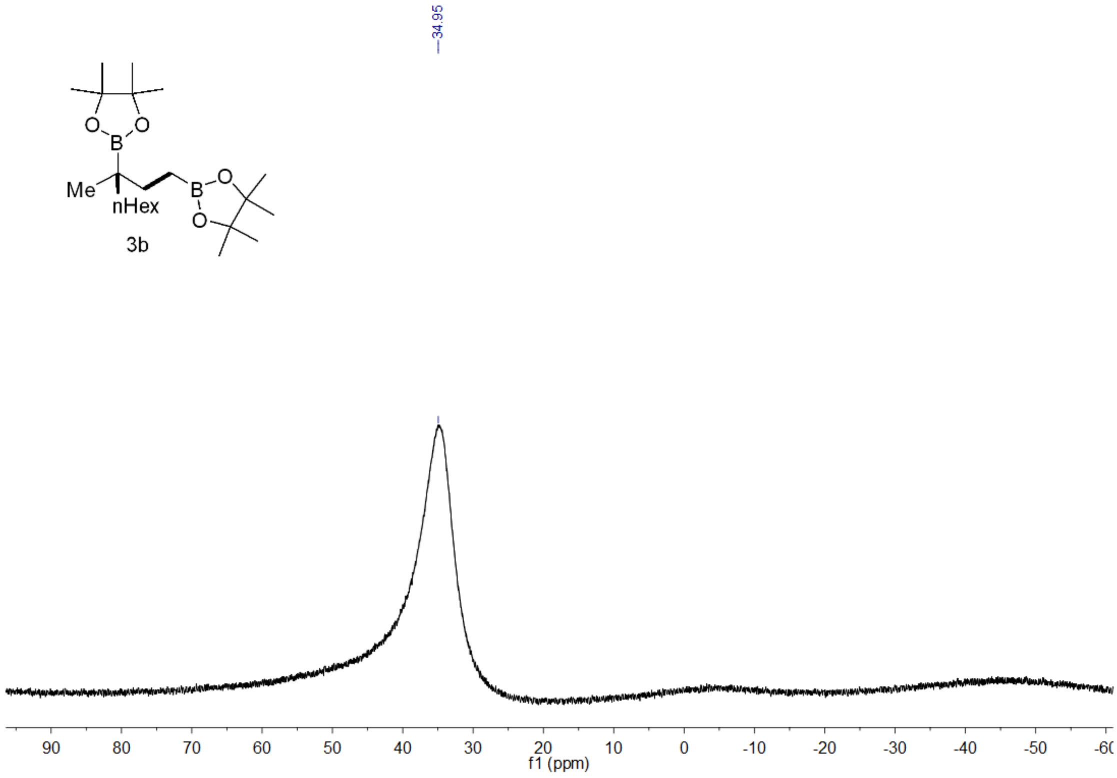
34.95
Me
nHex
3b
90 80 70 60 50 40 30 20 10 0 -10 -20 -30 -40 -50
f1 (ppm)

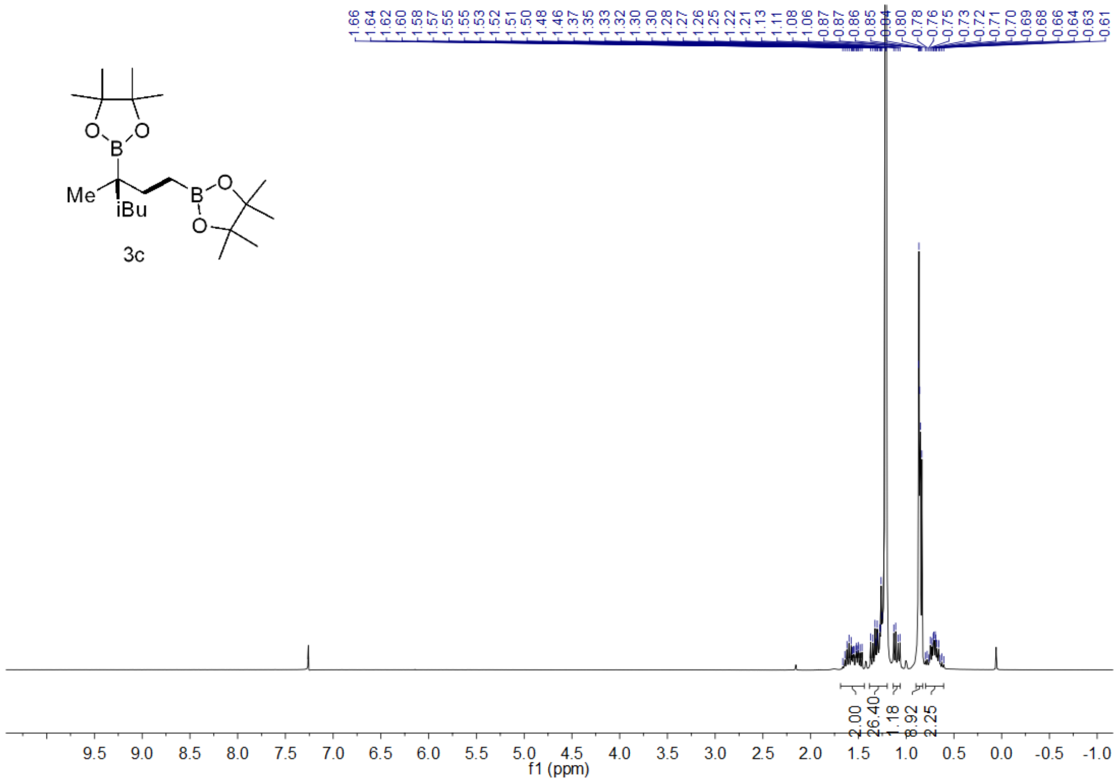
Me
iBu
3c
1.66 1.64 1.62 1.60 1.58 1.57 1.55 1.55 1.53 1.52 1.51 1.50 1.48 1.46 1.37 1.35 1.33 1.32 1.30 1.30 1.28 1.27 1.26 1.25 1.22 1.21 1.13 1.11 1.08 1.06 0.87 0.87 0.86 0.85 0.84 0.80 0.78 0.76 0.75 0.73 0.72 0.71 0.70 0.69 0.68 0.66 0.64 0.63 0.61
2.00 26.40 1.18 8.92 2.25
f1 (ppm)

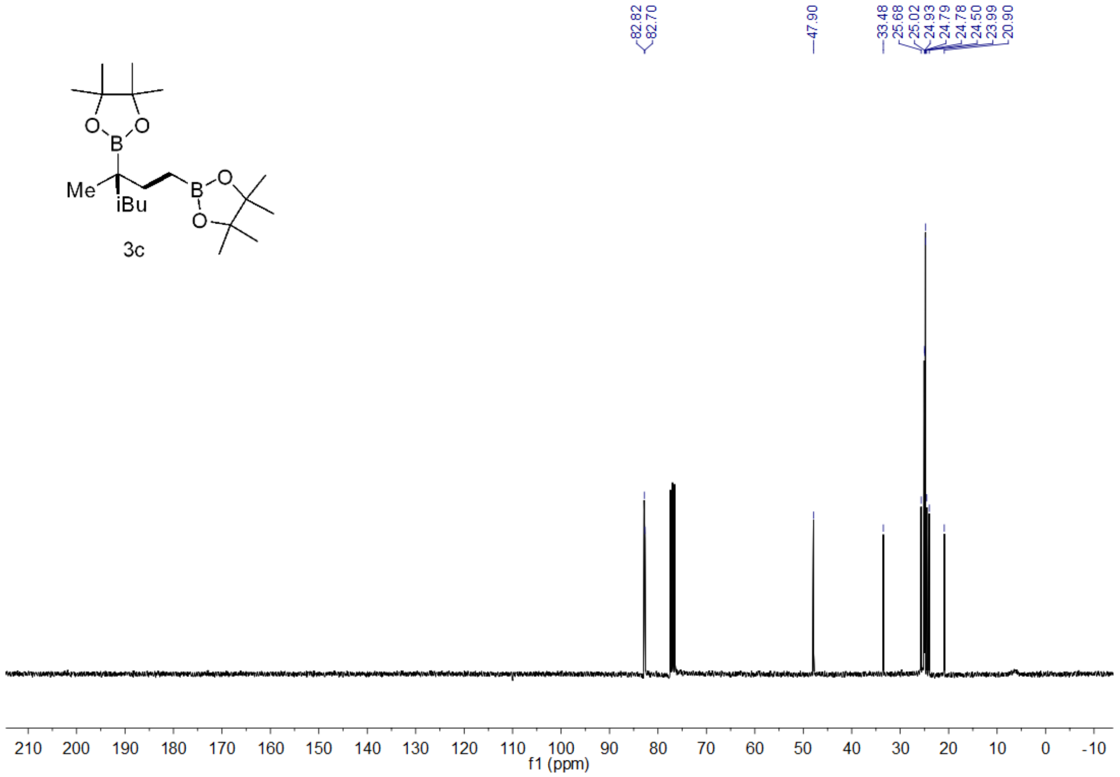
Me
iBu
3c
82.82 82.70
47.90
33.48 25.68 25.02 24.93 24.79 24.78 24.50 23.99 20.90
f1 (ppm)

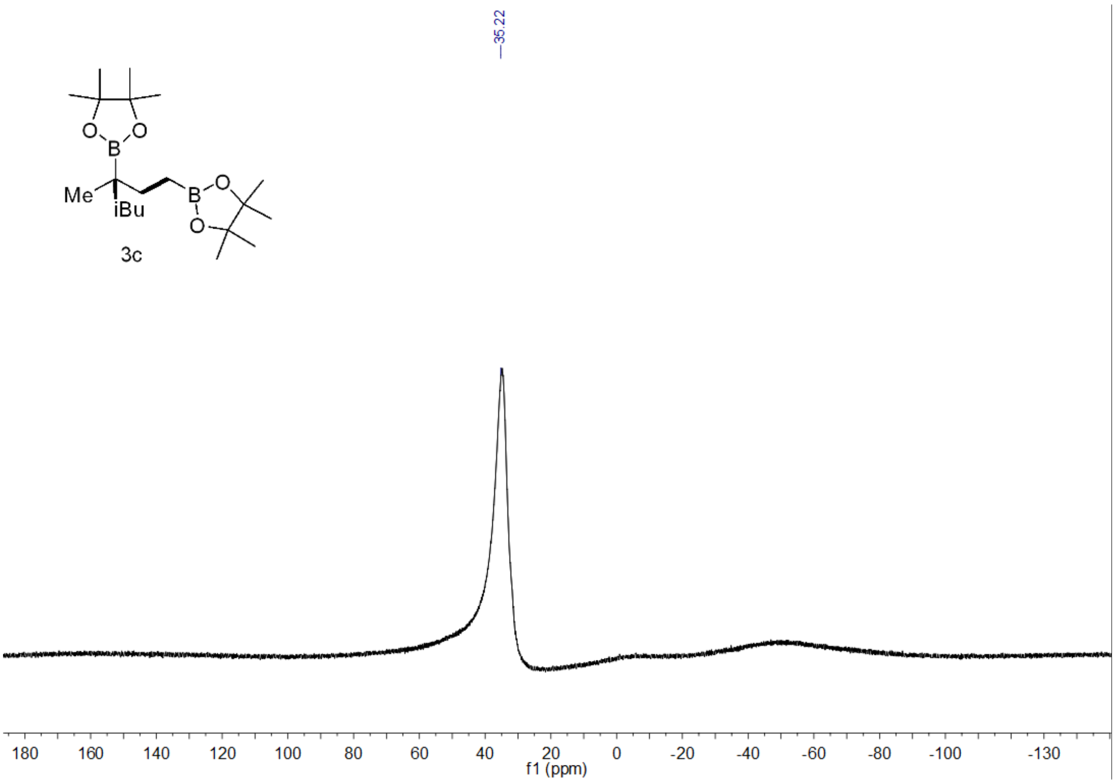
35.22
O B O
Me
iBu
B O O
3c
180 160 140 120 100 80 60 40 20 0 -20 -40 -60 -80 -100 -130
f1 (ppm)

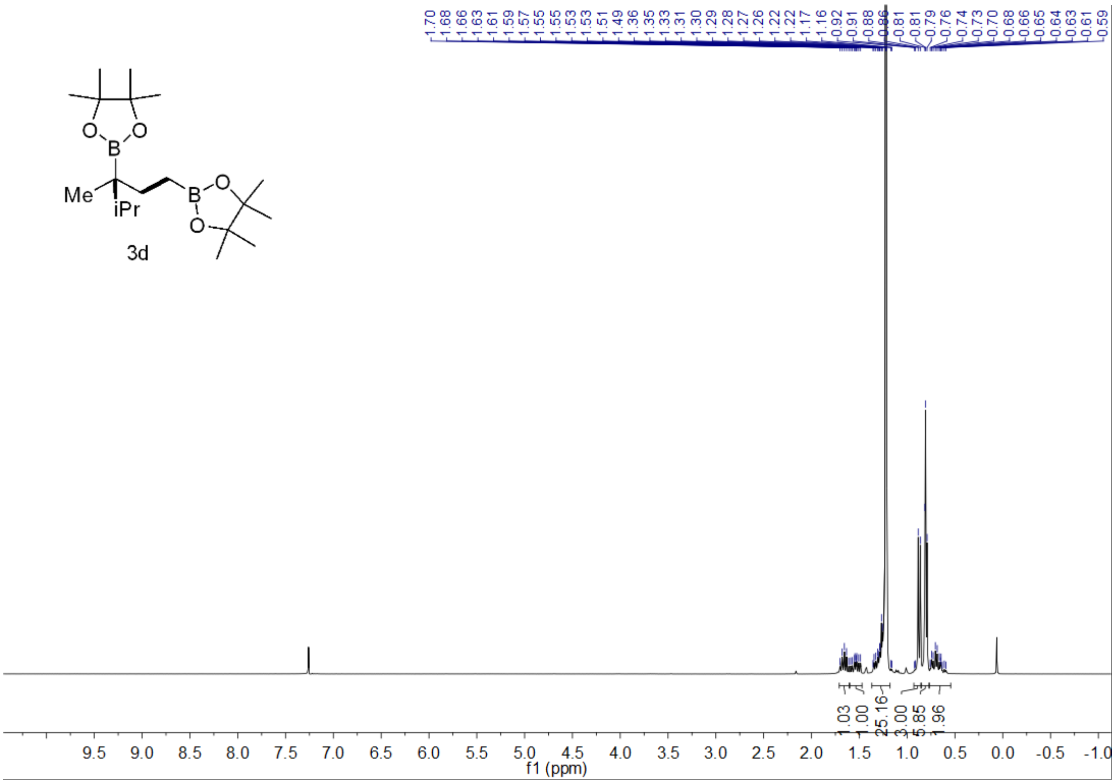
1.70 1.68 1.66 1.63 1.61 1.59 1.57 1.55 1.53 1.53 1.51 1.49 1.36 1.35 1.33 1.31 1.30 1.29 1.28 1.27 1.26 1.22 1.22 1.17 1.16 0.92 0.91 0.88 0.86 0.81 0.81 0.79 0.76 0.74 0.73 0.70 0.68 0.66 0.65 0.64 0.63 0.61 0.59
O B O
Me
iPr
B O O
3d
1.03
1.00
25.16
3.00
5.85
1.96
9.5 9.0 8.5 8.0 7.5 7.0 6.5 6.0 5.5 5.0 4.5 4.0 3.5 3.0 2.5 2.0 1.5 1.0 0.5 0.0 -0.5 -1.0
f1 (ppm)

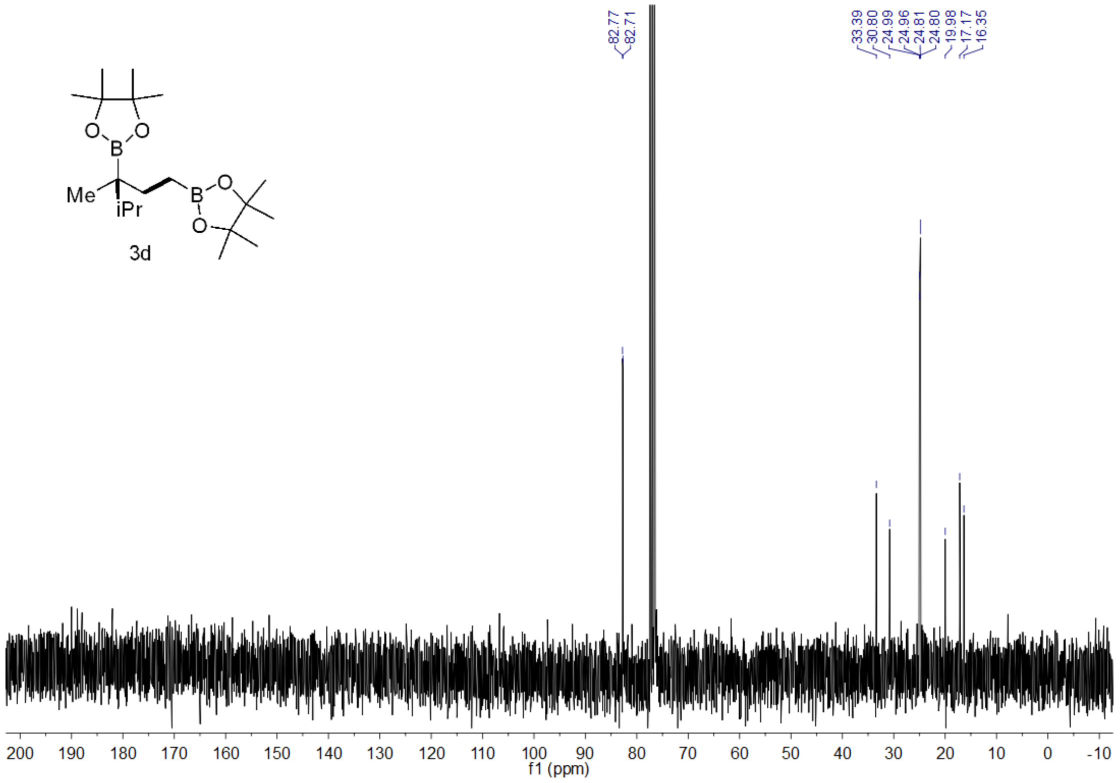

82.77
82.71
33.39
30.80
24.99
24.96
24.81
24.80
19.98
17.17
16.35
Me
iPr
3d
f1 (ppm)

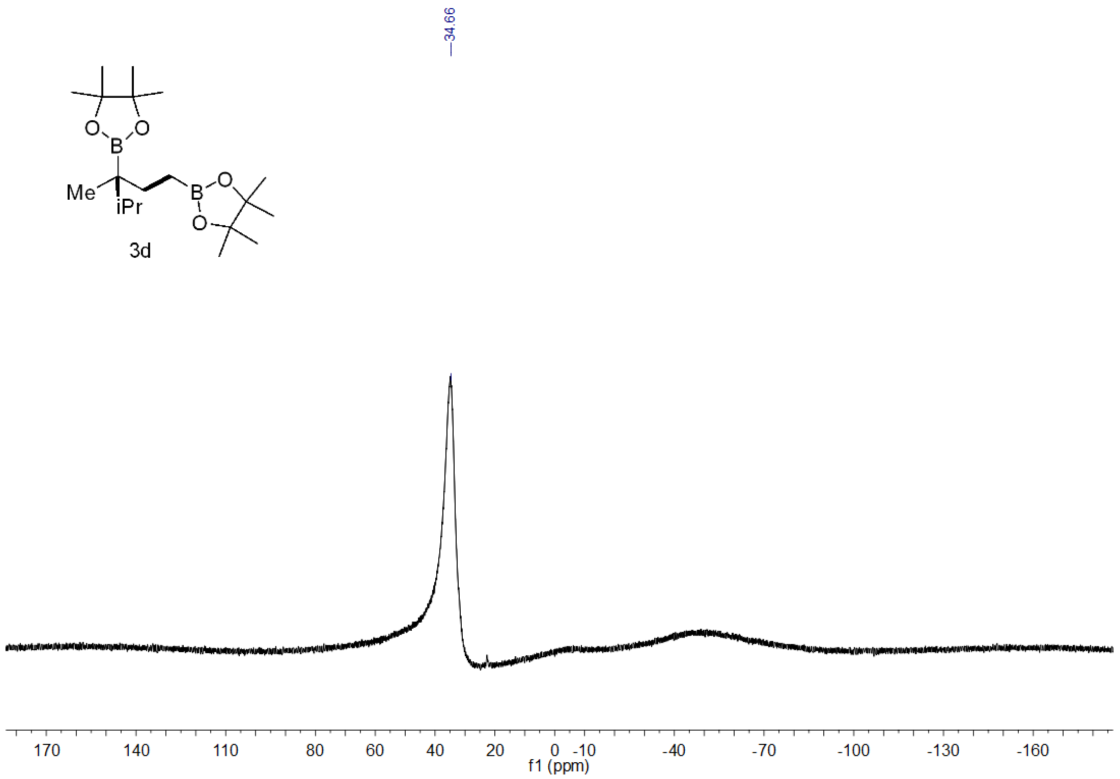

34.66
Me
iPr
3d
f1 (ppm)

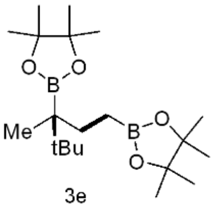
O
B
O
Me
tBu
B
O
O

3e

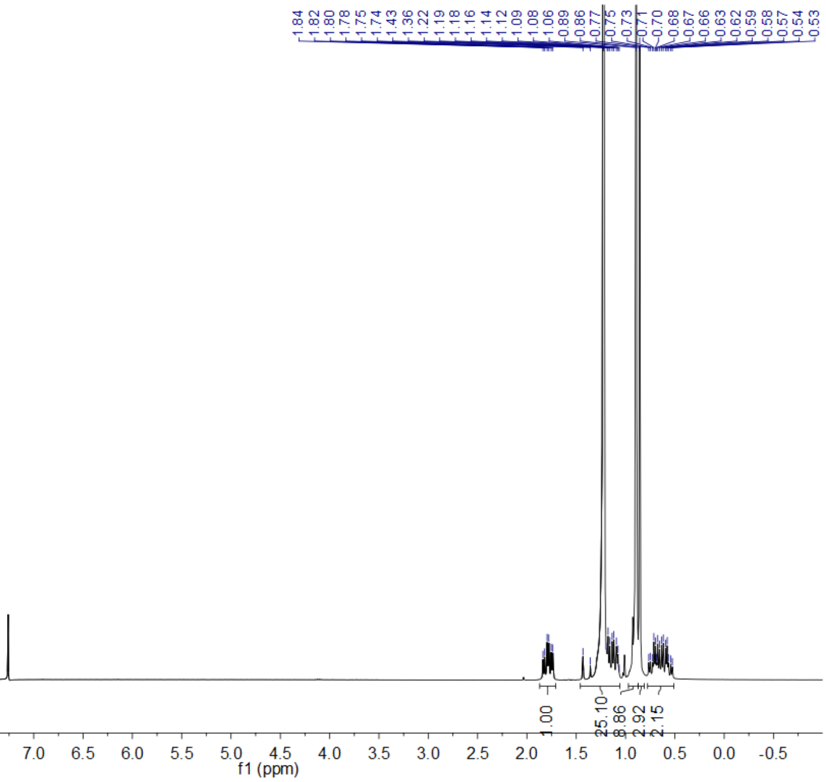
1.84
1.82
1.80
1.78
1.75
1.74
1.43
1.36
1.22
1.19
1.18
1.16
1.14
1.12
1.09
1.08
1.06
0.89
0.86
0.77
0.75
0.73
0.71
0.70
0.68
0.67
0.66
0.63
0.62
0.59
0.58
0.57
0.54
0.53
1.00
25.10
8.86
2.92
2.15
9.5 9.0 8.5 8.0 7.5 7.0 6.5 6.0 5.5 5.0 4.5 4.0 3.5 3.0 2.5 2.0 1.5 1.0 0.5 0.0 -0.5
f1 (ppm)

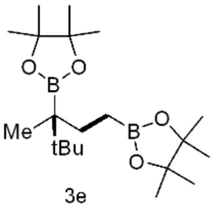
O
B
O
Me
tBu
B
O
O

3e

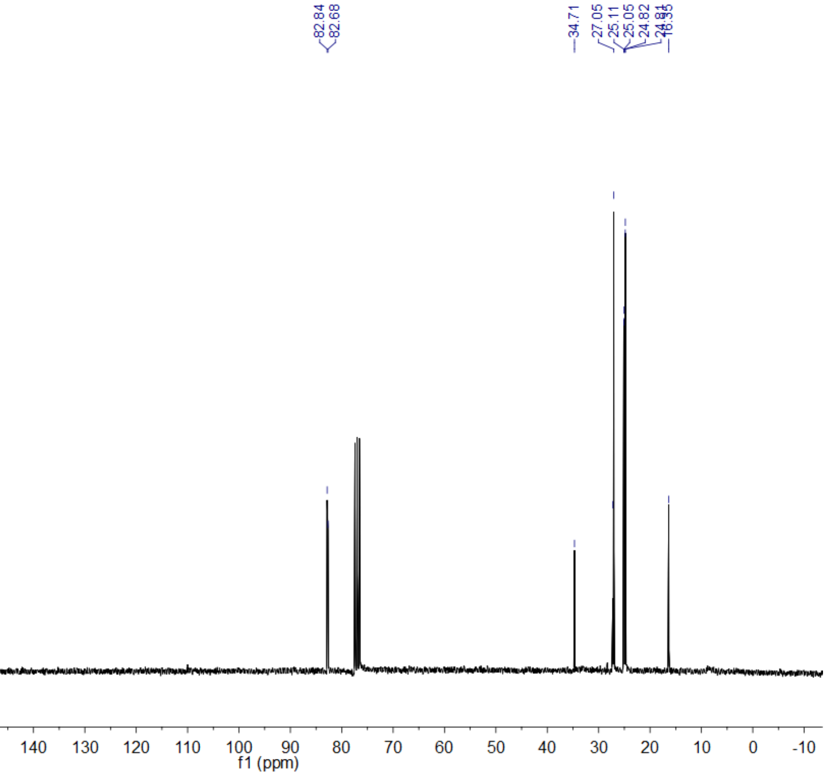
82.84
82.68
34.71
27.05
25.11
25.05
24.82
16.35
200 190 180 170 160 150 140 130 120 110 100 90 80 70 60 50 40 30 20 10 0 -10
f1 (ppm)

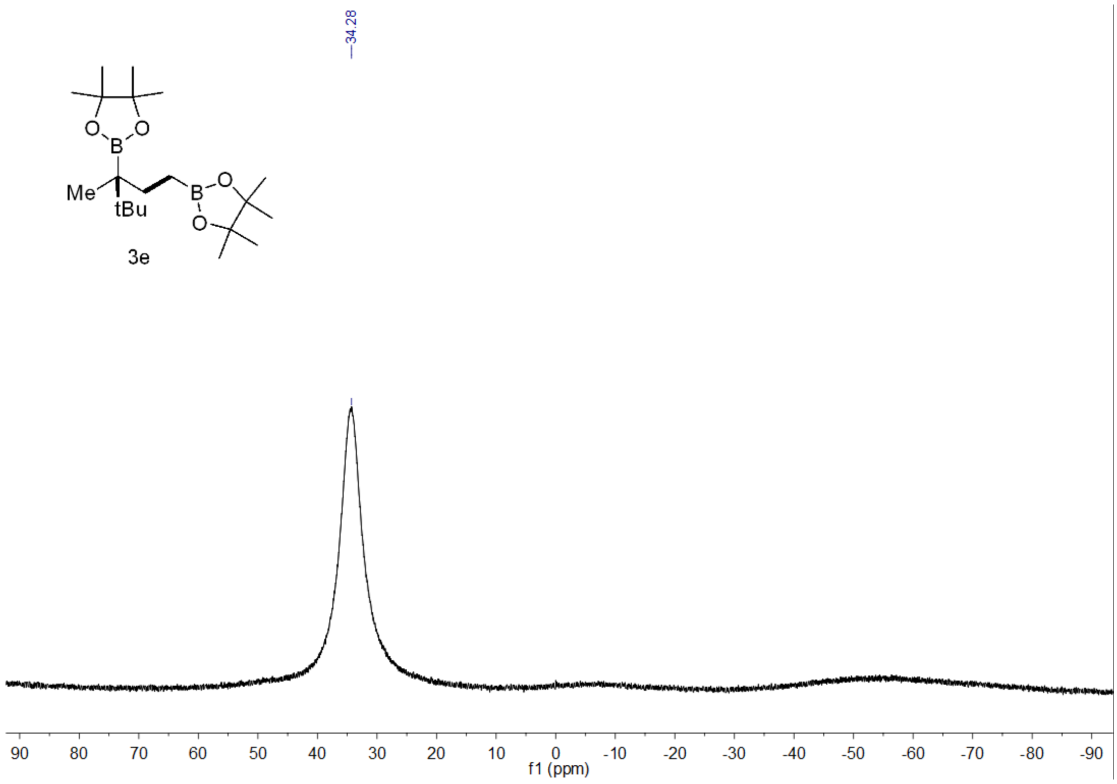
34.28
Me
tBu
3e
f1 (ppm)

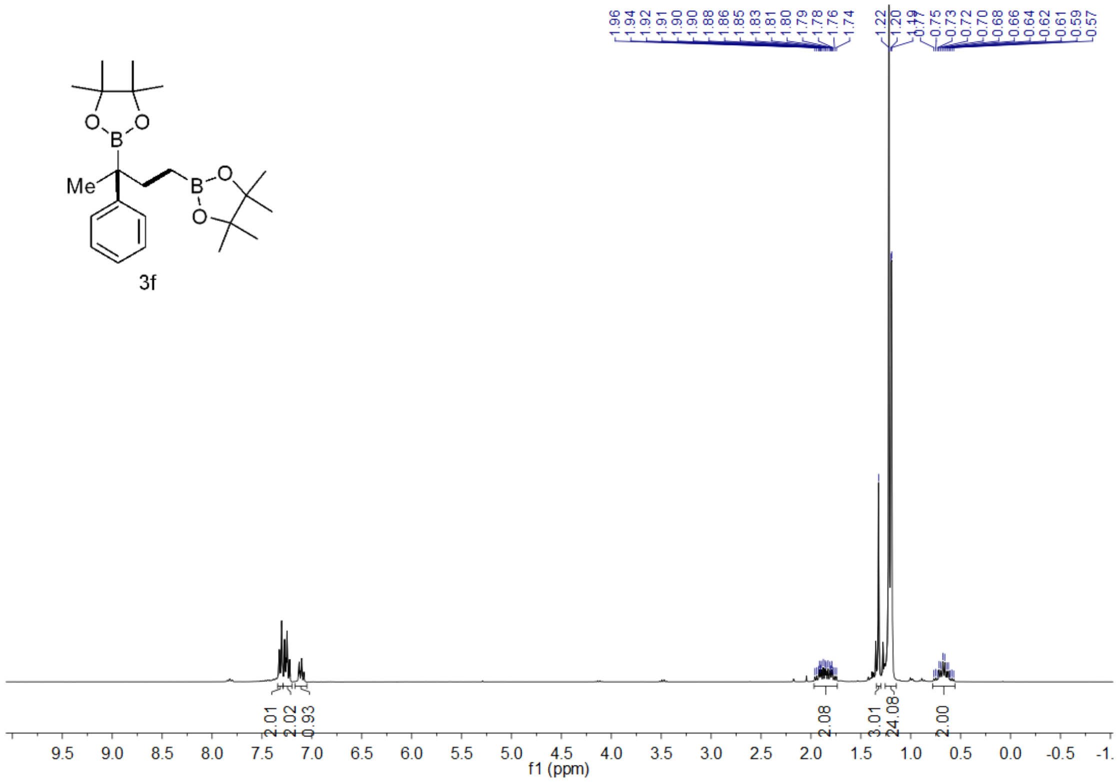
1.96 1.94 1.92 1.91 1.90 1.90 1.88 1.86 1.85 1.83 1.81 1.80 1.79 1.78 1.76 1.74
1.22 1.20 1.19 1.17 0.75 0.73 0.72 0.70 0.68 0.66 0.64 0.62 0.61 0.59 0.57
Me
3f
2.01 2.02 0.93 2.08 3.01 24.08 2.00
f1 (ppm)

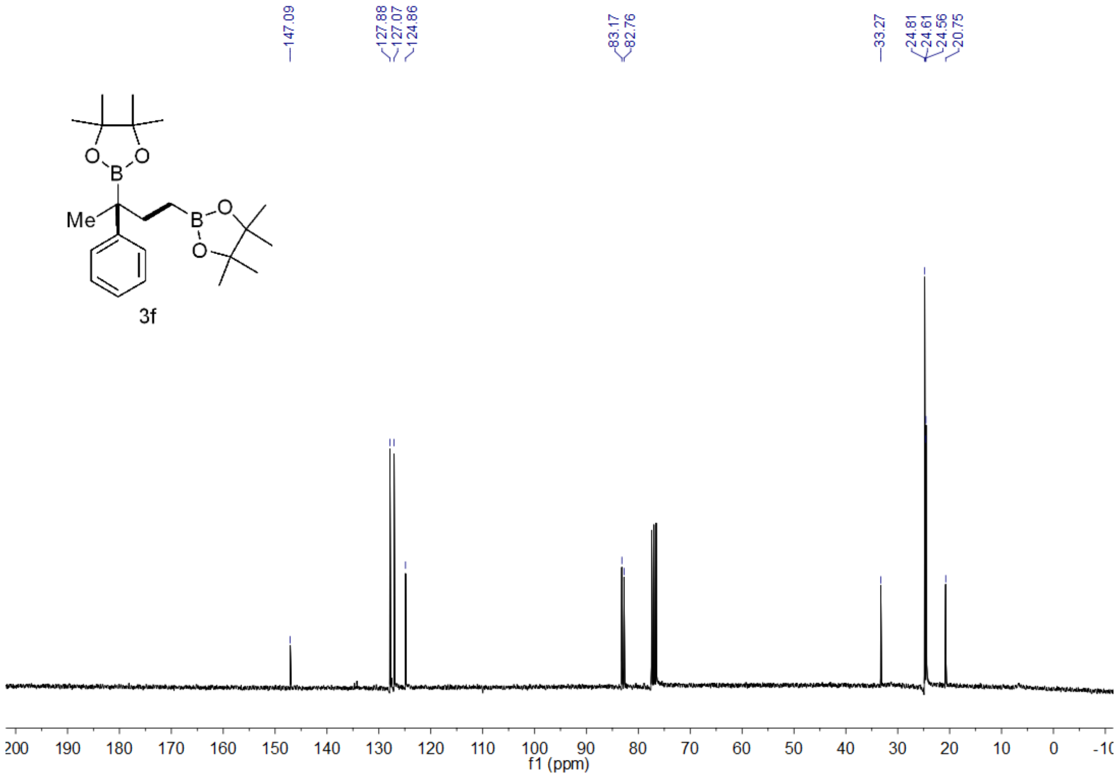
147.09
127.88
127.07
124.86
83.17
82.76
33.27
24.81
24.61
24.56
20.75
Me
O
B
O
3f
200 190 180 170 160 150 140 130 120 110 100 90 80 70 60 50 40 30 20 10 0
f1 (ppm)

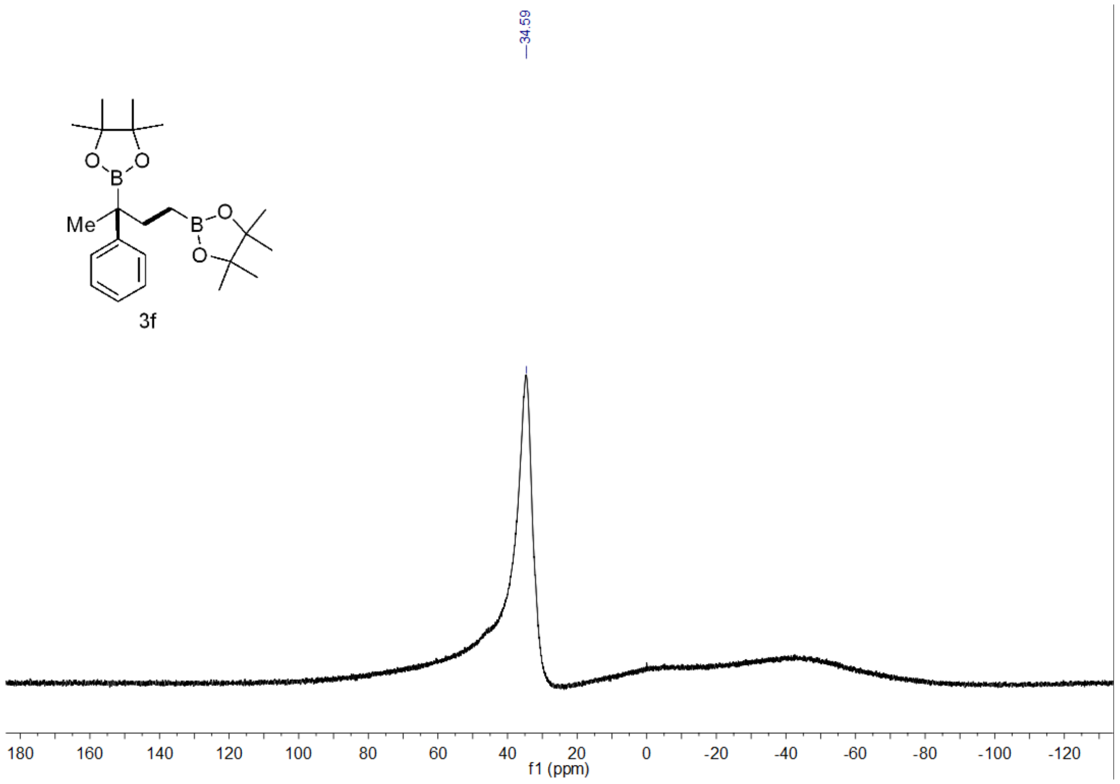
34.59
Me
O
B
O
3f
180 160 140 120 100 80 60 40 20 0 -20 -40 -60 -80 -100 -120
f1 (ppm)

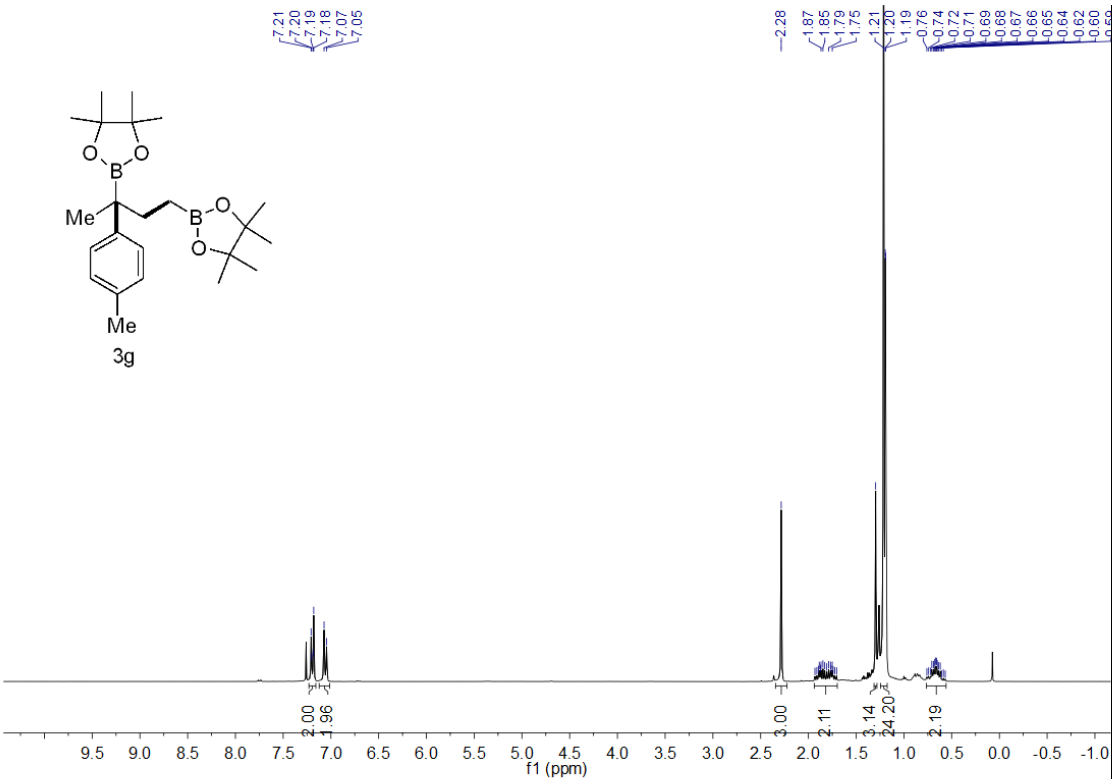
7.21
7.20
7.19
7.18
7.07
7.05
2.28
1.87
1.85
1.79
1.75
1.21
1.20
1.19
0.76
0.74
0.72
0.71
0.69
0.68
0.67
0.66
0.65
0.64
0.62
0.60
Me
B
O
Me
3g
2.00
1.96
3.00
2.11
3.14
24.20
2.19
f1 (ppm)

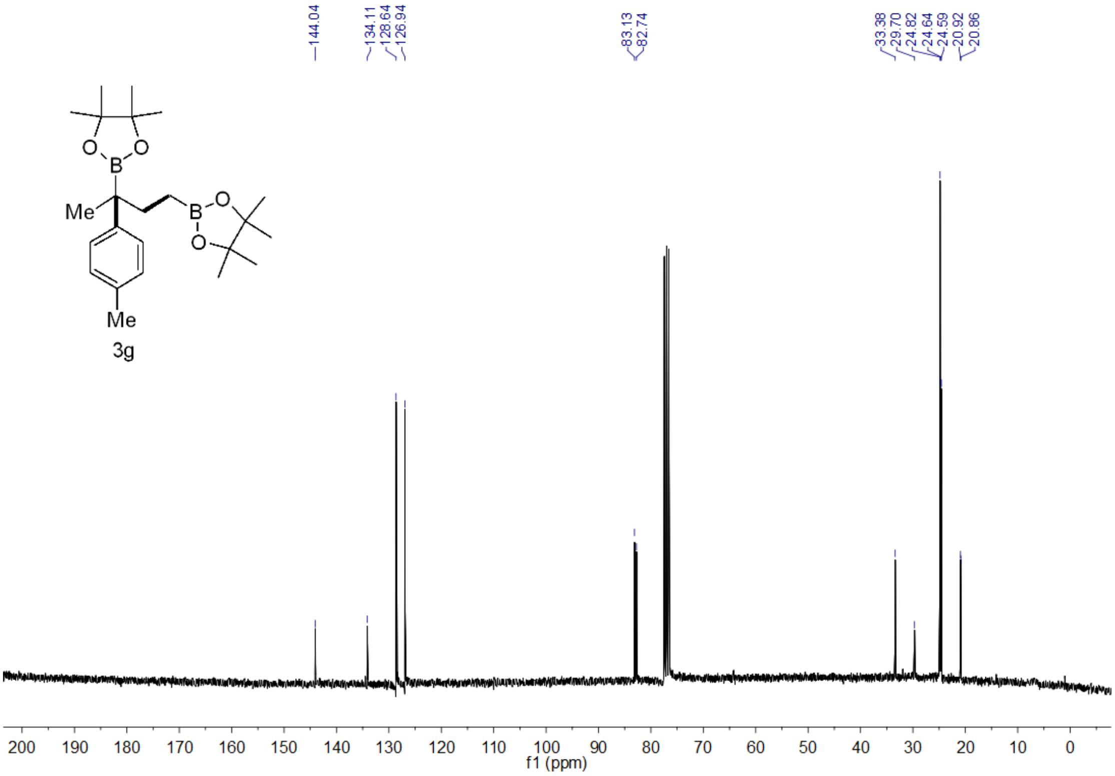
144.04
134.11
128.64
126.94
83.13
82.74
33.38
29.70
24.82
24.64
24.59
20.92
20.86
Me
B
O
Me
3g
f1 (ppm)

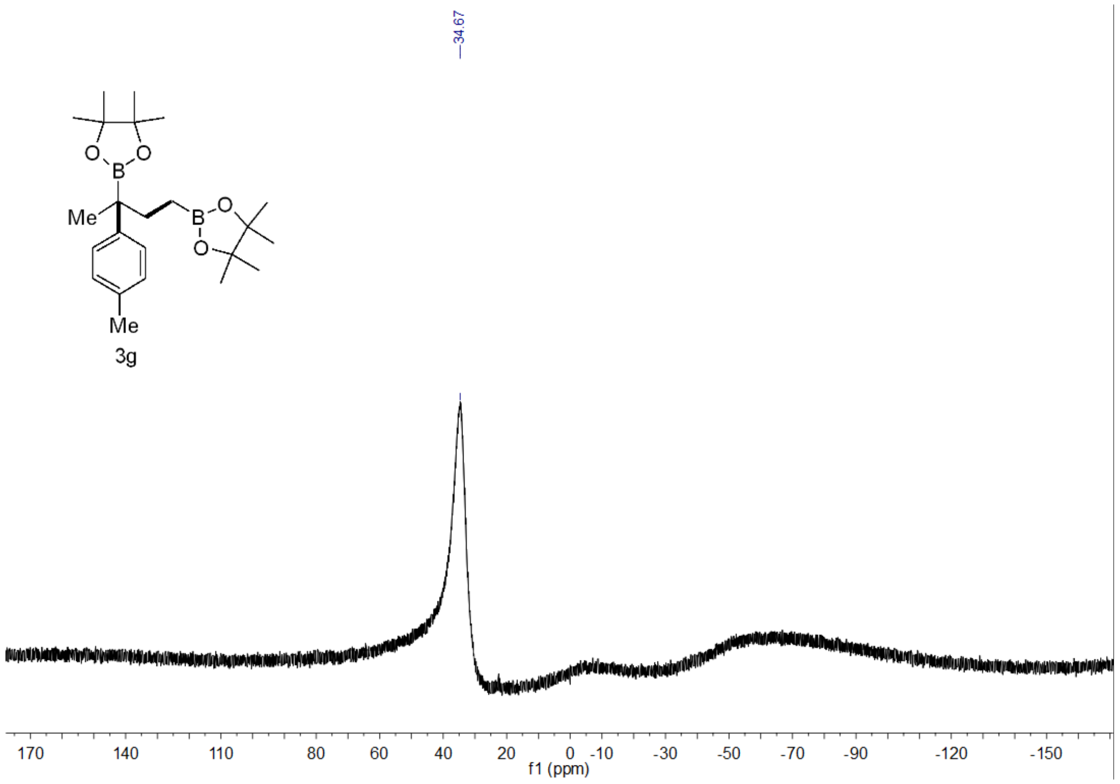
34.67
Me
Me
3g
170 140 110 80 60 40 20 0 -10 -30 -50 -70 -90 -120 -150
f1 (ppm)

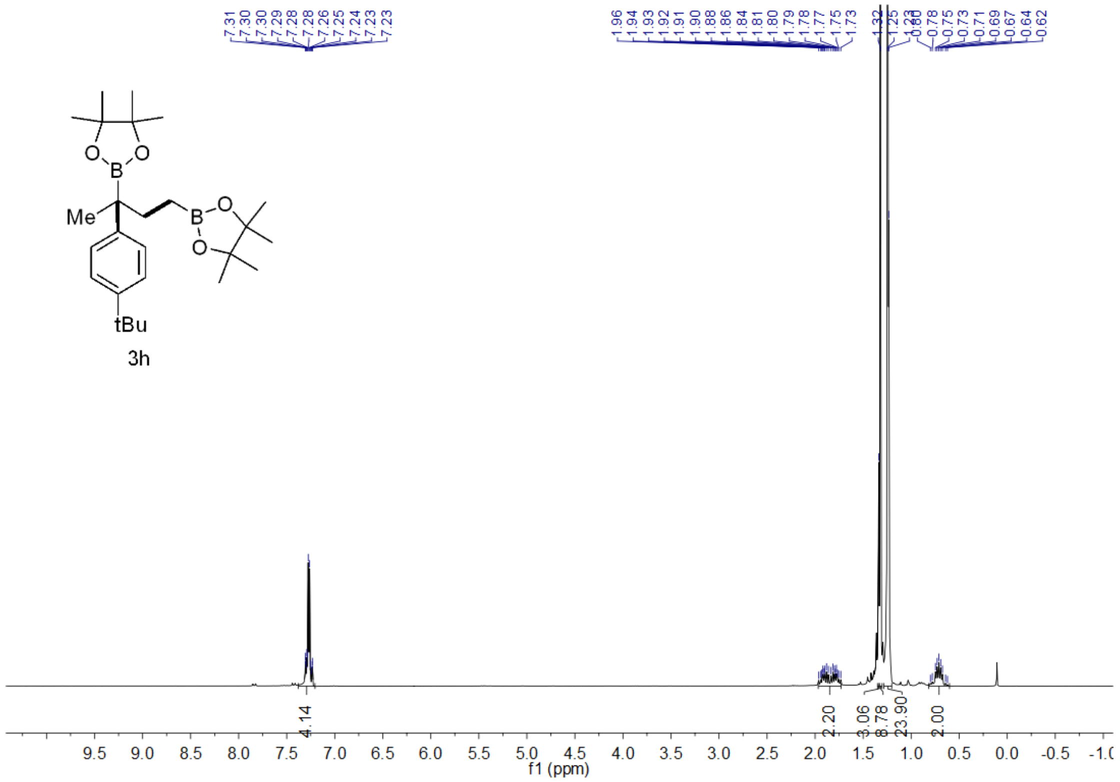
7.31 7.30 7.30 7.29 7.28 7.28 7.26 7.25 7.24 7.23 7.23
1.96 1.94 1.93 1.92 1.91 1.90 1.88 1.86 1.84 1.81 1.80 1.79 1.78 1.77 1.75 1.73
1.32 1.25
0.78 0.75 0.73 0.71 0.69 0.67 0.64 0.62
Me
tBu
3h
4.14 2.20 3.06 8.78 23.90 2.00
9.5 9.0 8.5 8.0 7.5 7.0 6.5 6.0 5.5 5.0 4.5 4.0 3.5 3.0 2.5 2.0 1.5 1.0 0.5 0.0 -0.5
f1 (ppm)

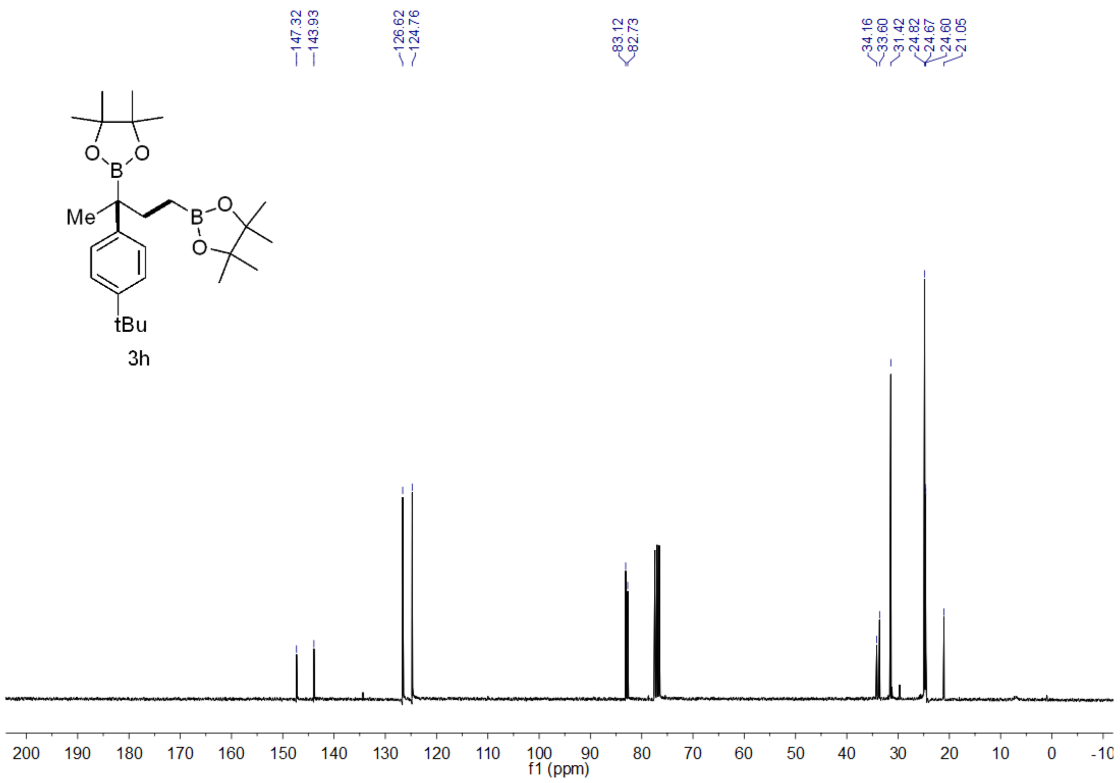
147.32
143.93
126.62
124.76
83.12
82.73
34.16
33.60
31.42
24.82
24.67
24.60
21.05
Me
tBu
3h
f1 (ppm)

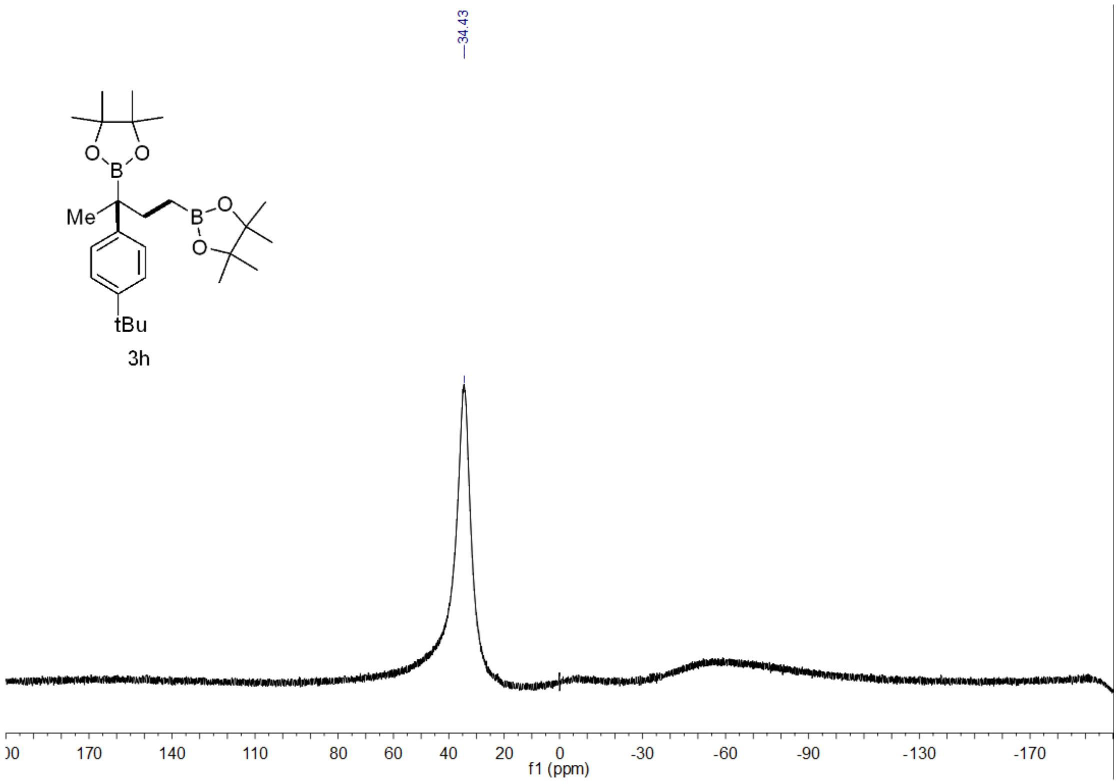
34.43
Me
tBu
3h
f1 (ppm)

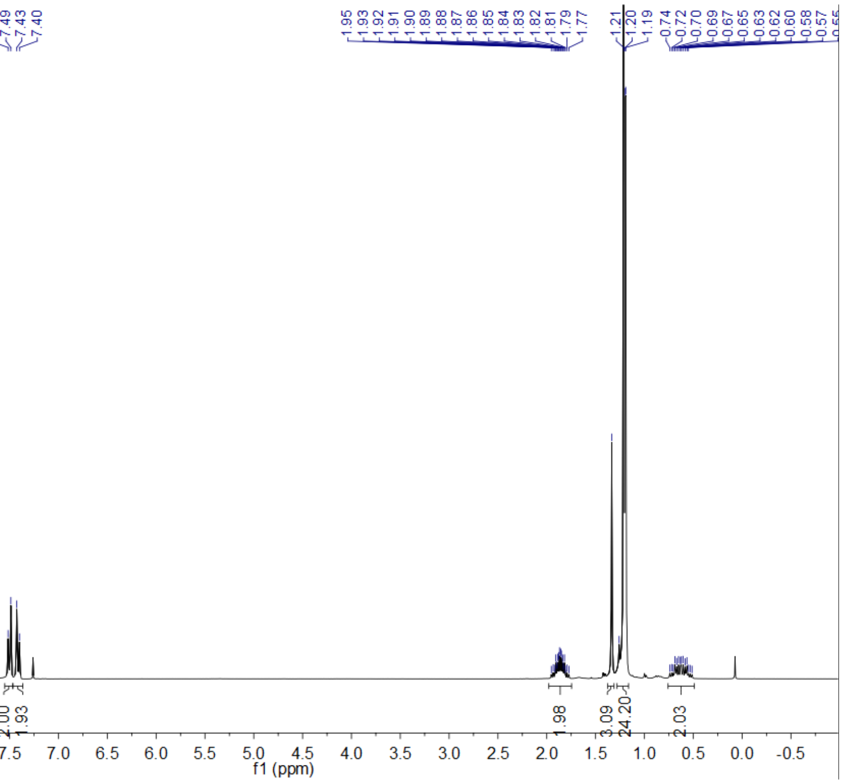
7.52
7.49
7.43
7.40
1.95
1.93
1.92
1.91
1.90
1.89
1.88
1.87
1.86
1.85
1.84
1.83
1.82
1.81
1.79
1.77
1.21
1.20
1.19
0.74
0.72
0.70
0.69
0.67
0.65
0.63
0.62
0.60
0.58
0.57
2.00
1.93
1.98
3.09
24.20
2.03
9.5 9.0 8.5 8.0 7.5 7.0 6.5 6.0 5.5 5.0 4.5 4.0 3.5 3.0 2.5 2.0 1.5 1.0 0.5 0.0 -0.5
f1 (ppm)

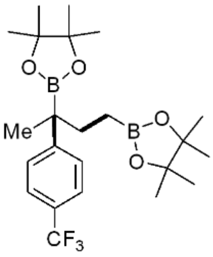
Me
B
O
O
B
O
O
CF_3

3i

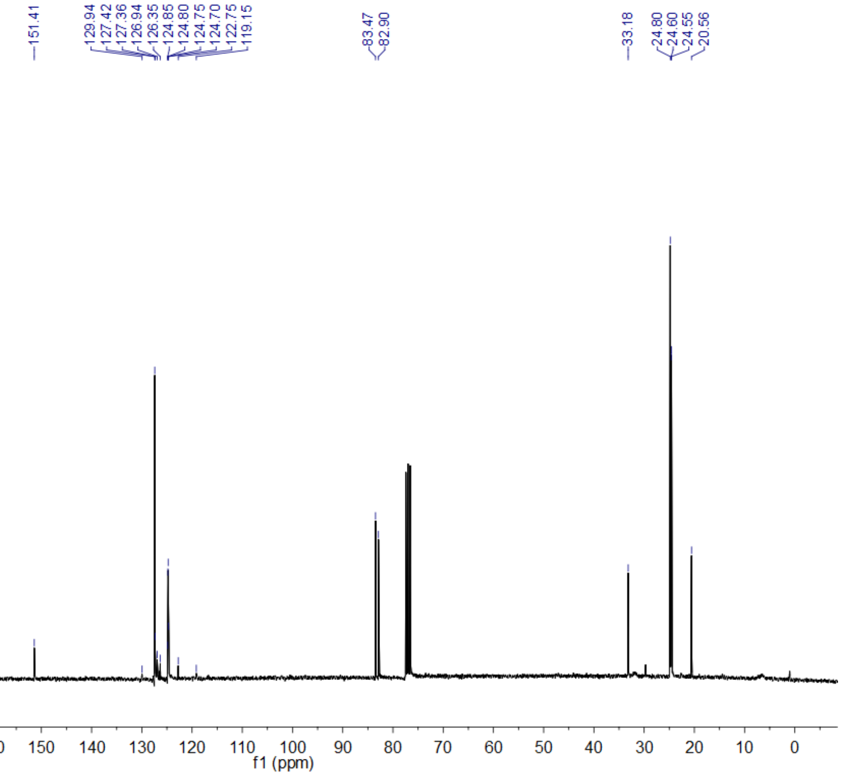
151.41
129.94
127.42
127.36
126.94
126.35
124.85
124.80
124.75
124.70
122.75
119.15
83.47
82.90
33.18
24.80
24.60
24.55
20.56
210 200 190 180 170 160 150 140 130 120 110 100 90 80 70 60 50 40 30 20 10 0
f1 (ppm)

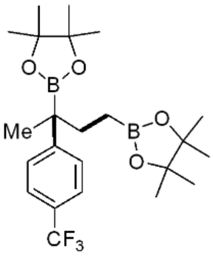
Me
B
O
O
B
O
O
CF_3

3i

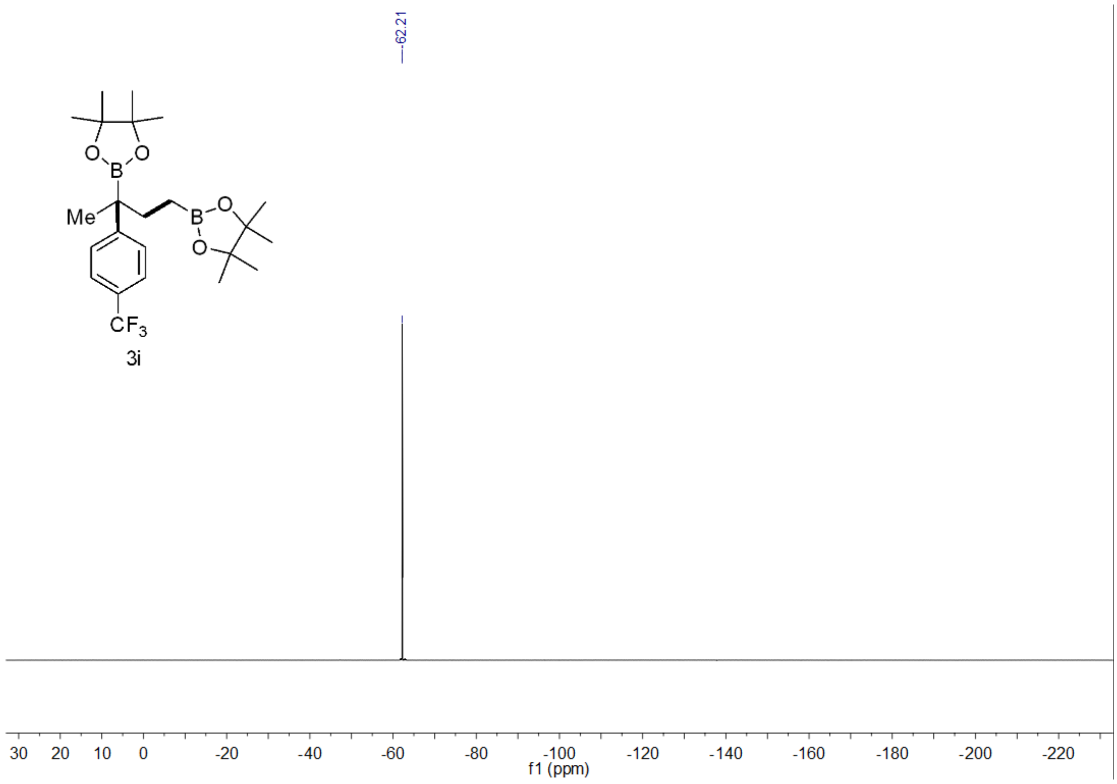
-62.21
Me
CF3
3i
30 20 10 0 -20 -40 -60 -80 -100 -120 -140 -160 -180 -200 -220
f1 (ppm)

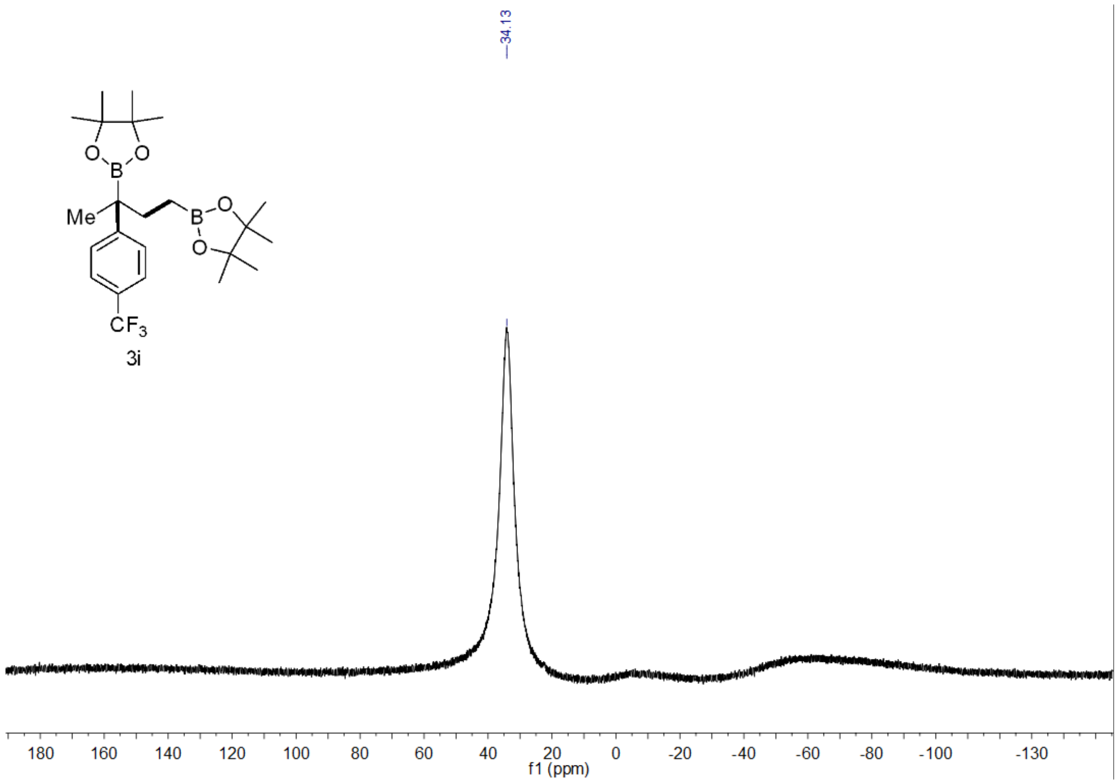
34.13
Me
CF3
3i
180 160 140 120 100 80 60 40 20 0 -20 -40 -60 -80 -100 -130
f1 (ppm)

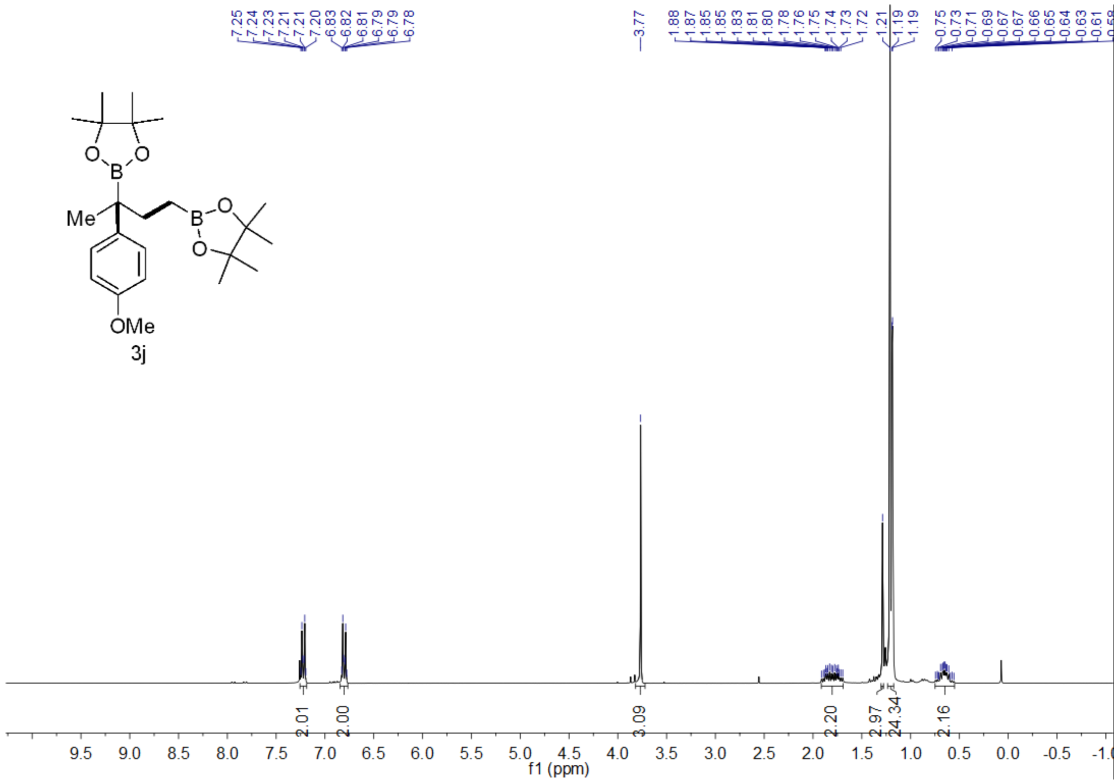
7.25
7.24
7.23
7.21
7.21
7.20
6.83
6.82
6.81
6.79
6.79
6.78
3.77
1.88
1.87
1.85
1.83
1.81
1.80
1.78
1.76
1.75
1.74
1.73
1.72
1.21
1.19
1.19
0.75
0.73
0.71
0.69
0.67
0.67
0.66
0.65
0.64
0.63
0.61
Me
B
O
O
OMe
3j
2.01
2.00
3.09
2.20
2.97
24.34
2.16
9.5 9.0 8.5 8.0 7.5 7.0 6.5 6.0 5.5 5.0 4.5 4.0 3.5 3.0 2.5 2.0 1.5 1.0 0.5 0.0 -0.5
f1 (ppm)

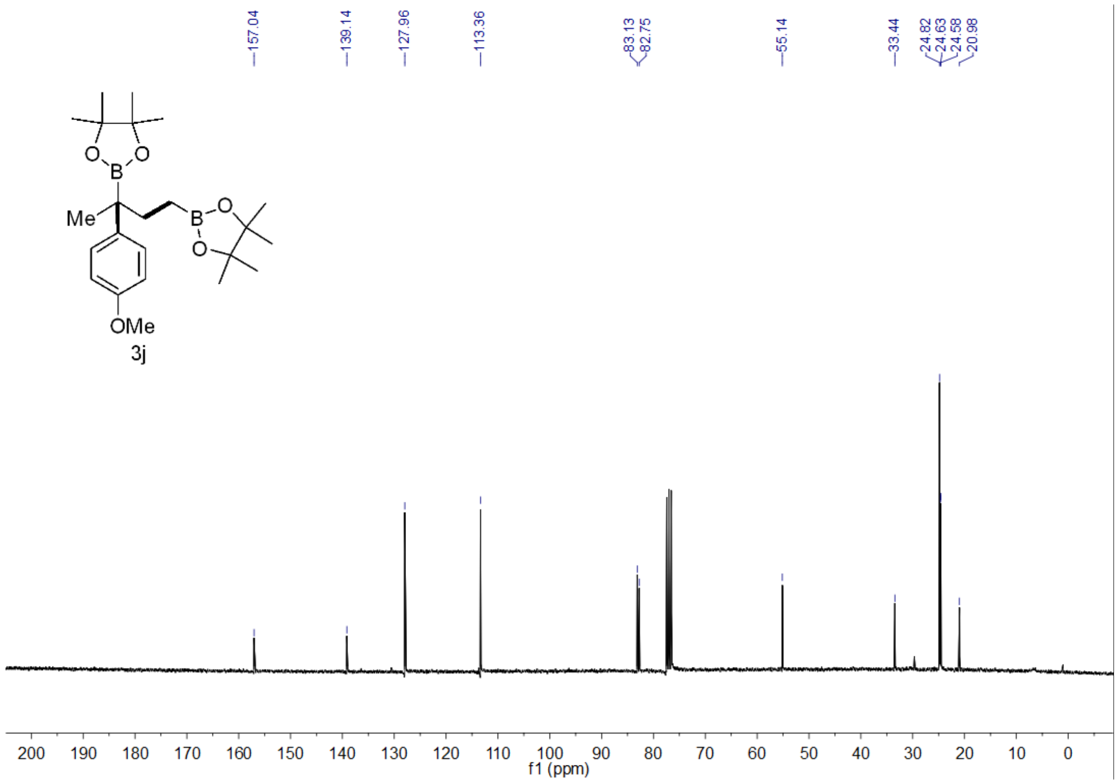
157.04
139.14
127.96
113.36
83.13
82.75
55.14
33.44
24.82
24.63
24.58
20.98
Me
B
O
O
OMe
3j
200 190 180 170 160 150 140 130 120 110 100 90 80 70 60 50 40 30 20 10 0
f1 (ppm)

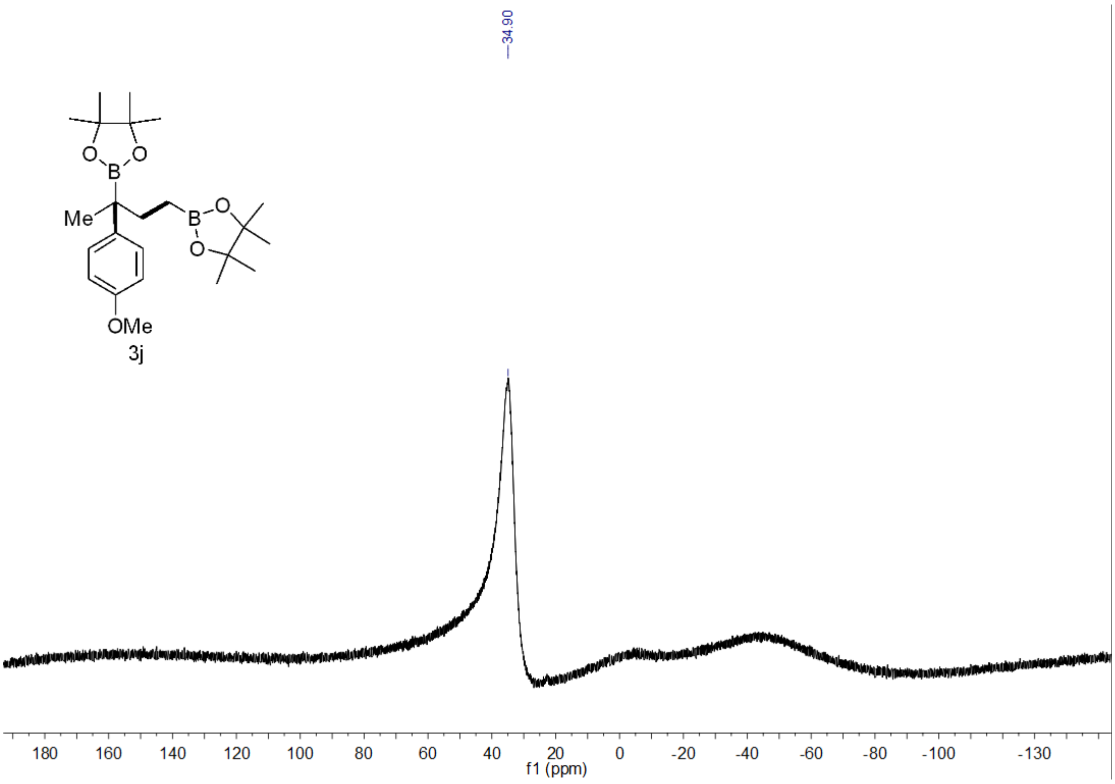

—34.90
Me
OMe
3j
f1 (ppm)

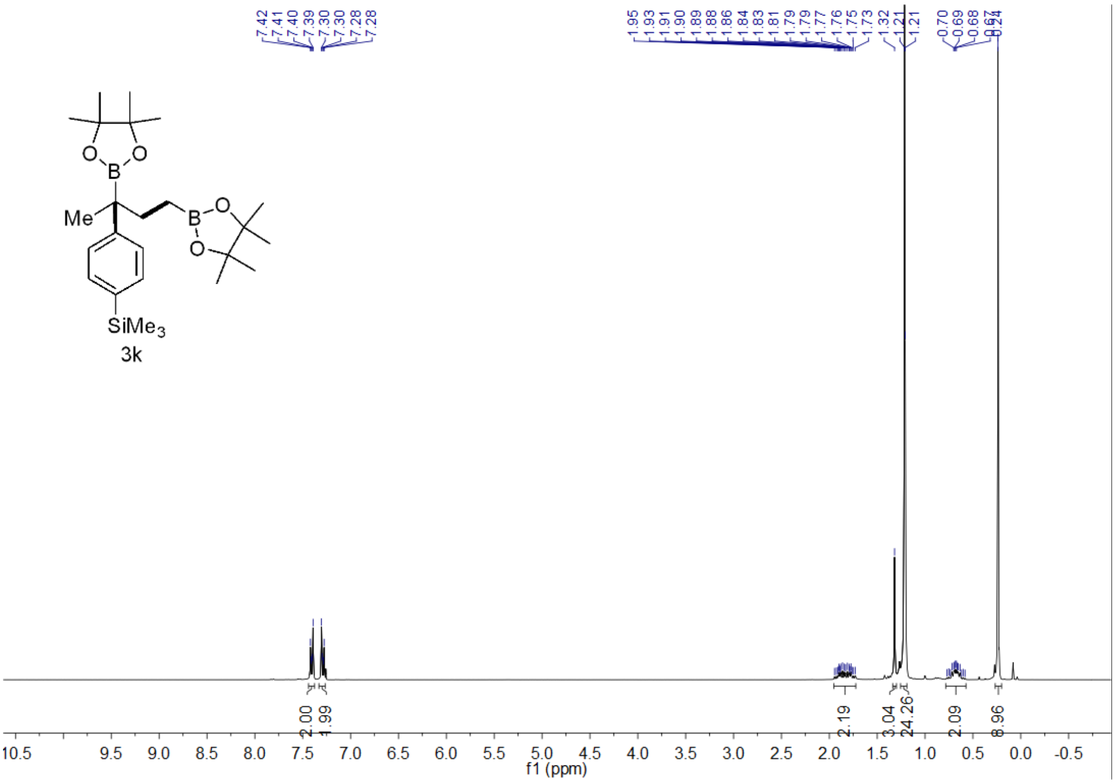

Me
SiMe3
3k
f1 (ppm)

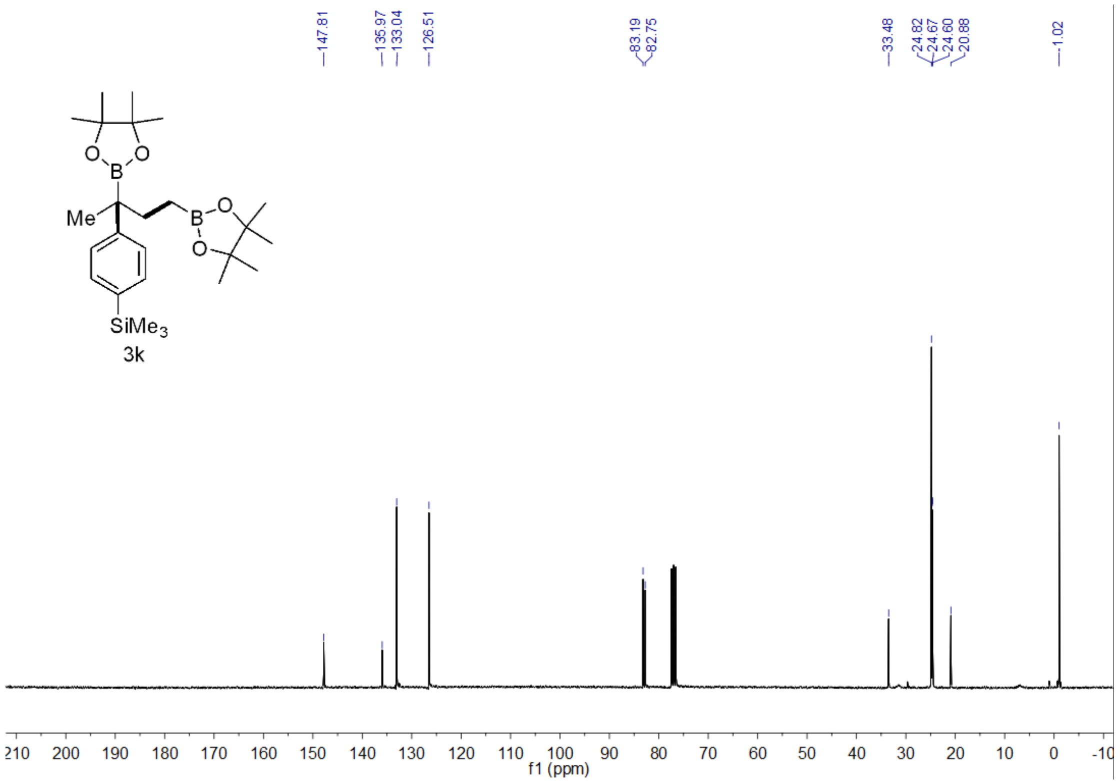
147.81
135.97
133.04
126.51
83.19
82.75
33.48
24.82
24.67
24.60
20.88
1.02
Me
SiMe3
3k
f1 (ppm)

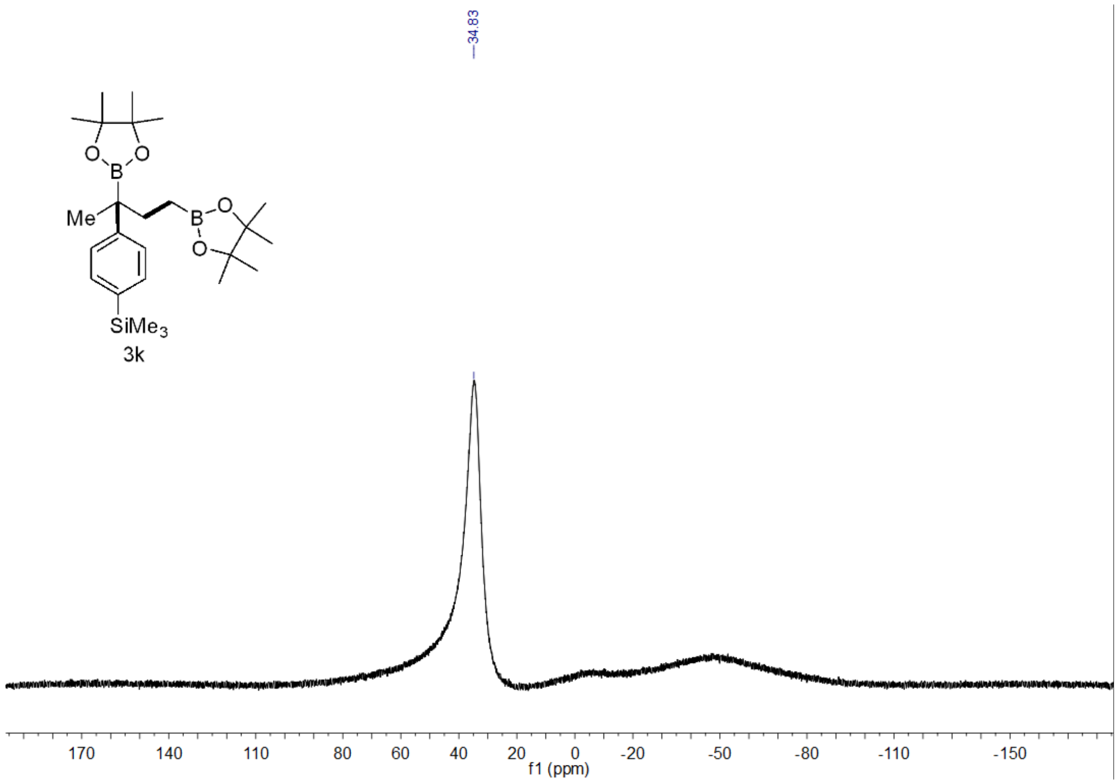
34.83
Me
SiMe3
3k
f1 (ppm)

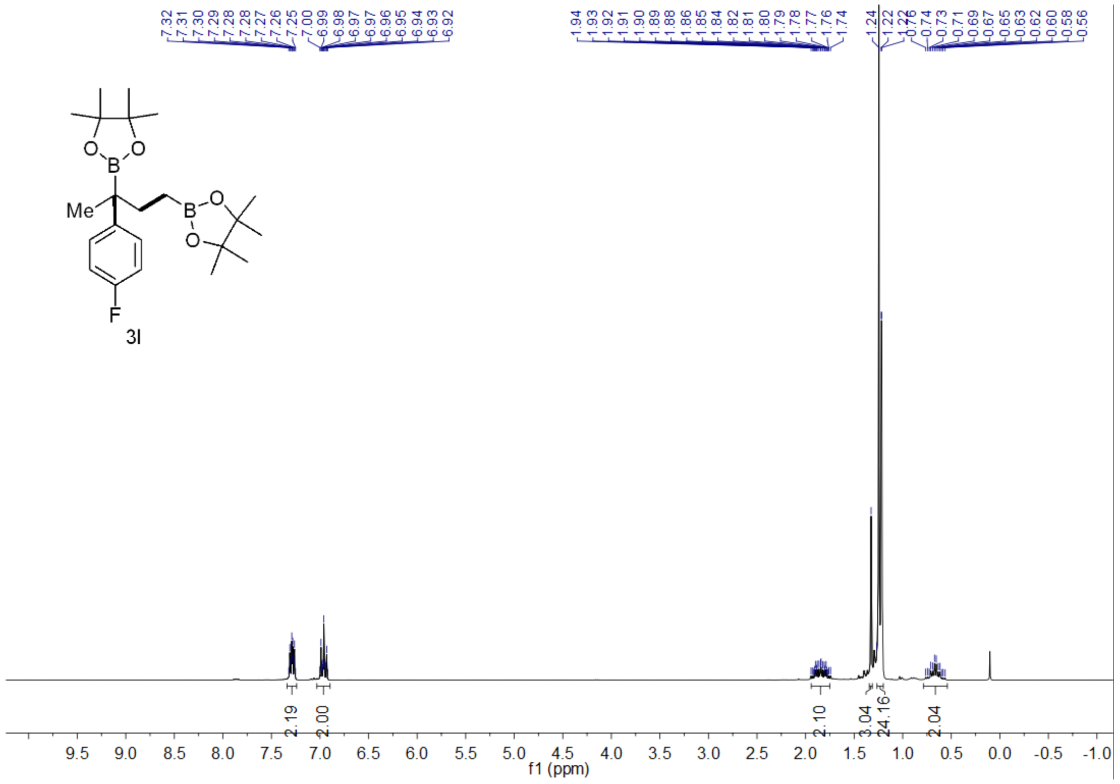
7.32 7.31 7.30 7.29 7.28 7.28 7.27 7.26 7.25 7.00 6.99 6.98 6.97 6.97 6.96 6.95 6.94 6.93 6.92
1.94 1.93 1.92 1.91 1.90 1.89 1.88 1.86 1.85 1.84 1.82 1.81 1.80 1.79 1.78 1.77 1.76 1.74
1.24 1.22 0.76 0.74 0.73 0.71 0.69 0.67 0.65 0.63 0.62 0.60 0.58 0.56
Me
B
O
F
3l
2.19 2.00 2.10 3.04 24.16 2.04
9.5 9.0 8.5 8.0 7.5 7.0 6.5 6.0 5.5 5.0 4.5 4.0 3.5 3.0 2.5 2.0 1.5 1.0 0.5 0.0 -0.5 -1.0
f1 (ppm)

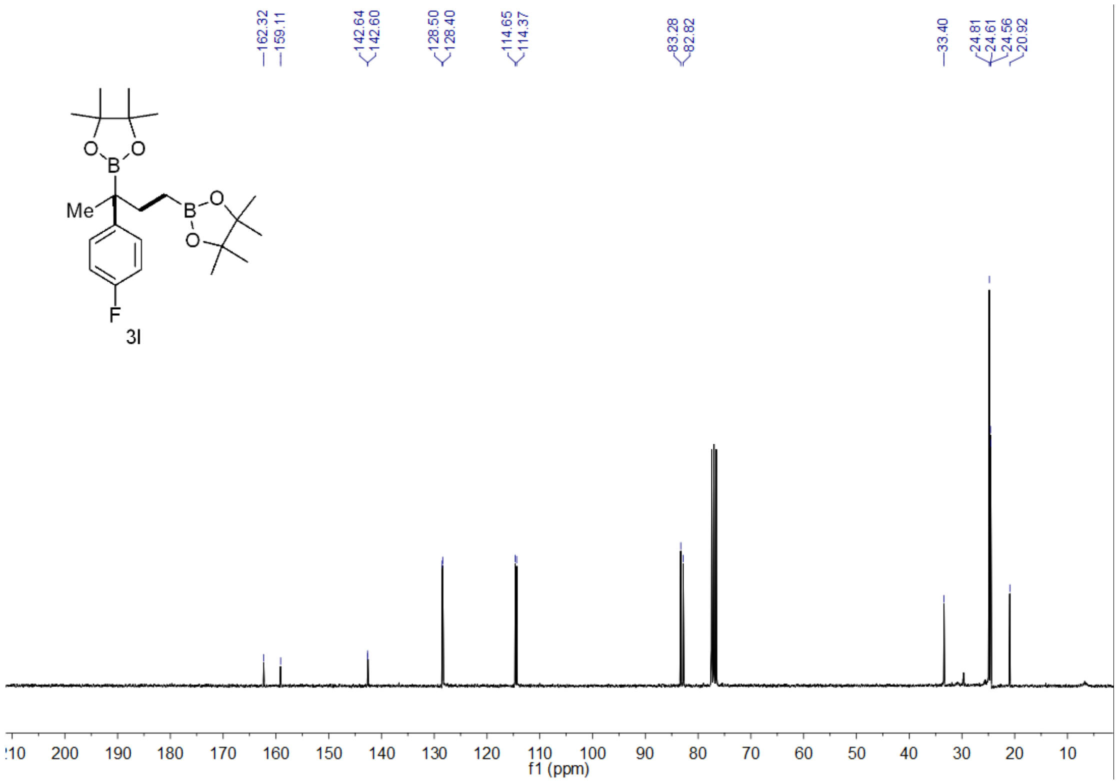
162.32 159.11 142.64 142.60 128.50 128.40 114.65 114.37 83.28 82.82 33.40 24.81 24.61 24.56 20.92
Me
B
O
F
3l
210 200 190 180 170 160 150 140 130 120 110 100 90 80 70 60 50 40 30 20 10
f1 (ppm)

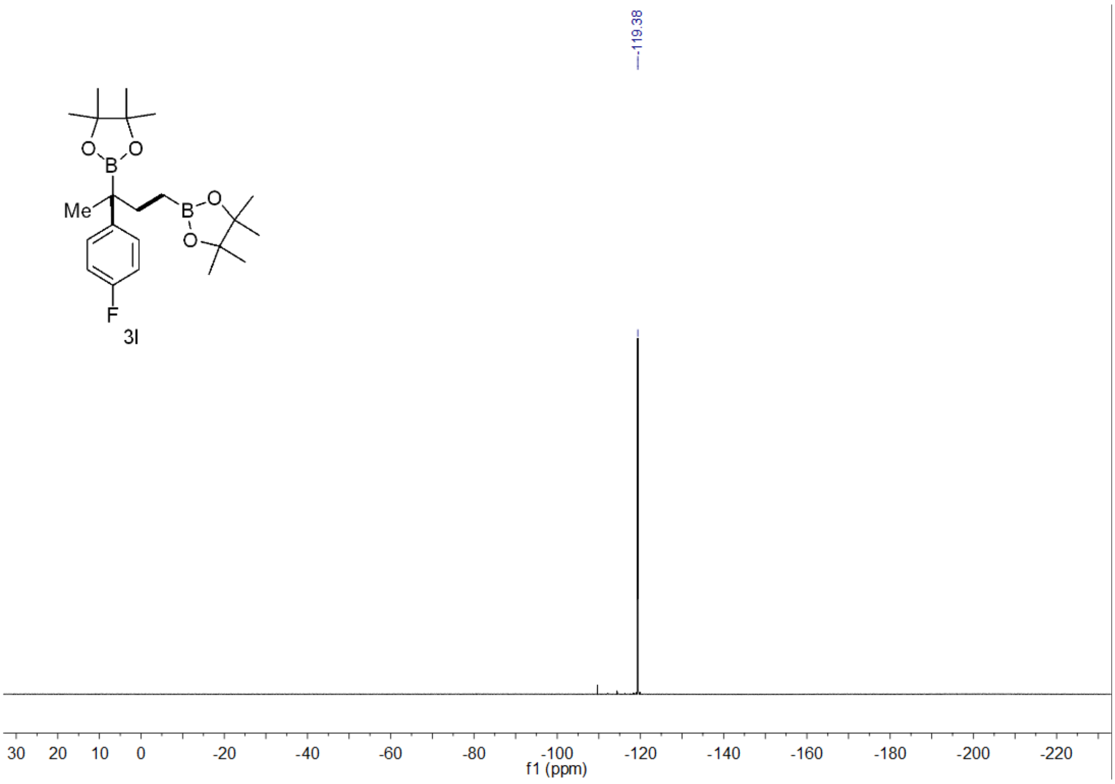

—-119.38
Me
O
B
F
3l
30 20 10 0 -20 -40 -60 -80 -100 -120 -140 -160 -180 -200 -220
f1 (ppm)

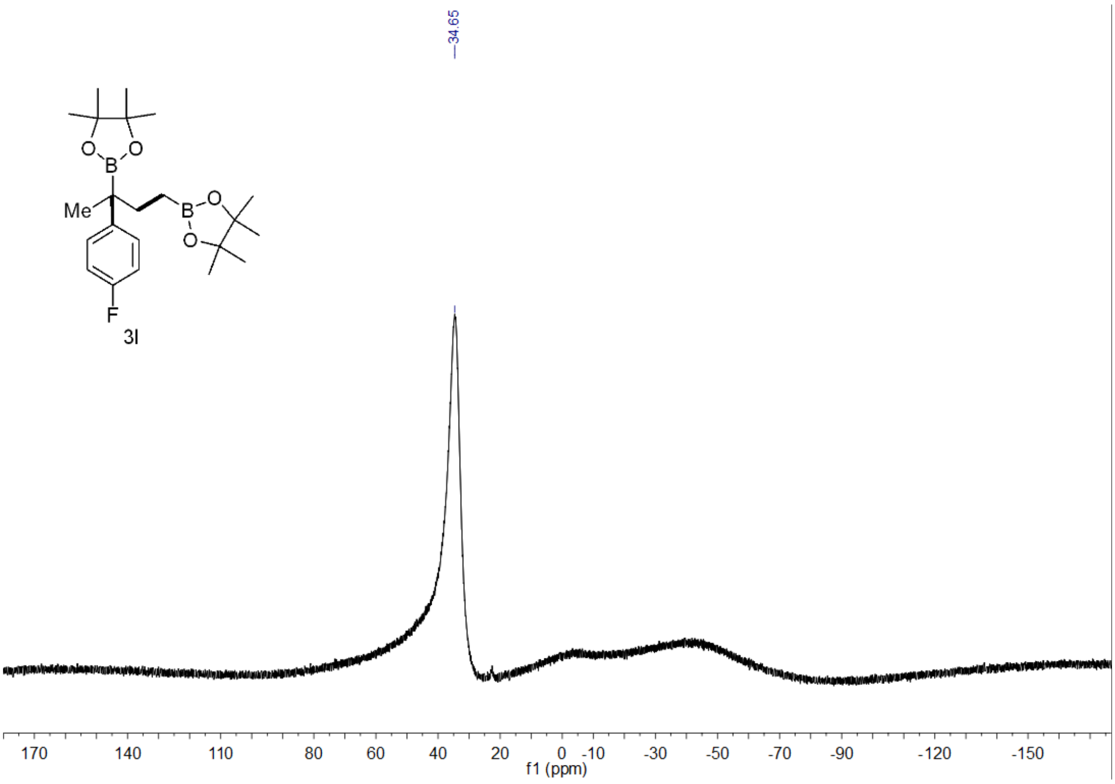

—34.65
Me
O
B
F
3l
170 140 110 80 60 40 20 0 -10 -30 -50 -70 -90 -120 -150
f1 (ppm)

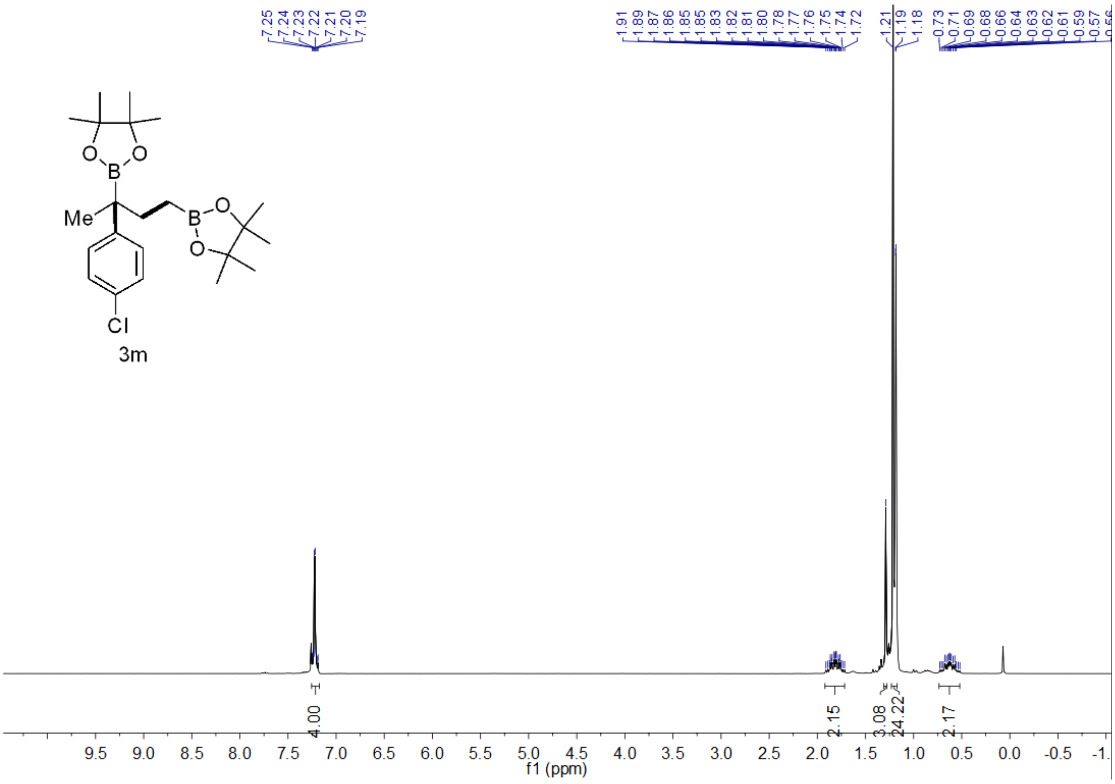
7.25 7.24 7.23 7.22 7.21 7.20 7.19
1.91 1.89 1.87 1.86 1.85 1.85 1.83 1.82 1.81 1.80 1.78 1.77 1.76 1.75 1.74 1.72
1.21 1.19 1.18
0.73 0.71 0.69 0.68 0.66 0.64 0.63 0.62 0.61 0.59 0.57
Me
B
O
Cl
3m
4.00
2.15
3.08
24.22
2.17
f1 (ppm)

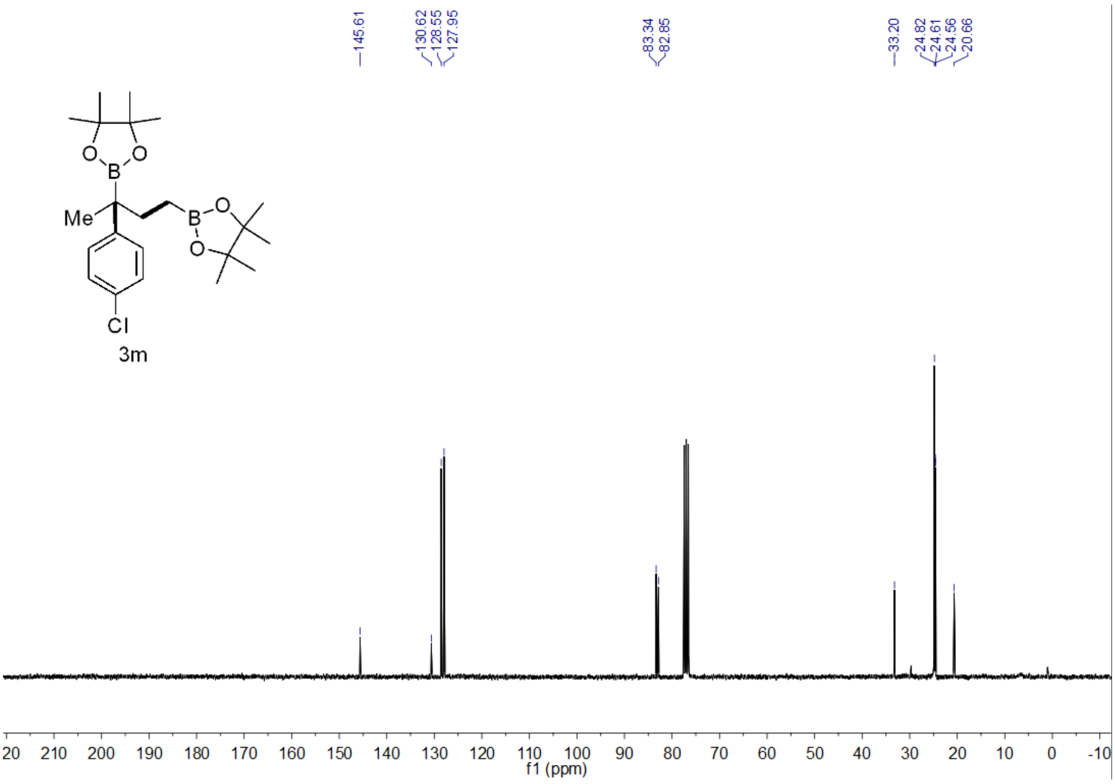
145.61
130.62 128.55 127.95
83.34 82.85
33.20
24.82 24.61 24.56 20.66
Me
B
O
Cl
3m
f1 (ppm)

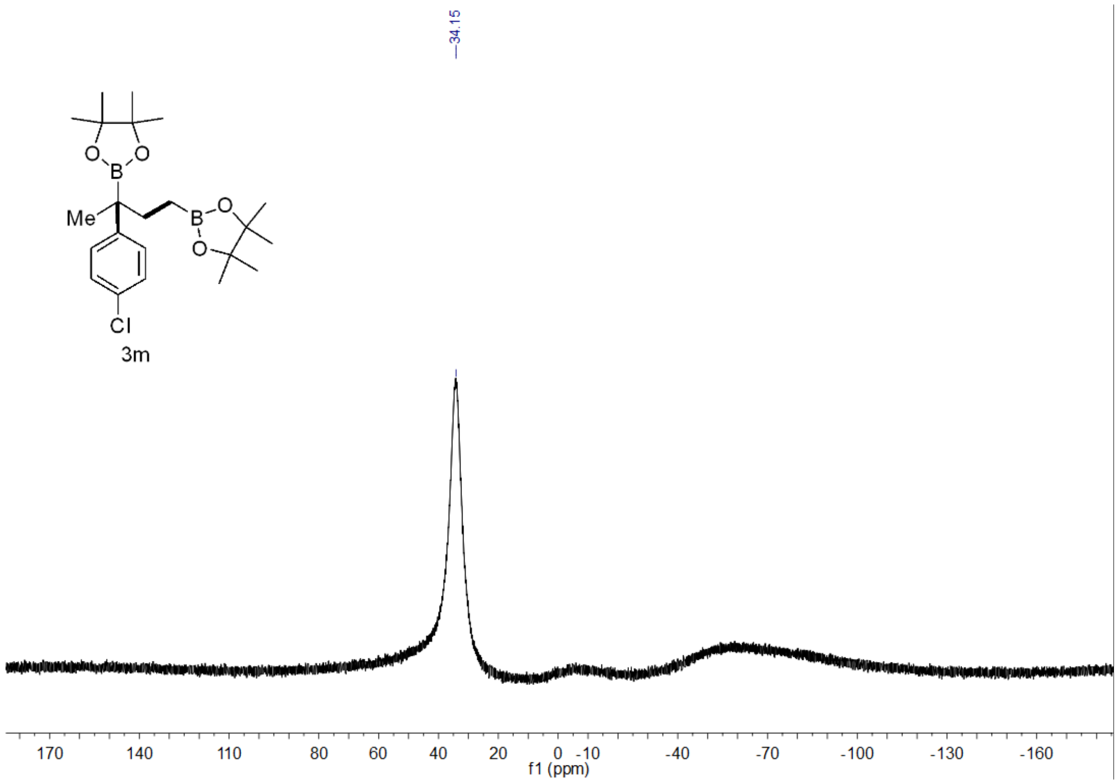

34.15
Me
Cl
3m
170 140 110 80 60 40 20 0 -10 -40 -70 -100 -130 -160
f1 (ppm)

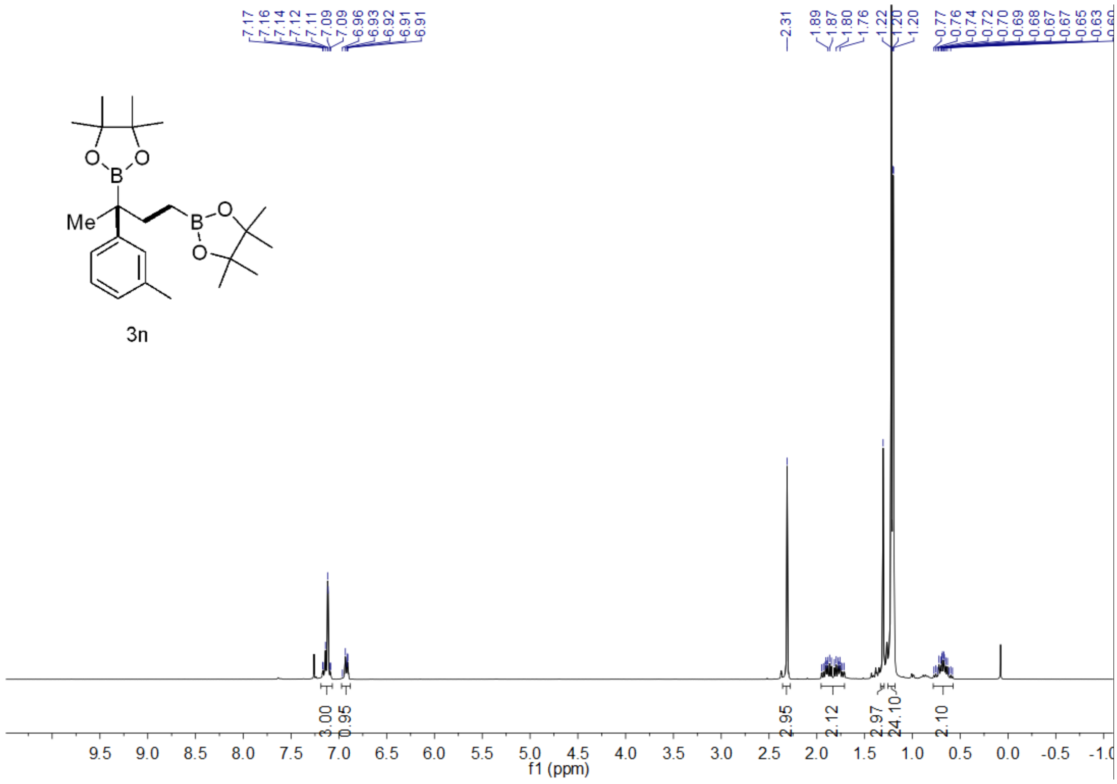

7.17 7.16 7.14 7.12 7.11 7.09 7.09 6.96 6.93 6.92 6.91 6.91
2.31
1.89 1.87 1.80 1.76
1.22 1.20 1.20
0.77 0.76 0.74 0.72 0.70 0.69 0.68 0.67 0.65 0.63
Me
3n
3.00 0.95 2.95 2.12 2.97 24.10 2.10
9.5 9.0 8.5 8.0 7.5 7.0 6.5 6.0 5.5 5.0 4.5 4.0 3.5 3.0 2.5 2.0 1.5 1.0 0.5 0.0 -0.5
f1 (ppm)

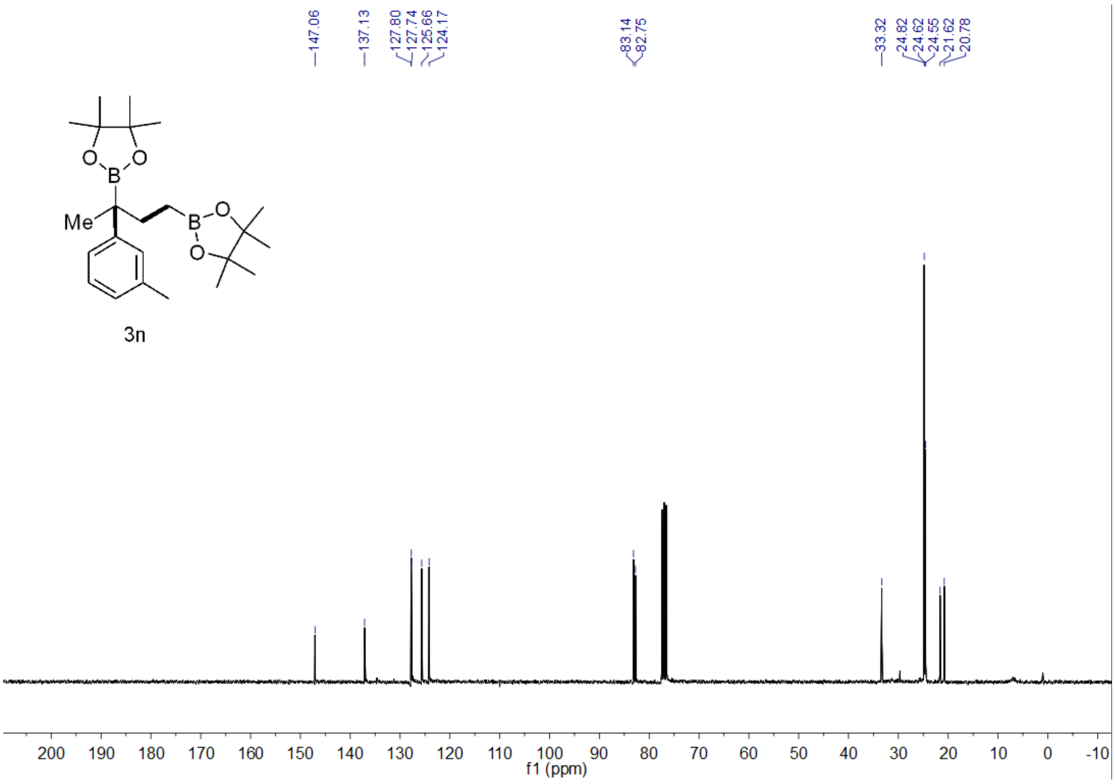
147.06
137.13
127.80
127.74
125.66
124.17
83.14
82.75
33.32
24.82
24.62
24.55
21.62
20.78
3n
f1 (ppm)

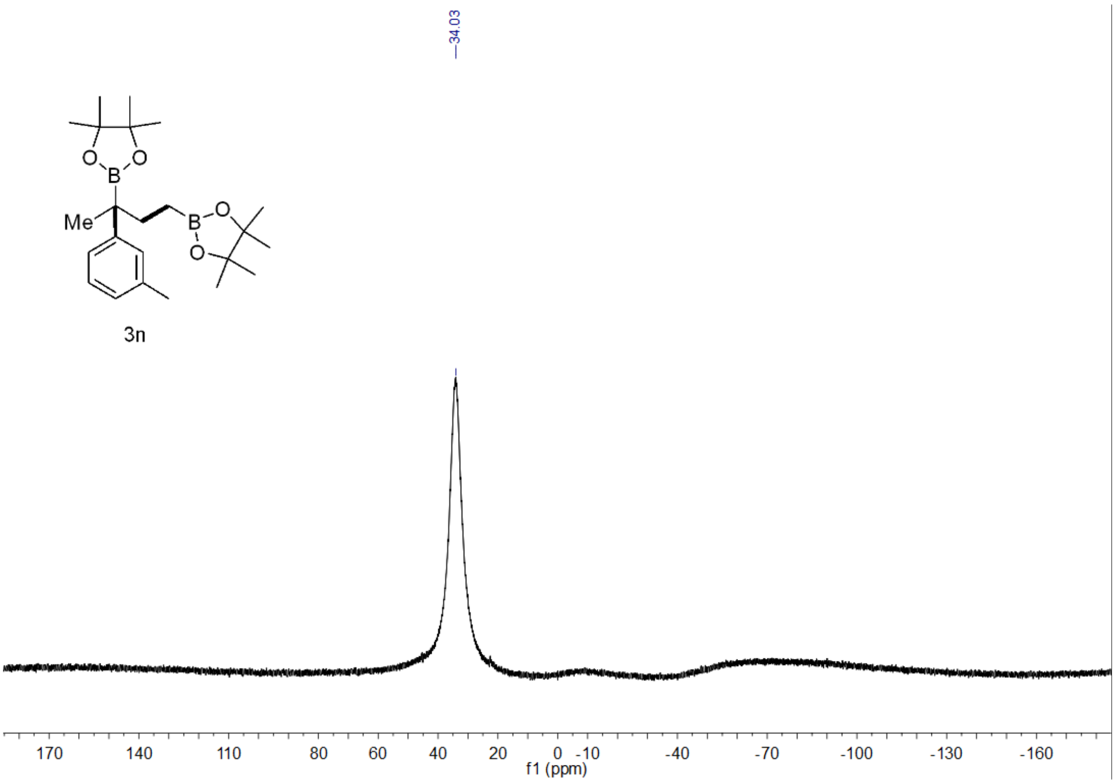
34.03
3n
f1 (ppm)

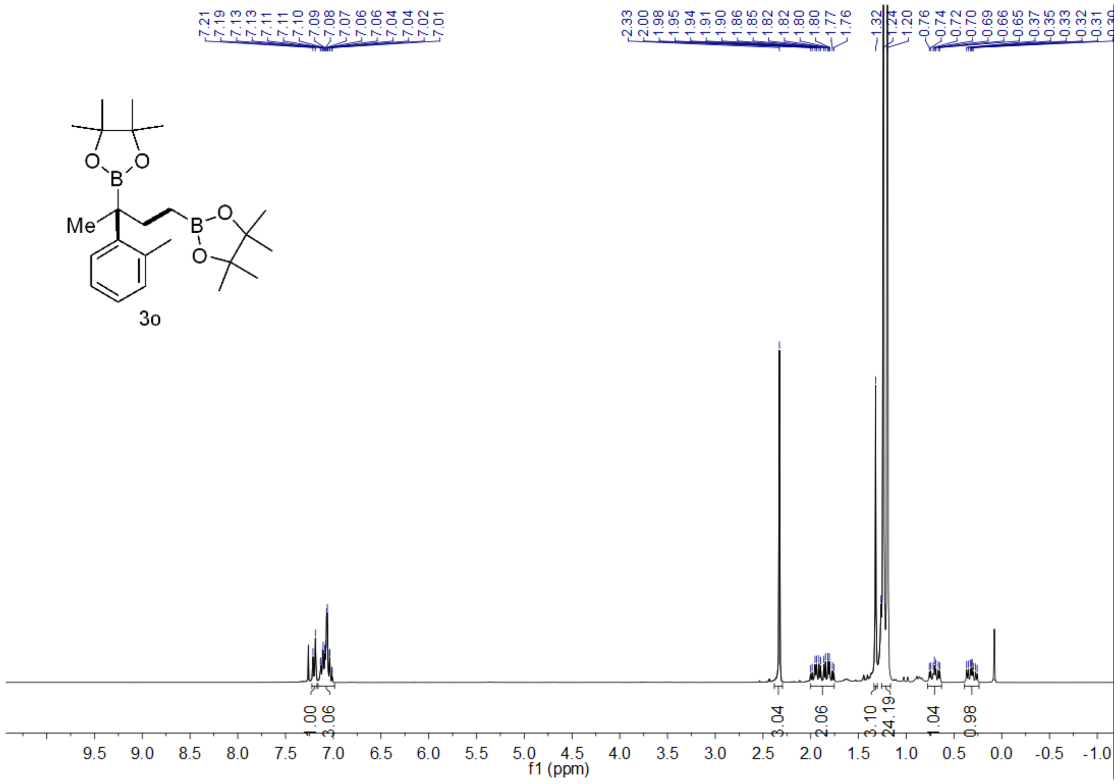
7.21
7.19
7.13
7.13
7.11
7.11
7.10
7.09
7.08
7.07
7.06
7.06
7.04
7.04
7.02
7.01
2.33
2.00
1.98
1.95
1.94
1.91
1.90
1.86
1.85
1.82
1.82
1.80
1.80
1.77
1.76
1.32
1.24
1.20
0.76
0.74
0.72
0.70
0.69
0.66
0.65
0.37
0.35
0.33
0.32
0.31
Me
B
O
3o
1.00
3.06
3.04
2.06
3.10
24.19
1.04
0.98
f1 (ppm)

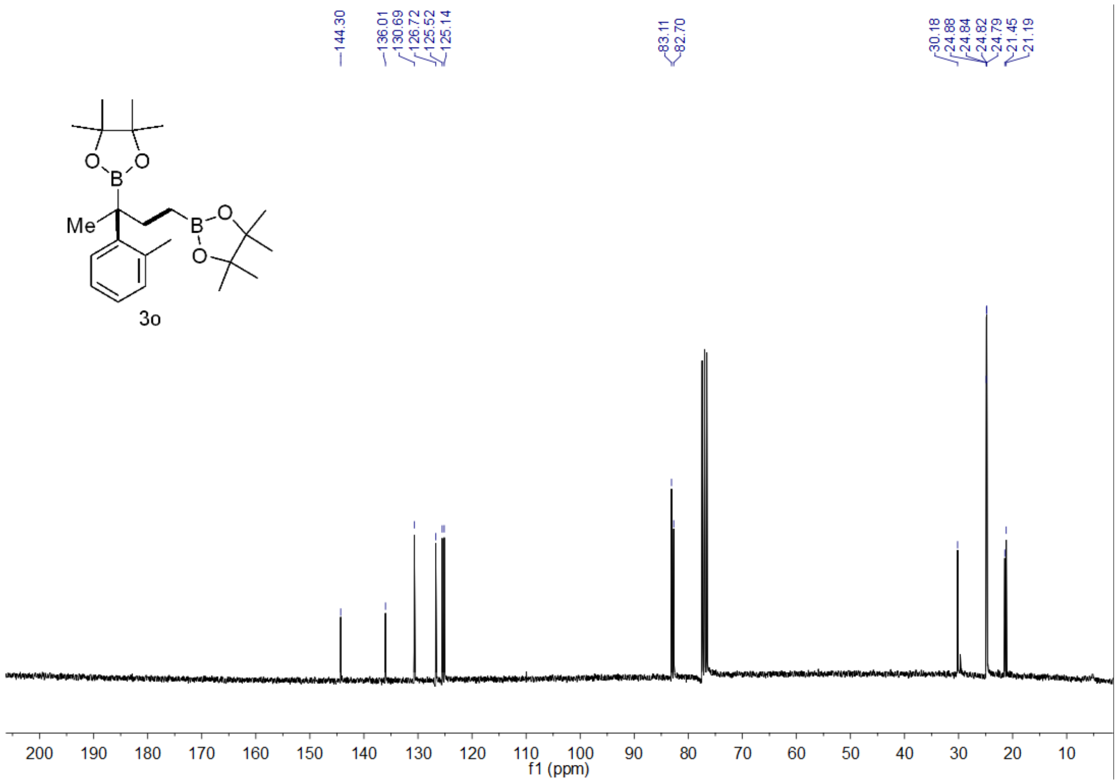
144.30
136.01
130.69
126.72
125.52
125.14
83.11
82.70
30.18
24.88
24.84
24.82
24.79
21.45
21.19
Me
B
O
3o
f1 (ppm)

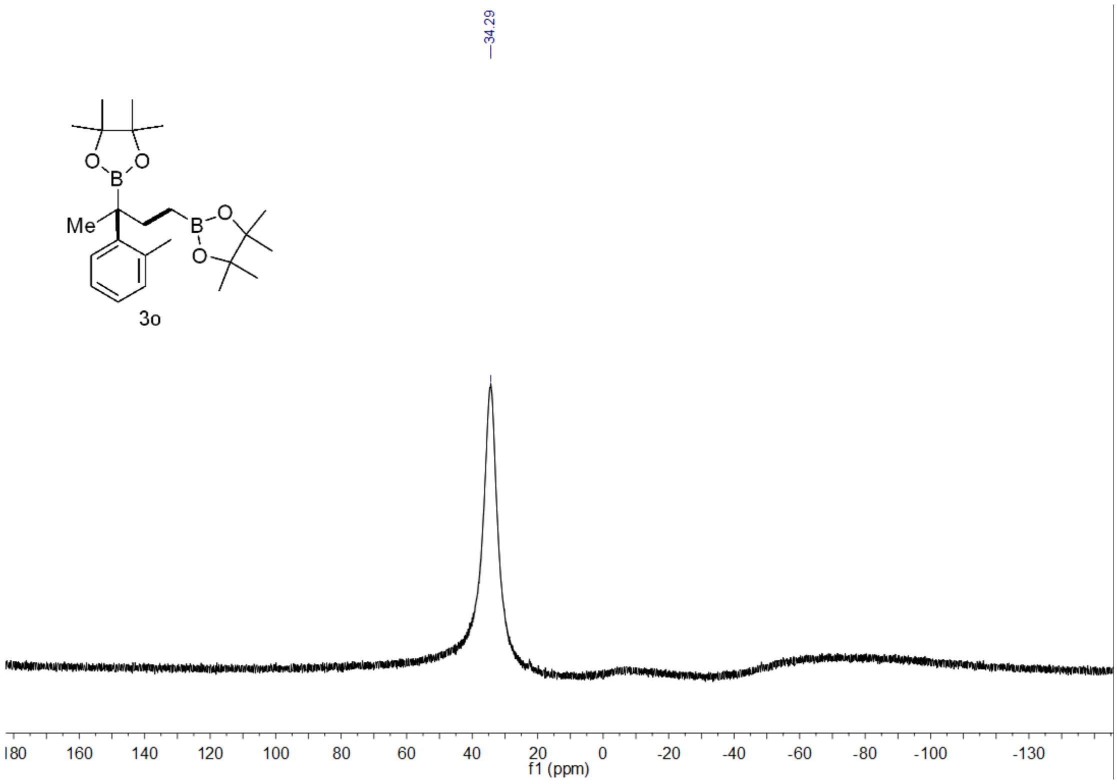

34.29
Me
B
O
3o
180
160
140
120
100
80
60
40
20
0
-20
-40
-60
-80
-100
-130
f1 (ppm)

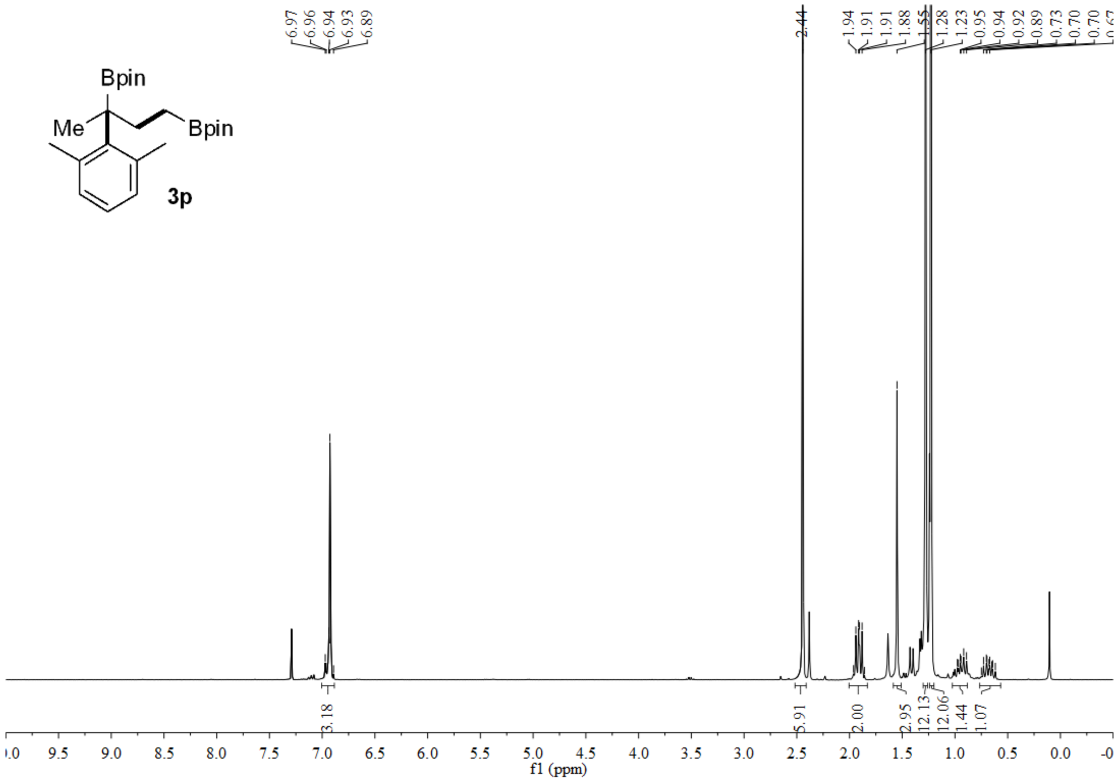

6.97
6.96
6.94
6.93
6.89
2.44
1.94
1.91
1.91
1.88
1.55
1.28
1.23
0.95
0.94
0.92
0.89
0.73
0.70
Bpin
Me
Bpin
3p
3.18
5.91
2.00
2.95
12.13
12.06
1.44
1.07
9.5
9.0
8.5
8.0
7.5
7.0
6.5
6.0
5.5
5.0
4.5
4.0
3.5
3.0
2.5
2.0
1.5
1.0
0.5
0.0
f1 (ppm)

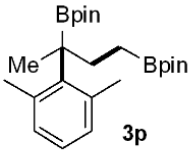
Bpin
Me
Bpin

3p

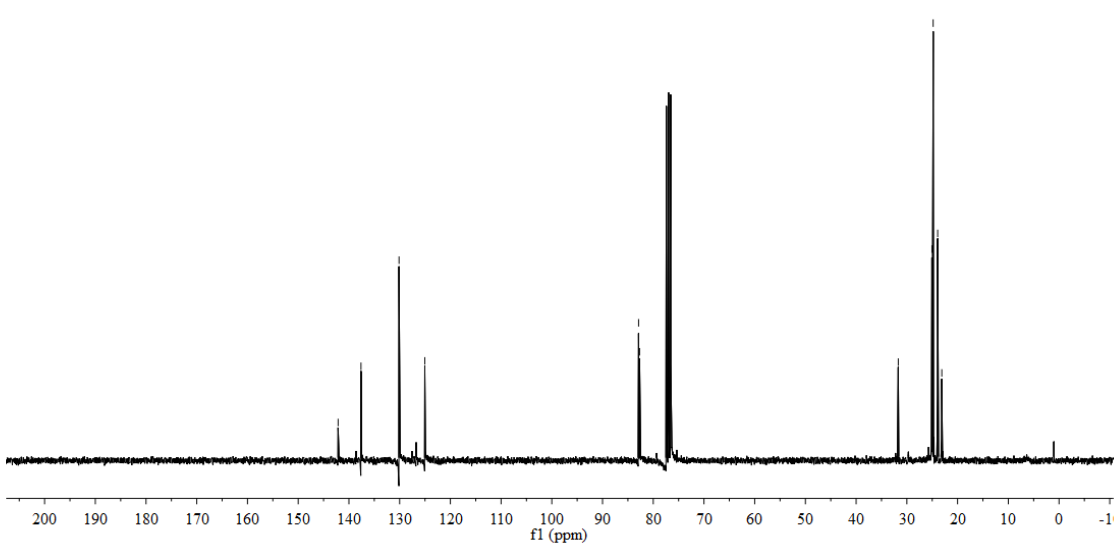
142.13
137.62
130.12
125.04
82.89
82.69
31.69
25.06
24.85
24.81
23.92
23.11
f1 (ppm)

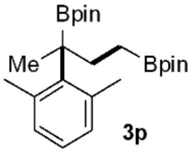
Bpin
Me
Bpin

3p

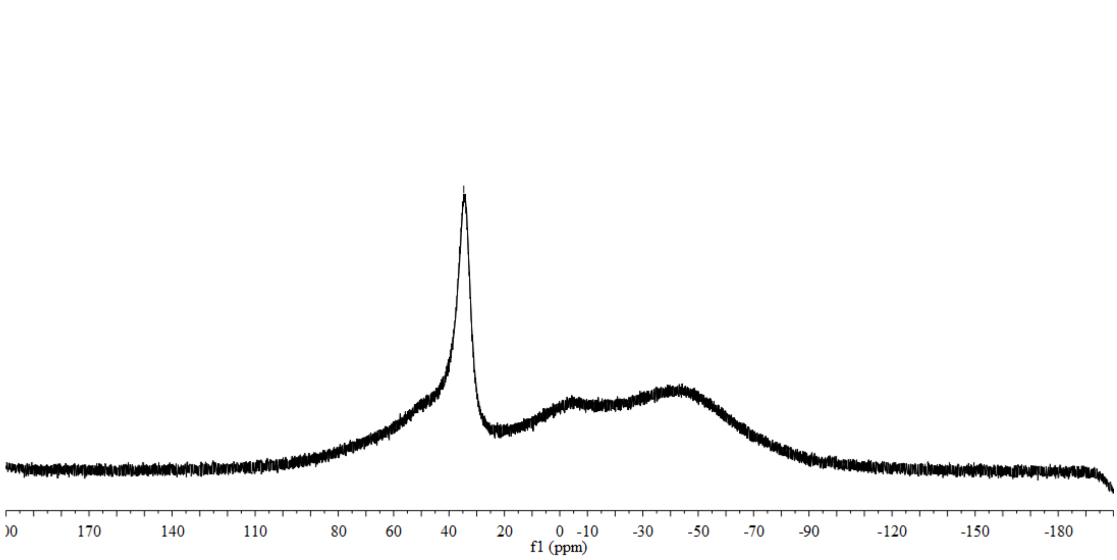
34.68
f1 (ppm)

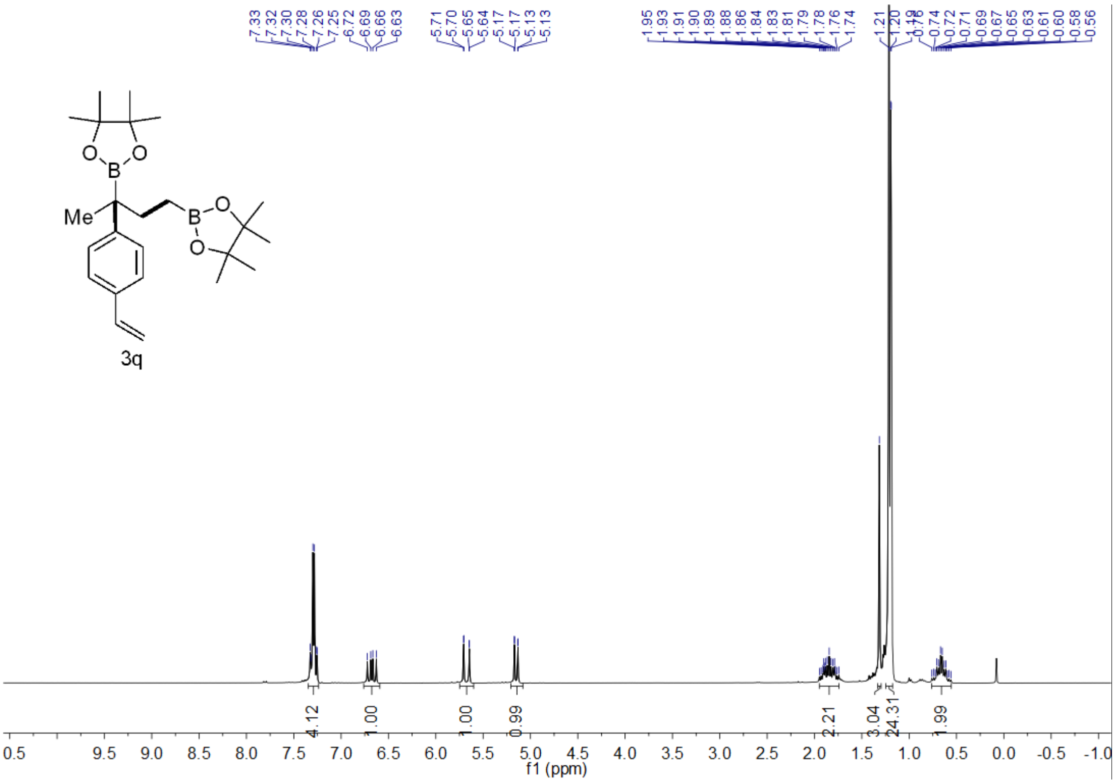
7.33 7.32 7.30 7.28 7.26 7.25 6.72 6.69 6.66 6.63
5.71 5.70 5.65 5.64 5.17 5.17 5.13 5.13
1.95 1.93 1.91 1.90 1.89 1.88 1.86 1.84 1.83 1.81 1.79 1.78 1.76 1.74
1.21 1.20
0.74 0.72 0.71 0.69 0.67 0.65 0.63 0.61 0.60 0.58 0.56
O B O
Me
B O O
3q
4.12 1.00 1.00 0.99 2.21 3.04 24.31 1.99
f1 (ppm)

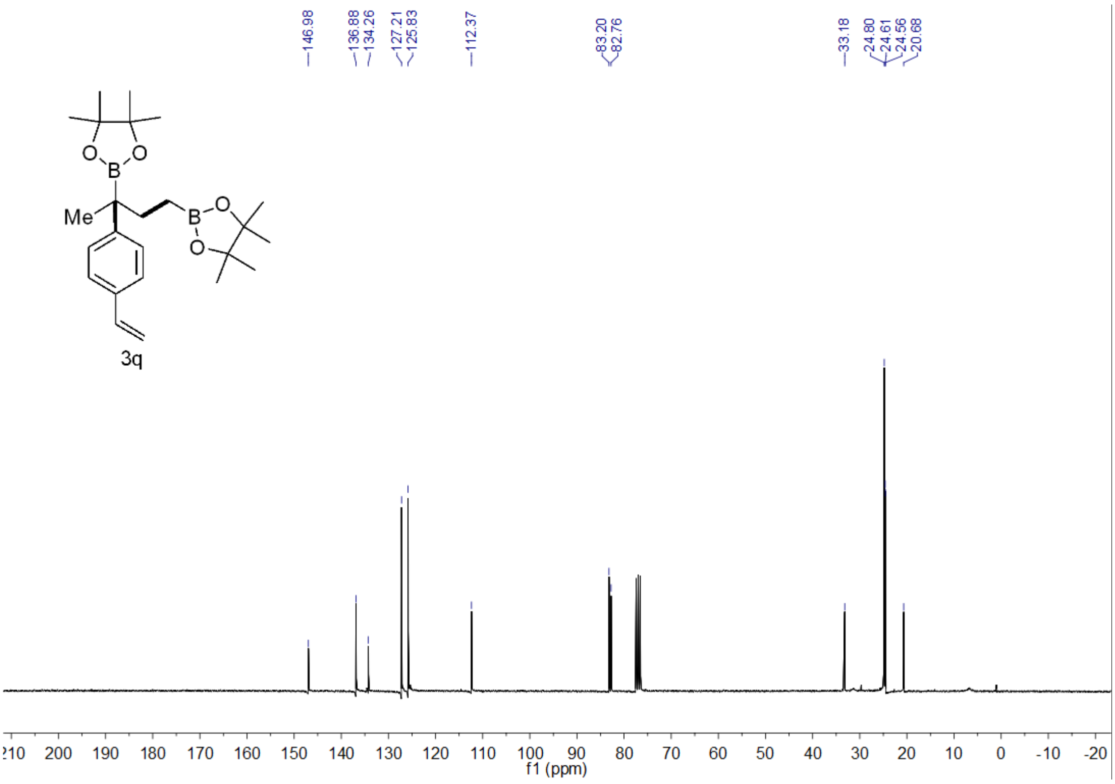
146.98
136.88 134.26 127.21 125.83
112.37
83.20 82.76
33.18
24.80 24.61 24.56 20.68
O B O
Me
B O O
3q
f1 (ppm)

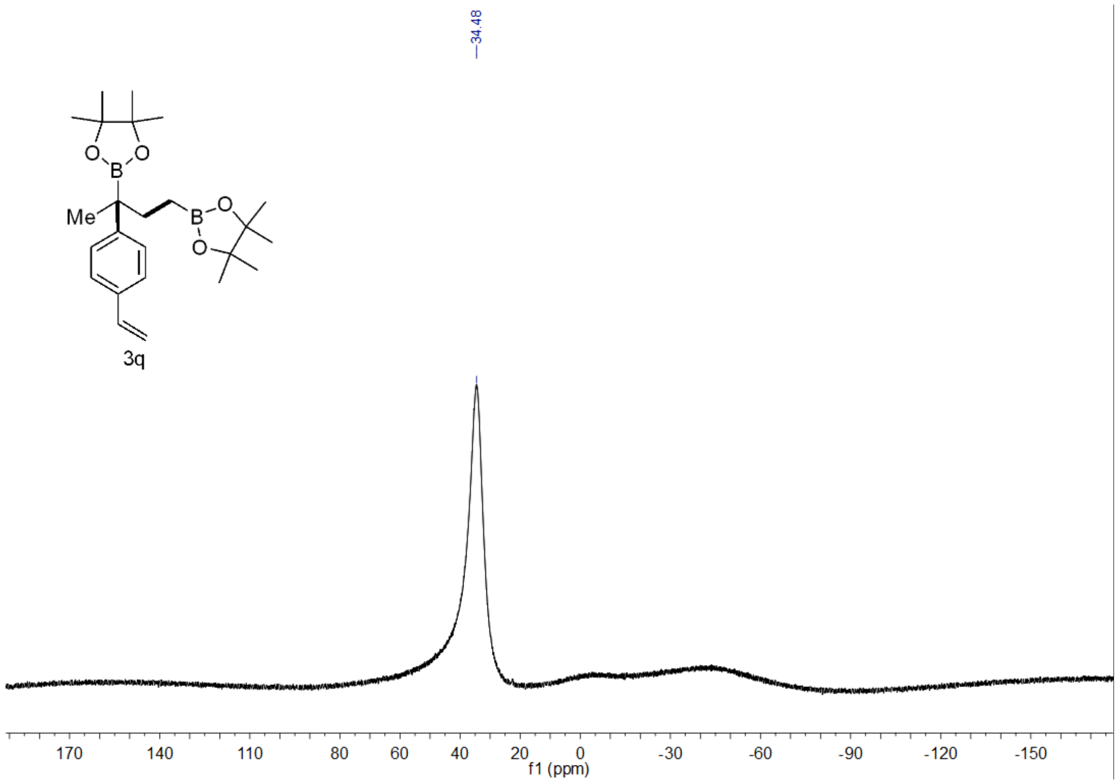

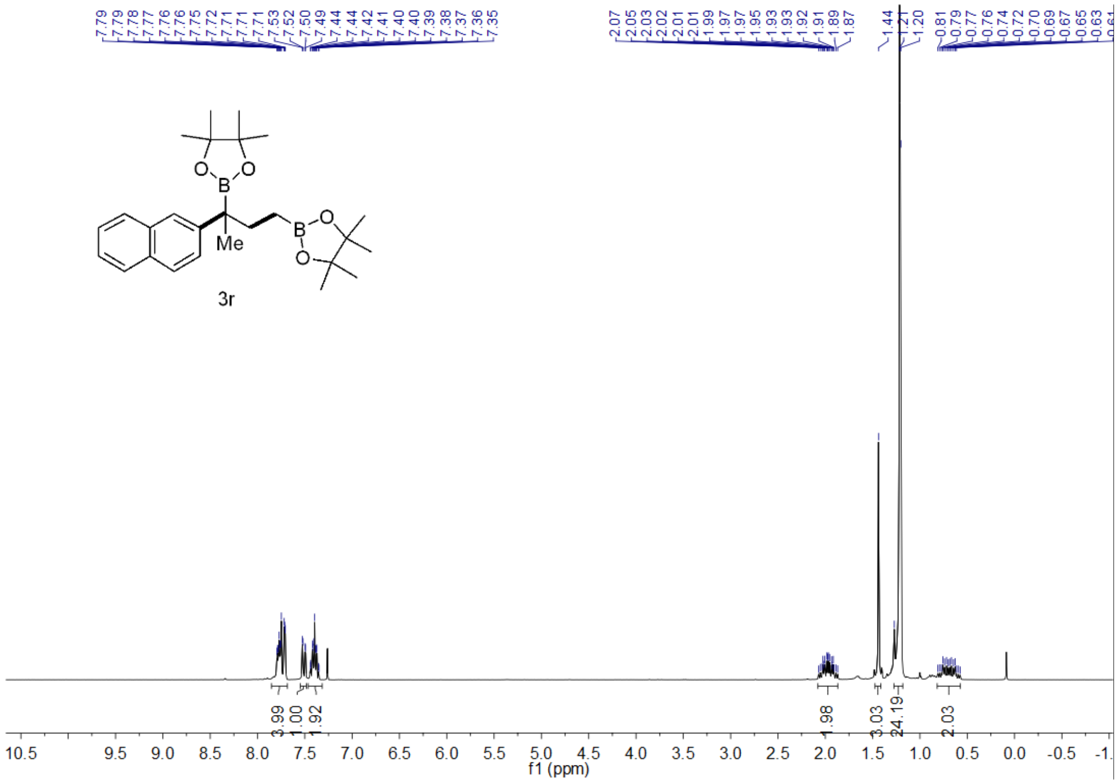

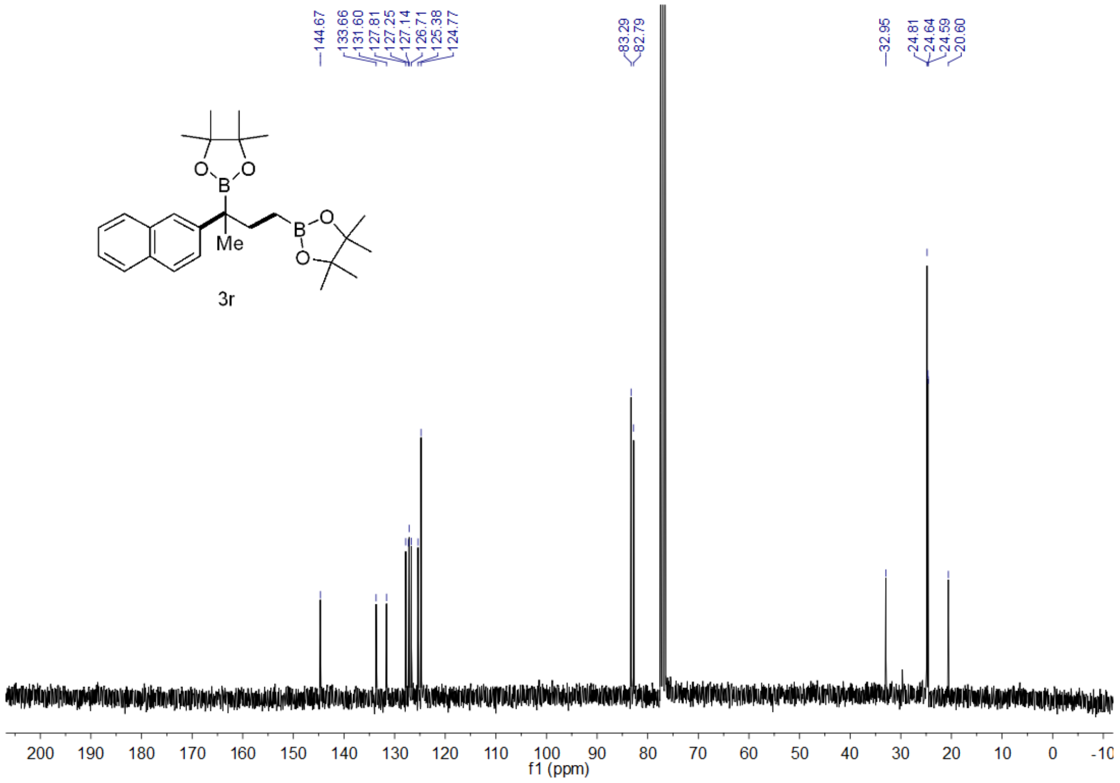
144.67
133.66
131.60
127.81
127.25
127.14
126.71
125.38
124.77
83.29
82.79
32.95
24.81
24.64
24.59
20.60
O
B
O
Me
B
O
O
3r
200
190
180
170
160
150
140
130
120
110
100
90
80
70
60
50
40
30
20
10
0
-10
f1 (ppm)

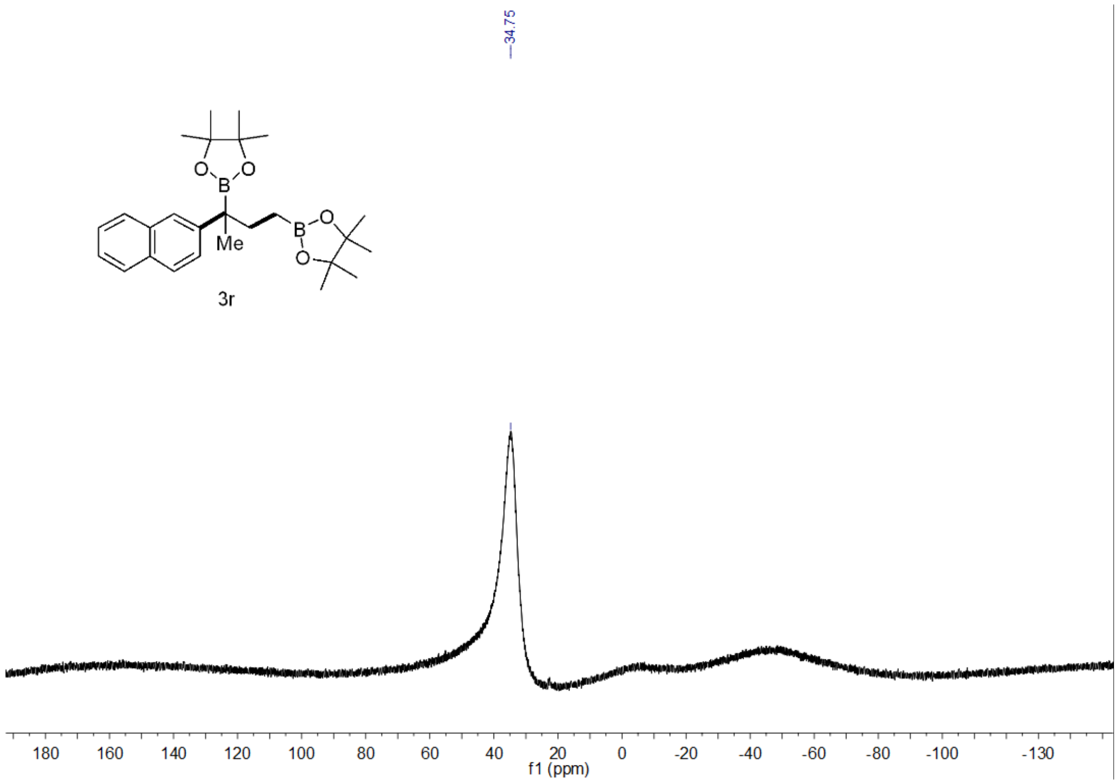
34.75
O
B
O
Me
B
O
O
3r
180
160
140
120
100
80
60
40
20
0
-20
-40
-60
-80
-100
-130
f1 (ppm)

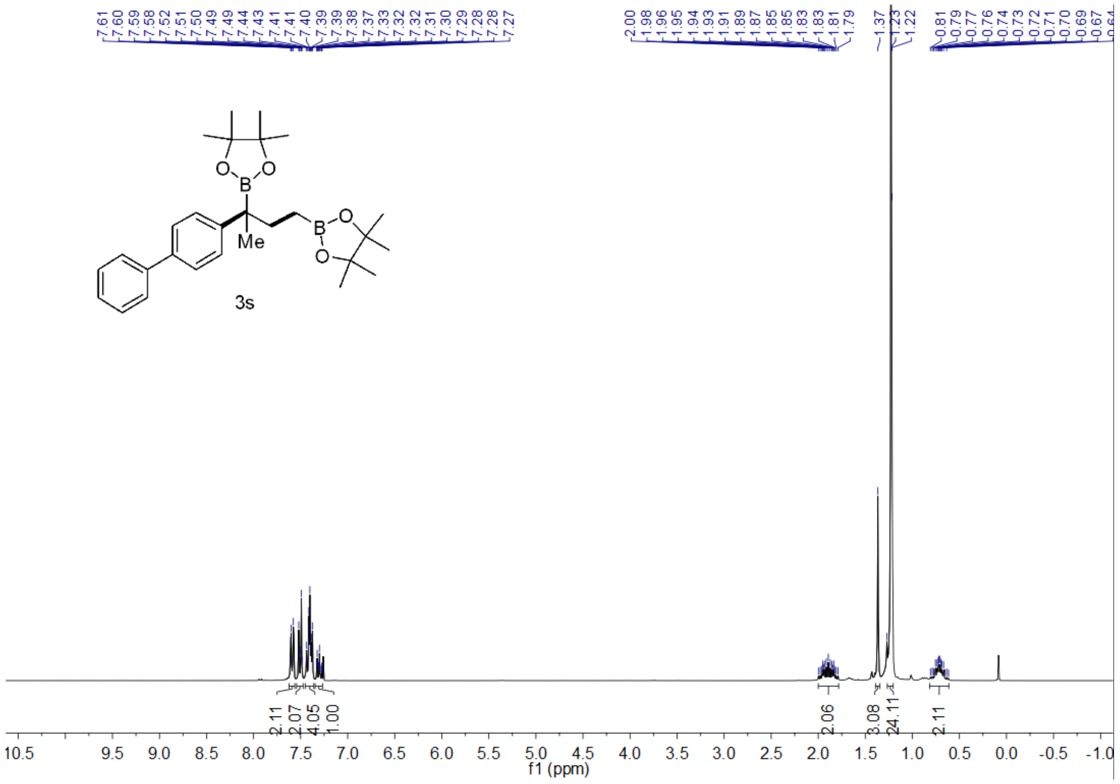
3s
Me
f1 (ppm)

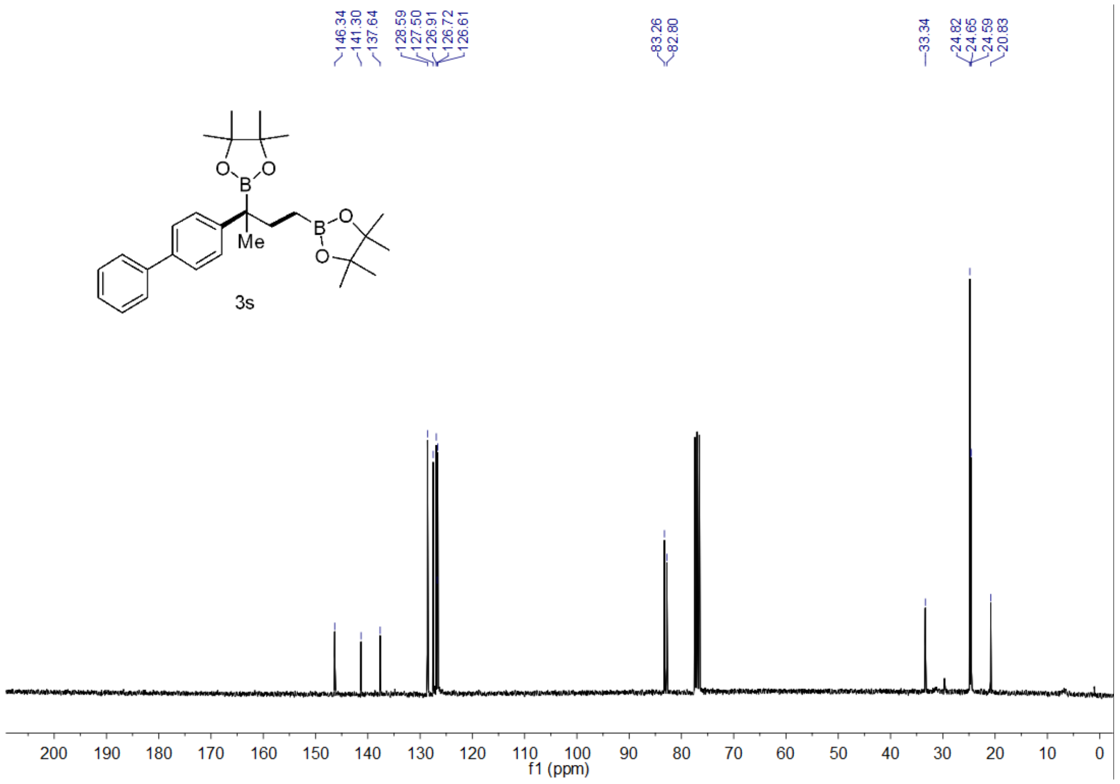
3s
Me
f1 (ppm)

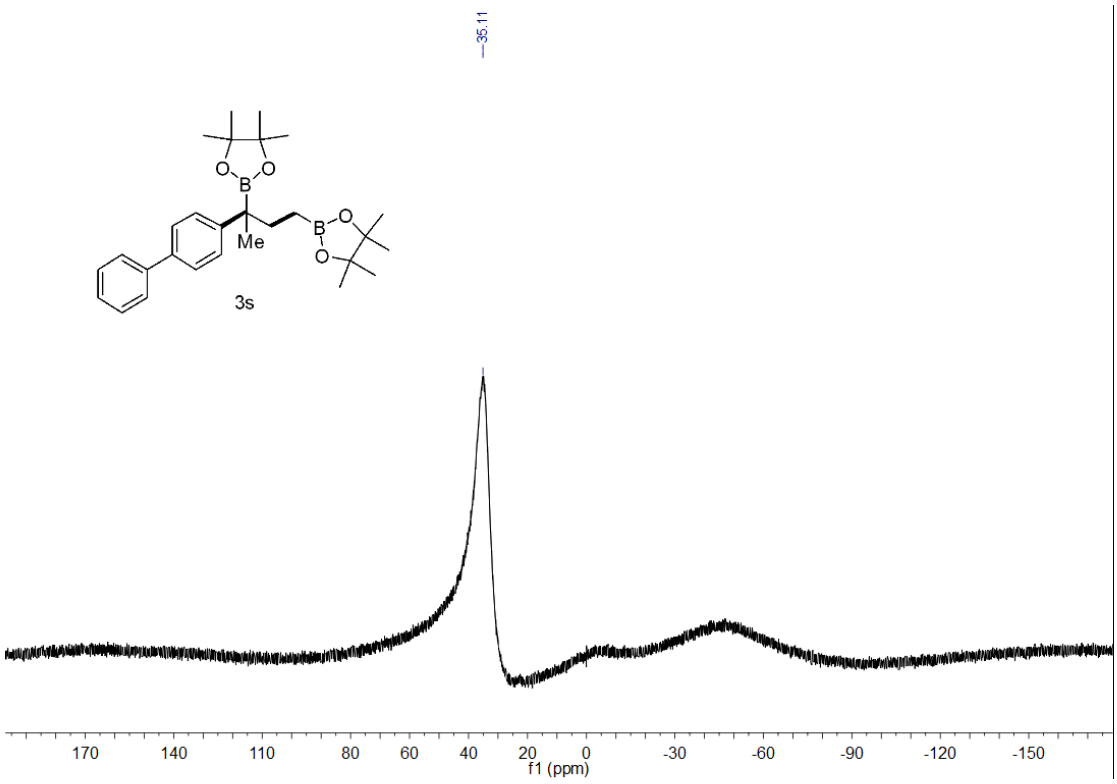
35.11
3s
Me
f1 (ppm)

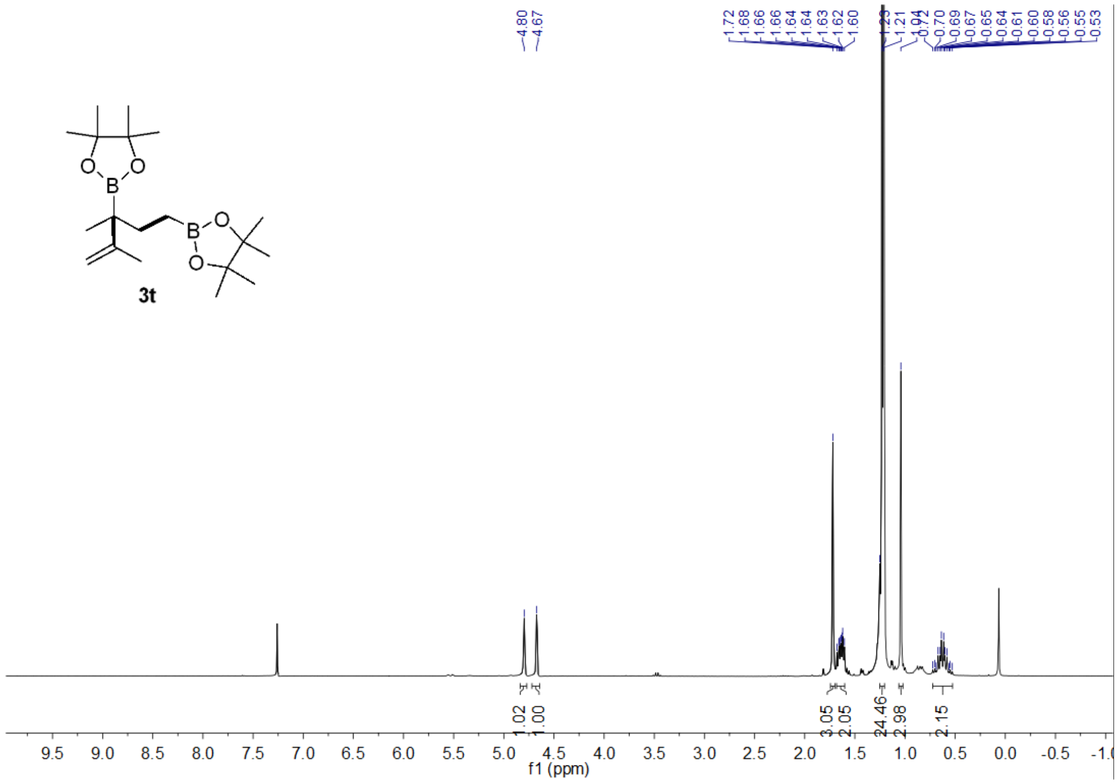
3t
f1 (ppm)

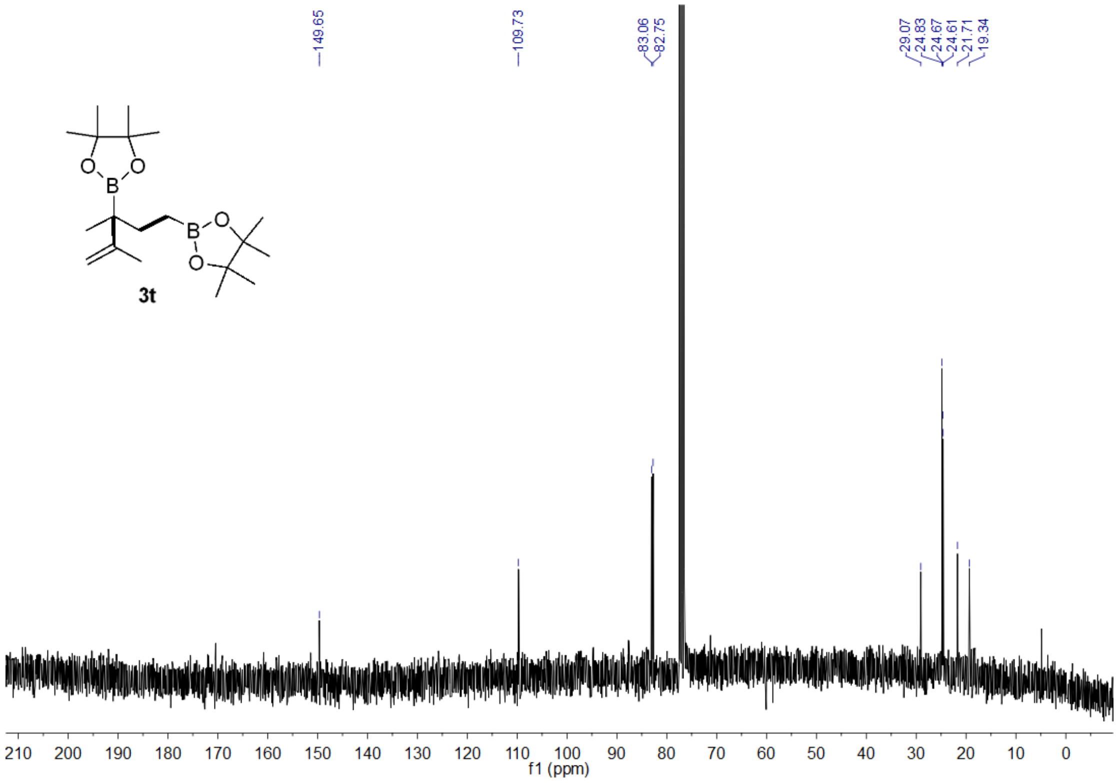

149.65
109.73
83.06
82.75
29.07
24.83
24.67
24.61
21.71
19.34
3t
210 200 190 180 170 160 150 140 130 120 110 100 90 80 70 60 50 40 30 20 10 0
f1 (ppm)

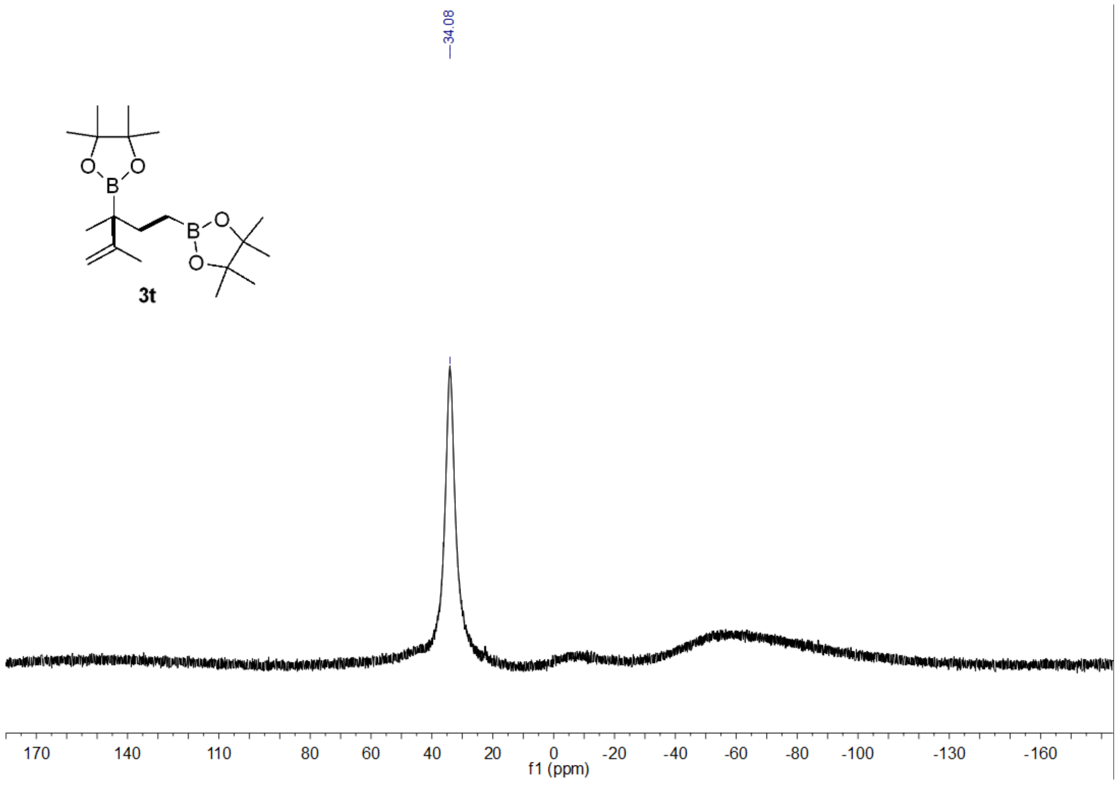

34.08
3t
170 140 110 80 60 40 20 0 -20 -40 -60 -80 -100 -130 -160
f1 (ppm)

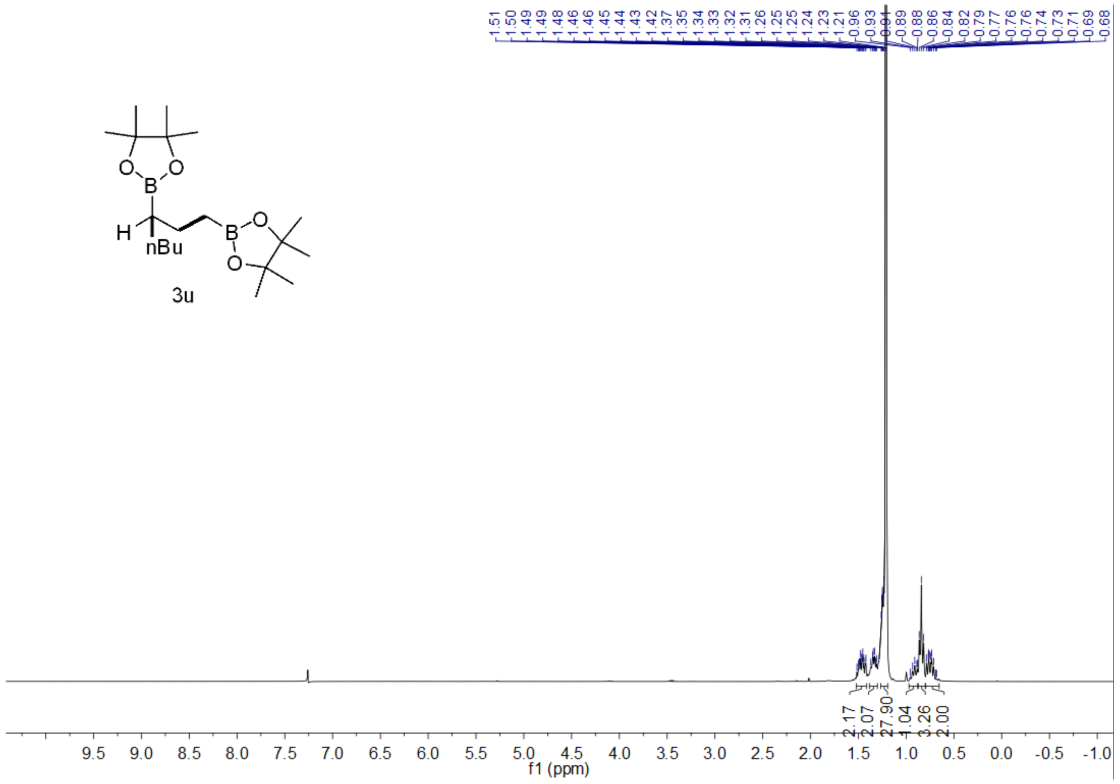
1.51 1.50 1.49 1.48 1.46 1.46 1.45 1.44 1.43 1.42 1.37 1.35 1.34 1.33 1.32 1.31 1.26 1.25 1.25 1.24 1.23 1.21 0.96 0.93 0.91 0.89 0.88 0.86 0.84 0.82 0.79 0.77 0.76 0.76 0.74 0.73 0.71 0.69 0.68
H
nBu
3u
2.17
2.07
27.90
1.04
3.26
2.00
f1 (ppm)

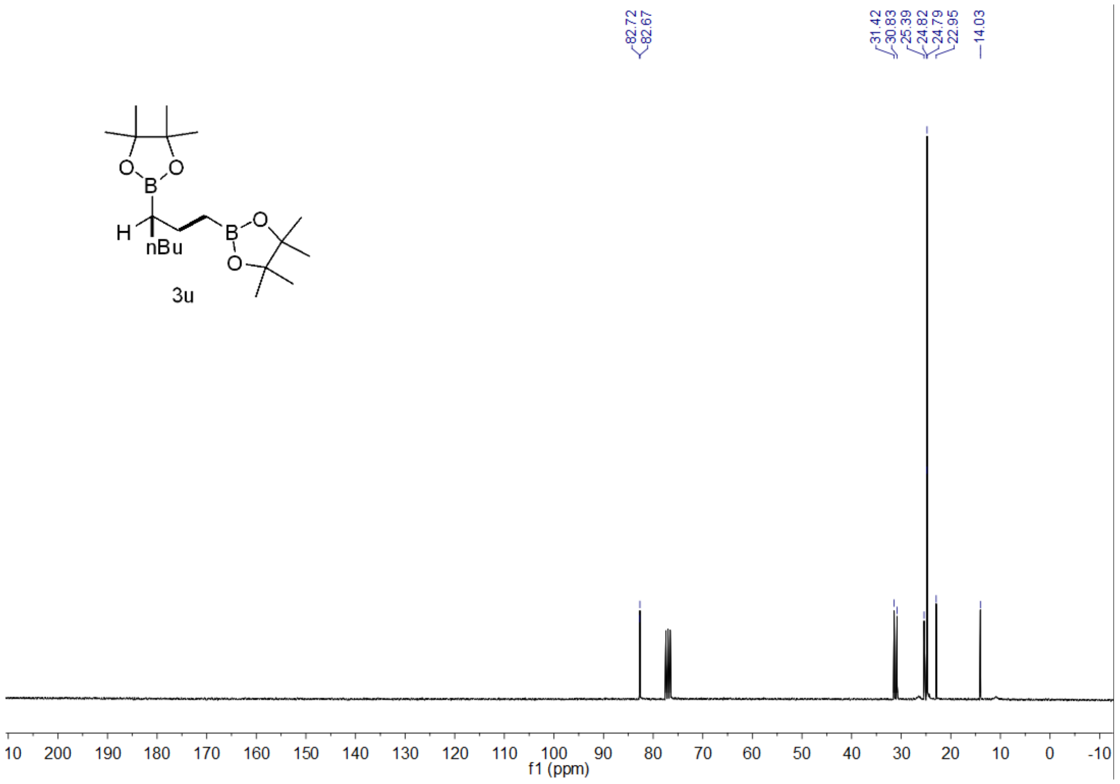
82.72
82.67
31.42
30.83
25.39
24.82
24.79
22.95
14.03
H
nBu
3u
f1 (ppm)

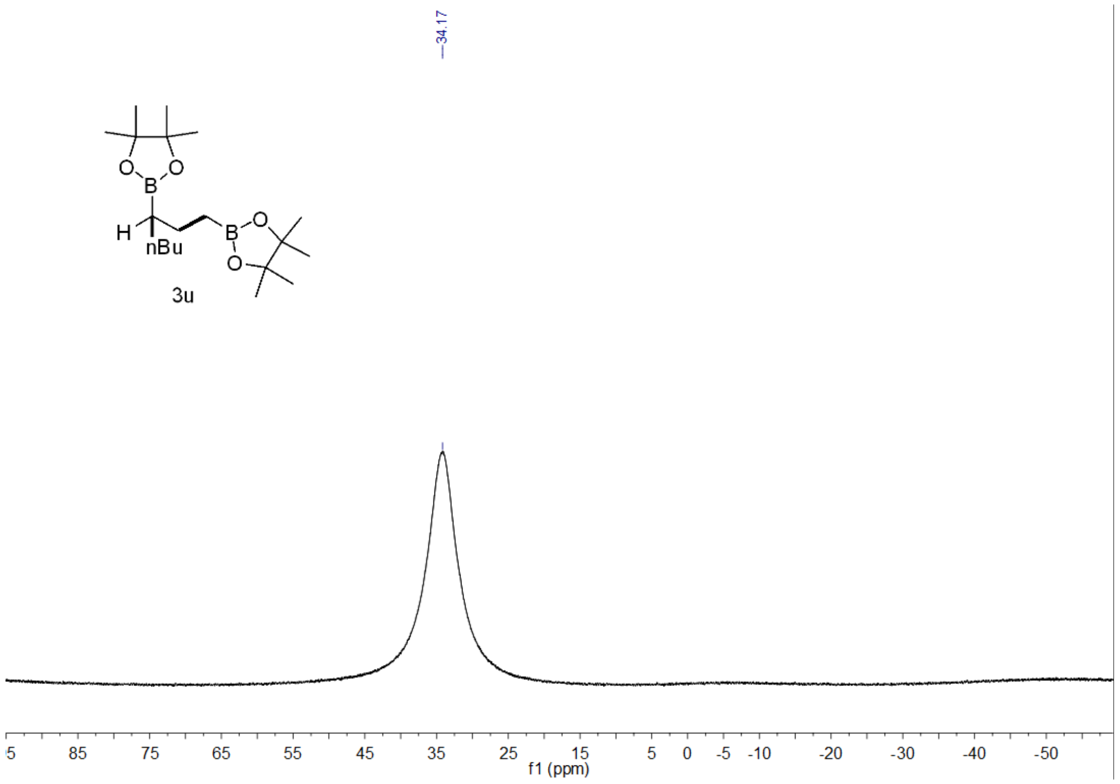

34.17
H
nBu
3u
5 85 75 65 55 45 35 25 15 5 0 -5 -10 -20 -30 -40 -50
f1 (ppm)

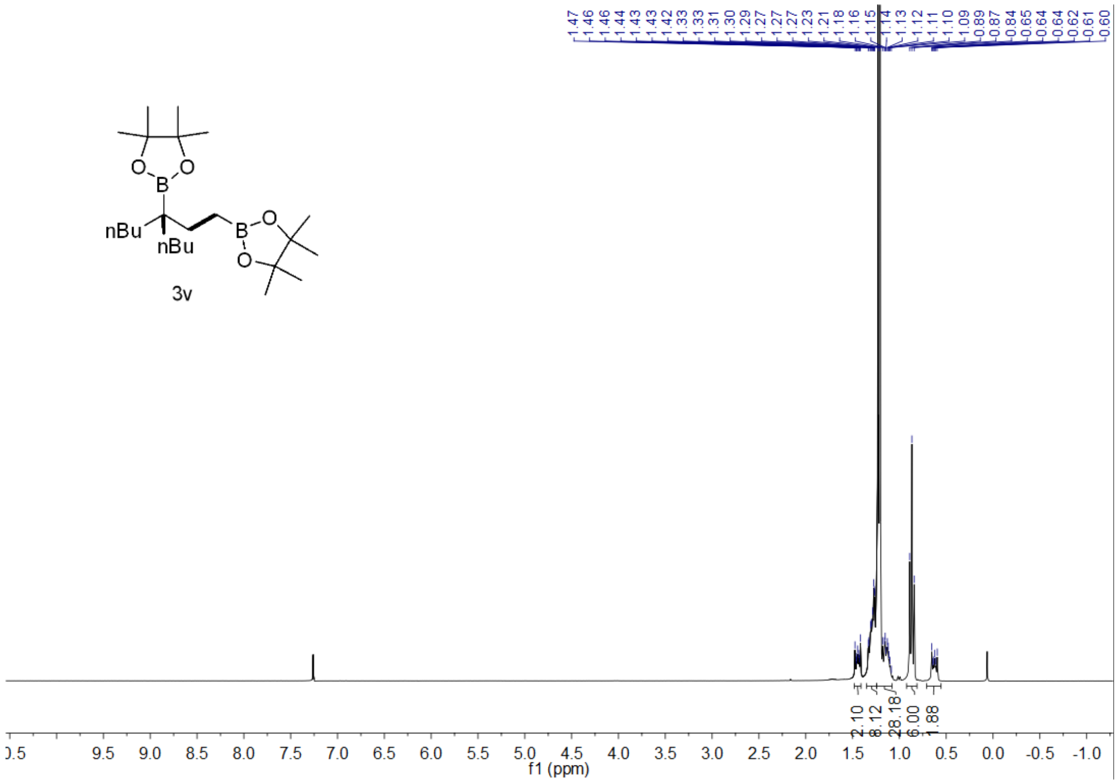

1.47 1.46 1.44 1.43 1.43 1.42 1.33 1.33 1.31 1.30 1.29 1.27 1.27 1.27 1.23 1.21 1.18 1.16 1.15 1.14 1.13 1.12 1.11 1.10 1.09 0.89 0.87 0.84 0.65 0.64 0.64 0.62 0.61 0.60
nBu
nBu
3v
2.10 8.12 28.18 6.00 1.88
.5 9.5 9.0 8.5 8.0 7.5 7.0 6.5 6.0 5.5 5.0 4.5 4.0 3.5 3.0 2.5 2.0 1.5 1.0 0.5 0.0 -0.5 -1.0
f1 (ppm)

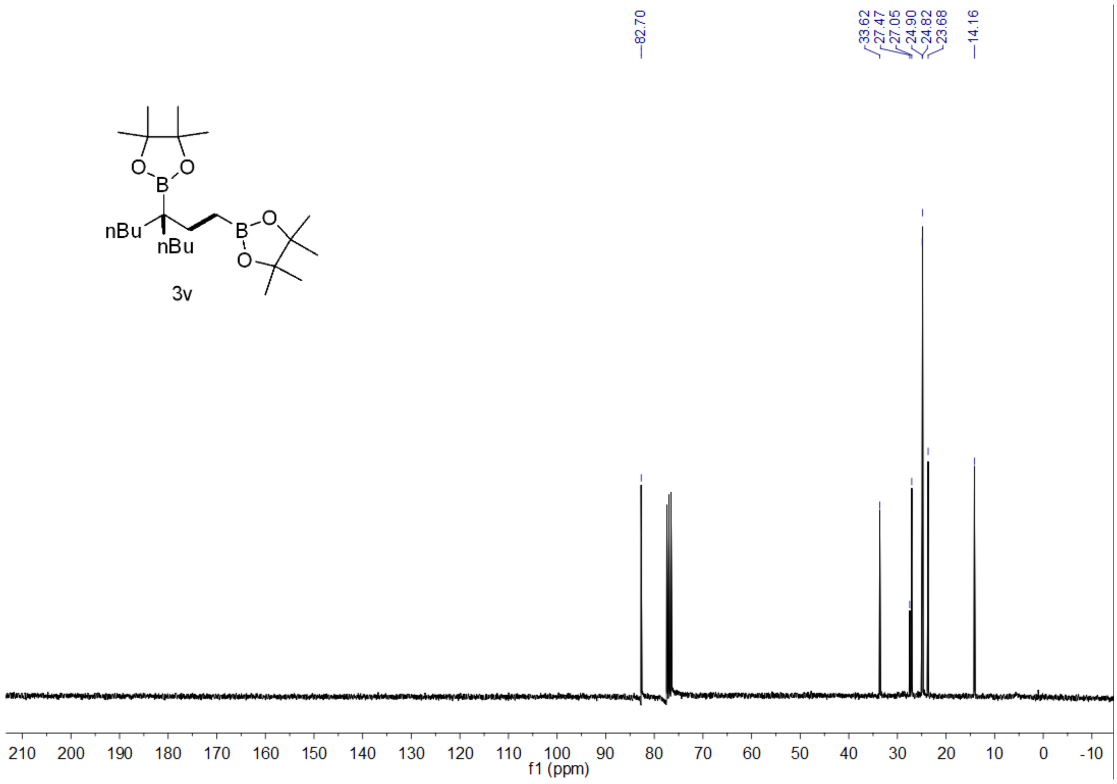
82.70
33.62
27.47
27.05
24.90
24.82
23.68
14.16
nBu
nBu
3v
210 200 190 180 170 160 150 140 130 120 110 100 90 80 70 60 50 40 30 20 10 0 -10
f1 (ppm)

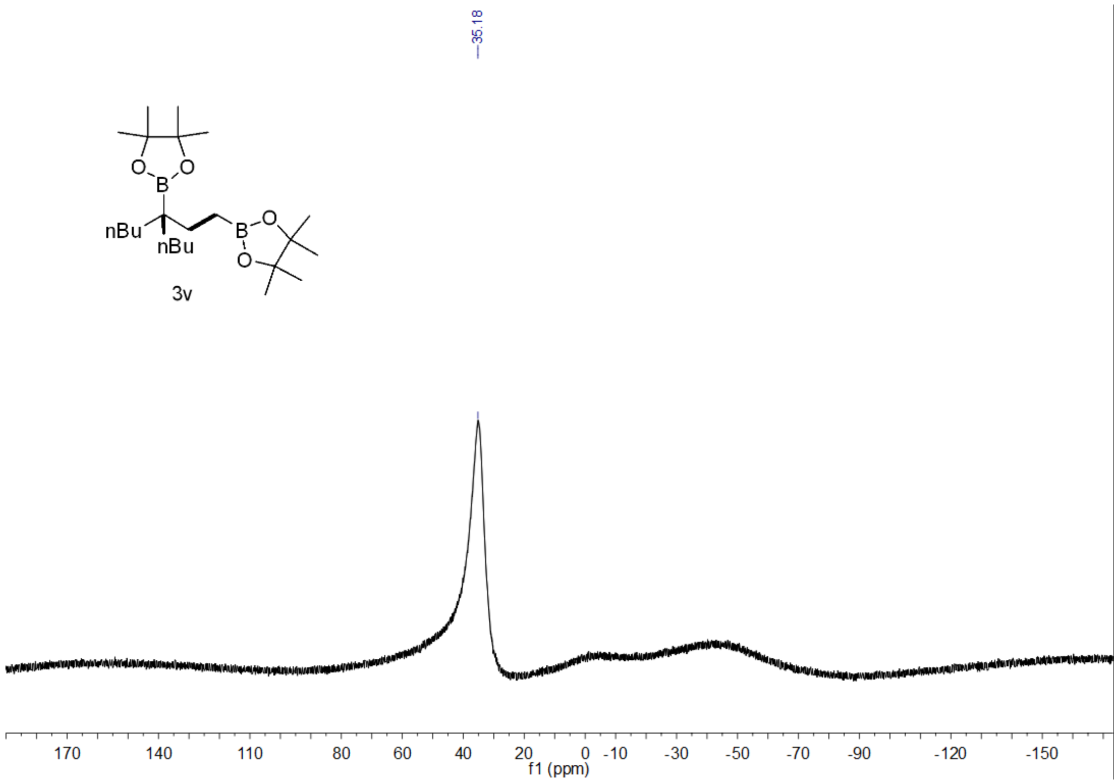
35.18
nBu
nBu
3v
170 140 110 80 60 40 20 0 -10 -30 -50 -70 -90 -120 -150
f1 (ppm)

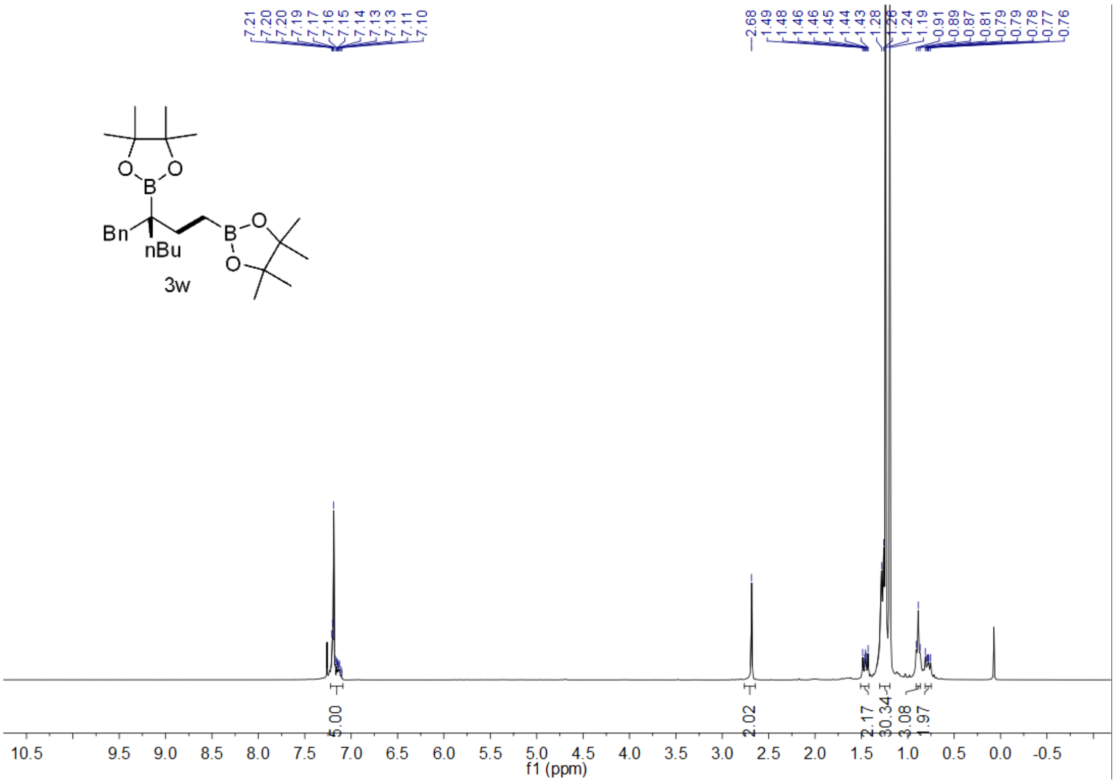
7.21
7.20
7.19
7.17
7.16
7.15
7.14
7.13
7.13
7.11
7.10
2.68
1.49
1.48
1.46
1.46
1.45
1.44
1.43
1.28
1.26
1.24
1.19
0.91
0.89
0.87
0.81
0.79
0.79
0.78
0.77
0.76
O
B
O
Bn
nBu
B
O
O
3w
5.00
2.02
2.17
30.34
3.08
1.97
f1 (ppm)

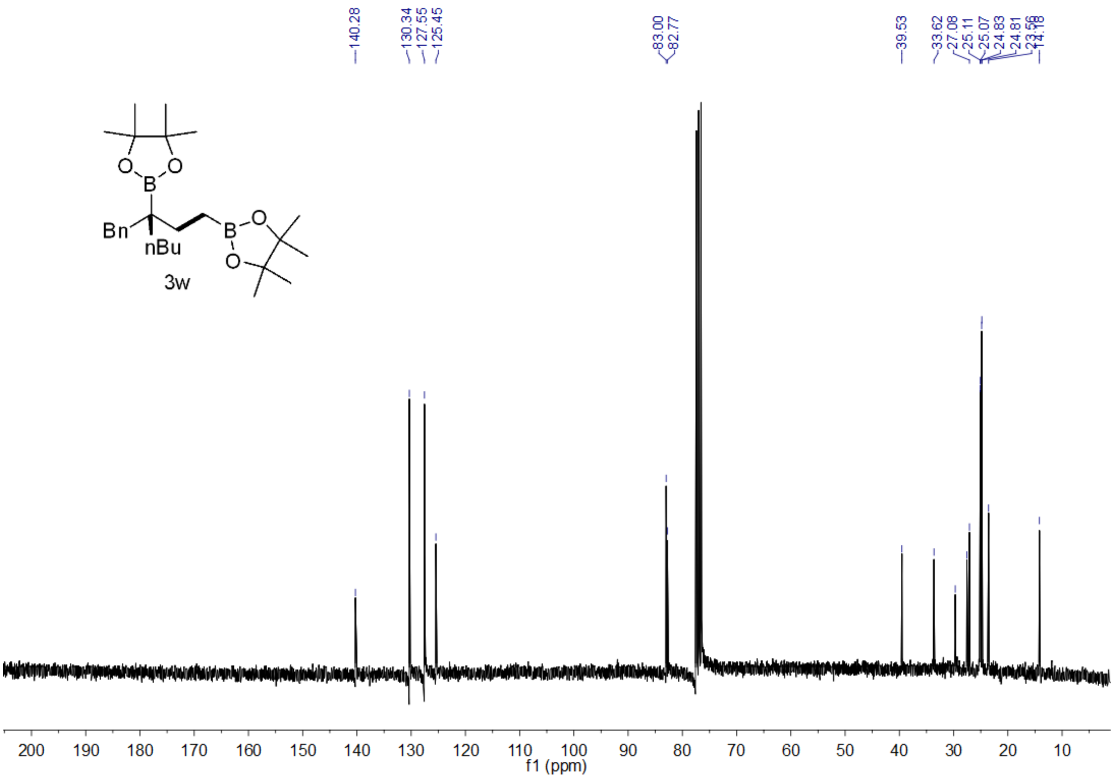
140.28
130.34
127.55
125.45
83.00
82.77
39.53
33.62
27.08
25.11
25.07
24.83
24.81
23.56
14.18
O
B
O
Bn
nBu
B
O
O
3w
f1 (ppm)

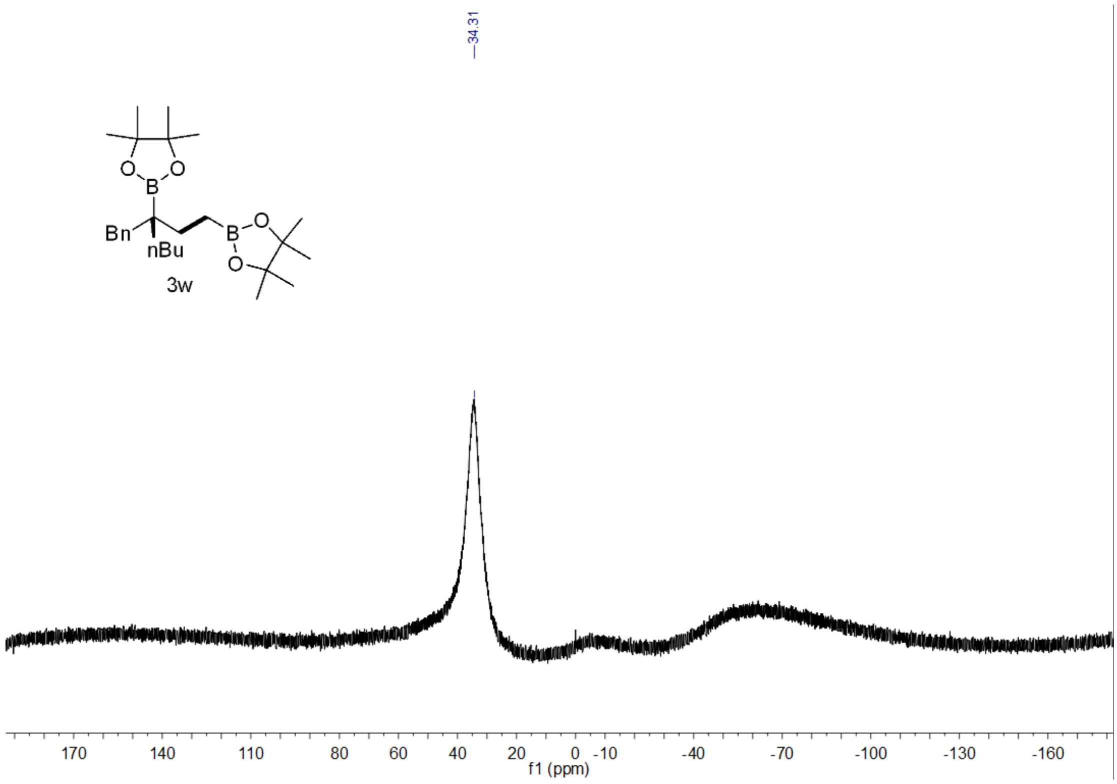
34.31
O
B
O
Bn
nBu
B
O
O
3w
170 140 110 80 60 40 20 0 -10 -40 -70 -100 -130 -160
f1 (ppm)

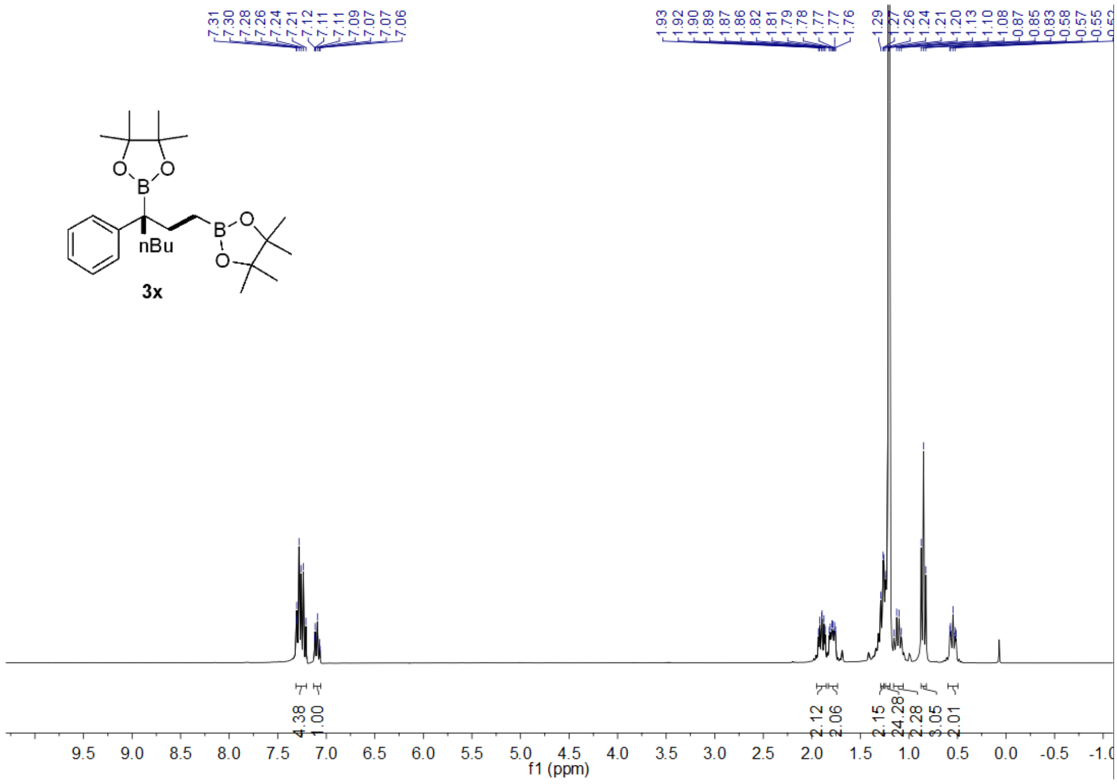
7.31 7.30 7.28 7.26 7.24 7.21 7.12 7.11 7.11 7.09 7.07 7.07 7.06
1.93 1.92 1.90 1.89 1.87 1.86 1.82 1.81 1.79 1.78 1.77 1.77 1.76
1.29 1.27 1.26 1.24 1.21 1.20 1.13 1.10 1.08 0.87 0.85 0.83 0.58 0.57 0.55
O
B
O
nBu
B
O
O
3x
4.38 1.00
2.12 2.06
2.15 24.28 2.28 3.05 2.01
9.5 9.0 8.5 8.0 7.5 7.0 6.5 6.0 5.5 5.0 4.5 4.0 3.5 3.0 2.5 2.0 1.5 1.0 0.5 0.0 -0.5 -1.0
f1 (ppm)

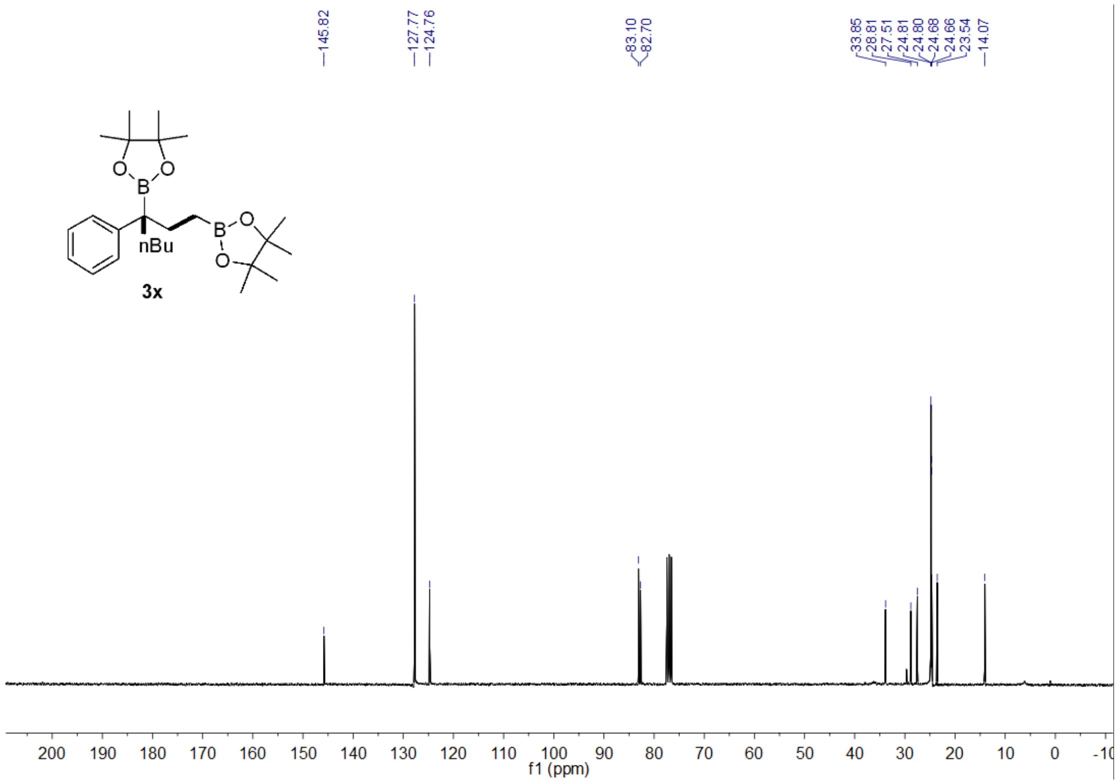
145.82
127.77
124.76
83.10
82.70
33.85
28.81
27.51
24.81
24.80
24.68
24.66
23.54
14.07
nBu
3x
f1 (ppm)

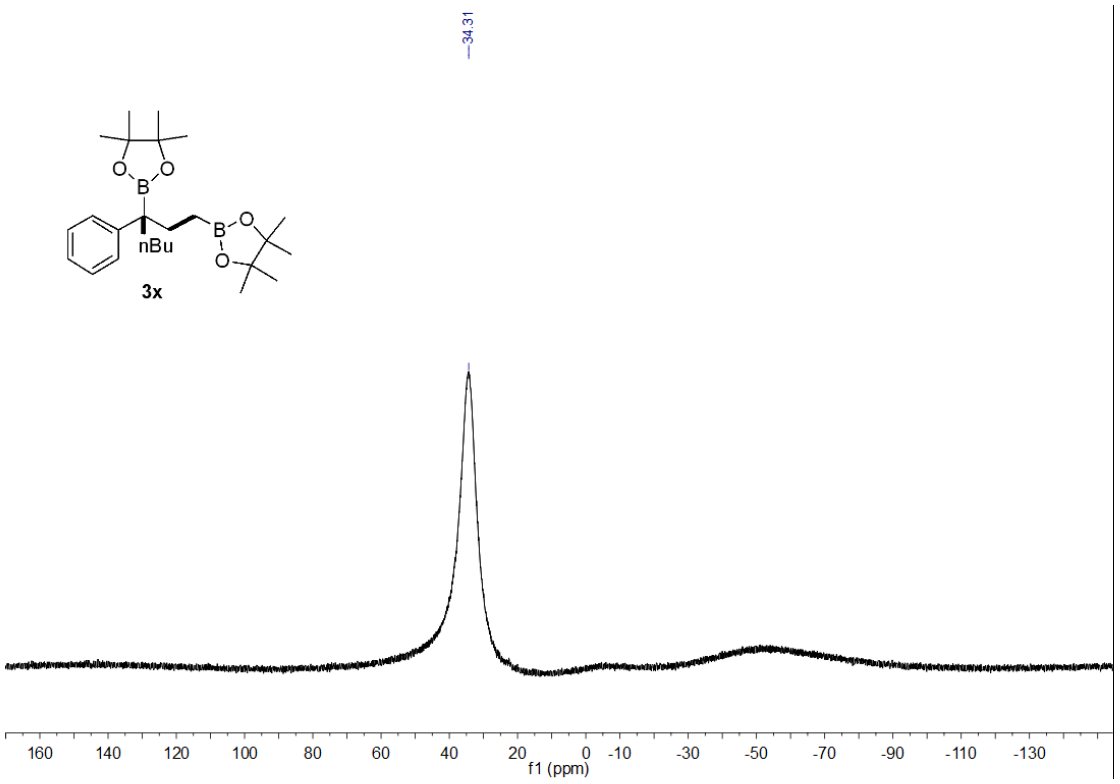
34.31
nBu
3x
f1 (ppm)

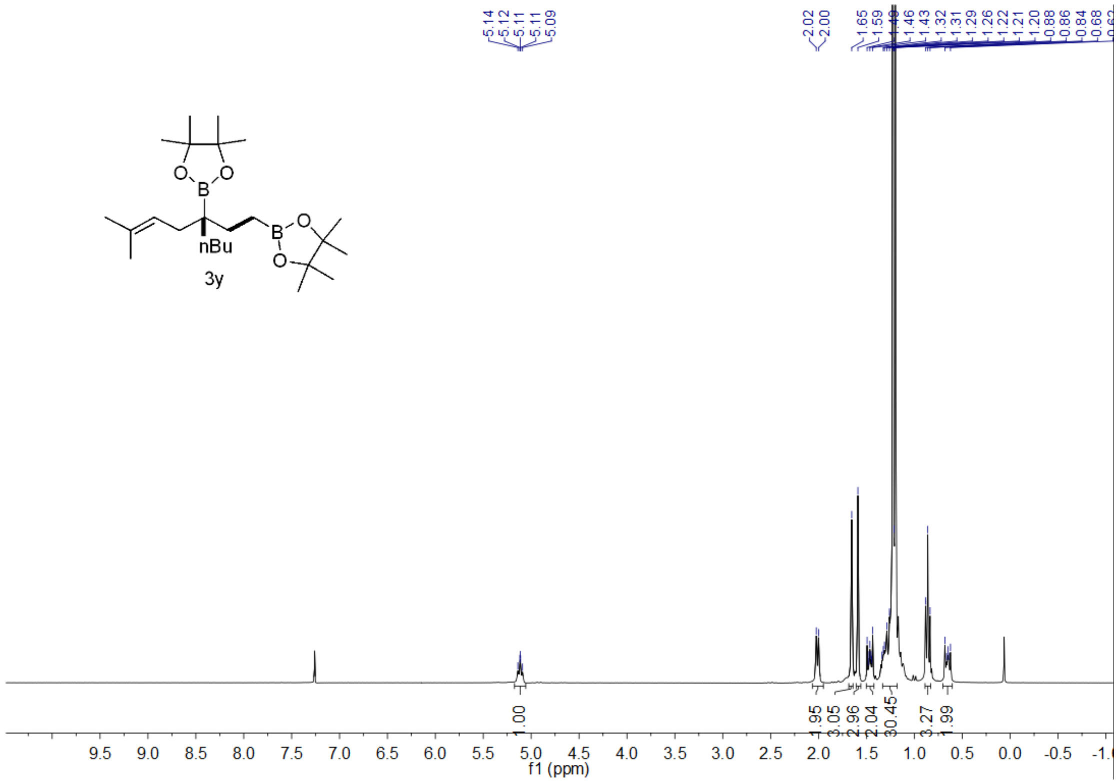
5.14
5.12
5.11
5.11
5.09
2.02
2.00
1.65
1.59
1.46
1.43
1.32
1.31
1.29
1.26
1.22
1.21
1.20
0.88
0.86
0.84
0.68
1.00
1.95
3.05
2.96
2.04
30.45
3.27
1.99
O
B
O
B
O
O
nBu
3y
9.5 9.0 8.5 8.0 7.5 7.0 6.5 6.0 5.5 5.0 4.5 4.0 3.5 3.0 2.5 2.0 1.5 1.0 0.5 0.0 -0.5
f1 (ppm)

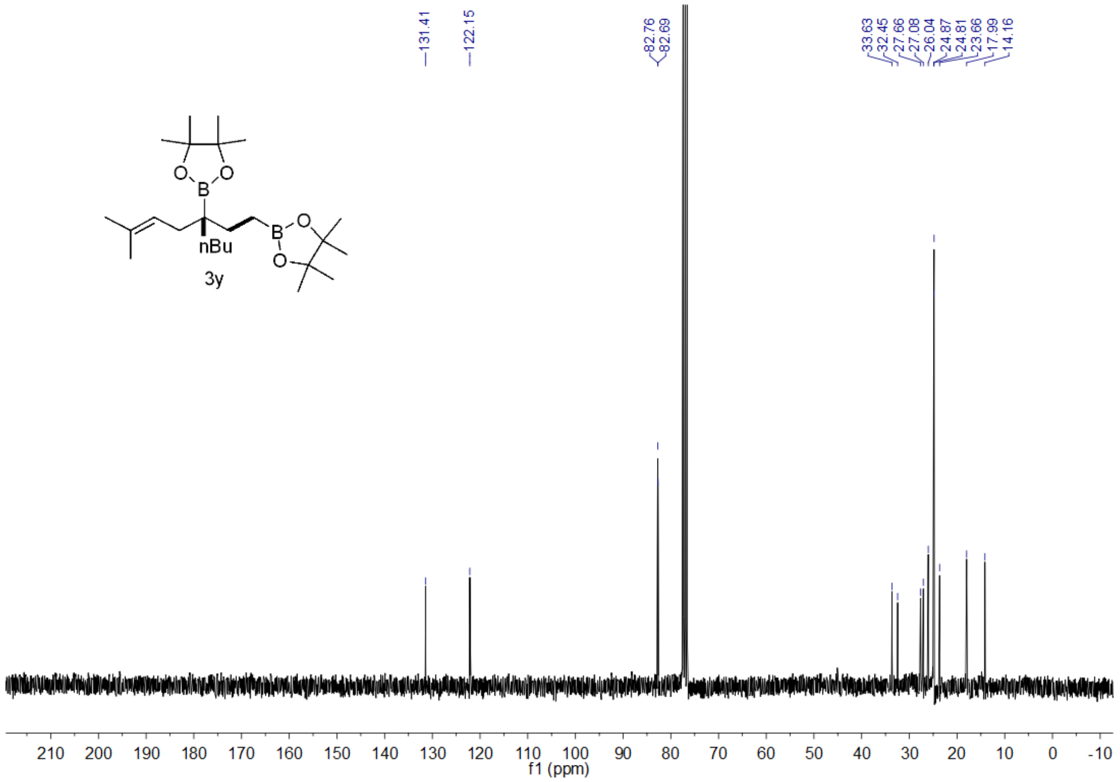
131.41
122.15
82.76
82.69
33.63
32.45
27.66
27.08
26.04
24.87
24.81
23.66
17.99
14.16
O
B
O
B
O
O
nBu
3y
210 200 190 180 170 160 150 140 130 120 110 100 90 80 70 60 50 40 30 20 10 0 -10
f1 (ppm)

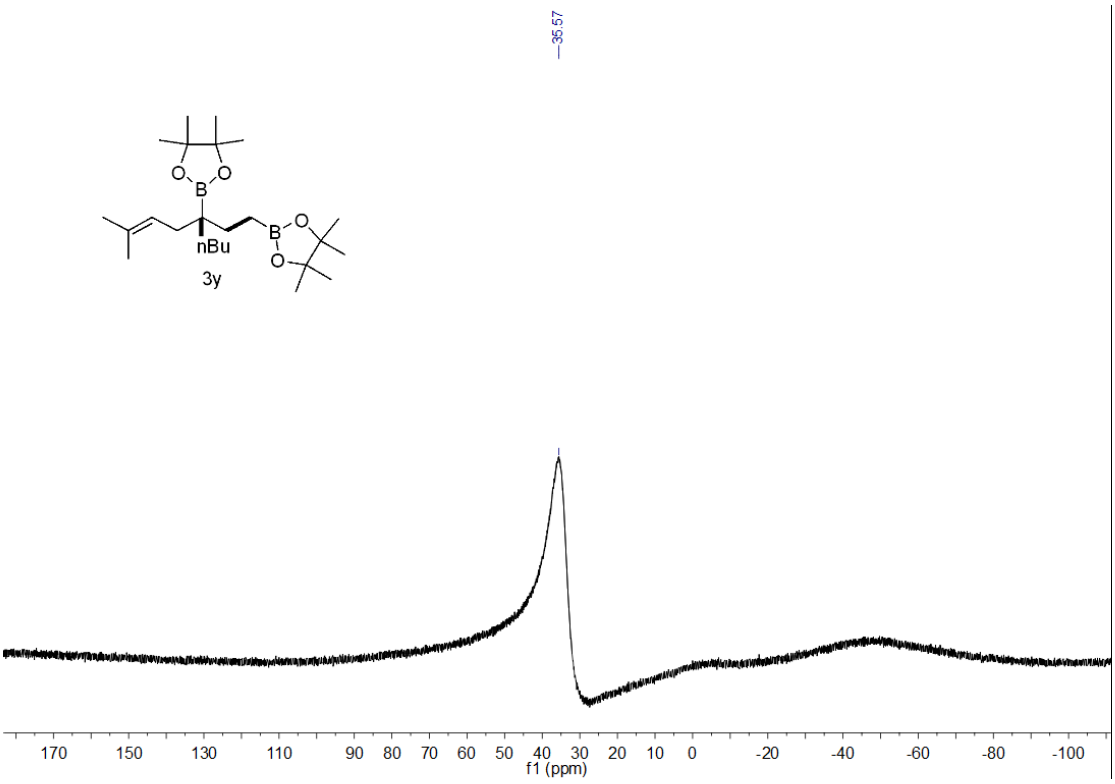

3y

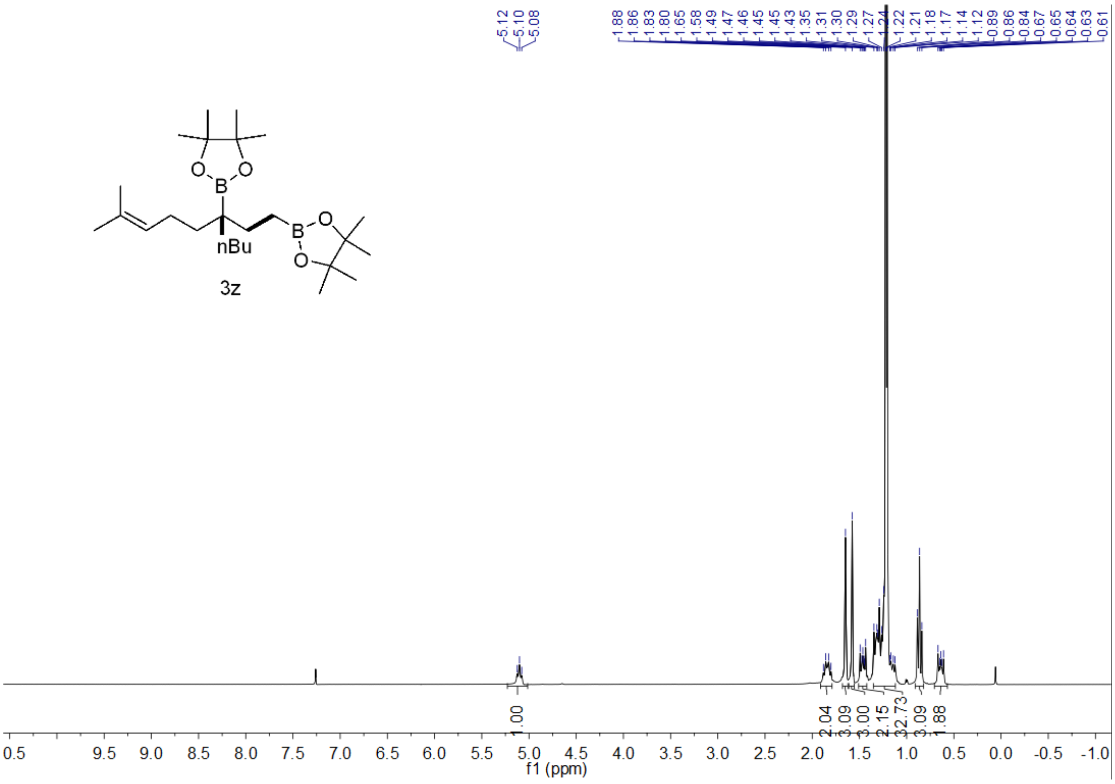

3z

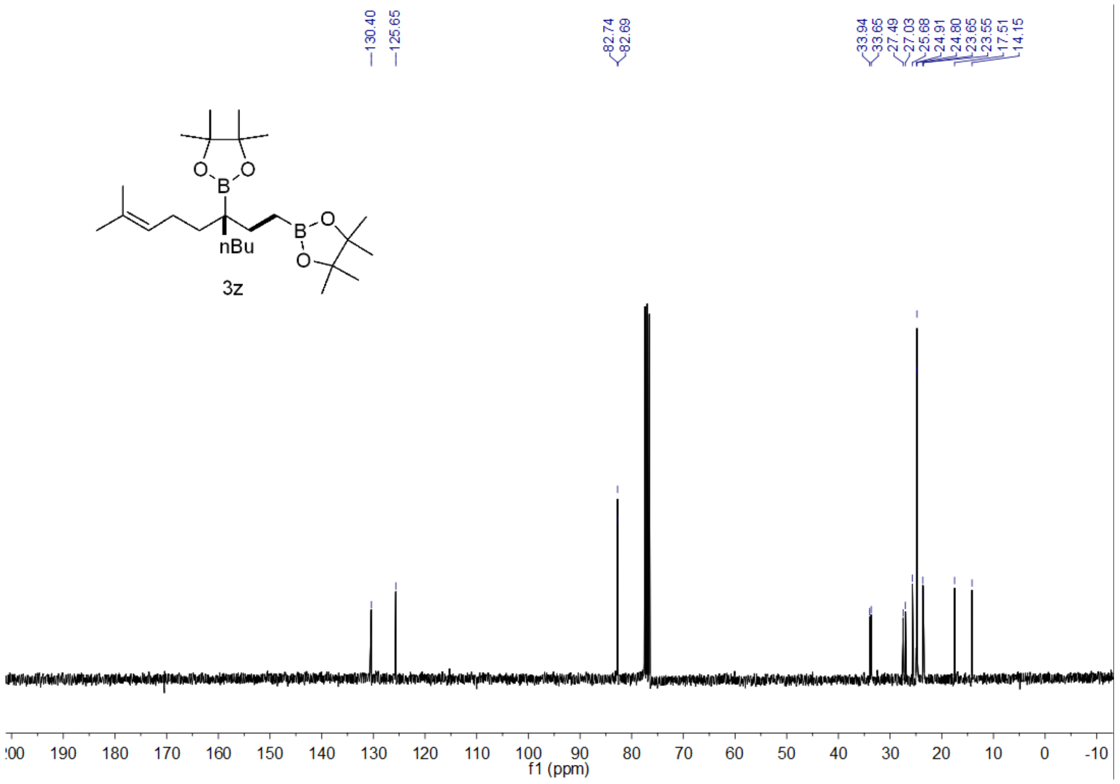
130.40
125.65
82.74
82.69
33.94
33.65
27.49
27.03
25.68
24.91
24.80
23.65
23.55
17.51
14.15
nBu
3z
f1 (ppm)

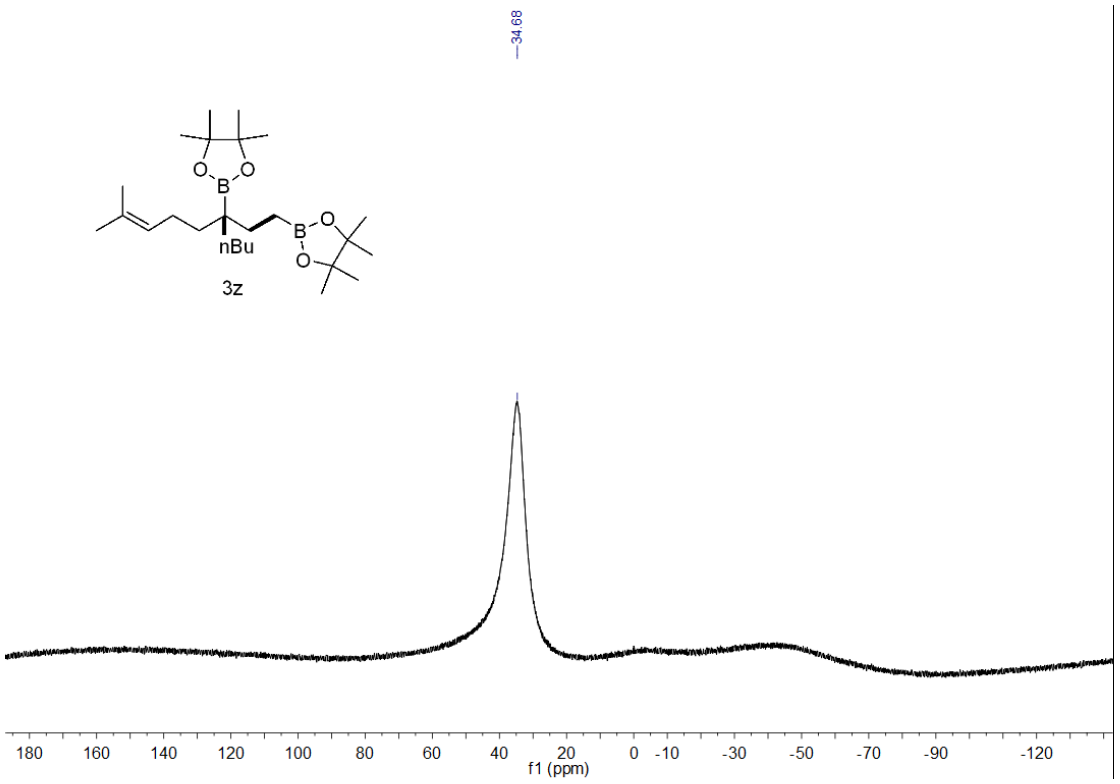
34.68
nBu
3z
f1 (ppm)

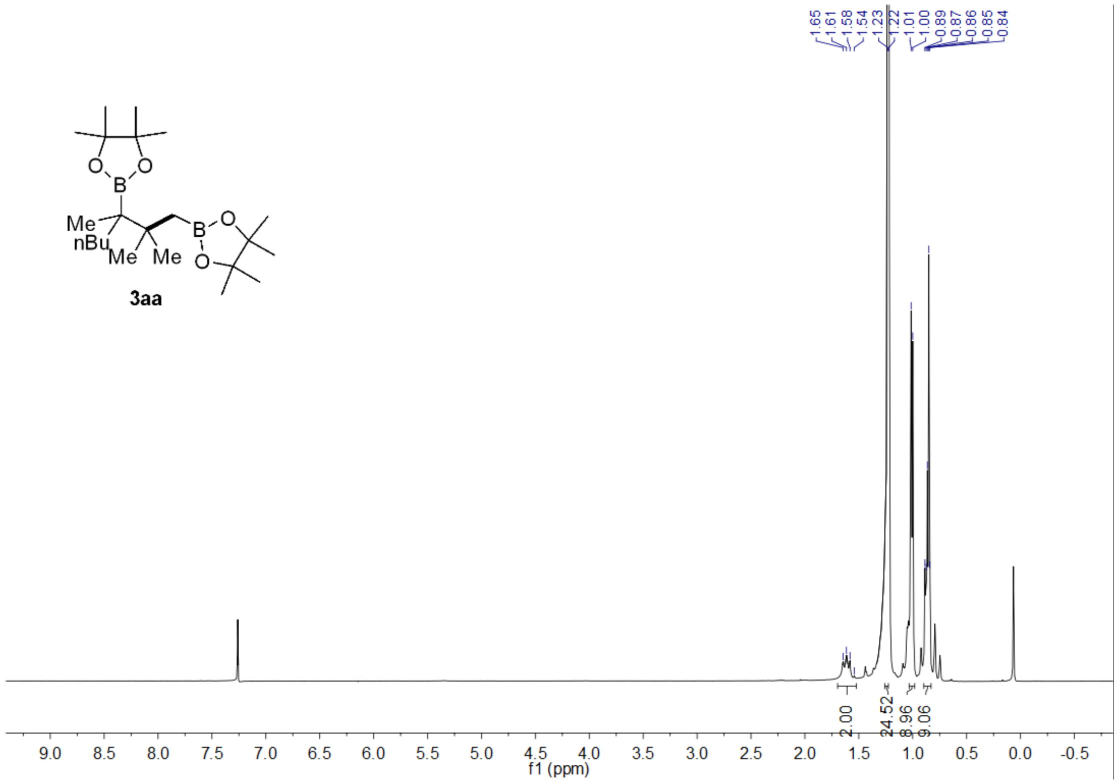
1.65
1.61
1.58
1.54
1.23
1.22
1.01
1.00
0.89
0.87
0.86
0.85
0.84
3aa
2.00
24.52
8.96
9.06
f1 (ppm)

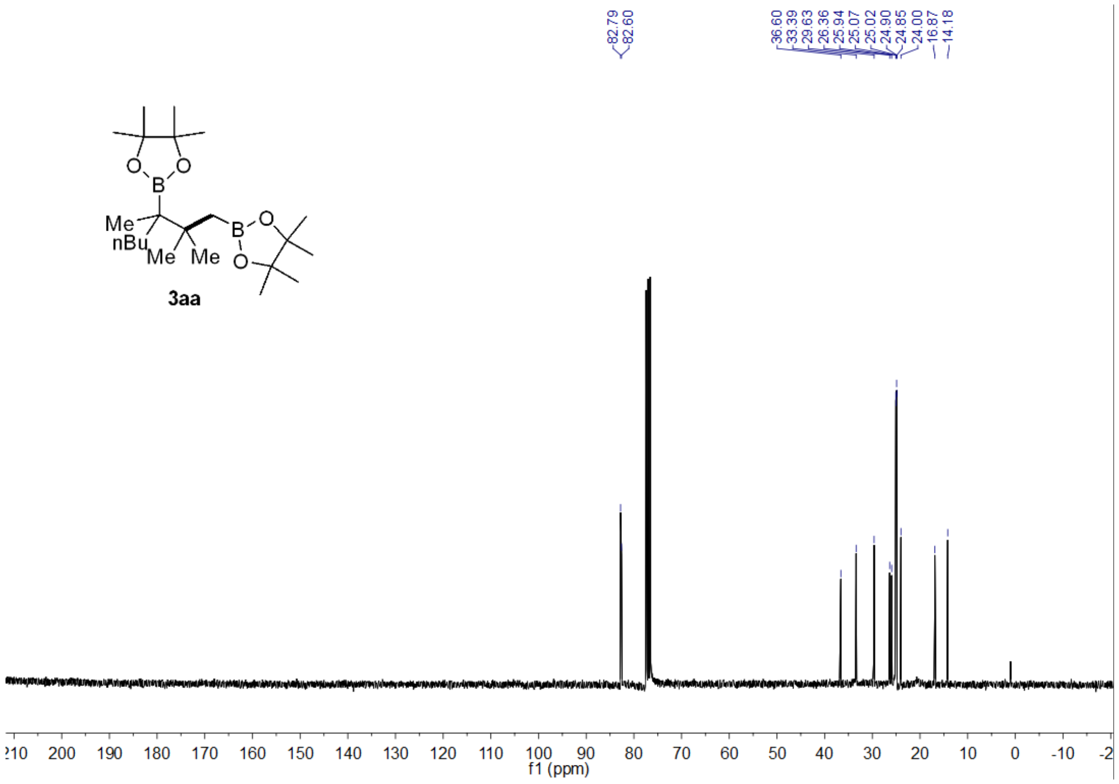
82.79
82.60
36.60
33.39
29.63
26.36
25.94
25.07
25.02
24.90
24.85
24.00
16.87
14.18
3aa
f1 (ppm)

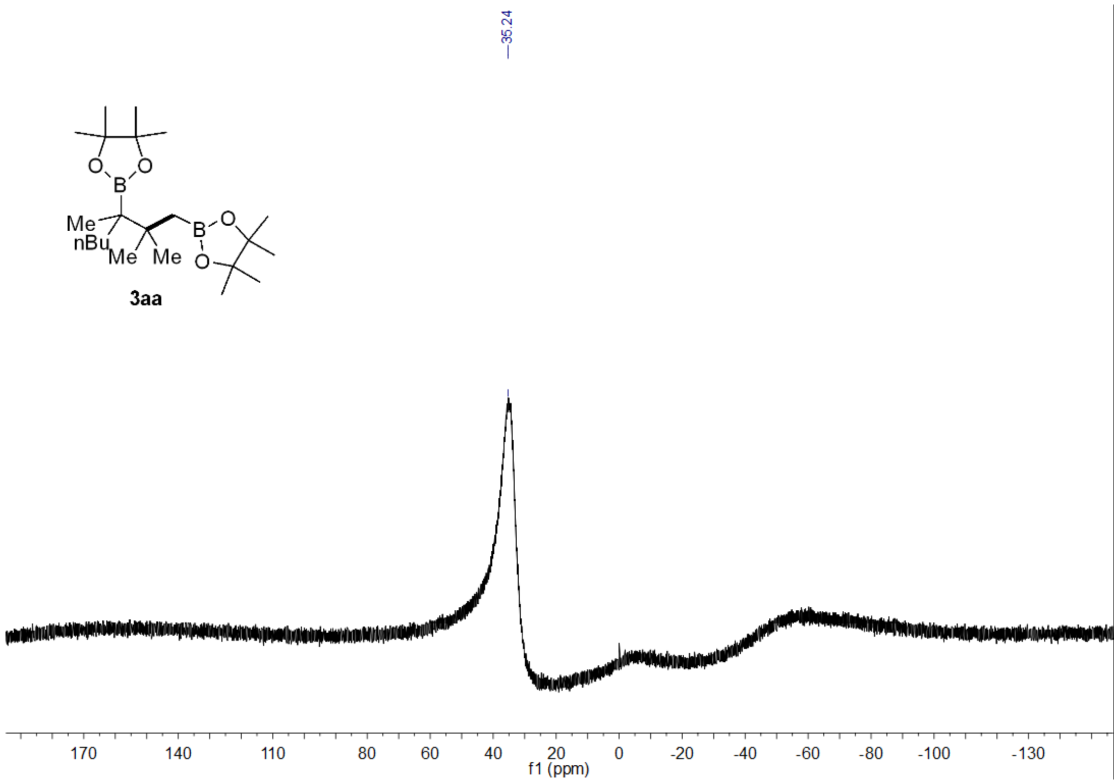
35.24
Me
nBu
Me
Me
B
O
O
3aa
f1 (ppm)

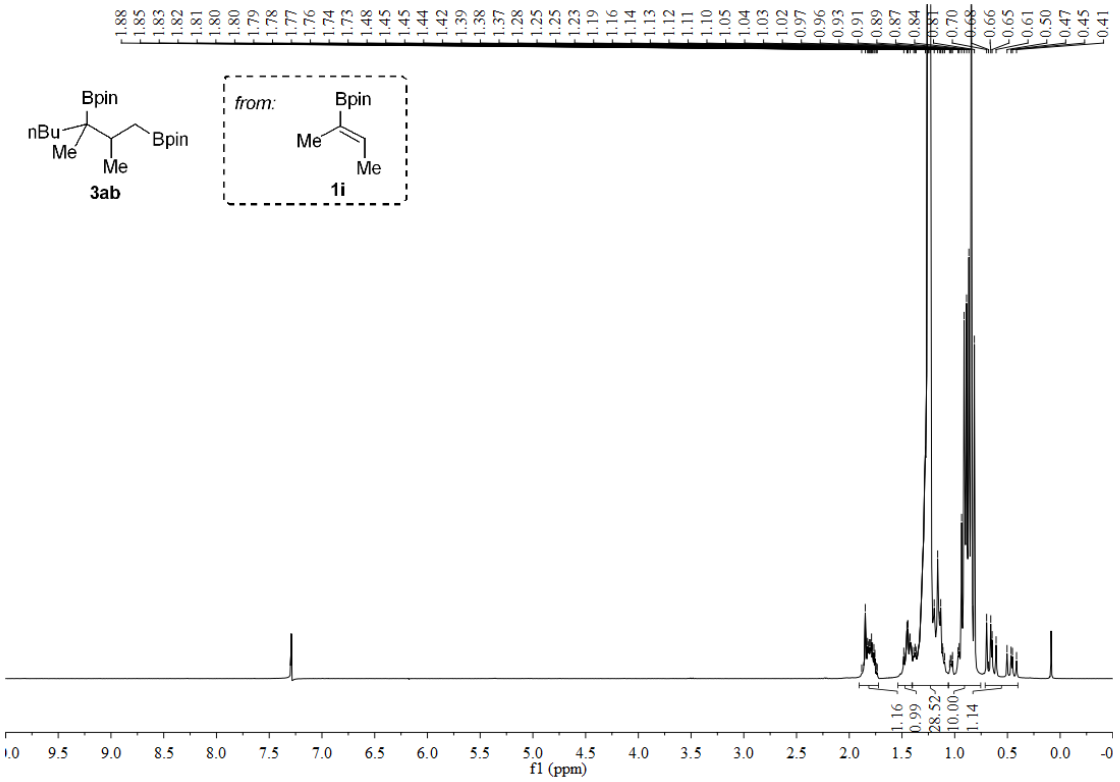
Bpin
nBu
Me
Me
Bpin
3ab
from:
Bpin
Me
Me
1i
f1 (ppm)

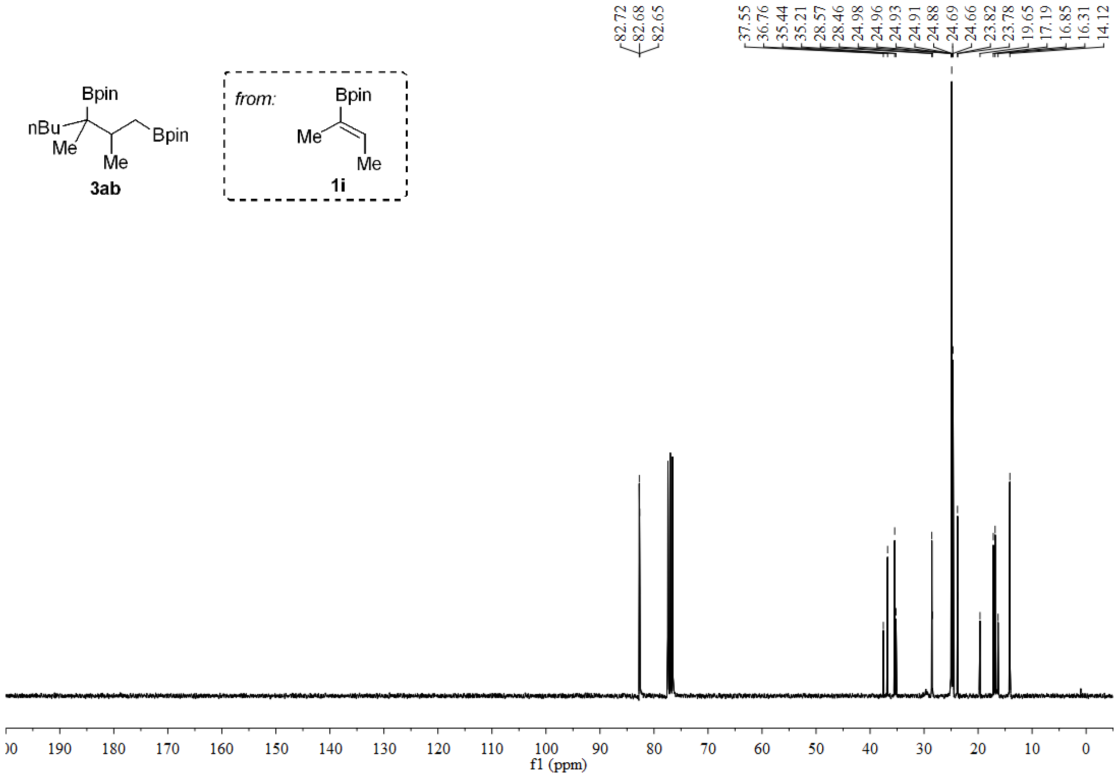

Bpin
nBu
Me
Bpin
Me
3ab
from:
Bpin
Me
Me
1i
82.72
82.68
82.65
37.55
36.76
35.44
35.21
28.57
28.46
24.98
24.96
24.93
24.91
24.88
24.69
24.66
23.82
23.78
19.65
17.19
16.85
16.31
14.12
f1 (ppm)

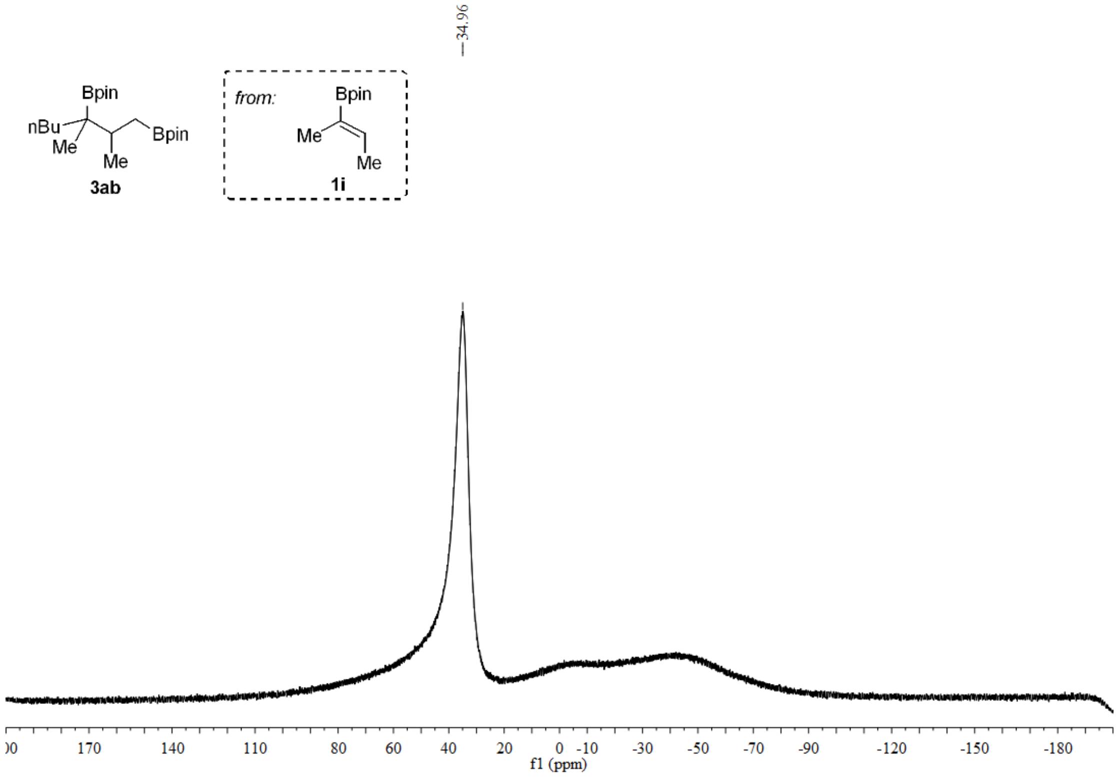

Bpin
nBu
Me
Bpin
Me
3ab
from:
Bpin
Me
Me
1i
34.96
f1 (ppm)

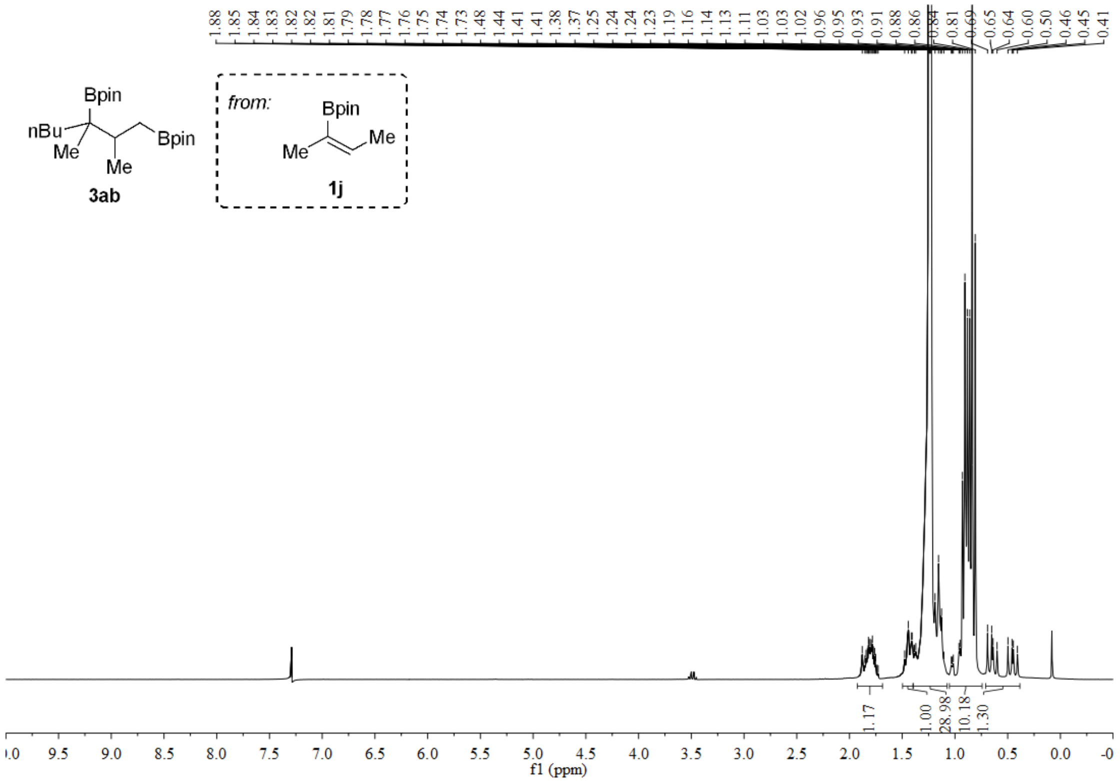
1.88 1.85 1.84 1.83 1.82 1.82 1.81 1.79 1.78 1.77 1.76 1.75 1.74 1.73 1.48 1.44 1.41 1.41 1.38 1.37 1.25 1.24 1.24 1.23 1.19 1.16 1.14 1.13 1.11 1.03 1.03 1.02 0.96 0.95 0.93 0.91 0.88 0.86 0.84 0.81 0.69 0.65 0.64 0.60 0.50 0.46 0.45 0.41
Bpin
nBu
Me
Bpin
Me
3ab
from:
Bpin
Me
Me
1j
1.17
1.00
28.98
10.18
1.30
f1 (ppm)

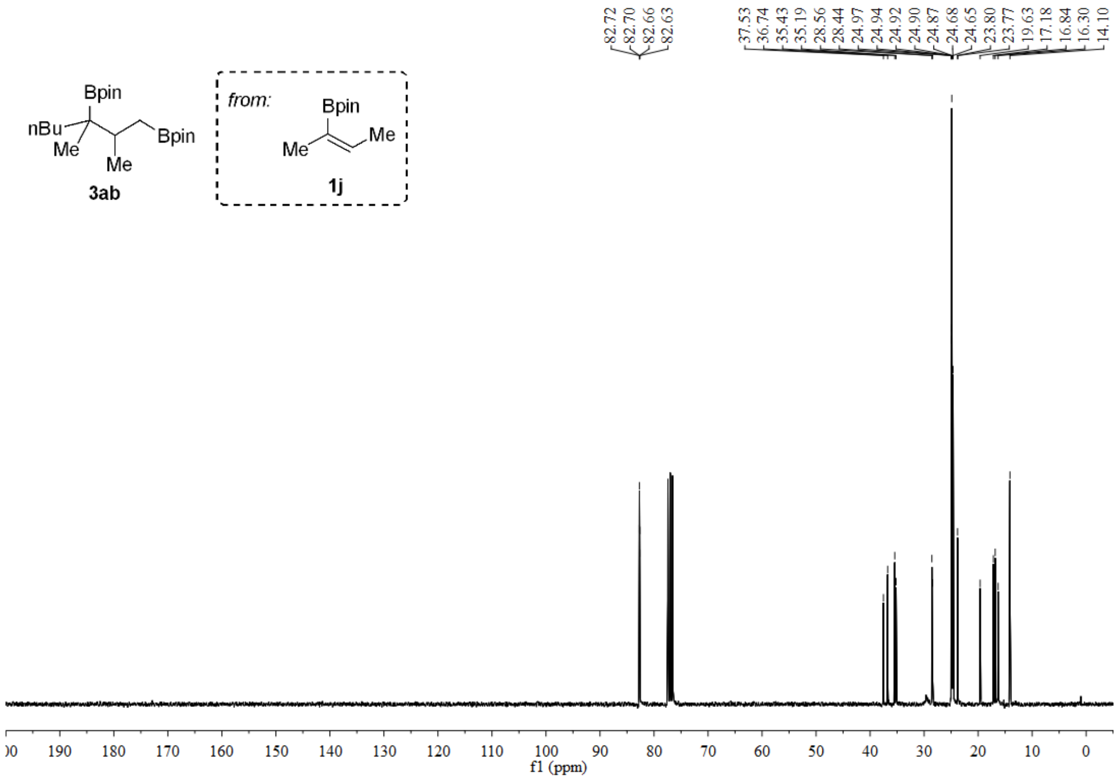
82.72 82.70 82.66 82.63
37.53 36.74 35.43 35.19 28.56 28.44 24.97 24.94 24.92 24.90 24.87 24.68 24.65 23.80 23.77 19.63 17.18 16.84 16.30 14.10
Bpin
nBu
Me
Bpin
Me
3ab
from:
Bpin
Me
Me
1j
f1 (ppm)

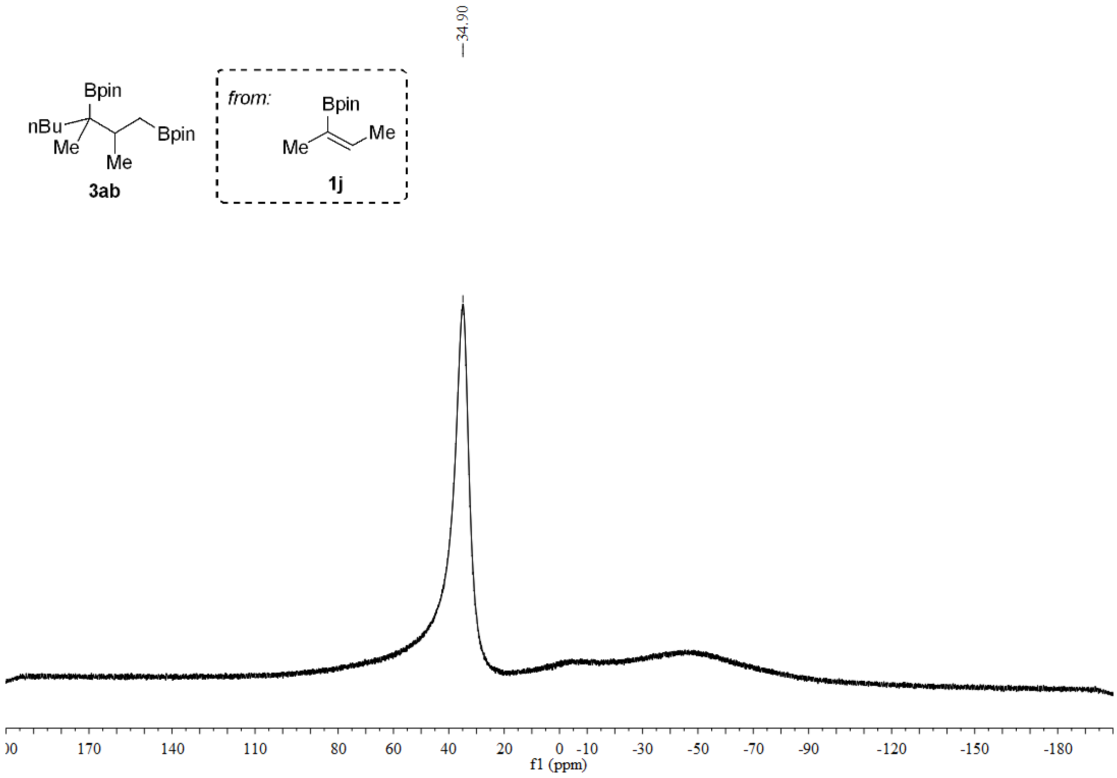
34.90
Bpin
nBu
Me
Bpin
Me
3ab
from:
Bpin
Me
Me
1j
f1 (ppm)

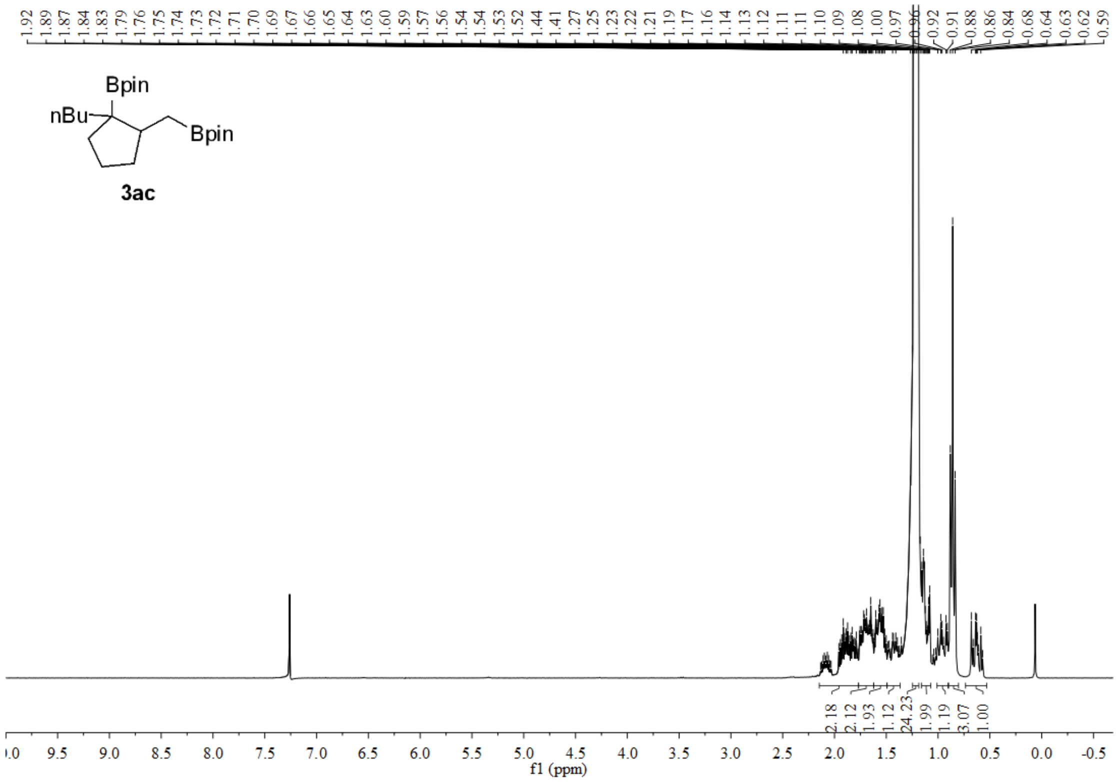
1.92 1.89 1.87 1.84 1.83 1.79 1.76 1.75 1.74 1.73 1.72 1.71 1.70 1.69 1.67 1.66 1.65 1.64 1.63 1.60 1.59 1.57 1.56 1.54 1.54 1.53 1.52 1.44 1.41 1.27 1.25 1.23 1.22 1.21 1.19 1.17 1.16 1.14 1.13 1.12 1.11 1.11 1.10 1.09 1.08 1.00 0.97 0.96 0.92 0.91 0.88 0.86 0.84 0.68 0.64 0.63 0.62 0.59
Bpin
nBu
Bpin
3ac
2.18 2.12 1.93 1.12 24.23 1.99 1.19 3.07 1.00
f1 (ppm)

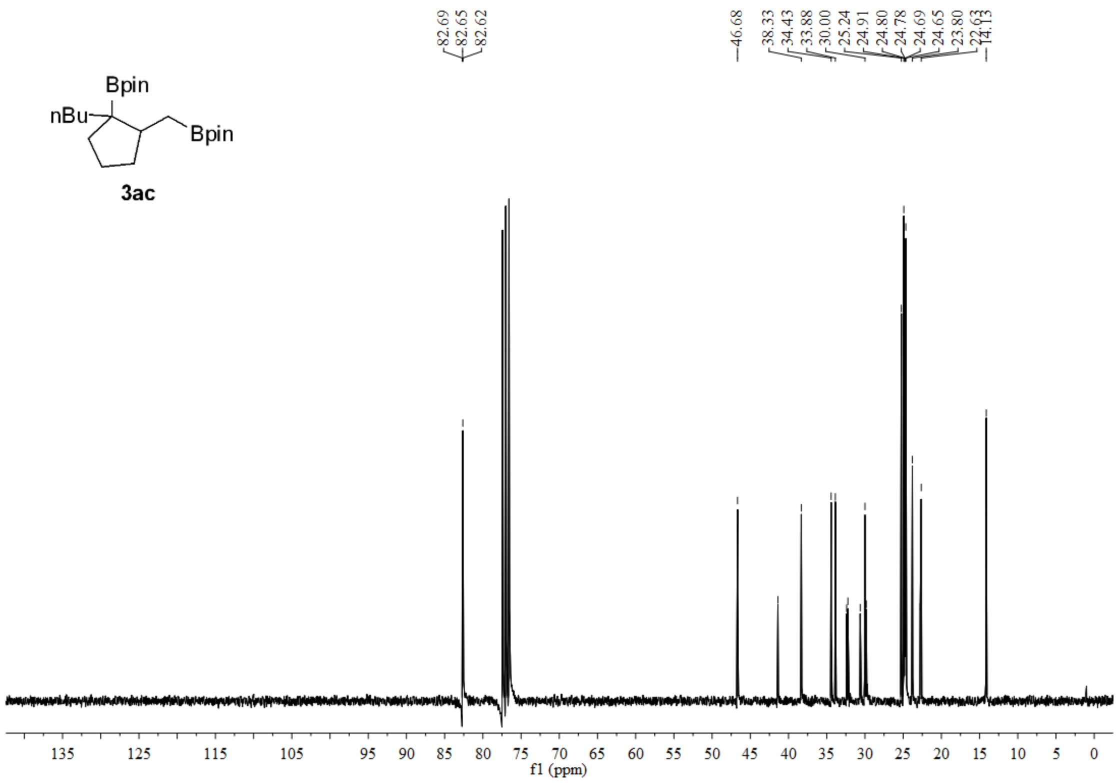
Bpin
nBu
Bpin
3ac
82.69
82.65
82.62
46.68
38.33
34.43
33.88
30.00
25.24
24.91
24.80
24.78
24.69
24.65
23.80
22.63
14.13
135
125
115
105
95
90
85
80
75
70
65
60
55
50
45
40
35
30
25
20
15
10
5
0
f1 (ppm)

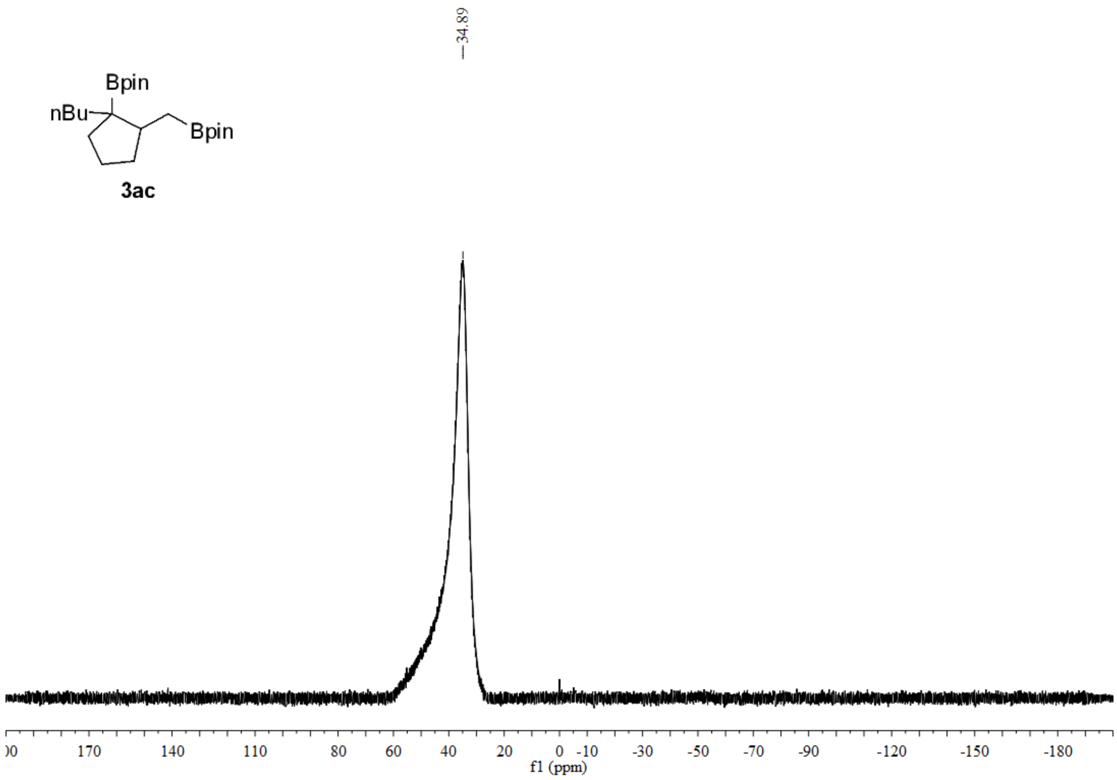
Bpin
nBu
Bpin
3ac
34.89
170
140
110
80
60
40
20
0
-10
-30
-50
-70
-90
-120
-150
-180
f1 (ppm)

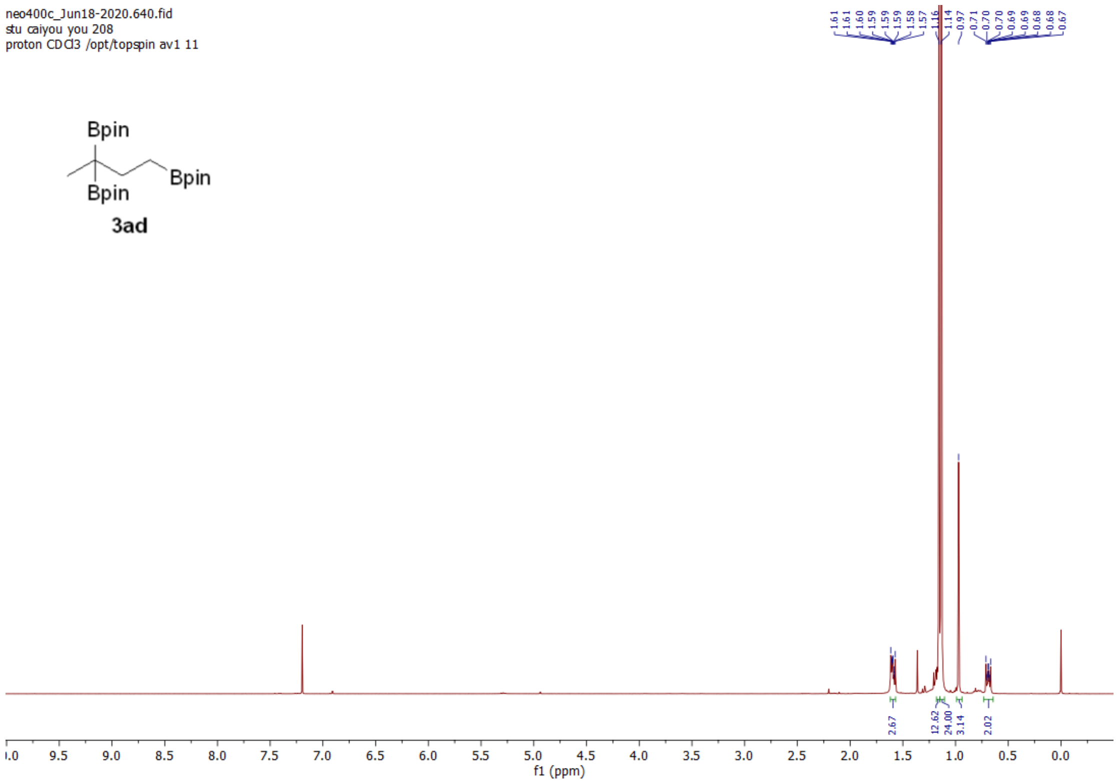
neo400c_Jun18-2020.640.fid
stu caiyou you 208
proton CDCl3 /opt/topspin av1 11
Bpin
Bpin
Bpin
3ad
1.61 1.61 1.60 1.59 1.59 1.59 1.58 1.57 1.16 1.14 0.97 0.71 0.70 0.70 0.69 0.69 0.68 0.68 0.67
2.67
12.62
24.00
3.14
2.02
f1 (ppm)

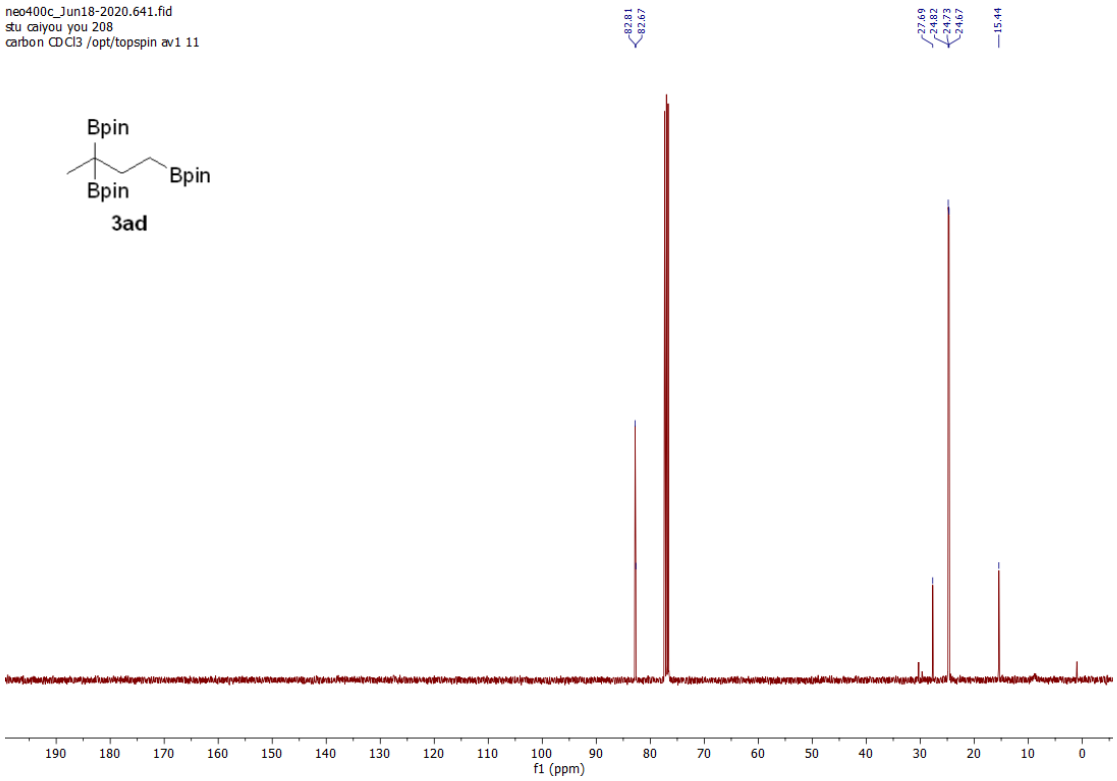
neo400c_Jun18-2020.641.fid
stu caiyou you 208
carbon CDCl3 /opt/topspin av1 11
82.81 82.67
27.69 24.82 24.73 24.67
15.44
Bpin
Bpin
Bpin
3ad
f1 (ppm)

neo400c_Jun22-2020.1111.fid
stu you 208
b11cpd CDCl3 /opt/topspin av1 29

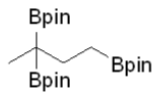

3ad

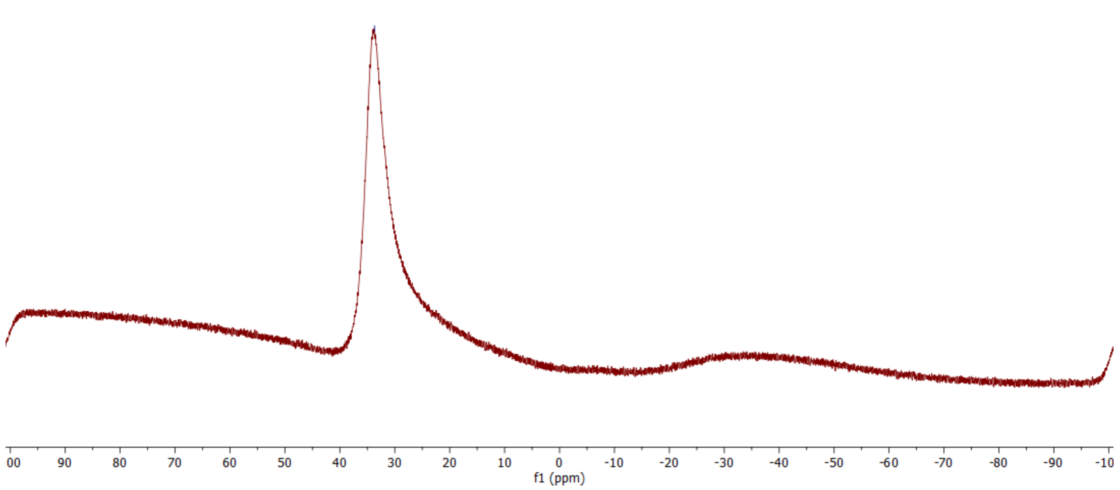

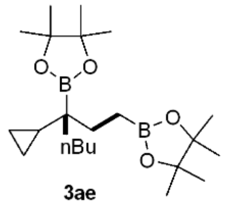

3ae

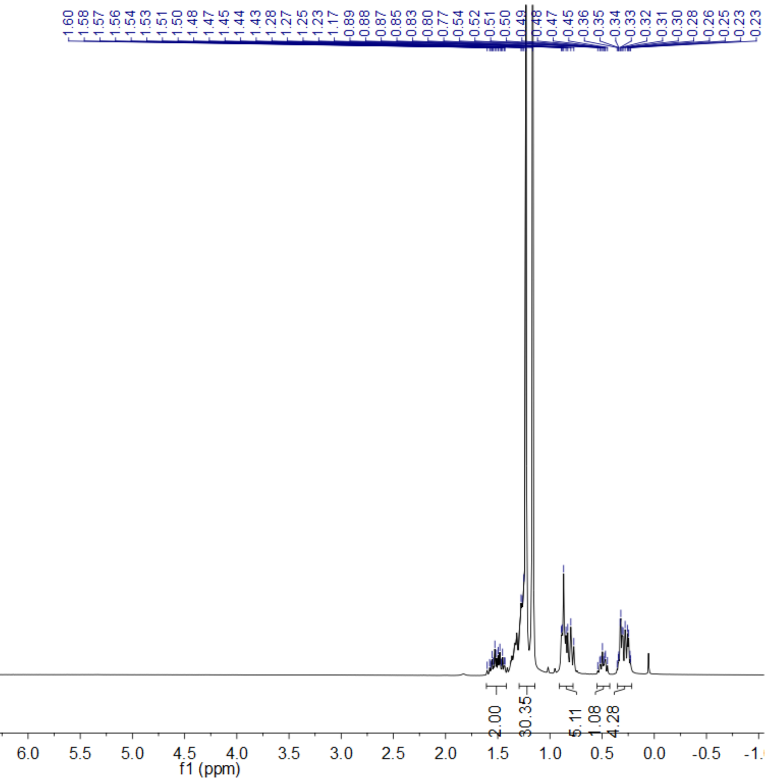

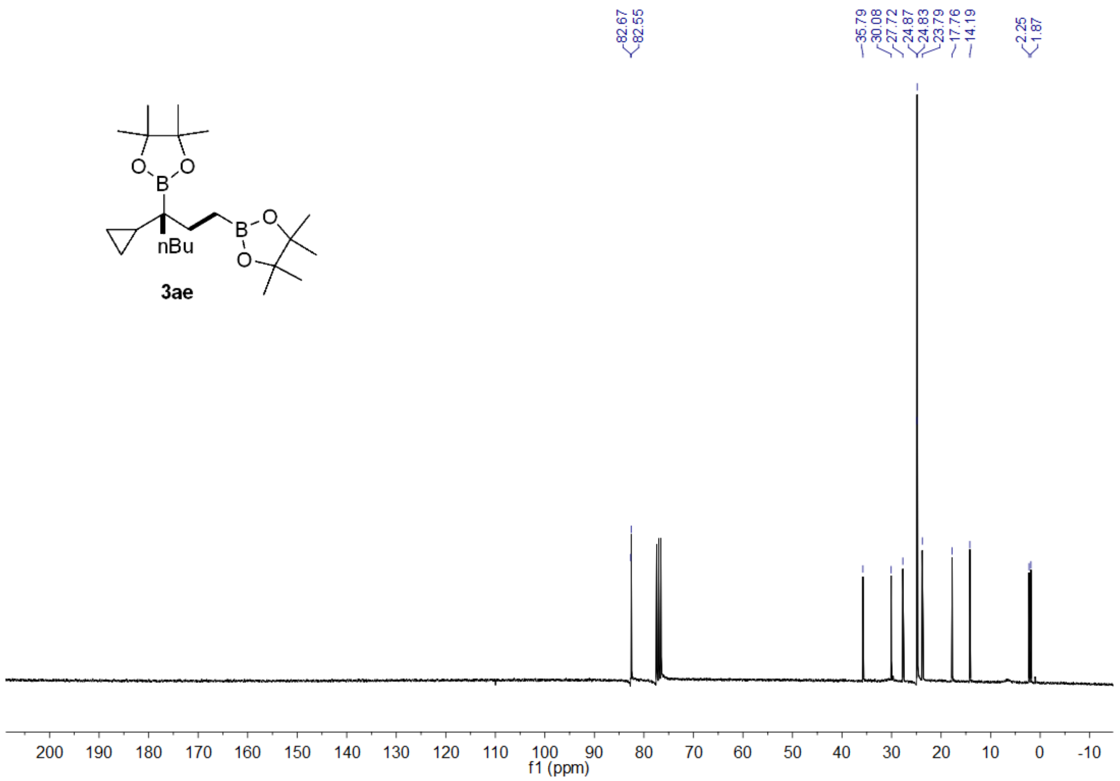
82.67
82.55
35.79
30.08
27.72
24.87
24.83
23.79
17.76
14.19
2.25
1.87
nBu
3ae
200 190 180 170 160 150 140 130 120 110 100 90 80 70 60 50 40 30 20 10 0 -10
f1 (ppm)

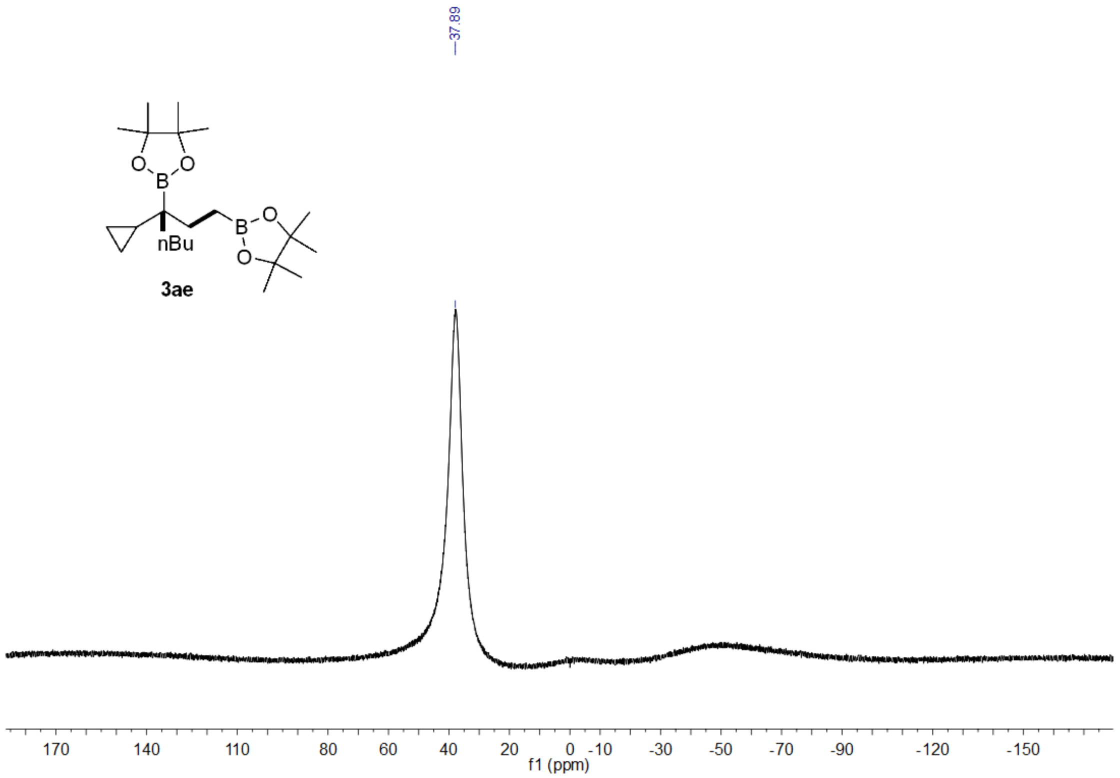
37.89
nBu
3ae
170 140 110 80 60 40 20 0 -10 -30 -50 -70 -90 -120 -150
f1 (ppm)

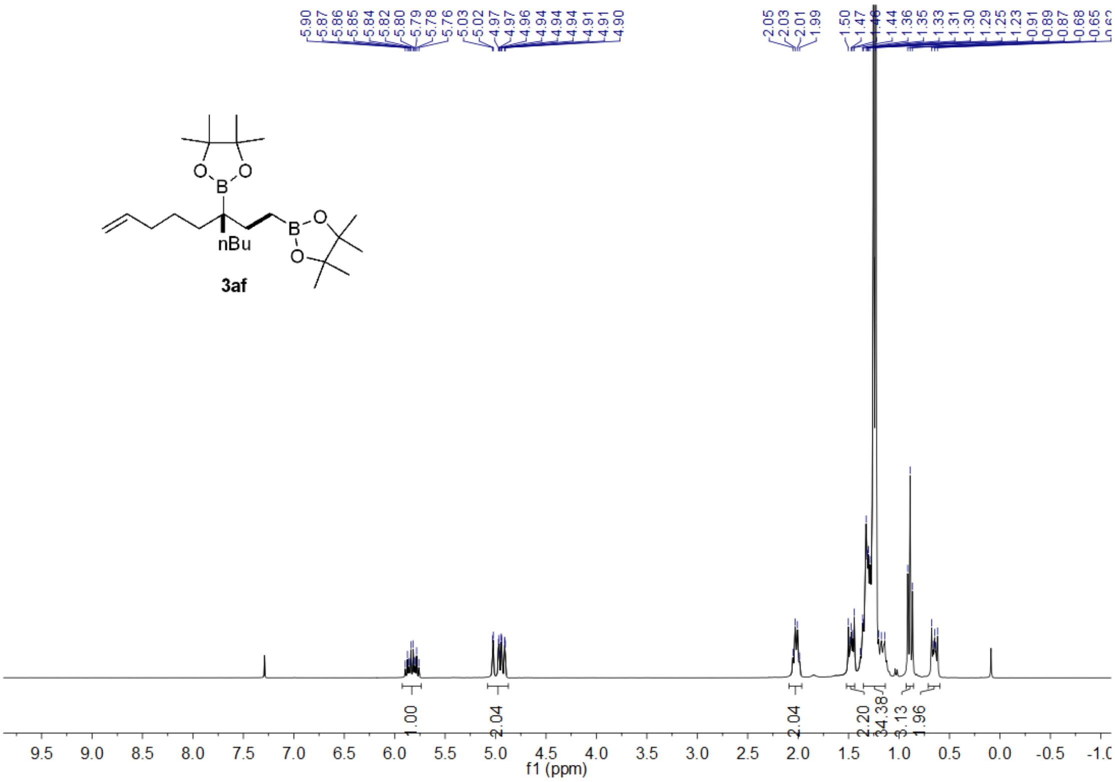

5.90
5.87
5.86
5.85
5.84
5.82
5.80
5.79
5.78
5.76
5.03
5.02
4.97
4.97
4.96
4.94
4.94
4.94
4.91
4.91
4.90
2.05
2.03
2.01
1.99
1.50
1.47
1.44
1.36
1.35
1.33
1.31
1.30
1.29
1.25
1.23
0.91
0.89
0.87
0.68
0.65
nBu
3af
1.00
2.04
2.04
2.20
34.38
3.13
1.96
f1 (ppm)

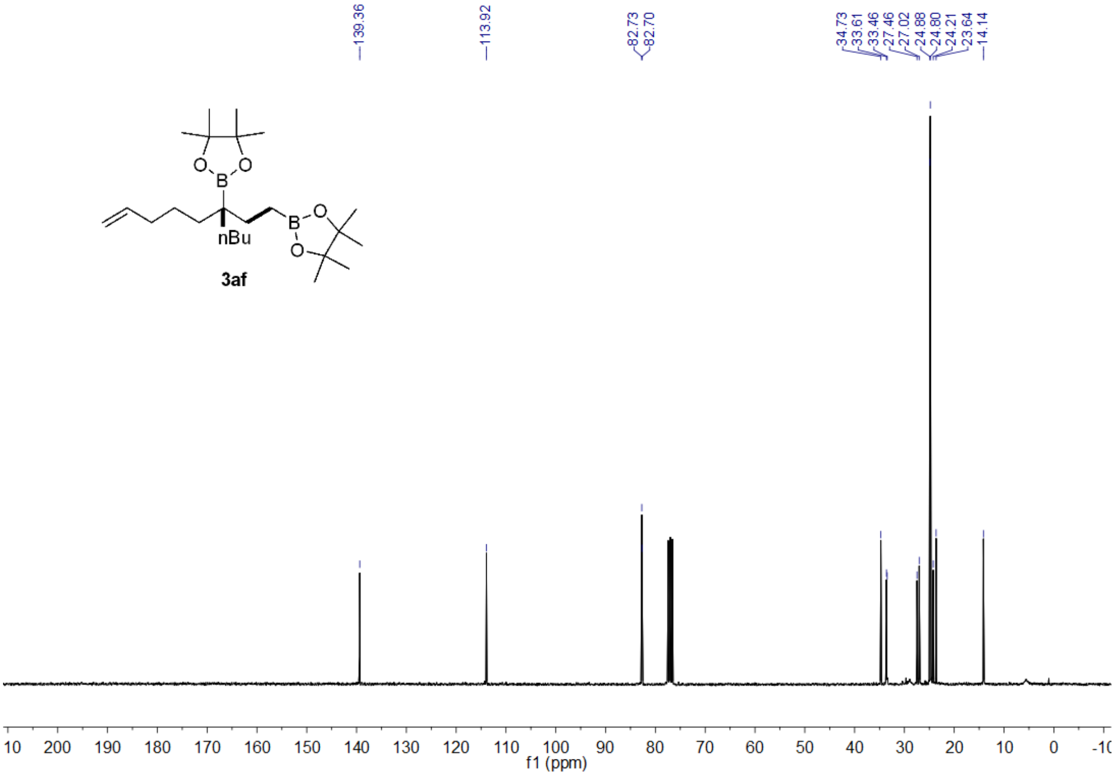

139.36
113.92
82.73
82.70
34.73
33.61
33.46
27.46
27.02
24.88
24.80
24.21
23.64
14.14
nBu
3af
f1 (ppm)

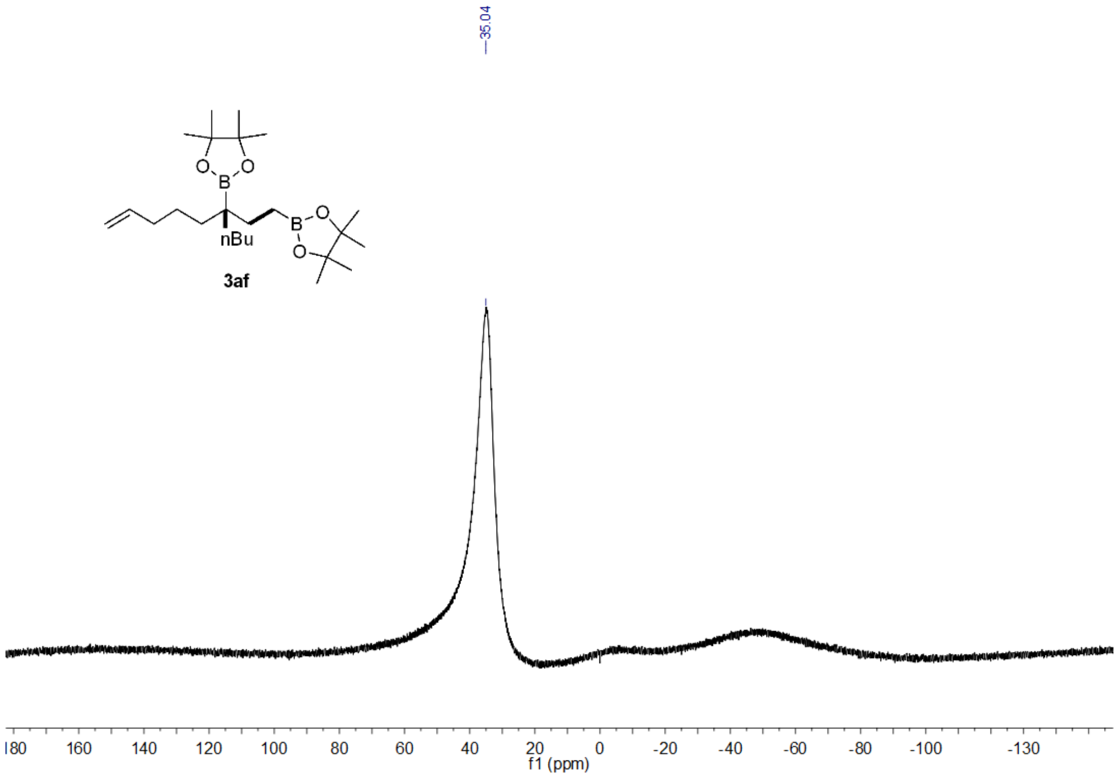
35.04
nBu
3af
f1 (ppm)

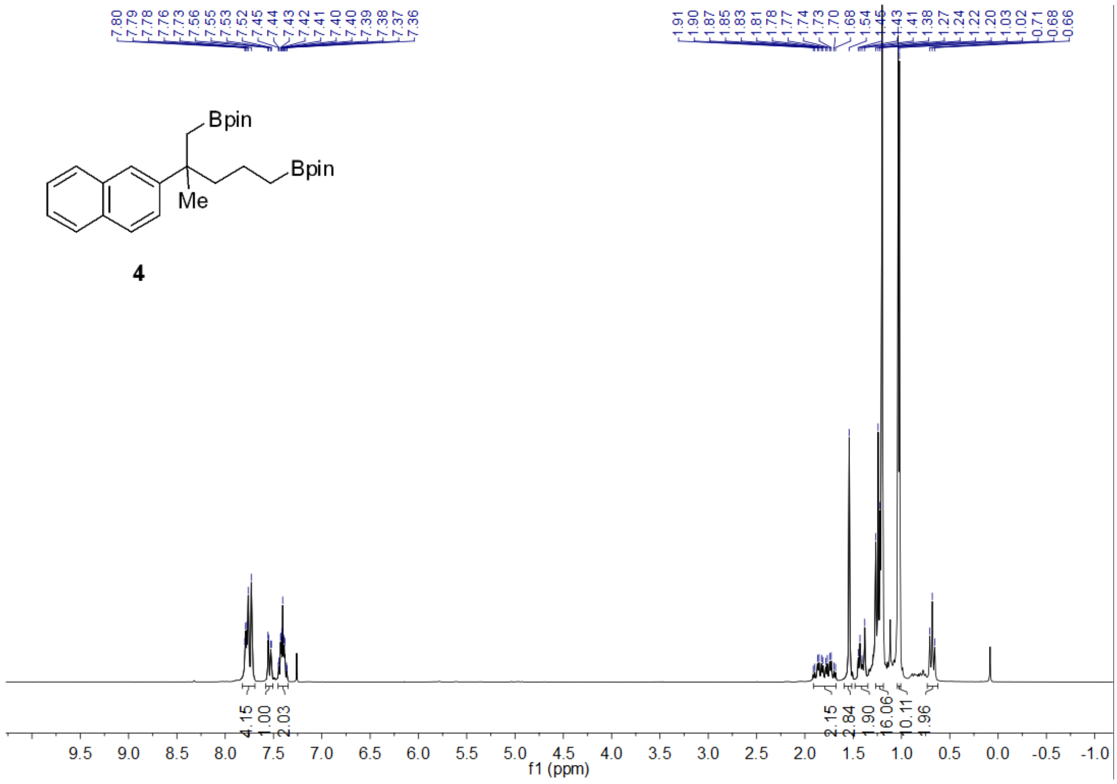
Bpin
Bpin
Me
4
f1 (ppm)

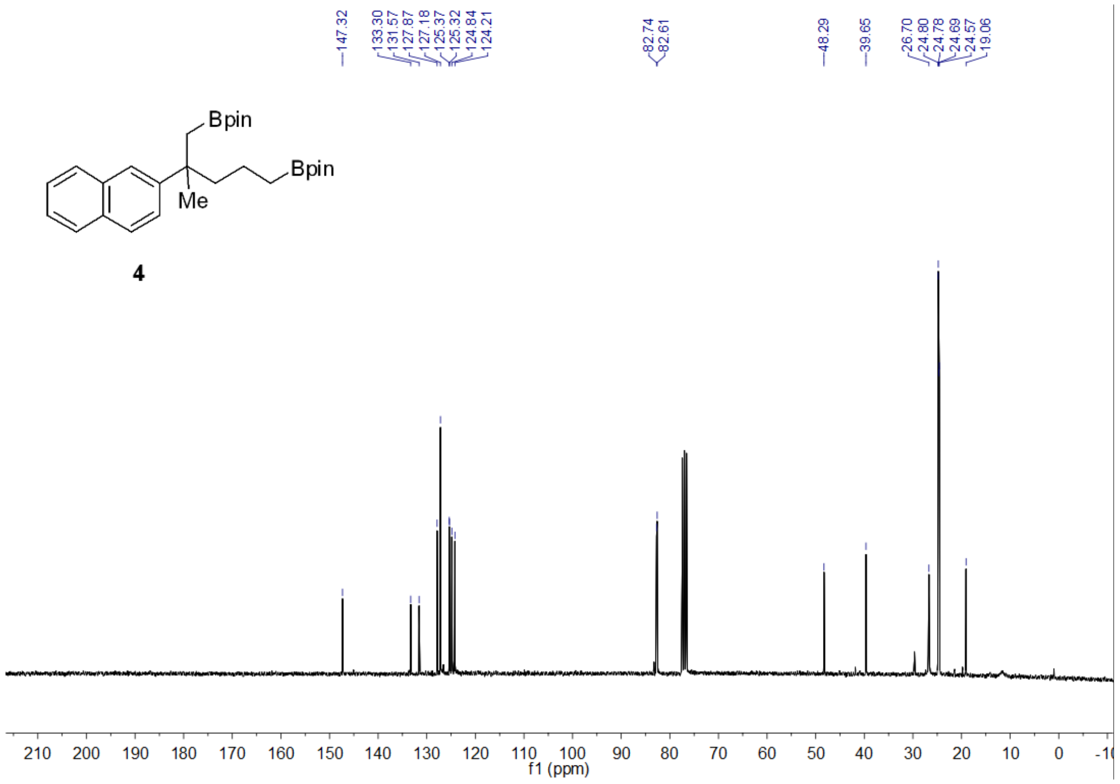

147.32
133.30
131.57
127.87
127.18
125.37
125.32
124.84
124.21
82.74
82.61
48.29
39.65
26.70
24.80
24.78
24.69
24.57
19.06
Bpin
Bpin
Me
4
210
200
190
180
170
160
150
140
130
120
110
100
90
80
70
60
50
40
30
20
10
0
f1 (ppm)

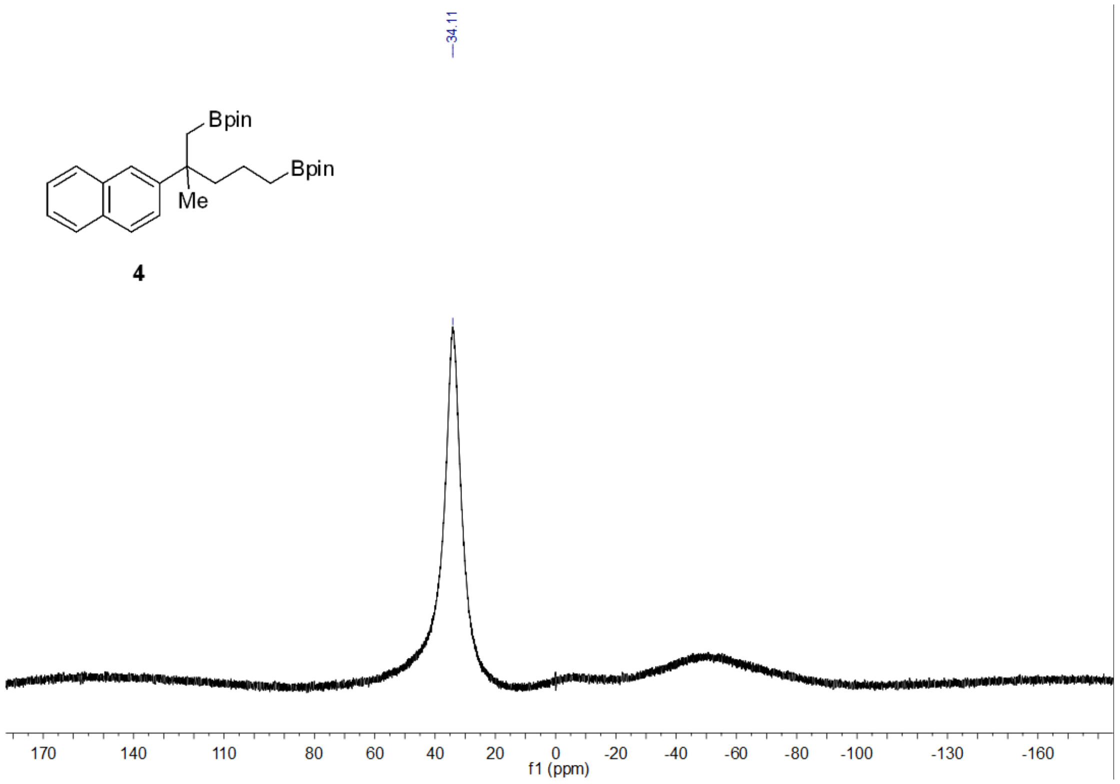

34.11
Bpin
Bpin
Me
4
170
140
110
80
60
40
20
0
-20
-40
-60
-80
-100
-130
-160
f1 (ppm)

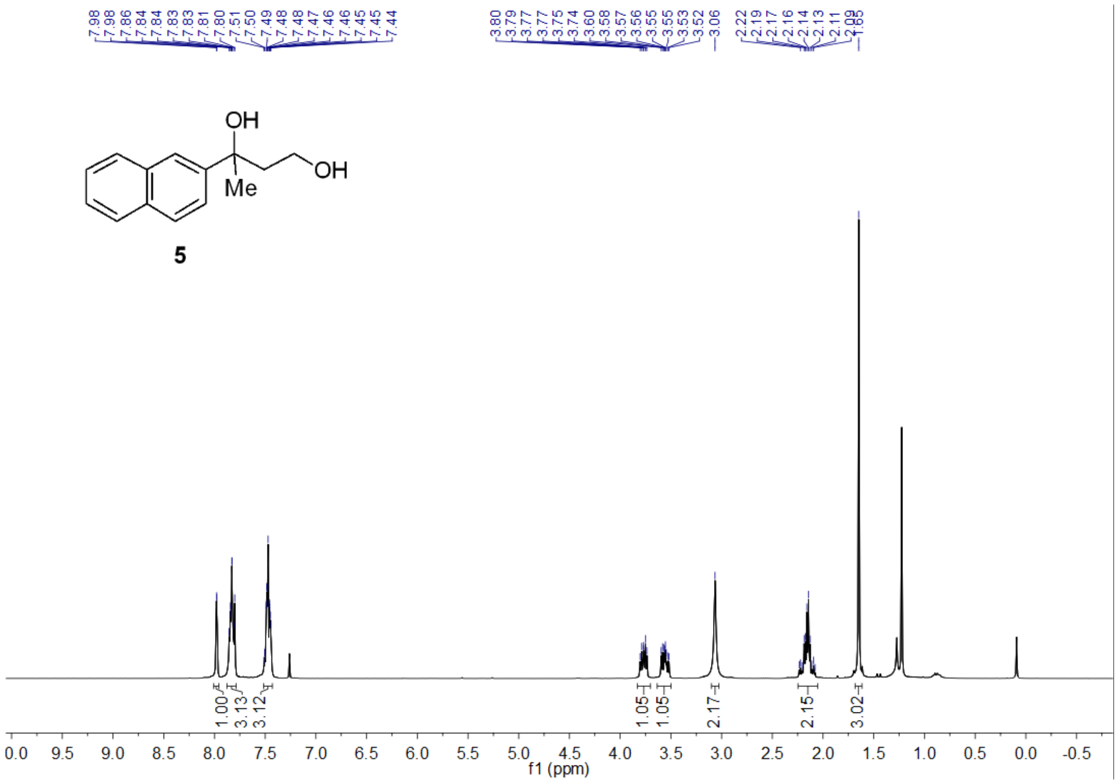
OH
OH
Me
5
1.00
3.13
3.12
1.05
1.05
2.17
2.15
3.02
f1 (ppm)

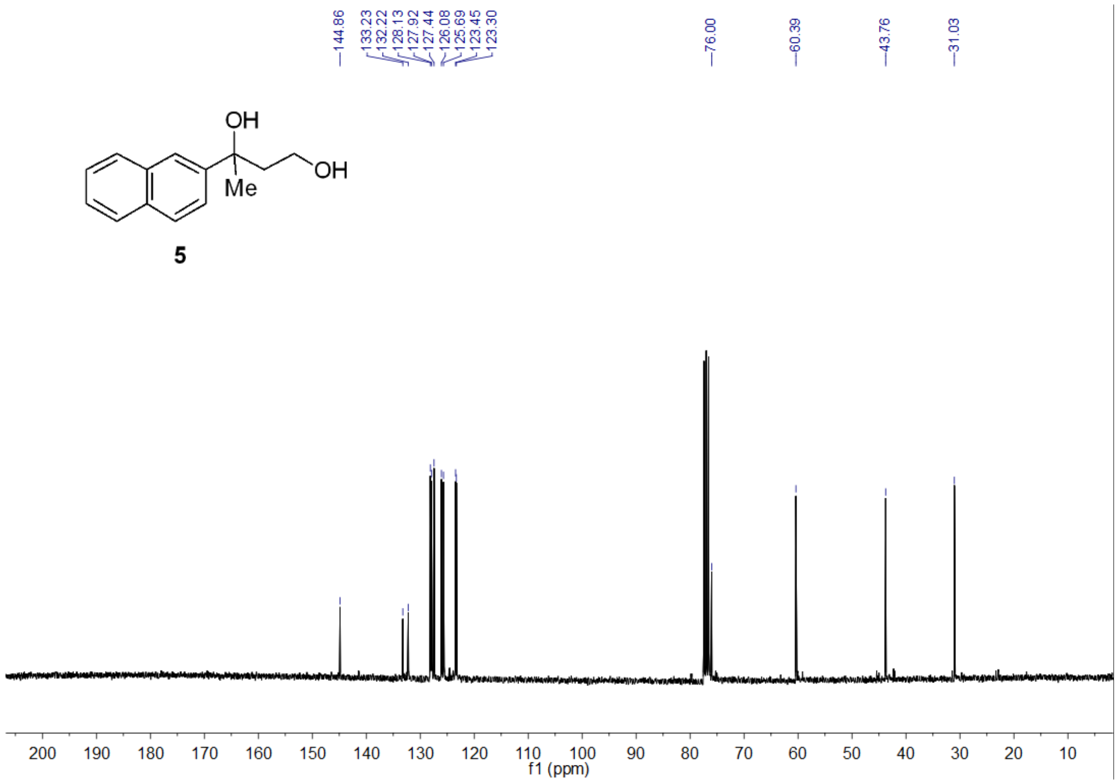
144.86
133.23
132.22
128.13
127.92
127.44
126.08
125.69
123.45
123.30
76.00
60.39
43.76
31.03
OH
OH
Me
5
f1 (ppm)

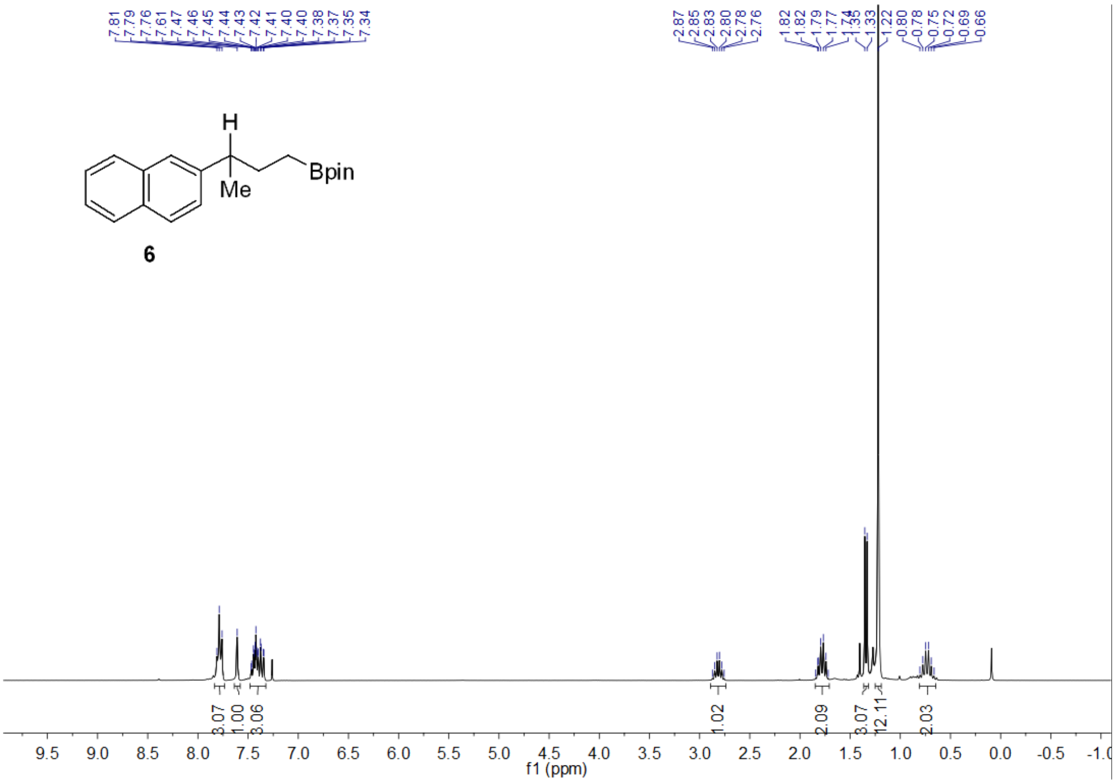
H
Me
Bpin
6
f1 (ppm)

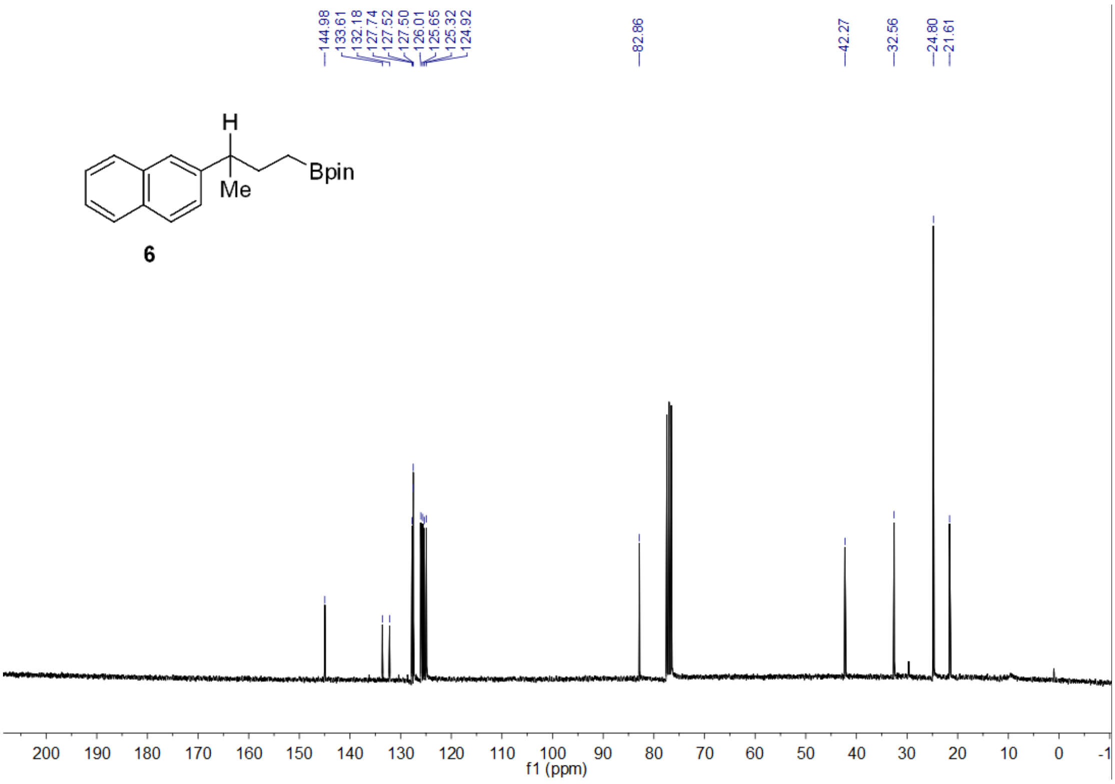
H
Me
Bpin
6
f1 (ppm)

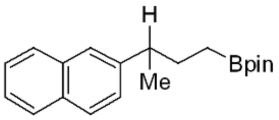
H
Me
Bpin

6

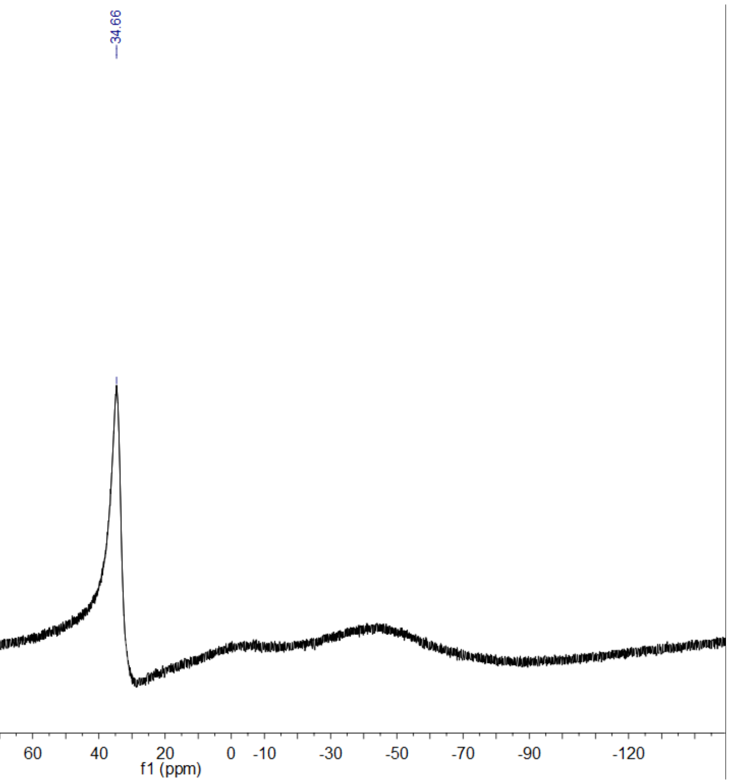
34.66
180
160
140
120
100
80
60
40
20
0
-10
-30
-50
-70
-90
-120
f1 (ppm)
